# CYP96T1 of *Narcissus* sp. *aff. pseudonarcissus* Catalyzes Formation of the *Para-Para' C-C* Phenol Couple in the Amaryllidaceae Alkaloids

**DOI:** 10.3389/fpls.2016.00225

**Published:** 2016-02-25

**Authors:** Matthew B. Kilgore, Megan M. Augustin, Gregory D. May, John A. Crow, Toni M. Kutchan

**Affiliations:** ^1^Donald Danforth Plant Science CenterSt. Louis, MO, USA; ^2^National Center for Genome ResourcesSanta Fe, NM, USA

**Keywords:** cytochrome P450, secondary metabolism, transcriptomics, Amaryllidaceae alkaloids, phenol coupling

## Abstract

The Amaryllidaceae alkaloids are a family of amino acid derived alkaloids with many biological activities; examples include haemanthamine, haemanthidine, galanthamine, lycorine, and maritidine. Central to the biosynthesis of the majority of these alkaloids is a *C-C* phenol-coupling reaction that can have *para-para', para-ortho'*, or *ortho-para'* regiospecificity. Through comparative transcriptomics of *Narcissus* sp. *aff. pseudonarcissus, Galanthus* sp., and *Galanthus elwesii* we have identified a *para-para' C-C* phenol coupling cytochrome P450, CYP96T1, capable of forming the products (10b*R*,4a*S*)-noroxomaritidine and (10b*S*,4a*R*)-noroxomaritidine from 4′-*O*-methylnorbelladine. CYP96T1 was also shown to catalyzed formation of the *para-ortho'* phenol coupled product, *N*-demethylnarwedine, as less than 1% of the total product. *CYP96T1* co-expresses with the previously characterized norbelladine 4′-*O*-methyltransferase. The discovery of CYP96T1 is of special interest because it catalyzes the first major branch in Amaryllidaceae alkaloid biosynthesis. CYP96T1 is also the first phenol-coupling enzyme characterized from a monocot.

## Introduction

**Figure 1:**
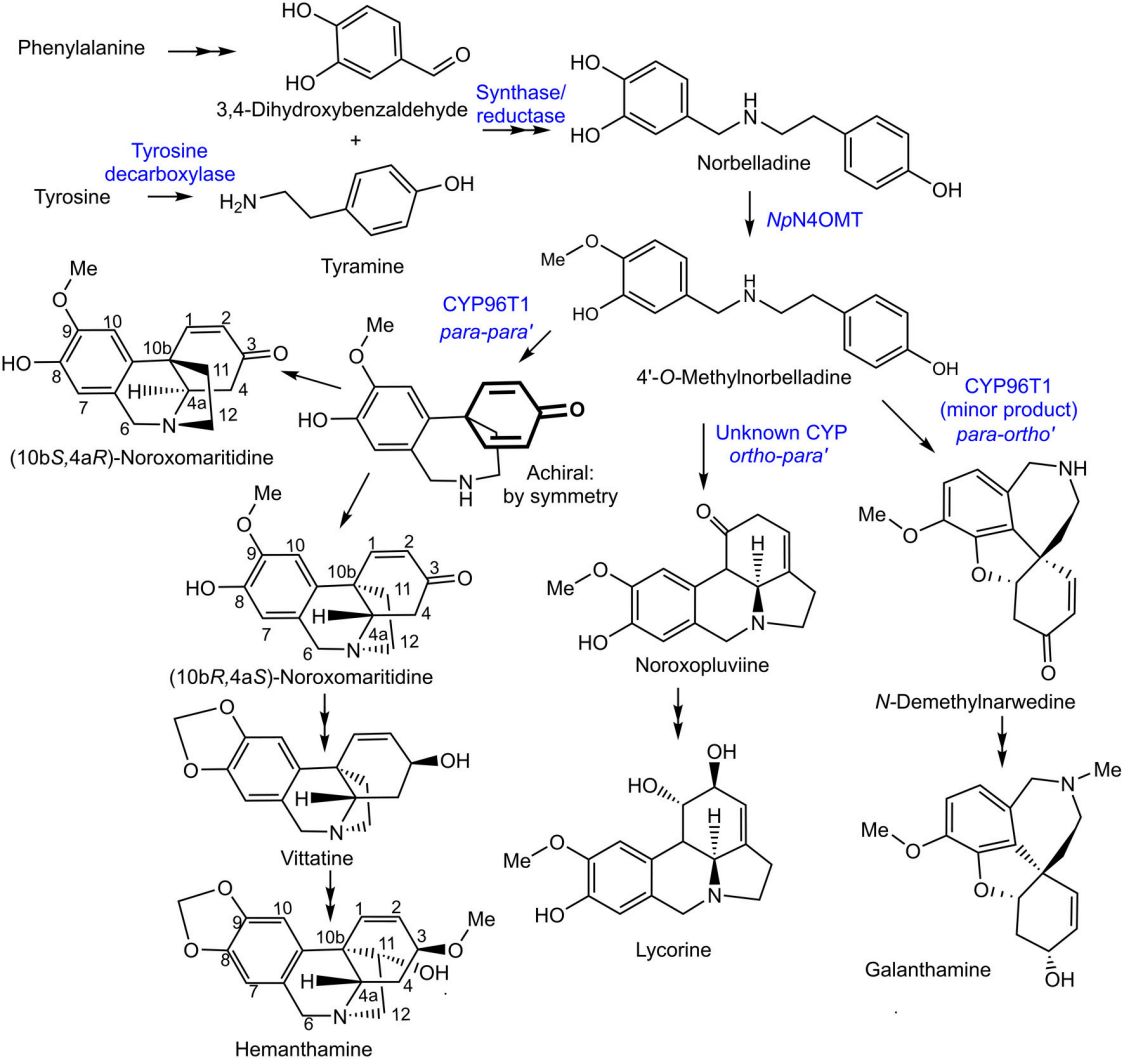
**Proposed biosynthetic pathways for representative Amaryllidaceae alkaloids directly derived from ***C-C*** phenol coupling**. The previously discovered *Np*N4OMT, the CYP96T1 discovered in this study, and potential enzyme classes involved in each step of the pathways are in blue.

**Figure 2:**
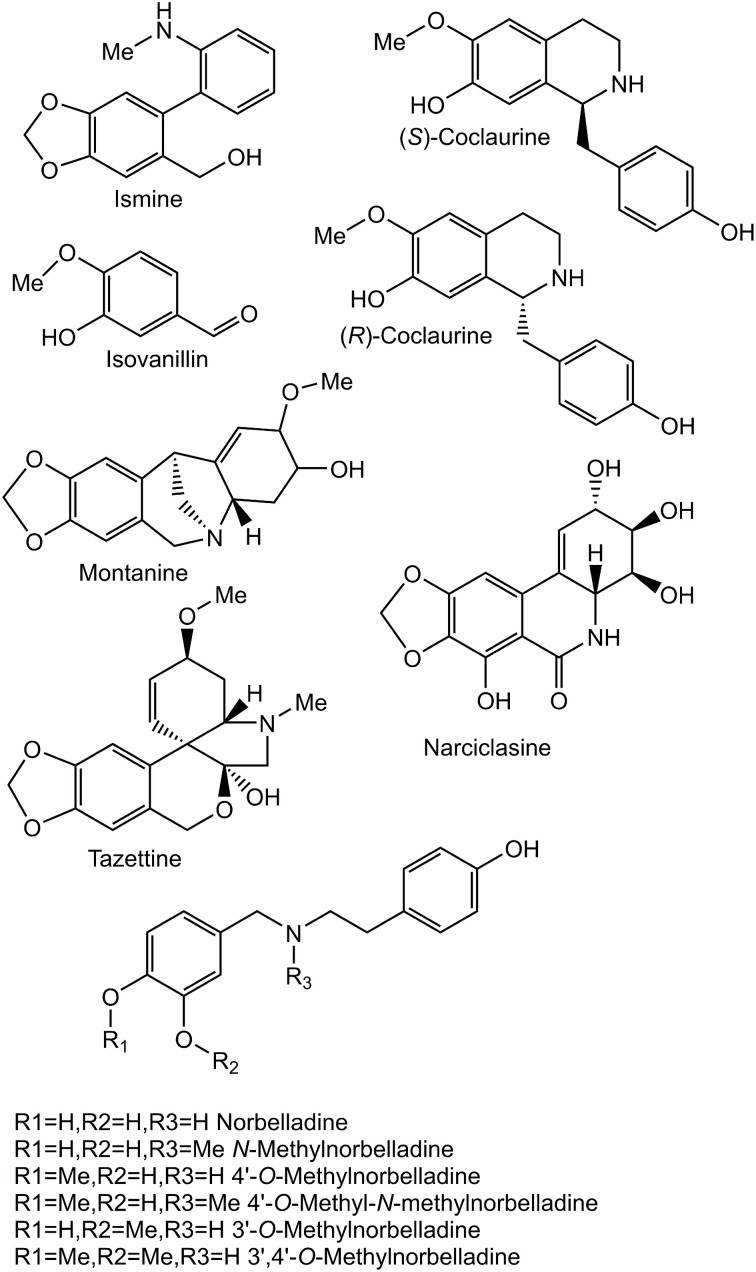
**Structures of relevant compounds**.

The Amaryllidaceae alkaloids are produced by species of Amaryllidaceae including *Narcissus* spp. (daffodil) and *Galanthus* spp. (snowdrop). Alkaloids from all major structural classes of Amaryllidaceae alkaloids have biological activities. Some of these alkaloids have potential pharmaceutical applications or are already established medicines. The alkaloid skeleton types, haemanthamine, narciclasine, tazettine, and montanine are derived from the *para-para' C-C* phenol coupled, (10b*R*,4a*S*)-noroxomaritidine, biosynthetic precursor (Wildman and Bailey, 1969; Fuganti et al., 1971; Feinstein and Wildman, 1976; see Figures 1, 2 for representative structures). Specific examples of alkaloids derived from (10b*R*,4a*S*)-noroxomaritidine include haemanthamine, maritidine, vittatine, and pretazettine. Haemanthamine has been shown to have antiproliferative and apoptotic effects on cancer cell lines and antioxidant activity in a 2,2-diphenyl-1-picrylhydrazyl scavenging assay (Oloyede et al., 2010; Van Goietsenoven et al., 2010; Havelek et al., 2014). Crinine and its derivatives are also derived from a *para-para' C-C* phenol coupling, however, the phenol-coupled product is the enantiomer, (10b*S*,4a*R*)-noroxomaritidine. Antibacterial activities have been noted for the derivatives of the (10b*S*,4a*R*)-noroxomaritidine skeleton including buphanidrine and distichamine (Cheesman et al., 2012). An example of an *ortho-para*' *C-C* phenol-coupling product is lycorine, derived from noroxopluvine. Lycorine has been documented to cause apoptosis in leukemia and multiple myeloma cancer cell lines (Liu et al., 2004, 2009; Li et al., 2007). Galanthamine is a representative derivative of the *para-ortho' C-C* phenol-coupled product *N-*demethylnarwedine and is used as an Alzheimer's treatment drug (Wilcock et al., 2003). It acts through acetylcholine esterase inhibition and nicotinic receptor binding (Irwin and Smith, 1960; Barik et al., 2005). The limited supply of some Amaryllidaceae alkaloids and diversity of biological activities make the biosynthesis of Amaryllidaceae alkaloids a topic of interest for biotechnology.

Haemanthamine is derived from the amino acids phenylalanine and tyrosine. Phenylalanine was established as a precursor by incorporation of [3-^14^C] phenylalanine into haemanthamine in *Nerine bowdenii* (Wildman et al., 1962a). The conversion of phenylalanine to 3,4-dihydroxybenzaldehyde in haemanthamine biosynthesis was clarified by feeding [3-^14^C]*trans*-cinnamic acid, [3-^14^C]*para*-hydroxycinnamic acid, [7-^14^C]benzaldehyde, [7-^14^C]*para*-hydroxybenzaldehyde, [^3^H]3,4-dihydroxybenzaldehyde, and [^3^H] threo-DL-phenylserine to *Narcissus pseudonarcissus*. Incorporation into haemanthamine from the precursors [3-^14^C]*trans*-cinnamic acid, [3-^14^C]*para*-hydroxycinnamic acid, [^3^H] 3,4-dihydroxybenzaldehyde, and trace incorporation of [7-^14^C] *para*-hydroxybenzaldehyde, but not other administered compounds, lead to the conclusion that the pathway proceeds as follows: phenylalanine is converted to *trans*-cinnamic acid, then to *para*-hydroxycinnamic acid, then to 3,4-dihydroxycinnamic acid or *para*-hydroxybenzaldehyde followed by conversion to 3,4-dihydroxybenzaldehyde (Suhadolnik et al., 1963). Observation of radiolabeled [3-^14^C] tyrosine incorporation into haemanthamine established tyrosine as a precursor (Jeffs, 1962). Tyrosine was demonstrated to contribute to the 11 and 12 carbons of haemanthamine by feeding [β-^14^C] tyrosine to *Sprekelia formosissima* and [α-^14^C] tyrosine to *Narcissus* ‘Twink’ daffodil followed by haemanthamine degradation experiments (Battersby et al., 1961b; Wildman et al., 1962b; Figure 1). These results indicate tyrosine also contributes carbons 1-4, 4a, and 10b because of their ring shape and proximity to the 11 and 12 carbons of haemanthamine. Equivalent sections of the galanthamine and lycorine carbon skeleton also originate from tyrosine (Battersby and Binks, 1960; Barton et al., 1963). Tyrosine is converted into tyramine by tyrosine decarboxylase, a well characterized enzyme in other secondary metabolite pathways (Lehmann and Pollmann, 2009).

3,4-Dihydroxybenzaldehyde and tyramine are condensed to a Schiff-base and reduced to norbelladine. The central role of norbelladine in Amaryllidaceae alkaloid biosynthesis was demonstrated by incorporation of [1-^14^C]norbelladine into haemanthamine, lycorine, and galanthamine (Barton et al., 1961, 1963; Battersby et al., 1961a,b). Next, norbelladine is methylated to 4′-*O*-methylnorbelladine. In 1963, crude enzyme extracts of *N. bowdenii* were used to perform a preliminary characterization of the 4′-*O*-methyltransferase conducting this methylation (Mann, 1963). This cation-dependent norbelladine 4′-*O*-methyltransferase (*N4OMT*) was identified in *Narcissus* sp. *aff. pseudonarcissus*, and enzymatically characterized by heterologous expression in *E. coli* (Kilgore et al., 2014). (10b*R*,4a*S*)-Noroxomaritidine is formed from the *para-para' C-C* phenol coupling of 4′-*O*-methylnorbelladine. The biosynthesis of haemanthamine deviates from alkaloids with *ortho-para'* and *para-ortho*' carbon skeletons at this branch point. The next step is thought to be a reduction of the ketone group to synthesize 8-*O*-demethylmaritidine followed by an oxide bridge formation to form vittatine. Conversion of vittatine to haemanthamine is thought to occur through hydroxylation followed by methylation (Figure 1). The conversion of vittatine to haemanthamine has been demonstrated by radiolabeling studies. The order of hydroxylation and methylation in this conversion is inferred from the presence of the hydroxylated 11-hydroxyvittatine in the *N. bowdenii* plants under investigation and the absence of the methylated (10b*R*,4a*S*)-buphanisine (Feinstein and Wildman, 1976). Haemanthamine accumulates *in planta* and is modified further to compounds such as haemanthidine and pretazettine in some Amaryllidaceae. The proposed biosynthesis of galanthamine from the *ortho-para'* product *N*-demethylnarwedine through the reduced intermediate *N*-demethylgalanthamine has been reviewed recently (Eichhorn et al., 1998; Kilgore et al., 2014).

Cytochrome P450 enzymes are a diverse enzyme family with numerous functions. Reactions catalyzed include hydroxylation, *C-C* and *C-O* phenol coupling, oxide bridge formation, carbon-carbon bond cleavage, demethylation, and rearrangements of carbon skeletons (Mizutani and Sato, 2011). Previously documented *C-C* phenol coupling by cytochrome P450 enzymes that synthesize salutaridine (CYP719A1), (*S*)-corytuberine (CYP80G2), and cyclodipeptide cyclo(l-Tyr-l-Tyr) (CYP121), suggest the *C-C* phenol coupling reactions found in Amaryllidaceae alkaloid biosynthesis are cytochrome P450 dependent (Ikezawa et al., 2008; Belin et al., 2009; Gesell et al., 2009). *C-C* phenol coupling reactions have also been documented in the human cytochrome P450s CYP2D6 and CYP3A4 with the substrate (*R*)-reticuline (Grobe et al., 2009). In addition to cytochrome P450s peroxidases and laccases are documented phenol-coupling enzymes (Schlauer et al., 1998; Davin et al., 2008; Constantin et al., 2012).

Orphan plant species are frequently of interest due to their unique metabolism. Study of this metabolism is problematic due to scarcity of genetic information, limited mutant libraries, and lack of efficient transformation methods. In addition, secondary metabolites can be phylogenetically restricted. Method development for efficient metabolic pathway elucidation in orphan species is therefore desirable. An efficient work-flow for the identification of biosynthetic genes has been previously developed and applied to Amaryllidaceae alkaloid biosynthesis. Methyltransferase transcripts correlating with galanthamine accumulation in *N*. sp. *aff. pseudonarcissus* were targeted and tested for norbelladine 4′-*O*-methyltransferase activity, leading to the discovery of the biosynthetic gene *N4OMT* (Kilgore et al., 2014). In this study, a similar work-flow is applied utilizing transcriptomic data from multiple species to identify cytochrome P450 genes that co-express with *N4OMT*. This led to the isolation and characterization of CYP96T1, which catalyzes formation of the *para-para'* and a small quantity of the *para-ortho' C-C* phenol couple with 4′-*O*-methylnorbelladine.

## Material and methods

### Plant tissue and chemicals

Leaf, bulb, and inflorescence tissues were collected from adult blooming *N*. sp. *aff. pseudonarcissus* and *Galanthus* sp. plants in St. Louis, MO and *Galanthus elwesii* in Pullman, WA. Chemicals acquired from Sigma Aldrich include ammonium acetate 97% A.C.S. reagent, HPLC grade ethanol, catalase from bovine liver, and tyramine 99%. Other chemicals purchased include ammonium acetate extra pure 25% solution in water and hydrogen peroxide 35 wt % solution in water from Acros Organics, ampicillin from GoldBio, and vanillin from Merck. Several compounds were obtained from our natural product collection including: veratraldehyde (can be acquired from Sigma), norbelladine, 4′-*O*-methylnorbelladine, (*R*)-coclaurine, and (*S*)-coclaurine (Battersby et al., 1964; Teitel and Brossi, 1968; Park, 2014; Ruiz-Olalla et al., 2015). Haemanthamine was previously isolated from *Narcissus pseudonarcissus*. Additional materials are as described previously (Gesell et al., 2009; Kilgore et al., 2014). Plant alkaloids were extracted with 70% ethanol as previously described (Kilgore et al., 2014).

### Transcriptome assembly and transcript abundance estimation

The transcriptomes assembled using ABySS and MIRA for *Galanthus* sp. and *G. elwesii* were assembled in the same manner as the previously described ABySS and MIRA *N*. sp. *aff. pseudonarcissus* transcriptome (Kilgore et al., 2014), but with 50 base paired-end reads with leaf, bulb, and inflorescence tissues. Alternative transcriptomes were made using Trinity. For these transcriptomes the same raw reads were assessed using FastQC followed by trimming with the FASTX tool kit^1^. The fastx_trimmer was used to remove the first 13 bases and fastq_quality_trimmer was used to remove all bases on the 3′ end with a Phred quality score lower than 28. Sequences below 30 bases or without a corresponding paired-end read were removed from the trimmed data set. Cleaned reads were input into the Trinity pipeline with default parameters for each data set (Haas et al., 2013). The unprocessed reads and Trinity assemblies were used with the Trinity tool RNA-Seq by Expectation-Maximization (RSEM) to obtain the transcripts per million mapped reads (TPM) for all transcripts in each tissue (leaf, bulb, and inflorescence) for each Trinity assembly. To assess quality, the following parameters were considered: the size of the resulting assembly and identification of homologs to the conserved genes *Zea mays* MADS6 (NP_001105153.1), *Arabidopsis thaliana* ribulose bisphosphate carboxylase small chain 1A (NP_176880.1), and the *Oryza sativa* ribulose-1,5-bisphosphate carboxylase/oxygenase large subunit (AAB02583.1). Assemblies and transcript expression data are deposited in the MedPlant RNA Seq Database, http://www.medplantrnaseq.org. ESTScan trained against *A. thaliana* open reading frames was used to predict peptides encoded in all Trinity assemblies (Iseli et al., 1999).

### Candidate gene identification

**Figure 3:**
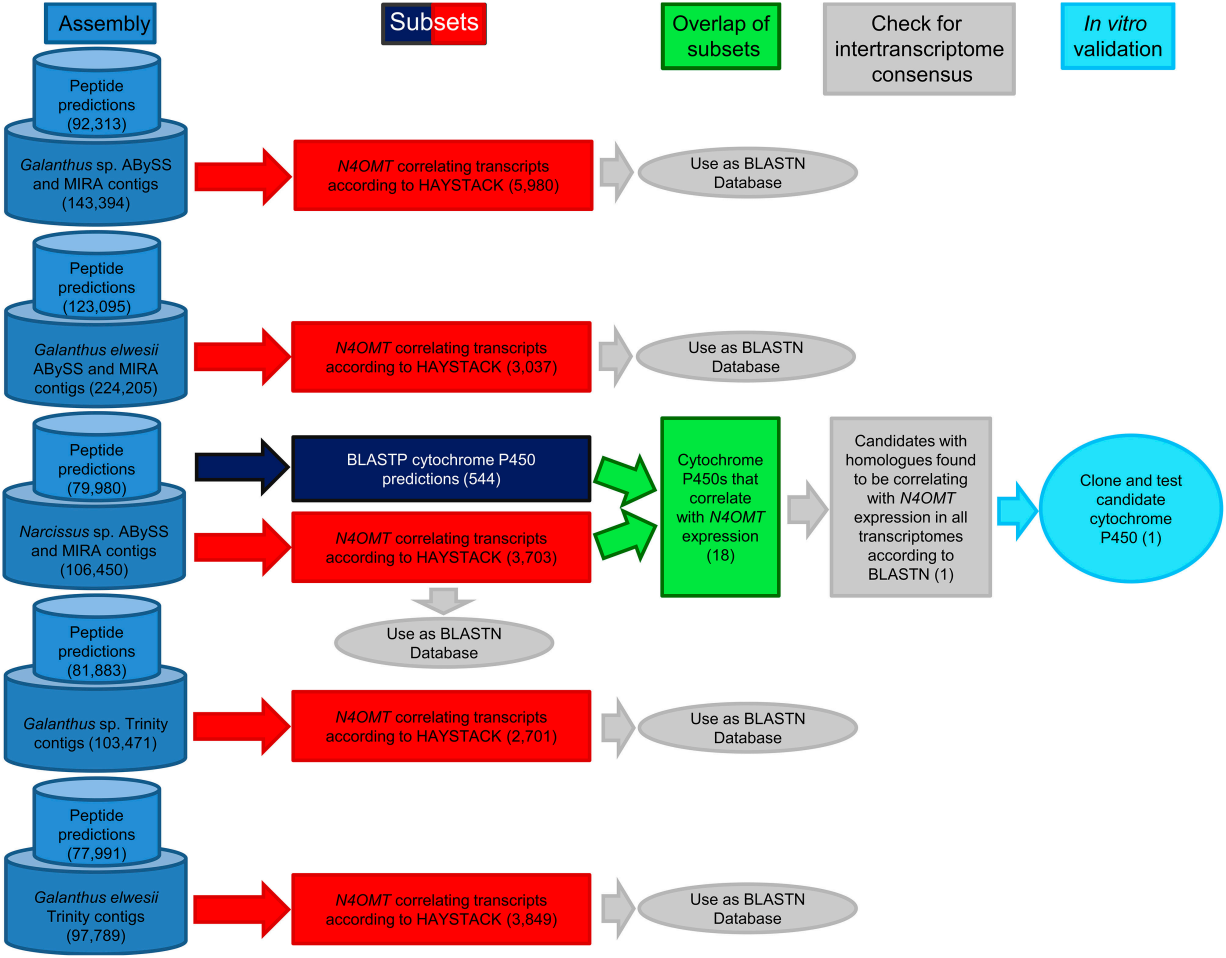
**Work-flow for identification of candidate cytochrome P450 enzymes**. Following the generation of transcriptome assemblies, cytochrome P450 enzymes were identified with BLASTP (Navy blue) and genes correlating with *N4OMT* were identified with HAYSTACK (Red). The genes present in both lists makeup the initial candidate gene list (Green). Homologs of these genes were identified in the *N4OMT* correlating lists of the other transcriptomes using BLASTN (Gray). Candidates with homologs in all five *N4OMT* correlating lists were cloned from *N*. sp. *aff. pseudonarcissus, Narcissus* sp. (light blue). The analysis for the *N*. sp. *aff. pseudonarcissus* ABySS and MIRA assembly is completely diagrammed to illustrate the process followed in every assembly. The number of transcripts selected in each step is in parentheses. The *N*. sp. *aff. pseudonarcissus* Trinity assembly is excluded from this work-flow due to its poor quality.

BLASTP with an e-value cut off of 1 × 10^−4^ was used to find homologs to known cytochrome P450 enzymes in all transcriptomes. A list of 472 unique, curated plant cytochrome P450 sequences from Dr. David Nelson, University of Tennessee, was used as a query against the ESTScan predicted peptides for each assembly (Supplementary Material 1). HAYSTACK was used to find transcripts co-expressing with *N4OMT* in each assembly (see Table 1 for *N4OMT* expression). All *Galanthus N4OMT* expression estimates were for the closest *NpN4OMT1* homolog in the assembly being used. *N*. sp. *aff. pseudonarcissus N4OMT* expression was based on the RT-PCR data for *NpN4OMT1* expression obtained previously (Kilgore et al., 2014). HAYSTACK parameters are as follows: correlation cutoff ≥ 0.8, background cutoff ≥ 1, fold cutoff ≥ 4, and *p*-value cutoff ≤ 0.05 (Mockler et al., 2007). Homologs to the *N*. sp. *aff. pseudonarcissus* cytochrome P450s co-expressing with *N4OMT* were identified using BLASTN with an e-value cut off of 1 × 10^−50^ queried against the transcripts co-expressing with *N4OMT* in the *Galanthus* spp. assemblies. For each *N*. sp. *aff. pseudonarcissus* cytochrome P450 candidate, the total number of assemblies with an *N4OMT* co-expressing BLASTN hit were determined. Candidates present in five of the five *N4OMT* co-expressing lists were considered top priority candidate genes and were cloned (Figure 3).

**Table 1:** **Models used in HAYSTACK analysis**.

**Model name**	**Leaf**	**Inflorescence**	**Bulb**
^N^*N*. sp. *aff. pseudonarcissus N4OMT* (relative units)	1	30	45
^N^*Galanthus* sp. *N4OMT* (RPM)	0.01	33.3	140
^N^*Galanthus elwesii N4OMT* (RPM)	2.24	22.6	71.7
^T^^C^*N*. sp. *aff. pseudonarcissus N4OMT* (TPM)	NA	NA	NA
^T^*Galanthus* sp. *N4OMT* (TPM)	2.42	29.0	94.7
^T^*Galanthus elwesii N4OMT* (TPM)	16.0	49.3	202
*N*. sp. *aff pseudonarcissus* galanthamine (μg/g)^*^	196	126	1070
*Galanthus* sp. galanthamine (μg/g)	0.0516	0.102	0.223
*Galanthus elwesii* galanthamine (μg/g)	1.05	0.759	1.39

N *AbySS and MIRA assembly*;

C *homolog not found*;

T *Trinity assembly*;

*RPM, reads per million; NA, not applicable*;

* *previously published in Kilgore et al. (2014)*.

### Polymerase chain reaction (PCR) and cloning

For cloning CYP96T1, designated as narcissus-20101112|22907 in the ABySS and MIRA *N*. sp. *aff. pseudonarcissus* assembly, a close homolog of CYP96T1 with the designation narcissus-20101112|13079 was identified with a complete ORF. Primers were based on the 5′ sequence of narcissus-20101112|13079 and the 3′ sequence of CYP96T1. Design of inner primers was validated by sequencing (GENEWIZ Inc.) of the outer PCR product using the following primers CYP96T forward outer (5′-ACATCCCCCCCAAAAAAAT CATAAC-3′), CYP96T reverse outer (5′-AGACCATCAATGTGAT CACCA-3′), and CYP96T reverse sequencing (5′-TGGTGAAATCG TTGAATTGGTTGT-3′). *N*. sp. *aff. pseudonarcissus* bulb cDNA was prepared as previously described (25). The outer PCR reaction contained 0.2 μM each of CYP96T forward outer and CYP96T reverse outer primers, 1 μl of bulb cDNA, 1X Phusion HF reaction buffer, 0.3 mM dNTPs, and 1 U NEB Phusion High-Fidelity DNA Polymerase in a 50 μl reaction. The PCR program parameters were as follows: 30 s 98°C 1 cycle, 15 s 98°C, 30 s 52°C, 2 min 72°C 35 cycles, and 5 min 72°C 1 cycle. Inner PCR parameters were identical except primers used were CYP96T forward inner (5′-aattGCGGCCGCATGGCCACT TCTTCTTCAGCA-3′) and CYP96T reverse inner (5′-aattTCTAGATCAC ATGACTGATCTCTTTCT-3′) adding NotI and XbaI restriction sites, respectively (underlined), the outer PCR reaction was added as template as opposed to cDNA, and the PCR cycles were reduced to 25.

The inner PCR product and pVL1392 vector were digested with NEB NotI and XbaI followed by PCR purification with the QIAquick PCR Purification Kit and ligation with NEB T4 DNA ligase according to manufacturer's instructions. The resulting construct was transformed into DH5α *E. coli* chemically competent cells. Recombinant bacteria were selected on Luria-Bertani 1.5% agar plates with 50 μg/ml ampicillin.

Transformants were screened by colony PCR with the following components: 0.67 μM polyhedrin forward (5′-AAAATGATA ACCATCTCGC-3′) and polyhedrin reverse (5′-GTCC AAGTTTCCCTGTAGA-3′) primers, ThermoPol Reaction buffer 1X, 0.2 mM dNTPs, and 1 U of NEB Taq DNA polymerase in a volume of 20 μl. The PCR parameters were as previously described (Kilgore et al., 2014). Plasmid minipreps of 5 ml cultures in LB supplemented with 50 μg/ml ampicillin were prepared using the QIAGEN QIAprep Spin Miniprep Kit according to manufacturer's instructions and sequenced with polyhedrin forward and reverse primers by Eurofins genomics. All reproducible sequences were named by Dr. David Nelson, University of Tennessee. These names were CYP96T1, CYP96T2, and CYP96T3 with the GenBank accession numbers KT693311, KT693312, and KT693313, respectively. The closest biochemically characterized homolog to the resulting CYP96T1 clone, CYP96A15, was identified in the UniProt database with BLASTP. CYP96T1, CYP96T2, CYP96T3, the original CYP96T1 sequence, and CYP96A15 were aligned with MUSCLE in the CLC main workbench version 6.9.1 (Edgar, 2004).

### Protein expression

Co-transfection of CYP96T1 in pVL1392 and Baculogold baculoviurus (BD Biosciences), viral amplification, protein expression, and microsome preparation in *Spodoptera frugiperda* Sf9 cells was performed as previously described (Gesell et al., 2009). Microsomes of Sf9 cells expressing CYP96T1 were solubilized with 0.17% emulgen 913 at 4°C for 15 min followed by centrifugation (15,000 × g for 15 min) before obtaining the CO difference spectra. The resulting CYP96T1 concentration was used to calculate concentration of CYP96T1 in all subsequent Sf9 cell cultures. CYP96T1 was always co-expressed with *Eschscholzia californica* cytochrome P450 reductase (CPR) (Gesell et al., 2009). For a negative control CPR was expressed without CYP96T1.

### 3′-*O*-methylnorbelladine and 3′,4′-*O*-dimethylnorbelladine synthesis

For the synthesis of 3′-*O*-methylnorbelladine sodium cyanoborohydride (50 mM), vanillin (5 mM), and tyramine (5 mM) were mixed in 2.5 ml anhydrous methanol for 2 days at room temperature. For synthesis of 3′,4′-*O*-dimethylnorbelladine, sodium cyanoborohydride (50 mM), veratraldehyde (5 mM), and tyramine (5 mM) were mixed in 2.5 ml anhydrous methanol for 2 days at room temperature. The reaction mix was then taken to dryness under N_2_. The resulting material was suspended in 200 μl of 1 M NaCO_3_ pH 9.5 and extracted twice with 400 μl of ethyl acetate by vortexing for 1 min, followed by centrifugation at 16,100 × g for 2 min at room temperature. Ethyl acetate extractions were pooled and dried under vacuum. Extractions were re-suspended in 10% acetonitrile and 0.1% formic acid and purified by fractionation using a Waters fraction collector III and Waters 1525 binary HPLC pump as previously described (Kilgore et al., 2014). 3′-*O*-methylnorbelladine was collected at 9 min for ~1 min and 3′,4′-*O*-dimethylnorbelladine at 11.5 min for ~1 min. Purified compound was dried under vacuum, re-suspended in H_2_O and quantified with a 4′-*O*-methylnorbelladine standard curve using peak area by HPLC with the method stated above.

### Enzyme assays

Screening assays contained 30 mM KPO_4_ pH 8.0, 1.25 mM NADPH, 10 μM substrate, and 70 μl of virus infected Sf9 cell suspension in 200 μl total volume. The assays were incubated for 2–4 hr at 30°C. 4′-*O*-metylnorbelladine was tested for all CYP96T variants. CYP96T1 was used for substrate specificity tests on norbelladine, *N*-methylnorbelladine, 4′-*O*-methyl-*N*-methylnorbelladine, 3′-*O*-methylnorbelladine, 3′,4′-*O*-dimethylnorbelladine, haemanthamine, (*S*)-coclaurine, (*R*)-coclaurine, and mixed (10b*S*,4a*R*)- and (10b*R*,4a*S*)-noroxomaritidine (see Figures 1, 2 for chemical structures). Assays derivatized with sodium borohydride were incubated 2 hr at 30°C followed by addition of 0.5 volumes 0.5 M sodium borohydride in 0.5 M sodium hydroxide and incubated 30 min at RT. The CYP96T1 assay resolved on a Chiral-CBH column and assays measured with HPLC used fresh CYP96T1 and CPR expressing Sf9 cell protein prepared using re-amplified virus. Kinetic assays were run in the linear time range for each substrate in 200 mM KPO_4_ pH 6.5 buffer with 40 μl assays. Assays testing the effects of added H_2_O_2_ (0.1%) and/or catalase (0.1 mg/ml) were done for 20 min at 30°C with 10 μM 4′-*O*-methylnorbelladine, 1 mM NADPH, and 200 mM KPO_4_ pH 6.5 buffer. Product for overnight enzyme assays of (*R*)-coclaurine, (*S*)-coclaurine, and 4′-*O*-methyl-*N*-methylnorbelladine were quantified at 277 nm against a noroxomaritidine standard curve with the same HPLC method and setup used for 3′-*O*-methylnorbelladine and 3′,4′-*O*-dimethylnorbelladine isolation. These products were subsequently used as standards for quantifying kinetic assays. *K*_*m*_ and *k*_*cat*_ values were estimated using R version 3.2.0 with nonlinear fitting.

### LC-MS/MS

Enzyme assays on all substrates were extracted as previously described and run on a QTRAP 4000 coupled to a IL-20AC XR prominence liquid auto sampler, 20AD XR prominence liquid chromatograph and Phenomenex Luna 5 μm C8(2) 250 × 4.60 mm column. HPLC gradient and MS settings were as previously described (Kilgore et al., 2014). Assay specific MS/MS parameters are presented in Table 2. Multiple Reaction Monitoring (MRM) parameters for relative quantification of (10b*S*,4a*R*)- and (10b*R*,4a*S*)-noroxomaritidine, *N*-demethylnarwedine, narwedine, and the two unknown compounds are presented in Table 3. For analysis of product chirality, a Chrom Tech, Inc. Chiral-CBH 100 × 4.0 mm, 5 μM column was used with a 30 min isocratic flow of 2.5% HPLC grade ethanol and 10 mM ammonium acetate with pH adjusted to 7.0 with ammonium hydroxide. Kinetic assays were quantified with an isocratic flow 20% acetonitrile and 0.08% formic acid with the Phenomenex Luna 5 μm C8(2) 250 × 4.60 mm column connected to the same QTRAP 4000 setup. MRM transitions used in kinetics and H_2_O_2_/catalase assays were 284.1/223.0 *m/z* for the (*S*)-coclaurine and (*R*)-coclaurine products, 286.1/271.0 *m/z* for the 4′-*O*-methyl-*N*-methylnorbelladine *para-para*' product, and 272.3/229.0 *m/z* for noroxomaritidine.

**Table 2:** **MS/MS parameters for substrate tests**.

**Substrate**	**Product specific parameters (Collision energy) (Declustering potential) (First quadrupole *m/z*)**	**Substrate specific parameters (Collision energy) (Declustering potential) (First quadrupole *m/z*)**
4′-*O*-Methylnorbelladine	(35)(70)(272.30)	(20)(60)(274.30)
4′-*O*-Methyl-*N*-methylnorbelladine	(35)(70)(286.20)	(20)(60)(288.30)
3′*-O*-Methylnorbelladine	(35)(70)(272.30)	(35)(60)(274.30)
3′,4′-*O*-Dimethylnorbelladine	(35)(70)(286.20)	(20)(60)(288.30)
Norbelladine	(35)(60)(258.00)	(15)(50)(260.00)
*N-*Methylnorbelladine	(35)(70)(272.30)	(20)(60)(274.30)
Haemanthamine	(35)(70)(300.12)/(35)(70) (318.13)^HO^	(35)(70)(302.14)
(10b*S*,4a*R*)- and (10b*R*,4a*S*)-Noroxomaritidine	(35)(70)(270.30)/(35)(70) (288.30)^HO^	(35)(70)(272.30)
Isovanillin and tyramine	(20)(40)(290.30)^a^/(20)(60)(272.20)^b^/(35)(70)(270.20)^c^	(20)(60)(138.20)/(20)(50)(153.20)
(*S*)-Coclaurine	(35)(70)(284.30)/(30)(60) (570.60)^dim^	(20)(70)(286.30)
(*R*)-Coclaurine	(35)(70)(284.30)/(30)(60) (570.60)^dim^	(20)(70)(286.30)
4′-*O-*Methylnorbelladine assays followed by sodium borohydride derivatization	(20)(60)(274.30)	(20)(60)(274.30)

HO *hydroxylation monitored*;

dim *dimer formation monitored*;

a *C-C phenol coupling with no amine aldehyde condensation*;

b *amine aldehyde condensation/amine aldehyde condensation with C-C phenol coupling and a reduction*;

c *amine aldehyde condensation with C-C phenol coupling*.

**Table 3:** **MS/MS parameters used in MRM studies**.

**Compound (*C-C* phenol coupling type)**	**MRM parameters (Collision energy) (Declustering potential) (First quadrupole *m/z*) (Second quadrupole *m/z*) (Retention time min)**
Noroxomaritidine(*para'-para*)	(35)(70)(272.3)(229.0)(5.3)
*N*-Demethylnarwedine(*para'-ortho*)	(35)(70)(272.3)(201.0)(7.9)
4′-*O*-Methyl-*N*-methylnorbelladine assay unknown 1(potential *para'-para* product)	(35)(70)(286.1)(271.0)(4.7)
4′-*O*-Methyl-*N*-methylnorbelladine assay unknown 2 (potential *ortho'-para* product)	(30)(70)(286.1)(243.0)(7.5)
Narwedine(*para'-ortho*)	(30)(70)(286.1)(229.1)(8.1)

Quantification of galanthamine and haemanthamine found in *N*. sp. *aff. pseudonarcissus* bulb scales was performed with a QTRAP 6500, the Phenomenex Luna 5 μm C8(2) 250 × 4.60 mm column, and an isocratic flow method 0.8 ml/min 0.08% formic acid and 20% acetonitrile. The MRM transitions used for galanthamine were *m/z* 288.1/270.0 collision energy 30 and declustering potential 70; *m/z* 288.1/213.1 collision energy 30 and declustering potential 70. For haemanthamine, the MRM transitions were *m/z* 302.1/270.1 collision energy 30 and declustering potential 70; *m/z* 302.1/252.1 collision energy 30 and declustering potential 70.

## Results

### Transcriptome assembly and transcript abundance estimation

Key statistics for each transcriptome including total number of transcripts, maximum transcript length, and average transcript length are summarized in Table 4. ABySS and MIRA assemblies were found to have a high number of incomplete ORFs. This was problematic for cloning and highlighted the potential problem of unannotated transcripts and inaccurate expression estimates in transcripts with short assemblies. Quality processed reads were reassembled with Trinity to provide alternate information on the same transcripts. These assemblies provided additional sequence information with comparable expression estimates. The *N*. sp. *aff. pseudonarcissus* Trinity assembly resulted in a large number of contigs but lacked well-characterized genes, such as ribulose bisphosphate carboxylase small chain 1A and *NpN4OMT1*. In addition, the maximum contig length was 40,450, well above the expected size range. For these reasons, further analysis of the *N*. sp. *aff. pseudonarcissus* Trinity assembly was abandoned. The other assemblies have comparable statistics regardless of assembly method. Because these assemblies are complementary to each other, both sets of *Galanthus* assembles were used for subsequent analysis (Table 4).

**Table 4:** **Transcriptome statistics**.

	*N*. sp. *aff. pseudonarcissus* AbySS and MIRA^*^^!^	*Galanthus* sp. AbySS and MIRA^*^	*Galanthus elwesii* AbySS and MIRA^*^	***N*. sp. *aff. pseudonarcissus* Trinity^#^**	***Galanthus* sp. Trinity^#^**	***Galanthus elwesii* Trinity^#^**
Sequences (number of sequences)	106,450	143,394	224,205	608,439	103,471	97,789
Longest (bp)	13,381	15,365	19,356	40,450	13,629	13,055
N50 (bp)	1130	1418	1330	931	1044	1139
Mean (bp)	551	664	602	671	723	777
Median (bp)	248	271	245	430	481	528

* *Assemblies were set to have a minimum cut off of 100 bp*;

# *assemblies were set to have a minimum cut off of 201 bp*;

! *assembly previously reported in Kilgore et al. (2014)*.

### Candidate gene identification and cloning

**Figure 4:**
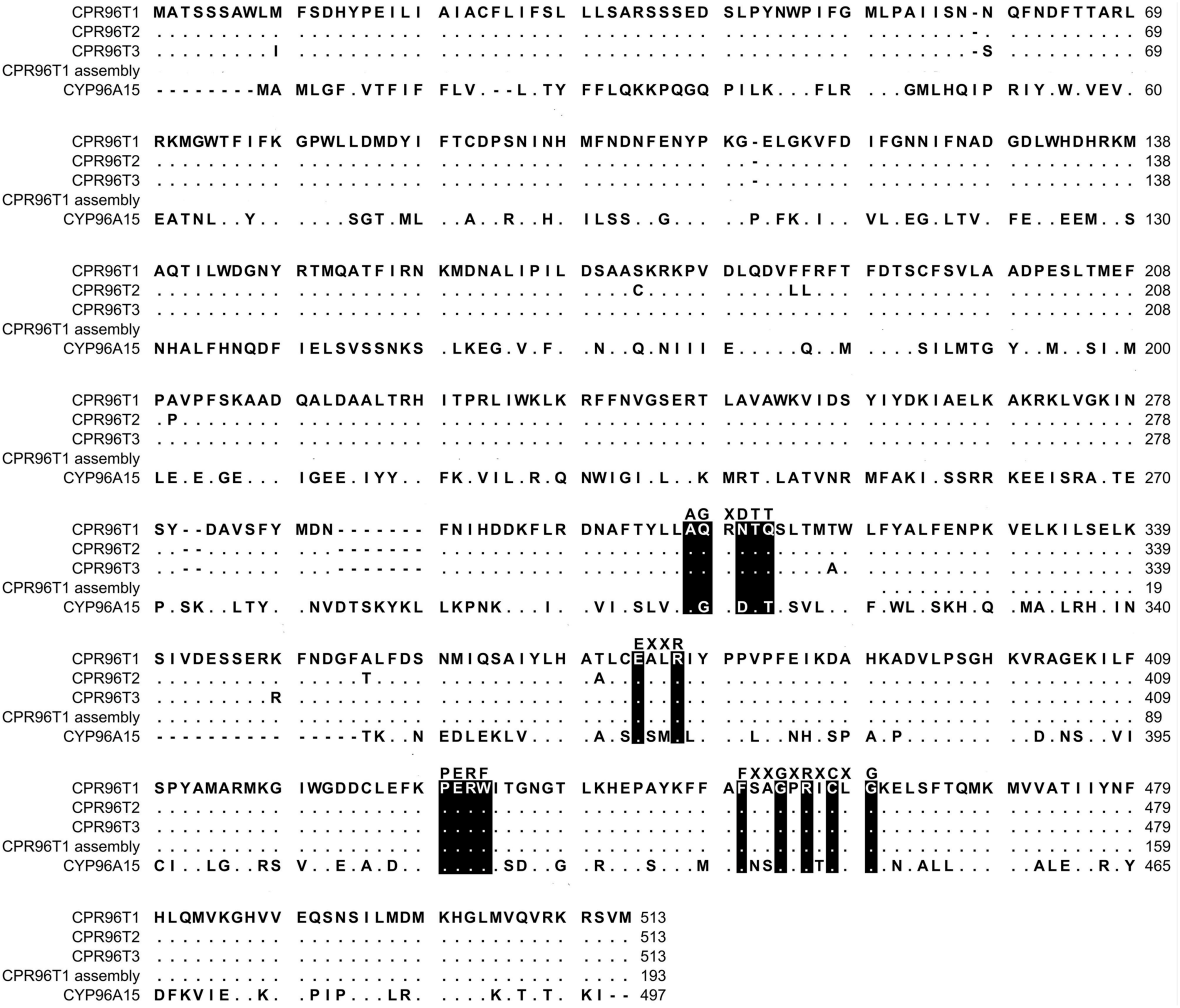
**MUSCLE alignment of protein sequences**. The sequences include CYP96T1, CYP96T2, CYP96T3, the CYP96T1 from the *N*. sp. *aff. pseudonarcissus* ABySS and MIRA assembly, and CYP96A15 from *A. thaliana* (Q9FVS9). Simplified consensus motifs for cytochrome P450 enzymes are placed above the corresponding color inverted CYP96T1 sequence. Dots are exact matches to CYP96T1 and dashes are gaps.

The pattern-matching algorithm HAYSTACK was used to identify transcripts that co-express with *N4OMT*. *N4OMT* is the only validated gene involved in Amaryllidaceae alkaloid biosynthesis to date. It is positioned in the pathway just prior to the *C-C* phenol-coupling step and co-accumulates with the Amaryllidaceae alkaloid galanthamine across *Narcissus* and *Galanthus* species (Table 1). Therefore, *N4OMT* gene expression is a suitable choice to serve as a model for analysis of co-expressing transcripts encoding additional Amaryllidaceae alkaloid biosynthetic genes. Since the *C-C* phenol-coupling enzyme is targeted herein, BLASTP was used to find transcripts that encode putative cytochrome P450 enzymes. The resulting 544 *N*. sp. *aff. pseudonarcissus* cytochrome P450 protein sequences were compared to the list of 3,704 *N4OMT* co-expressing transcripts identified by HAYSTACK. This resulted in the identification of 18 *N4OMT* co-expressing cytochrome P450 transcripts in the *N*. sp. *aff. pseudonarcissus* assembly. These transcripts were shown by BLASTP to be closely homologous to a diversity of cytochrome P450 families including CYP71, 72 73, 81, 86, 88, 90, 94, 95, and 704 (Table S1). One of the CYP86 candidates was re-annotated as a CYP96 after a closer examination of the full sequence and placed in a novel cytochrome P450 subfamily, CYP96T, by Dr. David Nelson, University of Tennessee. The *Galanthus* assemblies were interrogated using these 18 sequences to identify close homologs. This allowed for selection of the cytochrome P450 transcripts that consistently co-expressed with *N4OMT* across species in all assemblies. One candidate, (*CYP96T1*) co-expressed with *N4OMT* in all assemblies and was investigated further in *N*. sp. *aff. pseudonarcissus* where its correlation was 0.9995. A close homolog to *CYP96T1* with 99% identity in shared ORF sequence and the first 67 bases of the 3′ UTR was identified. In contrast to *CYP96T1*, this transcript was complete at the 5′ end of the ORF and contained 5′ UTR sequence information. This allowed the incomplete 5′ region of *CYP96T1* to be predicted by comparison. The PCR product generated with outer primers was sequenced and the inner primer sequences were found not to deviate from the assembly prediction. A clone was acquired with no conflicts to the previously known *CYP96T1* sequence and was used for functional characterization. Two additional variants were cloned reproducibly. The closest biochemically characterized homolog to *CYP96T1* was *CYP96A15* from *A. thaliana* (Q9FVS9) (Figure 4).

### Enzyme assays and analysis by LC-MS/MS

**Figure 5:**
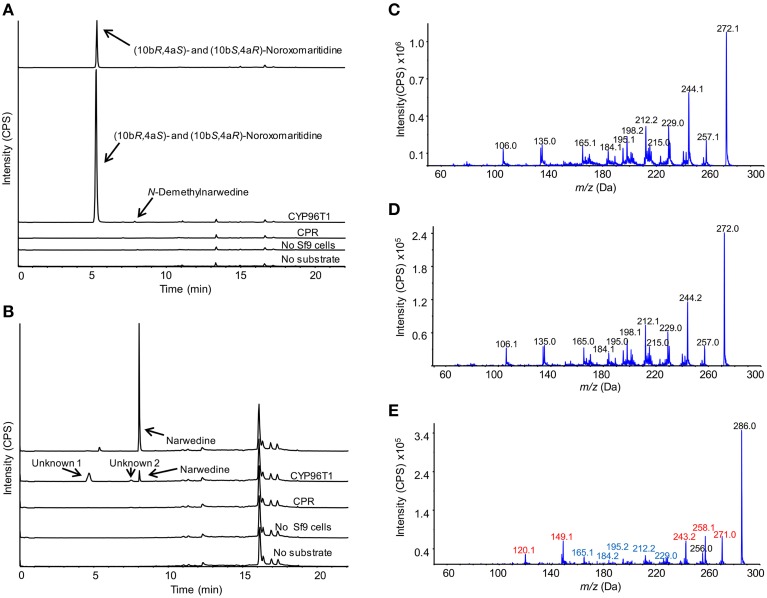
**LC-MS/MS enhanced product ion scan (EPI) monitoring the ***C-C*** phenol coupling of 4′-***O***-methylnorbelladine and 4′-***O***-methyl-***N***-methylnorbelladine in CYP96T1 assays**. Arrows indicate peaks unique to Sf9 cell containing assays with substrate present. **(A)** Standards and assays with 4′-*O*-methylnorbelladine as the substrate. Sample runs top to bottom (10b*R*,4a*S*)- and (10b*S*,4a*R*)-noroxomaritidine standard (1 μM), CYP96T1 assay, CPR assay, assay without Sf9 cells, and CYP96T1 assay without 4′-*O-*methylnorbelladine. **(B)** Standards and assays with 4′-*O*-methyl-*N*-methylnorbelladine as the substrate. Top to bottom narwedine standard, CYP96T1 assay, CPR assay, assay without Sf9 cells, and assay without 4′-*O-*methylnorbelladine. **(C)** EPI of the (10b*R*,4a*S*)- and (10b*S*,4a*R*)-noroxomaritidine standard. **(D)** EPI of the CYP96T1 (10b*R*,4a*S*)- and (10b*S*,4a*R*)-noroxomaritidine product with 4′-*O*-methylnorbelladine as substrate. **(E)** EPI of the CYP96T1 *para-para'* product (Unknown 1) with 4′-*O*-methyl-*N*-methylnorbelladine as substrate. Red fragments indicate the addition of one methyl group, 14 *m/z*, relative to (10b*R*,4a*S*)- and (10b*S*,4a*R*)-noroxomaritidine and blue fragments indicate the same *m/z* as (10b*R*,4a*S*)- and (10b*S*,4a*R*)-noroxomaritidine fragments. Intensity is presented in counts per second (CPS).

**Figure 6:**
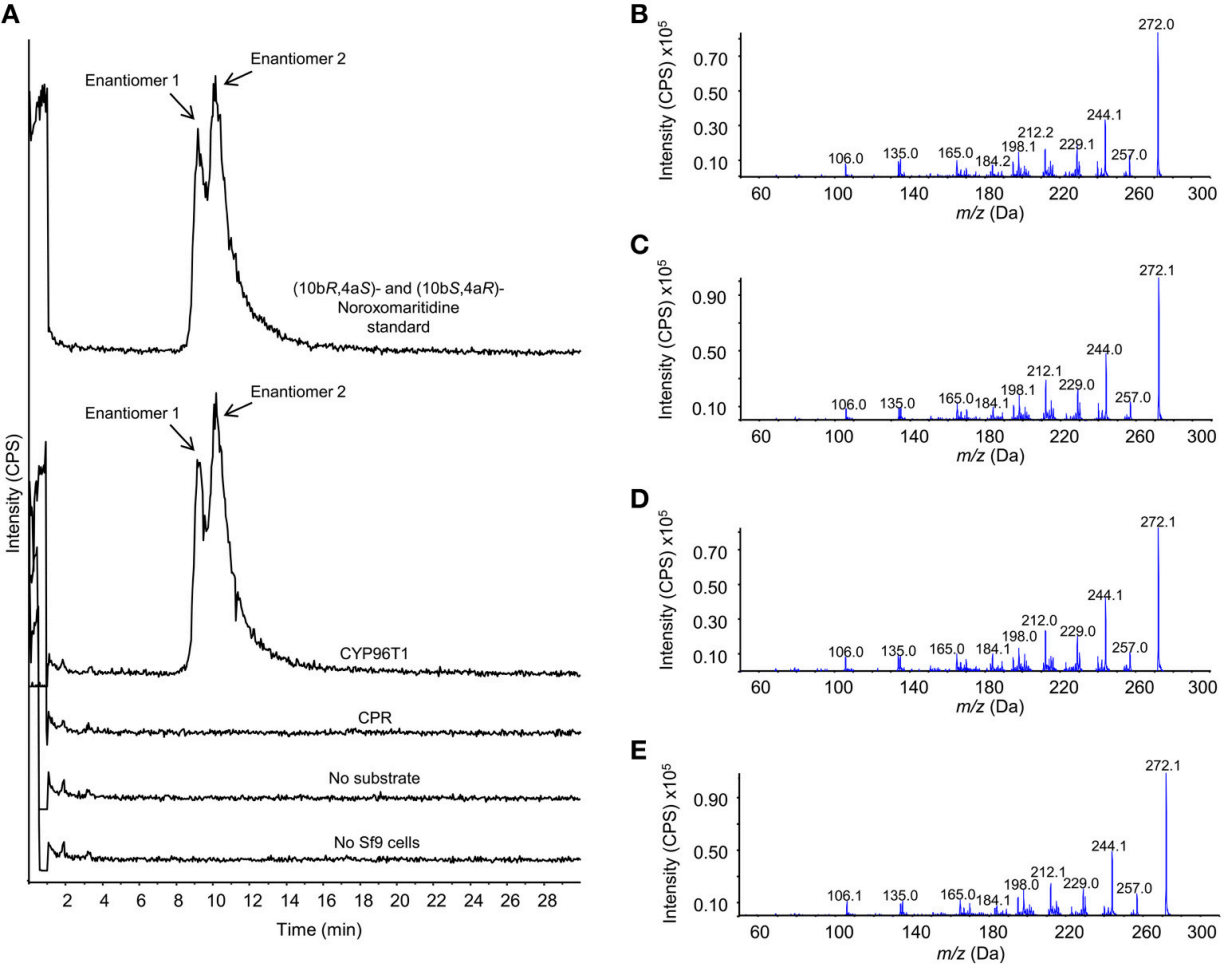
**Chromatographic separation and MS/MS analysis of the primary 4′-***O***-methylnorbelladine products (10b***S***,4a***R***)- and (10b***R***,4a***S***)-noroxomaritidine**. The enantiomers (10b*S*,4a*R*)- and (10b*R*,4a*S*)-noroxomaritidine were chromatographically separated with a chiral-CBH column and analyzed by MS/MS using an enhanced product ion (EPI) scan. **(A)** Samples, top to bottom: (10b*R*,4a*S*)- and (10b*S*,4a*R*)-noroxomaritidine standard, CYP96T1 assay, CPR assay, CYP96T1 assay without 4′-*O*-methylnorbelladine substrate and no Sf9 cells assay. **(B)** EPI fragmentation pattern for enantiomer 1 of (10b*R*,4a*S*)- and (10b*S*,4a*R*)-noroxomaritidine. **(C)** EPI fragmentation pattern for enantiomer 2 of (10b*R*,4a*S*)- and (10b*S*,4a*R*)-noroxomaritidine. **(D)** EPI fragmentation pattern for enantiomer 1 in the CYP96T1 assay with 4′-*O*-methylnorbelladine as substrate. **(E)** EPI fragmentation pattern for enantiomer 2 in the CYP96T1 assay with 4′-*O*-methylnorbelladine as substrate. Intensity is presented in counts per second (CPS).

**Figure 7:**
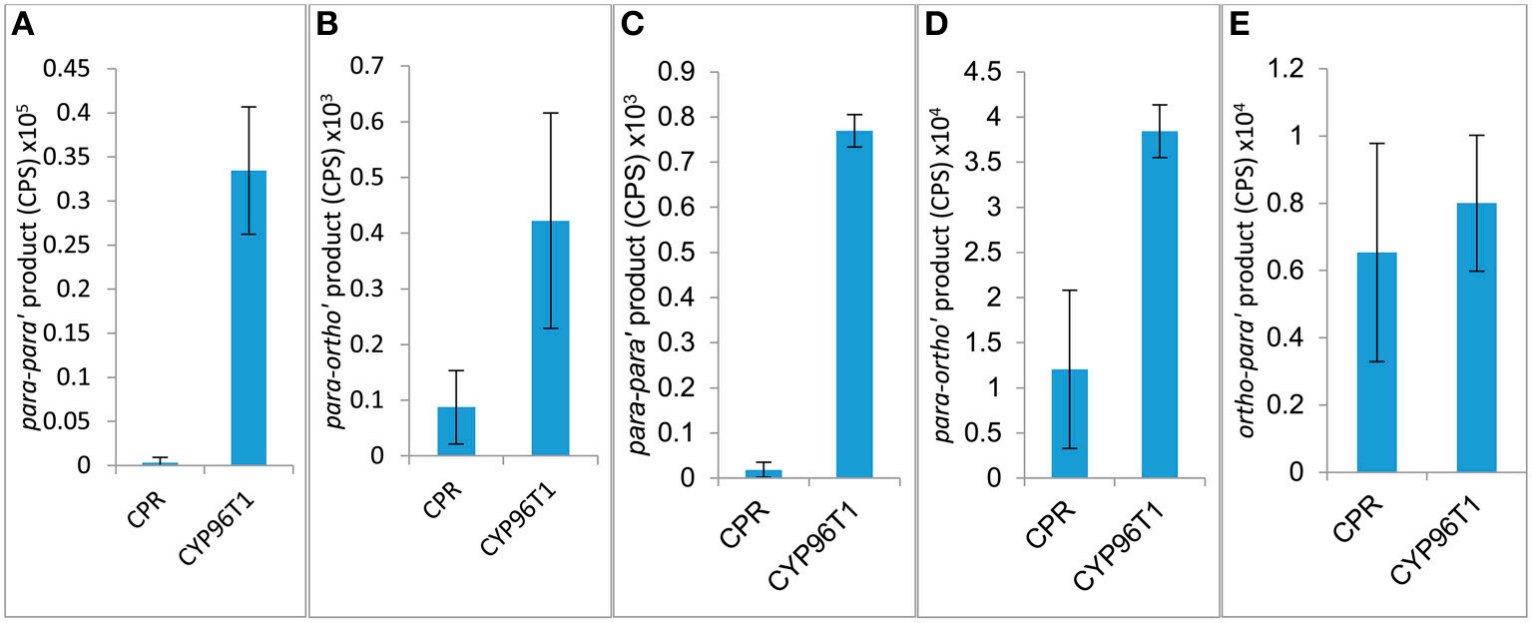
**Relative product formed in assays with 4′-***O***-methylnorbelladine (A,B) or 4′-***O***-methyl-***N***-methylnorbelladine (C,D,E) as substrate**. Assays are performed in triplicate only expressing *CPR* or with *CPR* in combination with *CYP96T1*. **(A)** *para-para'* [(10b*R*,4a*S*)- and (10b*S*,4a*R*)-noroxomaritidine] product. **(B)** *para-ortho'* (*N*-demethylnarwedine) product. **(C)** Potentially *para-para' C-C* phenol coupling (unknown 1) product. **(D)** *para-ortho'* (Narwedine) product. **(E)** Potentially *ortho-para' C-C* phenol coupling (unknown 2) product.

The concentration of CYP96T1 in Sf9 cell culture was determined to be 2.5 nM by CO-difference spectra. The temperature and pH optima for 4′-*O*-methylnorbelladine substrate were determined to be 30°C (half height ±5−10°C) and 6.5 (half height ±1), respectively. Testing of the CYP96T1 enzyme demonstrated that several structurally related alkaloids were *C-C* phenol coupled as detected by LC-MS/MS. These reactions were accompanied by a background reaction catalyzed by the Sf9 cells. 4′-*O*-methylnorbelladine was *C-C* phenol coupled into *N*-demethylnarwedine, (10b*S*,4a*R*)- and (10b*R*,4a*S*)-noroxomaritidine in CYP96T1 assays. (10bS,4a*R*)- and (10b*R*,4a*S*)-noroxomaritidine were identified by their identical liquid chromatographic retention times (Figure 5A) and mass spectrometric fragmentation pattern with (10b*S*,4a*R*)- and (10b*R*,4a*S*)-noroxomaritidine mixed standard (Figures 5C,D). To determine the chirality of the noroxomaritidine product, 4′-*O*-methylnorbelladine assays with CYP96T1 were analyzed with a chiral-CBH column by LC-MS/MS. Chromatographic separation of (10b*S*,4a*R*)- and (10b*R*,4a*S*)-noroxomaritidine standards was achieved preceding MS/MS analysis. Equal amounts of each enantiomer were observed (Figure 6A). A mass spectrometric comparison of standards (Figures 6B,C) and enzymatically formed (10b*S*,4a*R*)- and (10b*R*,4a*S*)-noroxomaritidine (Figures 6D,E) yielded identical MS/MS fragmentation patterns. The enzyme is, therefore, producing both (10b*S*,4a*R*)- and (10b*R*,4a*S*)-noroxomaritidine. A minor *N*-demethylnarwedine product was also detected in assays analyzed by HPLC on the Luna C8 column. The relative quantity of (10b*S*,4a*R*)- and (10b*R*,4a*S*)-noroxomaritidine and *N*-demethylnarwedine formed in assays with CYP96T1 are quantified in Figures 7A,B. HPLC was used to measure the relative contribution of these compounds to total product. (10b*S*,4a*R*)- and (10b*R*,4a*S*)-noroxomaritidine account for ~99% of the total product in CYP96T1 assays. (10b*S*,4a*R*)- and/or (10b*R*,4a*S*)-noroxomaritidine and *N*-demethylnarwedine are also produced in assays containing only Sf9 cells and 4′-*O*-methylnorbelladine, but not in an enzyme-free control, indicating Sf9 cells have the ability to catalyze the *C-C* phenol couple with 4′-*O*-methylnorbelladine (Figure 5A). In addition, the *N*-methylated form of 4′-*O*-methylnorbelladine, 4′-*O*-methyl-*N-*methylnorbelladine, was shown to produce several *C-C* phenol-coupled products when assayed with Sf9 cells alone, as indicated by the detection of products with a mass reduction of 2 *m/z*, including narwedine and two unknown products (Figures 5B, 7D). Unknown 1 is enzymatically produced from 4′-*O*-methyl-*N-*methylnorbelladine by CYP96T1, as indicated by the increase of product in assays containing CYP96T1 as compared to the CPR-only control (Figure 5B). Unknown 2 production can be explained by the endogenous activity of Sf9 cells only expressing CPR on 4′-*O*-methylnorbelladine (Figure 7E). These observations were confirmed by an MRM-based relative quantification of selected transitions of these three products (Figures 7C–E). The LC-MS/MS fragmentation pattern of unknown 1 is a mixture of masses found in the *para'-para* products (10b*S*,4a*R*)- and (10b*R*,4a*S*)-noroxomaritidine (165.1 *m/z*, 184.2 *m/z*, 195.0 *m/z*, 212.2 *m/z*, 229.0 *m/z*) and masses +14 *m/z* (120.1 *m/z*, 149.1 *m/z*, 243.2 *m/z*, 258.1 *m/z*, 271.0 *m/z*), representing the addition of a methyl moiety (Figure 5E). For this reason, it appears the enzyme is capable of catalyzing formation of the *para-para' C-C* phenol-couple regardless of *N*-methylation state (Figures 7A,C). To examine the ability of CYP96T1 to *C-C* phenol couple substrates with an altered carbon linker between the phenol groups, (*S*)-coclaurine, and (*R*)-coclaurine were also tested. Assays on ether (*S*)-coclaurine or (*R*)-coclaurine yield products with a mass −2 m/z, which is consistent with a *C-C* phenol coupling. Product formation is not observed when norbelladine or *N*-methylnorbelladine is used as substrate. These results indicate the 4′-*O*-methylation state of norbelladine may be important for substrate-enzyme binding. The substrates 3′-*O*-methylnorbelladine and 3′,4′-*O*-dimethylnorbelladine were tested to determine the relevance of 3′-*O*-methylation; products were not detected (Table 5).

**Table 5:** **Substrate specificity tests for CYP96T1**.

**Substrate**	***K_*m*_* (μM)**	***k_*cat*_* (1/min)**	*k_*cat*_/K_*m*_* (1/μM^*^min)	***K_*i*_***	**Modifications monitored**
4′-*O*-Methylnorbelladine	1.13 ± 0.54	15.0 ± 2.03	13	64.3 ± 26.40	*C-C* phenol coupling
4′-*O*-Methyl-*N*-methylnorbelladine	3.28 ± 2.27	2.44 ± 0.54	0.742	174 ± 140	*C-C* phenol coupling
(*S*)-Coclaurine	637 ± 156	1.34 ± 0.15	2.11 × 10^−3^	NA	Intramolecular phenol coupling and Intermolecular coupling
(*R*)-Coclaurine	659 ± 104	2.07 ± 0.14	3.15 × 10^−3^	NA	Intramolecular phenol coupling and Intermolecular coupling
3′*-O*-Methylnorbelladine	NA	ND	NA	NA	*C-C* phenol coupling
3′,4′-*O*-Dimethylnorbelladine	NA	ND	NA	NA	*C-C* phenol coupling
Norbelladine	NA	ND	NA	NA	*C-C* phenol coupling
*N-*Methylnorbelladine	NA	ND	NA	NA	*C-C* phenol coupling
Haemanthamine	NA	ND	NA	NA	Methoxy bridge formation and hydroxylation
(10b*S*,4a*R*)- and (10b*R*,4a*S*)-Noroxomaritidine	NA	ND	NA	NA	Methoxy bridge formation and hydroxylation
Isovanillin and tyramine	NA	ND	NA	NA	*C-C* phenol coupling, amine-aldehyde condensation, amine-aldehyde condensation, and *C-C* phenol coupling

*ND, not detected; NA, not applicable*.

The *K*_*m*_ of (*S*)-coclaurine, 636.7 μM, and (*R*)-coclaurine, 658.8 μM, are several orders of magnitude higher than the *K*_*m*_ values for 4′-*O*-methylnorbelladine, 1.13, and 4′-*O*-methyl-*N*-methylnorbelladine, 3.28 (Table 5). Substrate inhibition was observed in 4′-*O*-methylnorbelladine and 4′-*O*-methyl-*N*-methylnorbelladine with *K*_*i*_ values of 64.34 ± 26.36 μM and 173.7 ± 140.0 μM respectively. No substrate inhibition was observed in (*R*)-coclaurine or (*S*)-coclaurine with concentrations up to 1000 μM. The *k*_*cat*_ of 4′-*O*-methylnorbelladine was higher than observed for 4′-*O*-methyl-*N*-methylnorbelladine, (*R*)-coclaurine, or (*S*)-coclaurine. The *k*_*cat*_/*K*_*m*_ value 4′-*O*-methylnorbelladine is at least one order of magnitude larger than the 4′-*O*-methyl-*N*-methylnorbelladine, (*R*)-coclaurine, or (*S*)-coclaurine values consistent with the role of 4′-*O*-methylnorbelladine as the native substrate.

Some cytochrome P450 enzymes generate reactive H_2_O_2_ when lacking a substrate (Mishin et al., 2014). To confirm H_2_O_2_ generation by CYP96T1 was not the mechanism of action for *C-C* phenol coupling, the effects of H_2_O_2_ addition and catalase mediated H_2_O_2_ removal on *C-C* phenol coupling of 4′-*O*-methylnorbelladine were examined. This was done by adding all combinations of H_2_O_2_ and/or the H_2_O_2_-consuming enzyme catalase to CPR negative controls or CYP96T1 functional assays. The addition of 0.1% H_2_O_2_ to assays was found to enhance approximately 10-fold the production of the 4′-*O*-methylnorbelladine *C-C* phenol coupling in the CPR control, but no enhancement was observed in a functioning CYP96T1 assay. Although product formation resulted from H_2_O_2_ in CPR controls, the CYP96T1 assays produced ~100-fold more product than these control assays. The addition of 0.1 mg/ml catalase to the assays reversed the effects of supplemented H_2_O_2_ on CPR control assays, but did not reduce the level of product observed in the CYP96T1 assays, indicating that H_2_O_2_ is not involved in enzymatic phenol couple formation.

### Sodium borohydride assays and analysis by LC-MS/MS

**Figure 8:**
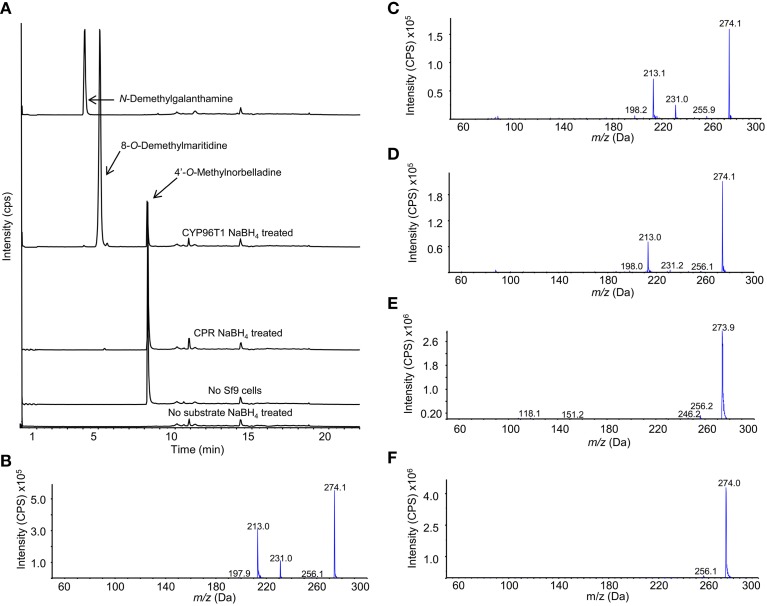
**LC-MS/MS Enhanced Product Ion (EPI) scan of sodium borohydride (NaBH_**4**_) treated CYP96T1 assays with 4′-***O***-methylnorbelladine substrate. (A)** Chromatograph with the following sample runs top to bottom: *N*-demethylgalanthamine standard, CYP96T1 assay, CPR assay, assay with no Sf9 cells and CYP96T1 assay without 4'-*O*-methylnorbelladine. **(B)** EPI fragmentation pattern of the *N*-demethylgalanthamine standard peak eluting at 4 min. **(C)** EPI fragmentation pattern of the *N*-demethylgalanthamine product in the CYP96T1 assay. **(D)** EPI fragmentation pattern of *epi-N*-demethylgalanthamine from the CYP96T1 assay. **(E)** EPI fragmentation pattern of (10b*S*,4a*R*)- and (10b*R*,4a*S*)-noroxomaritidine standard reduced to stereoisomeric 8-*O-*demethylmaritidine. **(F)** EPI fragmentation pattern of reduced (10b*S*,4a*R*)- and (10b*R*,4a*S*)-noroxomaritidine product from CYP96T1 assays.

Enzymatically formed *N-*demethylnarwedine from enzyme assays with CYP96T1 was converted to *N-*demethylgalanthamine by sodium borohydride reduction and detected by LC-MS/MS (Figure 8A). Sodium borohydride selectively reduced the ketone group on (10b*S*,4a*R*)- and (10b*R*,4a*S*)-noroxomaritidine and *N*-demethylnarwedine to yield a stereoisomeric mixture of the corresponding alcohols 8-*O*-demethylmaritidine and *N*-demethylgalanthamine. Confirmation of *N*-demethylgalanthamine in these assays is demonstrated by the identical retention time (Figure 8A) and fragmentation pattern (Figures 8B,C) with *N*-demethylgalanthamine standard. Another peak is also present with a different retention time (Figure 8A) and very similar fragmentation pattern (Figure 8D) and is likely the diastereomer *epi*-*N*-demethylgalanthamine formed by non-stereospecific ketone reduction. Stereoisomeric 8-*O*-demethylmaritidine is present in sodium borohydride reduced CYP96T1 4′-*O*-methylnorbelladine assays as the largest product peak (Figure 8A). This is validated by a comparison of the LC-MS/MS fragmentation pattern of (10b*S*,4a*R*)- and (10b*R*,4a*S*)-noroxomaritidine reduced by sodium borohydride to the corresponding peak in the CYP96T1 assay (Figures 8E,F).

To examine the potential relevance of the minor enzymatic product *N*-demethylnarwedine to galanthamine production in *N*. sp. *aff. pseudonarcissus*, flowering plants were collected and the scales of three bulbs were examined for haemanthamine and galanthamine content. As a result, haemanthamine was found to be 2.23 ± 0.38 mg/g fresh weight and galanthamine levels 0.246 ± 0.021 mg/g fresh weight. This translates into an 8.62-fold molar haemanthamine to galanthamine ratio. This prevalent ratio of haemanthamine relative to galanthamine *in planta* could indicate a higher significance of contribution of the CYP96T1 *N*-demethylnarwedine product to galanthamine biosynthesis than is at first evident from the ratio of enzymatically formed phenol-coupled products *in vitro*.

## Discussion

CYP96T1 converts 4′-*O*-methylnorbelladine to the products (10b*S*,4a*R*)- and (10b*R*,4a*S*)-noroxomaritidine indicating that this enzyme is involved in the biosynthesis of (10b*R*,4a*S*)-noroxomaritidine-derived alkaloids such as haemanthamine. Because (10b*S*,4a*R*)-noroxomaritidine derivatives have not been previously reported from *Narcissus* spp., the enantiomeric mixture of (10b*S*,4a*R*)- and (10b*R*,4a*S*)-noroxomaritidine made by CYP96T1 is interesting. One possibility is that the enzyme accepts 4′-*O*-methylnorbelladine in the two conformations required to make the two enantiomers. The enzyme subsequently would catalyze the phenol-phenol coupling and may still be bound to the intermediate upon nitrogen ring closure. This would, however, lead to an altered position of the 4′-*O*-methyl group in the active site and would likely lead to a preference for one conformation over the other. Since this preference is not observed, a second possibility that the CYP96T1 enzyme is only making the achiral intermediate that later spontaneously forms the different enantiomeric forms of noroxomaritidine is more likely. If the second possibility is the case, the absence of (10b*S*,4a*R*)-noroxomaritidine derivatives in *Narcissus* spp. may result from another enzyme perhaps associated with CYP96T1 directing the chirality of the ring closure, or in either case (10b*S*,4a*R*)-noroxomaritidine could be subject to degradation.

The production of *N*-demethylnarwedine by CYP96T1 is of interest to galanthamine biosynthesis. The low amount produced relative to (10b*S*,4a*R*)- and (10b*R*,4a*S*)-noroxomaritidine indicates that under the assay conditions used *N*-demethylnarwedine is not the enzyme's primary product. Kinetic analysis shows a clear preference for 4′-*O*-methylnorbelladine over all other tested substrates (Table 5).

**Figure 9:**
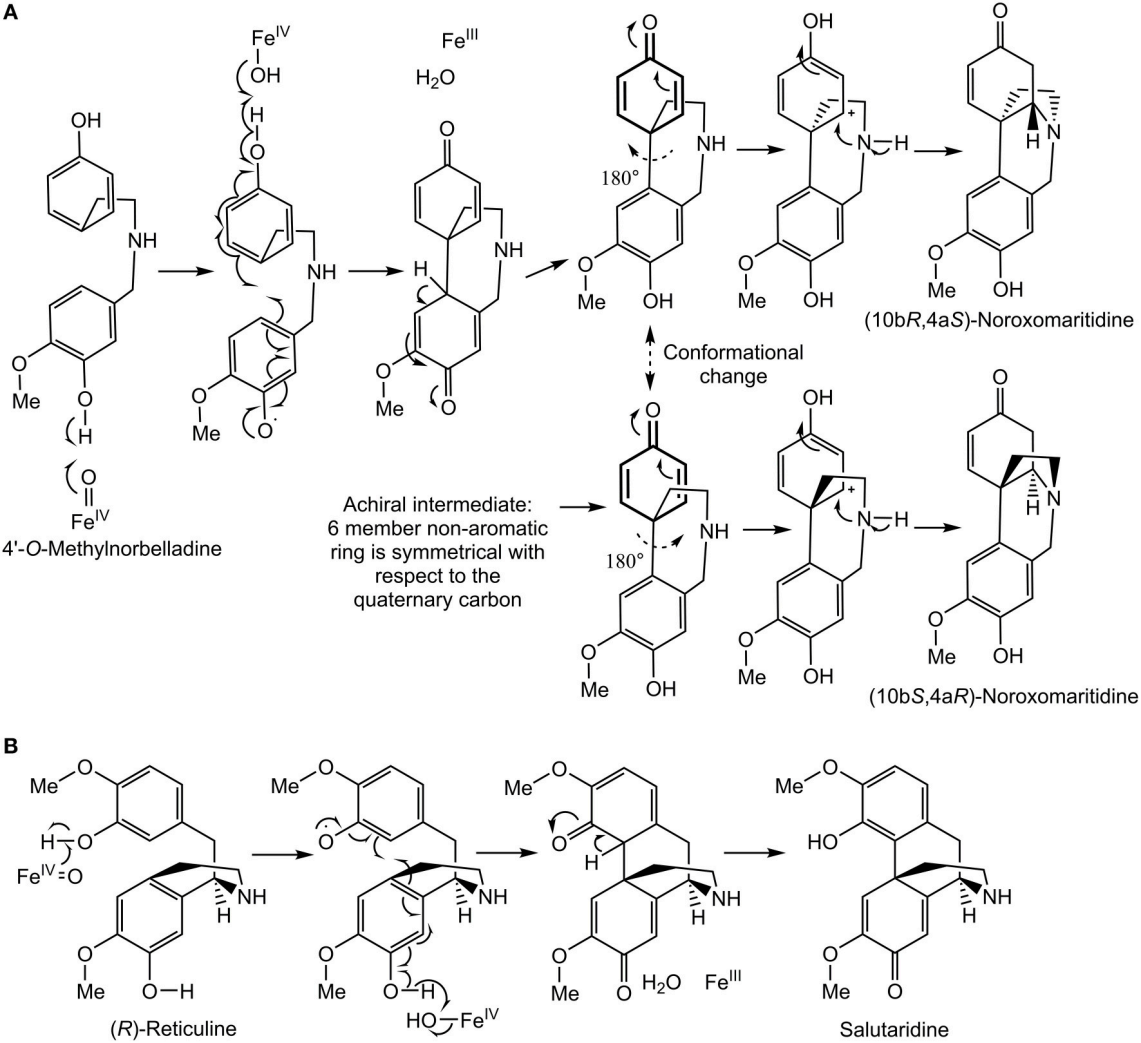
**Proposed ***C-C*** phenol coupling mechanisms. (A)** 4′-*O*-methylnorbelladine *para-para' C-C* phenol coupling mechanism followed by spontaneous nitrogen ring closure to form noroxomaritidine. **(B)** (*R*)-reticuline *para-ortho' C-C* phenol coupling mechanism to form salutaridine panel adapted from Grobe et al. (2009).

A diradical mechanism has been proposed for formation of the *C-C* phenol coupled product of (*R*)-reticuline and 4′-*O*-methylnorbelladine (Eichhorn et al., 1998; Grobe et al., 2009) (Figures 9A,B). A radical is formed on a hydroxyl group *ortho* or *para* to the position for formation of a carbon bond. To determine if the 3′ (*para'*) hydroxyl group is important to *C-C* phenol coupling, 3′-*O*-methylnorbelladine and 3′,4′-*O*-dimethylnorbelladine were tested for enzymatic activity; product formation was not observed. The lack of activity with a methoxy group at the *para*' position indicates that a free hydroxyl moiety is important at this position to enable extraction of a hydroxyl radical by the enzyme (Figure 9A). These results support the proposed mechanism for *C-C* phenol coupling of 4′-*O*-methylnorbelladine.

The oxygen binding and activation motif (A/G)GX(D/E)TT is substantially different between CYP96T1 (and its variants) when compared to cytochrome P450 enzymes that catalyze hydroxylation reactions. The substitutions G322Q, D324N, and T326Q replace key hydrophobic, acidic, and alcoholic groups with neutral hydrophilic amide groups. This is consistent with the proposal of Mizutani and Sato that cytochrome P450 enzymes not performing hydroxylation reactions can have a significantly altered oxygen binding and activation site (Mizutani and Sato, 2011). The highly conserved (387-389) EXXR, PERF (430-433) PXRX, and heme binding (464-473) FXXGXRXCXG motifs are present (Syed and Mashele, 2014). These motifs are thought to have more universal functions than substrate hydroxylation including maintenance of proper structural integrity and heme placement in cytochrome P450 enzymes (Hasemann et al., 1995; Hatae et al., 1996).

Presented herein is the first documented *C-C* phenol coupling cytochrome P450 enzyme in monocots. It is in the CYP96 family of cytochrome P450 enzymes, which falls into the CYP86 clan. The CYP96A15 from *A. thaliana* has been previously documented to be a midchain alkane hydroxylase involved in wax synthesis (Greer et al., 2007). Previously documented members of the CYP86 clan have shown activity toward fatty alcohols, fatty acids, alkanes, and derivatives thereof (Nelson and Werck-Reichhart, 2011). This makes this phenolic alkaloid a novel substrate class for this clan of cytochrome P450 enzymes. All other documented *C-C* phenol coupling plant cytochrome P450 enzymes are in the CYP71 clan (Nelson and Werck-Reichhart, 2011). This indicates the *C-C* phenol coupling activity of CYP96T1 was acquired independently from other known *C-C* phenol coupling cytochrome P450 enzymes. This independent origin of *C-C* phenol coupling could help direct the search for new *C-C* phenol coupling cytochrome P450 enzymes. The independent evolution of CYP96T1 shows that future searches for novel *C-C* phenol coupling enzymes should look broadly across the cytochrome P450 families because lineages of cytochrome P450 enzymes responsible for these reaction activities have likely not all been identified. Other phenol-phenol coupling reactions potentially performed by cytochrome P450s include the intramolecular coupling of 4′-*O*-methylnorbelladine to oxonorpluvine in lycorine biosynthesis, (*S*)-autumnaline to isoandrocymbine in colchicine biosynthesis, and the intermolecular *C-C* phenol coupling of dioncophylline A biosynthesis (Bringmann et al., 2000; Herbert, 2003).

When searching for additional phenol coupling enzymes responsible for the biosynthesis of the galanthamine and lycorine skeletons, the untested cytochrome P450 homologs co-expressing with *N4OMT* are prime candidates. These *N4OMT* co-expressing transcripts could also encode the hydroxylases or oxide bridge forming enzymes found in haemanthamine and lycorine biosynthesis.

## Author contributions

MK contributed to the study planning, performed most experiments, and wrote most of the manuscript. MA performed the isolation of RNA for sequencing and contributed to the writing of the manuscript. GM, JC performed the sequencing and subsequent transcriptome assemblies for *Galanthus* spp. and provided feedback on the manuscript. TK acquired funding for the study, conceived the study, and contributed to the writing of the manuscript. All authors approved the final version of the manuscript.

## Funding

This work was supported by the National Institutes of Health award number 1RC2GM092561 (NIGMS). This material is based upon work supported by the National Science Foundation under Grant No. DBI-0521250 for acquisition of the QTRAP LC-MS/MS.

### Conflict of interest statement

The authors declare that the research was conducted in the absence of any commercial or financial relationships that could be construed as a potential conflict of interest. A patent application has been filed for the sequences of CYP96T1-3.

## Footnotes

^1^“FASTX-Toolkit website.” Available online at: http://hannonlab.cshl.edu/fastx_toolkit (Accessed Jul 2, 2014).
