# Supplementary material for: CYP96T1 of Narcissus sp. aff. pseudonarcissus Catalyzes Formation of the Para-Para' C-C Phenol Couple in the Amaryllidaceae Alkaloids
Source: Front Plant Sci. 2016 Feb 25;7:225. doi: 10.3389/fpls.2016.00225 (PMC4766306; doi:10.3389/fpls.2016.00225)
Supplement: Supplementary file 1 [file Table1.DOCX]

Supplementary Material

# CYP96T1 of *Narcissus* sp*. aff. pseudonarcissus* Catalyzes Formation of the *Para-Para’* *C-C* Phenol Couple in the Amaryllidaceae Alkaloids

**Matthew B. Kilgore, Megan M. Augustin , Gregory D. May, John A. Crow, Toni M. Kutchan^*^**

^*^**Correspondence:** Toni M. Kutchan: [tkutchan@danforthcenter.org](mailto:tkutchan@danforthcenter.org)

# Supplementary cytochrome P450 list used in BLASP search

>CYP55B1 Chlamydomonas reinhardtii (green algae)(possible CYP55 fungal origin)

MAPQHDFPFSRPKGVEPPAEYKELRSKCPVAPGRLFDGSKIWLISRHKELKEVLQDGRFS

KVRTLPGFPELSPGGKAAAQSGNAATFVDMDPPEHTKYRGMVWPYLTPEAVEQLRPSIQA

KADKLVDAMIARGGPLDLNEAFSMPLPFRVIYDFIGIPEADFAYLSANVAVRSSGSSNAK

DAAAAADDLVKYMDNLVAEKERNPTGKDLISELVTKQLRPGHMTREQLVQTAFLMLVAGN

ATVATQINLGVISLLQHPDQLAAMKADPARLVPAATEEICRFHTGSSYALRRLAVADVQV

DGQLVKKGEGIIALNQSANRDESVFPDPDRFDIHRQSNPQQVGFGYGTHVCVAEWLARAE

IQVAIGTLFRRLPNLRLAVPESQIQYSDPARDVGLAALPVTW*

>CYP72A1v1 Catharanthus roseus

MEMDMYTIRKAIAATIFALVVAWAWRVLDWAWFTPKRIEKRLRQQGFRGNPYRFLVGDVK

ESGKMHQEALSKPMEFNNDIVPRLMPHINHTINTYGGNSFTWMGRIPRIHVMEPELIKEV

LTHSSKYQKNFDVHNPLVKFLLTGVGSFEGAKWSKHRRIISPAFTLEKLKSMLPAFAICY

HDMLTKWEKIAEKQGSHEIDIFPTFDVLTSDVISKVAFGSTYEEGGKIFRLLKELMDLTI

DCMRDVYIPGWSYLPTKRNKRMKEINKEITDMLRFIINKRMKALKAGEPGEDDLLGVLLE

SNIQEIQKQGNKKDGGMSINDVIEECKLFYFAGQETTGVLLTWTTILLSKHPEWQERARE

EVLQAFGKNKPEFERLNHLKYVSMILYEVLRLYPPVIDLTKIVHEDTKLGPYTIPAGTQV

MLPTVMLHREKSIWGEDATEFNPMRFADGVANATKNNVTYLPFSWGPRVCLGQNFALLQA

KLGLAMILQRFTFDVAPSYVHAPFTILTVQPQFGSHVIYKKLES

>CYP72A5 Zea mays (maize) from Schuler revised

MLREVSPWALASVVASVSLLWLVVWTLEWAWWTPWRLERALRVQGLKGTRYRLFTGDLRE

TARANREARKKPLPLGSHDIAPRVQPMHHSTIKEYGKLSFTWFGPTPRVMIPDPELVKEV

LSNKFGHFGKPRSNRIGRLLANGLVNHDGEKWAKHRRILNPAFHHEKIKGMMPVFSTCCI

EMITRWDNSMPSEGSSEIDVWPEFQNLTGDVISRTAFGSNYQEGRRIFELQGELAERLIQ

SVQTIFIPGYWFLPTKNNRRMRAIDVEIRKILREIIGKREKDTKNRETNNDDLLGLLLES

NTRQSNGNASLGLTTEDVIEECKLFYFAGMETTSVLLTWTLIVLSMHPEWQERAREEVLS

HFGRTTPDYDSLSRLKTITMILHEVLRLYPPATFLTRRTYKEMELGGIKYPAGVDLLLPV

IFIHHDPDIWGKDASEFNPERFANGISSATRHQAAFFPFGGGPRICIGQSFALLEAKMTL

CTILQRFSFELSPSYTHAPYTVITLHPQHGAQIRLKKLSP

>CYP72A7 Arabidopsis thaliana

MSFSVVAALPVLVAVVVLWTWRIVKWVWIKPKMLESSLKRQGLTGTPYTPLVGDIKRNVD

MMMEARSKPINVTDDITPRLLPLALKMLNSHGKTFFIWIGPLPTIVITNPEQIKEVFNKV

NDFEKASTFPLIRLLAGGLASYKGDKWASHRRIINPAFHLEKIKNMIPAFYHCCSEVVCQ

WEKLFTDKESPLEVDVWPWLVNMTADVISHTAFGSSYKEGQRIFQLQGELAELIAQAFKK

SYIPGSRFYPTKSNRRMKAIDREVDVILRGIVSKREKAREAGEPANDDLLGILLESNSEE

SQGNGMSVEDVMKECKLFYFAGQETTSVLLVWTMVLLSHHQDWQARAREEVMQVLGENNK

PDMESLNNLKQMTMIFNEVLRLYPPVAQLKRVVNKEMKLGELTLPAGIQIYLPTILVQRD

TELWGDDAADFKPERFRDGLSKATKNQVSFFPFGWGPRICIGQNFAMLEAKMAMALILQK

FSFELSPSYVHAPQTVMTTRPQFGAHLILHKL

>CYP72A61 Glycine max (soybeans, Fabales)

MRGLGLNLTPITTFAIITVIATVLIWWFWNALNWVWLRPKRIERRLKEQGIQGNSYRPLI

GDIRDMVKMIKEAKSKPMDPHSNDIAPRVLPYVVHTIAKYGKSSFMWLGPTPRVFILDPD

KFKEMATKVYDFQKPDTSPLFKLLASGFANYDGDKWAKHRKIVSPAFNVEKMKLLVPIFC

QSCDDLISKWESLLSSSNGSCELDVWPFVQNVSSDVLARAGFGSSYQEGKKIFELQREMI

QLTMTLFKFAFIPGYRFLPTHTNRRMKAIDKEIRESLMVIINRRLKAIKAGEPTNNDLLG

ILLESNYKESEKSSGGGMSLREVVEEVKLFYLAGQEANAELLVWTLLLLSRHPDWQEKAR

EEVFQVFGNEKPDYERIGQLKIVSMILQESLRLYPPVVMFARYLRKDTKLGELTIPAGVE

LVVPVSMLHQDKEFWGDDAGEFNPERFSEGVSKATKGKLSYLPFGWGPRLCIGQNFGLLE

AKVAVSMILQRFSLHFSPSYAHAPSFIITLQPERGAHLILRKL

>CYP72C1 Arabidopsis thaliana

MLEIITVRKVFLIGFLILILNWVWRAVNWVWLRPKRLEKYLKKQGFSGNSYRILMGDMRE

SNQMDQVAHSLPLPLDADFLPRMMPFLHHTVLKHGKKCFTWYGPYPNVIVMDPETLREIM

SKHELFPKPKIGSHNHVFLSGLLNHEGPKWSKHRSILNPAFRIDNLKSILPAFNSSCKEM

LEEWERLASAKGTMELDSWTHCHDLTRNMLARASFGDSYKDGIKIFEIQQEQIDLGLLAI

RAVYIPGSKFLPTKFNRRLRETERDMRAMFKAMIETKEEEIKRGRGTDKNQRLLFSMLAS

NTKTIKEQGPDSGLSLDDLIDDCKAFYLAGQNVTSSLFVWTLVALSQHQDWQNKARDEIS

QAFGNNEPDFEGLSHLKVVTMILHEVLRLYSPAYFTCRITKQEVKLERFSLPEGVVVTIP

MLLVHHDSDLWGDDVKEFKPERFANGVAGATKGRLSFLPFSSGPRTCIGQNFSMLQAKLF

LAMVLQRFSVELSPSYTHAPFPAATTFPQHGAHLIIRKL*

>CYP72D1 Populus trichocarpus (cottonwood)

MEDFIFRGFLSSSLLLSLYVVFRVAHTFWLKPKSQEKRLRKQGIRGTSYKILNGDMKEFA

RSSKEARSRPLALNQEIAPRVFPFFYKMVQIYGKVSLCWMGTRPSLLLADPELVRLVLTD

TSGHIIKPPRNALVGLLQLGVSTLEGDKWAKRRRLMTPAFHVERLRGMIPAFSACCCDLV

QRWKKLAGPQGSCELDVASEFNILASDVIARAAFGSSYEEGKRIFDLQKDQVTLVLEAFY

SIYFPGLRFIPSKKNKKRYSIDKEIKAALRNIIHKKEQAMQNGDLGDADLLGLLLKGRDD

ADNDMKIEDVIEECKLFFFAGQETTANLLTWTLVVLSMHPDWQEKAREEVLQICGKRTPD

TDSIKQLRIVSMILNEVLRLYPPVNLLYRHTLKETSIRGMSIPAGVDLLLPFLFLHYDPE

YWGDNAEEFKPERFSEGVSKASKDEIAFYPFGWGPRFCLGQNFALTEAKMALTMILQNFW

FELSPSYTHAPGNVITLQPQHGAPIILHQL*

>CYP73A5 Arabidopsis thaliana

MDLLLLEKSLIAVFVAVILATVISKLRGKKLKLPPGPIPIPIFGNWLQVGDDLNHRNLVD

YAKKFGDLFLLRMGQRNLVVVSSPDLTKEVLLTQGVEFGSRTRNVVFDIFTGKGQDMVFT

VYGEHWRKMRRIMTVPFFTNKVVQQNREGWEFEAASVVEDVKKNPDSATKGIVLRKRLQL

MMYNNMFRIMFDRRFESEDDPLFLRLKALNGERSRLAQSFEYNYGDFIPILRPFLRGYLK

ICQDVKDRRIALFKKYFVDERKQIASSKPTGSEGLKCAIDHILEAEQKGEINEDNVLYIV

ENINVAAIETTLWSIEWGIAELVNHPEIQSKLRNELDTVLGPGVQVTEPDLHKLPYLQAV

VKETLRLRMAIPLLVPHMNLHDAKLAGYDIPAESKILVNAWWLANNPNSWKKPEEFRPER

FFEEESHVEANGNDFRYVPFGVGRRSCPGIILALPILGITIGRMVQNFELLPPPGQSKVD

TSEKGGQFSLHILNHSIIVMKPRNC*

>CYP74A1 Arabidopsis thaliana

MASISTPFPISLHPKTVRSKPLKFRVLTRPIKASGSETPDLTVATRTGSKDLPIRNIPGN

YGLPIVGPIKDRWDYFYDQGAEEFFKSRIRKYNSTVYRVNMPPGAFIAENPQVVALLDGK

SFPVLFDVDKVEKKDLFTGTYMPSTELTGGYRILSYLDPSEPKHEKLKNLLFFLLKSSRN

RIFPEFQATYSELFDSLEKEAFPLRESGFRRFQRRNRLLFLGSSFLRDESRRYKLKADAP

GLITKWVLFNLHPLLSIGLPRVIEEPLIHTFSLPPALVKSDYQRLYEFLRIRGEILVEAD

KLGISREEATHNLLFATCFNTWGGMKILFPNMVKRIGRAGHQVHNRLAEEIRSVIKSNGG

ELTMGAIEKMELTKSVVYECLRFEPPVTAQYGRAKKDLVIESHDAAFKVKAGEMLYGYQP

LATRDPKIFDRADEFVPERFVGEEGEKLLRHVLWSNGPETETPTVGNKQCAGKDFVVLVA

RLFVIEIFRRYDSFDIEVGTSPLGSSVNFSSLRKASF*

>CYP74B2 Arabidopsis thaliana

MAATSPRPPPSTSLTSQQPPSPPSQLPLRTMPGSYGWPLVGPLSDRLDFQGPDKFFRTRA

EKYKSTVFRTNIPPTFPFFGNVNPNIVAVLDVKSFSHLFDMDLVDKRDVLIGDFRPSLGF

YGGVRVGVYLDTTEPKHAQIKGFAMETLKRSSKVWLQELRSNLNIFWGTIESEISKNGAA

SYIFPLQRCIFSFLCASLAGVDASVSPDIAENGWKTINTWLALQVIPTAKLGVVPQPLEE

ILLHTWPYPSLLIAGNYKKLYNFIDENAGDCLRLGQEEFGLTRDEAIQNLLFVLGFNAYG

GFSVFLPSLIGRITGDNSGLQERIRTEVRRVCGSGSDLNFKTVNEMELVKSVVYETMRFS

PPVPLQFARARKDFQISSHDAVFEVKKGELLCGYQPLVMRDANVFDEPEEFKPDRYVGET

GSELLNYLYWSNGPQTGTPSASNKQCAAKDIVTLTASLLVADLFLRYDTITGDSGSIKAV

VKAK*

>CYP75A14 Pinus taeda

MVSLNLNEFMLWFLSWLALYIGFRYVLRSNLKLKKRRLPPGPSGWPVVGSLPLLGAMPHV

TLYNMYKKYGPVVYLKLGTSDMVVASTPAAAKAFLKTLDINFSNRPGNAGATYIAYDSQD

MVWAAYGGRWKMERKVCNMHMLGGKALEDWQPVRDAEMGFMLRNILSHSQRGETVNVPDL

LNICAANMIGQIILSKRVFETEGDEANEFKDMVVELMTCAGYFNIGDFIPSVAWMDLQGI

QRGMKKLHKKWDALIQRMIDEHQSTAKQRASKPDFLDVVMSQRDNCDGQGGRLSDVHIKA

LLLNLFTAGTDTSSSVIEWTLAELMNNPKLLKRVHEEMDAVIGRERRLKESDLANLPYFV

AVCKEGFRKHPSTPLSLPRVSTEACEVDGYYIPKNTRLMVNIWGIGRDPEVWEKPEEFNP

DRFVGSKIDPRGNDFELIPFGAGRRICAGTRMGITMVEYNLGSLIHAFDWDVPPNQEGLN

MDEAFGLALQKAVPLVAKVSPRLPLHLY*

>CYP75A17 Glycine max flavonoid 3', 5'-hydroxylase

MDSLLLLKEIATSILIFLITRLSIQTFLKSYRQKLPPGPKGWPVVGALPLMGSMPHVTLA

KMAKKYGPIMYLKMGTNNMVVASTPAAARAFLKTLDQNFSNRPSNAGATHLAYDARDMVF

AHYGSRWKLLRKLSNLHMLGGKALDDWAQIRDEEMGHMLGAMYDCNKRDEAVVVAEMLIF

MANMIGQVILSRRVFETKGSESNEFKDMVVELMTVAGYFNIGDFIPFLAKLDLQGIERGM

KKLHKKFDALLTSMIEEHVASSHKRKGKPDFLDMVMAHHSENSDGEELSLTNIKALLLNL

FTAGTDTSSSIIEWSLAEMLKKPSIMKKAHEEMDQVIGRDRRLKESDIPKLPYFQAICKE

TYRKHPSTPLNLPRISSEPCQVNGYYIPENTRLNVNIWAIGRDPDVWNNPLEFMPERFLS

GKNAKIDPRGNDFELIPFGAGRRICAGTRMGIVLVHYILGTLVHSFDWKLPNGVRELDME

ESFGLALQKKVPLAALVTPRLNPSAYIS

>CYP75B1 Arabidopsis thaliana

MATLFLTILLATVLFLILRIFSHRRNRSHNNRLPPGPNPWPIIGNLPHMGTKPHRTLSAM

VTTYGPILHLRLGFVDVVVAASKSVAEQFLKIHDANFASRPPNSGAKHMAYNYQDLVFAP

YGHRWRLLRKISSVHLFSAKALEDFKHVRQEEVGTLTRELVRVGTKPVNLGQLVNMCVVN

ALGREMIGRRLFGADADHKADEFRSMVTEMMALAGVFNIGDFVPSLDWLDLQGVAGKMKR

LHKRFDAFLSSILKEHEMNGQDQKHTDMLSTLISLKGTDLDGDGGSLTDTEIKALLLNMF

TAGTDTSASTVDWAIAELIRHPDIMVKAQEELDIVVGRDRPVNESDIAQLPYLQAVIKEN

FRLHPPTPLSLPHIASESCEINGYHIPKGSTLLTNIWAIARDPDQWSDPLAFKPERFLPG

GEKSGVDVKGSDFELIPFGAGRRICAGLSLGLRTIQFLTATLVQGFDWELAGGVTPEKLN

MEESYGLTLQRAVPLVVHPKPRLAPNVYGLGSG

>CYP75B3Oryza sativa (rice)

MDVVPLPLLLGSLAVSAAVWYLVYFLRGGSGGDAARKRRPLPPGPRGWPVLGNLPQLGDK

PHHTMCALARQYGPLFRLRFGCAEVVVAASAPVAAQFLRGHDANFSNRPPNSGAEHVAYN

YQDLVFAPYGARWRALRKLCALHLFSAKALDDLRAVREGEVALMVRNLARQQAASVALGQ

EANVCATNTLARATIGHRVFAVDGGEGAREFKEMVVELMQLAGVFNVGDFVPALRWLDPQ

GVVAKMKRLHRRYDNMMNGFINERKAGAQPDGVAAGEHGNDLLSVLLARMQEEQKLDGDG

EKITETDIKALLLNLFTAGTDTTSSTVEWALAELIRHPDVLKEAQHELDTVVGRGRLVSE

SDLPRLPYLTAVIKETFRLHPSTPLSLPREAAEECEVDGYRIPKGATLLVNVWAIARDPT

QWPDPLQYQPSRFLPGRMHADVDVKGADFGLIPFGAGRRICAGLSWGLRMVTLMTATLVH

GFDWTLANGATPDKLNMEEAYGLTLQRAVPLMVQPVPRLLPSAYGV*

>CYP76A3 Petunia

MVLSESNFLLCLISISIASVFFFLLKKTSRSYKLPPGPSGLPIVGNMFDLGDLPHIKMEG

MRNQYGPVMWLKIGAINTLVIQSAQAATAFFKNHDANFLERVVVEVNRVCNYLQGSLALA

PYGNYWRMLRRICSMELFVHSRINNSESIRRKSVDKMIQWIETHGKKEQGQGIEITRFVF

LASFNMLGNLIMSKELAADPDSTTASEFFDAMMGQVEWSGTPNISDVFPLLRWLDIQGLR

RKMKRDMGKGKEILSTFIKERIKEQENGRAKGTDFLDVLLAFEGKGKDEPAKLSEHEINI

FILEMFLAGTETSSSTTEWALTELLRNPETMARVKAEIAEVVGPNKKFEESDIDKVPYMQ

AVVKETFRLHPPLPFLLPRKATQDTKFMGYDVPKGTQIFINAWAIGRDPECWHDPLDFIP

ERFIGSKIDFKGLNYELIPFGAGRRMCVGVPLGHRMVHFVLGTLLHEFNWELPHNMSSKS

IDMTERLGTTVRKLEPLKVIPNKCKLS

>CYP76B4 Medicago sativa (alfalfa)

MDMLQSSTLSYLVIIFTFSMLLLIKFLIPTNKTNQKNHSKLPPGPSPLPIIGNLLKLGNK

PHHSLANLSNIHGPIMTLKLGQVTTIVISSADIAKEVLQTHDTLLSNRTVPDALSVLNHD

QYSLSFMRVSPRWRDLRKICNNQLFSNKTLDSSQALRRRKLQDLLDDIKKCSEIDEAVDI

GRVAFMTTINLLSNTFFSADFVHSAEEAGEYKEIVVSILKEVGAPNLSDFFPMLTVFDLQ

GIRRRSVVSVKKVLSIFRRFVGERLKLREGTGSIENDDVLDALLNISLDDGKIEMDKDEI

EHLLLNIFVAGTDTTTYTLEWAMAELMHNPEIMSKVQKELEQVVGKGIPIQETDIAKLPY

MQAVIKETFRLHPPVPLLLPRKAETDVEIGDYIIPKDAQVLVNAWVIGRDPNKWDNANVF

VPERFLDSEIDVKGHHFELIPFGSGRRICPGLPLAIRMLPMMLGSLVNCFDWKLEDGLNI

DDLNKEDEYGITLEKSQPVRIVPIKLTIQ

>CYP76C1 Arabidopsis thaliana

MDIISGQALLLLFCFILSCFLIFTTTRSGRISRGATALPPGPPRLPIIGNIHLVGKHPHR

SFAELSKTYGPVMSLKLGSLNTVVIASPEAAREVLRTHDQILSARSPTNAVRSINHQDAS

LVWLPSSSARWRLLRRLSVTQLLSPQRIEATKALRMNKVKELVSFISESSDREESVDISR

VAFITTLNIISNILFSVDLGSYNAKASINGVQDTVISVMDAAGTPDAANYFPFLRFLDLQ

GNVKTFKVCTERLVRVFRGFIDAKIAEKSSQNNPKDVSKNDFVDNLLDYKGDESELSISD

IEHLLLDMFTAGTDTSSSTLEWAMTELLKNPKTMAKAQAEIDCVIGQNGIVEESDISKLP

YLQAVVKETFRLHTPVPLLIPRKAESDAEILGFMVLKDTQVLVNVWAIGRDPSVWDNPSQ

FEPERFLGKDMDVRGRDYELTPFGAGRRICPGMPLAMKTVSLMLASLLYSFDWKLPKGVL

SEDLDMDETFGLTLHKTNPLHAVPVKKRANIN

>CYP76D1 Cicer arietinum

WRSLRRACATKIFSPQQLDSTQFHRKRKVQDLLNYVQKCCEKGEALDFGEVVLATVMNSI

SETFISMDLFHYCDPSNDDDNKKSREFKEMVFGIMEEVGRPNVVDFFPFLKLFDPQGVRT

RMRNHFEKLLAFFYEVMKERMRLRASGESKEYKDVLDSFLDLLNEENSQLCRHDVLHLFT

DLFVAGIDTTSTTMEWAMAELLHNPSKLARLRKELEQIHGKFGQIEESDASKLPYLRAVV

KEILRLHPSVPFLVPHKSKDDGELGGFMVPKNAQILVNVWSIGRNSSIWDNQIHLNLKDF

WRVKLISKGRDFELVPFGAGRRICPGLPLASRSIHYIMASLLHHFNFKLADDLKPDDMDM

SHKFGVTLHKAQPLRVVPIKA

>CYP76E1 Medicago sativa (alfalfa)

MDHQTLLLVITFVSATILIFFLRKSNQTQNSTKLPPGPYPLPIIGNILELGKNPHKALTK

LSKIYGPIMTLKLGSITTIVISSPQVAKQVLHDNSQIFSNRTVPHAITAVDHDKFSVGWV

PTLNLWKKLRKNCATKVFSTKMLDSTKILRQQKLQELLDYVNEKSHNGEVFDIGETVFIN

VLNSISNTLFSMDLAHSTPDEKSQEFKTIIWGIMEEAGKPNISDFFPILRPLDPQGLYAR

MTNHMKKLCEIFDGIIEERICLKDSKGDYEVCNDVLDSLLNINIGEATSELSRNEMVHLF

LDLFVAGIDTTSSMIEWIIAELLRNPDKLTKVRKELCQTIGKGETIEESHISKLPFLQAV

VKETFRLHPPIPLLLPHKCDELVNILDFNVPKNAQVLVNVWAMGRDPAIWDNPNTFVPER

FMECDINYKGNNFELIPFGAGKRICPGLPLAHRTMHLMVASLLHNFEWNLADGLIPEHLN

MDEQFGLTLKRVQPLRVEAISSA

>CYP76F1 Cicer arietinum

NSARVMTLKLGQVTTVVISSADMAKEVLLTHDLITSNRTVPDALSVLNHDQYSLSFMRVS

PRWRDLRKICNYQLFSNKTLDSSQALRRRKLQDLLNDIERCSKVGEAVDVGKAAFKTTVN

LLSNTFFSVDFVHSAKEAGEYKEIIVSILKEVGVPNVSDFFPMLKFLDLQGIRKRSIVSV

KKVLSIFKRFVGERVKMREGTGSIGNDDVLDALLNMSSDGGKIEMDKDEIEHLLLNIFVA

GTDTTTYTLEWAMAELIHNPEMMSKLKEELEKTVGKGIPVEETDIAKLPYMQAVIKETFR

LHPPVPLLLPRRAEIDVKIGDYVIPKDAQILINAWVVGRDPTKWENPNVFIPERFLDSEI

DIKGHHFELIPFGSGRRTCPGLPLAIRMLPLMLGSLVNCFDWKLEDGLNVEDFNKEDEFG

ITLEKSQPVRIVPTKLY

>CYP76G1 Arabidopsis thaliana

MINQLTKNELIGLFTSIAVLIYVTCLFYTKRCRTRLPPGPNPWPVIGNIFQLAGLPPHDS

LTKLSRRHGPIMTLRIGSMLTVVISSSEVAREIFKKHDAALAGRKIYEAMKGGKSSDGSL

ITAQYGAYWRMLRRLCTTQFFVTRRLDAMSDVRSRCVDQMLRFVEEGGQNGTKTIDVGRY

FFLMAFNLIGNLMFSRDLLDPDSKRGSEFFYHTGKVMEFAGKPNVADFFPLLRFLDPQGI

RRKTQFHVEKAFEIAGEFIRERTEVREREKSDEKTKDYLDVLLEFRGGDGVDEEPSSFSS

RDINVIVFEMFTAGTDTTTSTLEWALAELLHNPRTLTKLQTELRTYFKSSNQKLQEEDLP

NLPYLSAVIMETLRLHPPLPFLVPHKAMSTCHIFDQYTIPKETQVLVNVWAIGRDPKTWI

DPIMFKPERFISDPDARDFKGQDYEFLPFGSGRRMCPALPLASRVLPLAIGSMVRSFDWA

LENGLNAEEMDMGERIGITLKKAVPLEAIPIPYRGT*

>CYP76H6 Oryza sativa (rice)

MASALFLWLSWLVLSLLSIYLLDLLAHSRRRLPPGPRPLPLIGSLHLLGDQPHRSLAGLA

KTYGPLMSLRLGAVTTVVVSSPDVAREFLQKHDAVFATRSAPDAAGDHTRNSVPWLPPGP

RWRELRKIMATELFATHRLDALHELRQEKVSELVDHVARLARDGAAVDVGRVAFTTSLNL

LSRTIFSRDLTSLDDRGASKEFQQVVTDIMGAAGSPNLSDFFPALAAADLQGWRRRLAGL

FERLHRVFDAEIEHRRRVAGEEHGKVKDDFLRVLLRLAARDDDTAGLDDDTLRSVFTLLK

DLFAAGSDTSSSTVEWAMAELLRNPLPMAKACDELQRVIGSTRRIEESDIGRLPYLQAVI

KETFRLHPPVPFLLPRQATTTIQILGYTIPKGAKVFINVWAMGRDKDIWPEAEKFMPERF

LERATDFKGADFELIPFGAGRRICPGLPLAVRMVHVVLASLLINFKWRLPIKVERDGVNM

TEKFGVTLAKAIPLCAMATST

>CYP76K1 Oryza sativa (rice)

MELTTISPVFLISLLGVPLLYLLWSKASKSPSGAPAAPPPPPGPTPFPVIGNIPDLLRGG

ELHRALTGLAASYGPVMSLRLGMASTVVLSSPDVAHEALHKKDGAISSRWVPDNANVLGH

QDVSMAWLPSSSPLWKHMRTLASTLLFTSRRLGASRGIRERKARELVDYLGARSGRPVRV

GLAVFGSVLNFMSNVFFSEDVVELGSETGQEFQQLIADSVAETAKPNISDFFPFLSALDL

SRRRRAAAKNLKKFYDFFDDVIDRRLSSGEKPGDLLDSLLELHAKSQLERPLIRALMDLF

IAGSHTTTTTVEWAMAELLRNPSKMAKARAELGEAFGRGAVEEGELARLPYLNAVIKETL

RLHPPAPLLLPHRVSSDSEPAGGVTLGGYSVPSGARVLINAWAIGRDPAAWSPEPDAFSP

ERFLGREADYWGRTLEFIPFGSGRRACPGIPLAVAVVPMVVAAMVHSLEWRLPEGMAPGD

VDVGDRFGAVLELATPLWAVPVKV

>CYP76L1 Oryza sativa (rice)

MEASTILWLLYVSLASCLLYKVFVSTKNGHPKIAARRPPGPTPVLLLGNVFDLRGELHLA

LARLAEEHGPVMSLKLGTATAVVASSAAAARDALQRYDHVLAARAVCDAARALGTHERSI

VWLPGSSALWKRLRAVCTNHLFSARGLDATRAVREAKVRELVEHLRGHAAGAGEEEAAAV

DVGRVVFSAVINLVSNVLFSEDVADLSSDRAQELEMLVRDTVEEATKPNLSDLFPVLAAL

DLQGRRRRTAVHIRKFHDFFDEIISRRQNAGGEGERKEDFLDVLLQLHSADQLSLDTIKT

FLGDLFTAGTDTNSITVEWAMAELLRHPAAMSRARAELRDALGAKPHPDESDIGRLPYLS

AVVMETMRLHPPSPLLMPHEAVADGAAVGGYAVPRGTKVIVNVWSIMRDPASWPRPEEFE

PERFVAAGGSFRGGEMLEFMPFGAGRRACPGTPMATRVVTLVLASLLHAFEWRLPGGMRP

CDVDVRGRFGTSLNMVTPLKAVPVPVPARP

>CYP76M15 Zea mays (maize)

MATPELWWYWWLWVTTMLAVVVSTVVCYLTNQHRRWGGWGSSSGRRRPPGPRPLPLIGNL

LDLRRAPGSLHHTLARLARAHGAPVMRLDLGLVPAVVVSSRDAAREAFAAHDRRIAARPV

PDSKRALGFCDRSMLSLPSSAPLWRTLRGVMAAHVLSPRSLAASRAARERKVADLIGYLR

ARAGTVVDLKEAVYGGVANLVSTAMFSIDVVDVGAAESSSSSSAAAHGLQELLEELMQCM

AQPNVSDFFPFLSALDLQGCRRRVAVQLGQVLQVLDDITDRRLASSSSSSTSSKGGDRRG

DFLDILLDLQSTGKITRDNVTLTLFDIFAAGSDTMALTVVWAMAELLRNPGVMARLRAEV

RDALGGRDAVEEADAAGLPFLQAVVREAMRLHPAAPVLLPHKAVEDGVQIGGYAVPRGCT

VIFNSWAIMRDPAAWERPDEFLPERFLARDLDFRGKQLEFVPFGSGRRLCPGVPMAERVV

PLVLASLVHAFQWQLPAGMSADQVDVSDKFTTTSVLAFPPIKAVPLL*

>CYP76N1 Oryza sativa (rice)

MAASLAWLLVAIVLASLYLAMHHRVAAARRRRLPPGPTPLPLVGNLLSVSRSGPHRSLAR

LAERYCPLMRVRLGVVDYVVASSPAVAGDIHHHSHNAHLASRPLFDVWRGAEHHRNSVIV

LPLHGVWRAQRRLATEEVMSPRRLDALAPTRREKVRELMRCVARRAARGEPVEVGLEAFE

AFLGILSCTAFSADLVDPDLRDAVQEATKLAATPNASDFFPAMAAADLQGLRRRMGKLVA

RAYGIIDELLARRKGGREAGEPRKDDMLDVALDNEDEWKNNNPVIDRNNIKGLIADLFVA

GTDSGSTAIEWAIVELLQNPQSMQKVKDEFRRVLGTRTEIEESDISQLPYLQAVLKETLR

LHPSVPMTYYKAEATVEVQGYIIPKGTNIILNIWAIHRKPDVWADPDRFMPERFMETDTN

FFGKHPEFIPFGGGRRICLGLPLAYRMVHMVLASLLFHFDWKLPEGAEKDGVDMREKYGM

VLHKETPLKALAIETYNR

>CYP76P1 Oryza sativa (rice)

MAIFIGCICSLALLLLCSHVFQLLSDARRRLPPGPRPLPVIGNLLDVAGELPHRSLACVA

ERYGPLVTLRLGTMLAVVASSPATARDVLHRHGASITDRGTPDAWSTDGHDGNSIFAFPT

RHHRWRALRRLGAEQLFSPRRVEEQRPLRRDAVRGLLRHVAELAAASGGGGAAVVDVGRA

AFAAMASLLFGALFSAGIDAATSCRFRDAAREFALLTMTPNVSEFFPVVAMADLQGLRRR

TARHITWMYQLIDGHVERRMRGRETAGGCGAAHGEKEKDLLDVMLDMSEKEEQNDDSSLT

MNDLLMAGSETSSAVIEWAMAELLQNPQTMTKLQEELKKVIGSKTCIDEEDIDQLPYLQA

VIKETHRLHPAIPLLMYKAAVPVEIQGYKIPKETTVIVNTWAIHQNSEVWIEPDKFIPER

FLQKEISLSSGSTNMELIPFSAGRRFCLGYPVANRMLHVMLASLVHQFQWTLPEVVKKNG

GVDMAEKFGITLSMATPLHAIAKNIV*

>CYP76Q1 Oryza sativa (rice)

MAFFLVACLPWVCFILLSLYVFQLFADARRRLPPGPWPPKPLIGDLLALGKGDQQHRSLA

RLADRYGPVMSLRLGTVLTVVVSTPDAMREIFHKNKDNLAGRPTADAFNAMGHSANSLLG

LEHPGVRWRAIRRFSTAELLAPRRLAALQPLCRDKVRGLVRGVSELAARGEPVHVRRVAL

DMALSLILSAIYSVDLDPESTAVFRSVVEEAMLLIGTANLSDLFPAIAALDLQGVRRRVA

ELFTITYRQYDEQVARRRPERDAGEAGKNDLLNVVLDMEREWQQKGSVLSHDAMRVLFTD

LYGAGASTTSVLIEWAIADLLQNPESMRKIKEEITNVIGTNAQIQESDIARLPYLQAVVK

ETLRLRAVAPLVPRRAEATIEVQGFTIPKGTNVILNLWAINRDARAWNDPDKFMPERFIG

NDINYLGQNFQFVPFGVGRRICLGLPLAQKVMYLVLGTLVHQFEWTLPEELKDTGIDMTE

KCGMVLCLANPLKVMAKKM*

>CYP76S1 Centaurium erythraea (Common Centaury)

MDVIFPLLVAFITWAIASSLTFRRFGRLPPGPFPVPVIGNIHQLGKHPNQSLAKLSKIYG

PLMSLKLGTQTAIVASSSTVVREILQKHDQVFSSRTIPSALHAHDHHKFSMALLPASSRW

RHLRKITKEQMFSVQRLDESQGLRQDKLKELRDYLHSCCVTGQAVNIGEAAFTTTLNLMS

CTLFSVNFASFDSKFSDELKRDICAFVQVIAAPNLADFSPVLRHVDPQGLLKRTKTYMQK

VFDSFEDIITKRLQERGTSQQDSLRRHDLLEALLDEMEKNDSAFTINDMKHLILDLFIAG

ADSTSSTTEWGMAELLHNPEKMEKAKAELNEVIGQKNLVEESDISRLPYLQAVVKEVFRL

HPPGPLLVPHKADADVEIDGYVVPKNANVLVNVWALGRDSSSWADPEAFMPERFLDNEID

VKGQHFELIPFGAGRRMCPGLPLSYRMLH

>CYP76T1 Populus trichocarpus (cottonwood)

MEYLFYLLLISFCWACLHVLNASVLLRRKSGCTVLPPAPRQLPIIGNILALGDKPHRTLA

KLSQTYGPLMTLKLGRITTIVISSPNIAKEALQKHDQALSSRTVPDALHVQYYNYHKNSM

IWLPASTQWKFLRKLTATQMFTSQRLDASRALRGKKVQELLEYVHEKCNNGHAVDVGRSV

FTTVLNLISNTFFSLDVTNYNSDLSQEFSNLVVGFLEQIGKPNIADYFPILRLVDPQGIR

RKTNNYLKRLTQIFDSIINERTRLRSSSVASKASHDVLDALLILAKENNTELSSTDIQVL

LIDFFIAGTDTTSSTVEWAMTELLLNPDKMVKAKNELQQVEGPVQESDISKCPYLQAIVK

ETFRLHPPSPFLPRKAVSEVEMQGFTVPKNAQVLITIWAIGRDPAIWPEPNSFKPERFLE

CQADVKGRDFELIPFGAGRRICPGLPLGHKMVHLTLASLIHSFDWKIADDLTPEDIDMSE

TFGFTLHKSEPLRAIPMKT*

>CYP76U1 Oryza sativa (rice)

MAFFLPLAFSLFLAVISAYVLQLLADARRRLPPGPWPLPLIGNLHQLDHLPHRSLARLAA

RHGPLMSLRLGTVRAVVASSPEMAREVLQRH55795NADIAARSFGDSMRAGGHCENSVV

CLPPRRRWRALRRLSTVGLFSPRRLDAMRALLEEKVAELVRRVSGHAARGEAVDVGHAAH

VAALGVLSRTMFSVDLDPEAAREVSDIVDEASVLGTGPNVSDFFPAIAPADLQGVRRRMA

RLVKRMYAIIDEQIERRMHGRTAGEPRKNDLLDVMLEEGESKEDSNEINRDAIRGLDLFT

GGETTSHTMECAMAELLQCPNSMRRVHELKSVIGSKQQMDEHDITKLPYLPPYEAEATIE

IQGYTIPKGAKVLINLWAINRCANTWTEPDKFMPERFYDSDITFMGRDFQLIPFGAGKRI

CLGLPLAHRMVHLMLGSLLHRFTWTLPAEAGKNGVDMRERFGLTLSFVAPLYVIAQEIQ*

>CYP76V1 Oryza sativa (rice)

MAFFHLCISSLLLVFIISYIFQPLLDARRRFPPGPHRLPVISNLHNIGKNPHHAFARLAD

RYGPLMSIRLGGVRAVVAMSADAAREILQRNNADITGRGGMDSWHACGHHANSSIALWPR

WKWCAMRMLCTEELLGVTHAMREEVARELAHRVSDGSAGGMPVSVAREAFAAVAGVLWWS

MFSEDMDAATTRQLRDVIEEAVVVAGAPNLSDYFPVIAAADVMGVRRRMDNLVGWVYGII

DVQIDRRRRRRIVCEPRKNDLLDVAFDMEGEVESEGWVMNQDTMRGMAIYQDLLVAGSGS

TSSTIEWAMAELLQNPKSMIQLPEELKGLMGTKTHVAESDISQLPYLQAVIKETLRLHPT

VPIAFNKAEATVEIQGYKIPQGTTVYVNIWAICRRAKIWDDLDKFMPYRFLGRDINFLGT

NFEFIPFGAGRRICLGMPLAEGMLHLMLASLLHRFEWTIPDEVKGDDLDMAEEFGLVLSM

AKPLRAVAKET*

>CYP76X1 Trifolium pratense (Fabales)

MDYVGSGMLLLLTCIVACFIGSLYARSRKSNYRLPPGPSIFTIMSHVFELYYKPQQTLAK

FAKFYGPVMLIKLCTETTVIISSSDMAKEILHTNDSLFTDRSVPDNTTTHNHNNFSLVFL

PFSPLWQHLRKICHNNLFSNKTLDGSQELRRMKLKDLLNDMHKSSLKGETVDIGRAAFKA

CINFLSYTFVSQDFVESLDDEYKDIVSTLLSAVGTPNIADHFPILKILDPQGIKRHTTKY

VAKVFHALDIIIDQRMKLRKSEDYVSKNDMLDSLLDISKEDSQKMDKKQIKHLLLDLLVA

GTETSAYGLERAMTRLVHDPKAMSKARKELEETIGLGNPIEESDIDRLPYLNAVIKESLR

LHPPAPMLLPRKARVDVEISGYTIPKGAQVLINEWAIGRTDIWDDADSFSPERFLGSEID

VKGRHFKLTPFGSGRRICPGSPLAVRMLHLMLGSLINSFDWKLENNMEAKDMNLDKPLRA

IPVALNKVY

>CYP76Y1 Vitis vinifera

MELNSFLLLCMPLVLCLFFLQFLRPSSHATKLPPGPTGLPILGSLLQIGKLPHHSLARLA

KIHGPLITLRLGSITTVVASSPQTAKLILQTHGQNFLDRPVPEAIDSPQGTIAWTPVDHV

WRSRRRVCNNHLFTSQSLDSLQHLRYKKVEQLLQHIRKHCVSGTPVDIGLLASATNLNVL

SNAIFSVDLVDPGFESAQDFRDLVWGIMEGAGKFNISDYFPMFRRFDLLGVKRDTFSSYR

RFYEIVGDIIKSRIKCRASNPVTRNDDFLDVILDQCQEDGSLFDSENIQVLIVELFYAGS

DTSTITTEWAMTEFLRNPGVMQKVRQELSEVIGAGQMVRESDMDRLPYFQAVVKETLRLH

PAGPLLLPFKAKNDVELSGFTIPSNSHVLVNMWAIARDPSYWEDPLSFLPERFLGSKIDY

RGQDFEYIPFGAGRRICPGMPLAVRMVQLVLASIIHSFNWKLPEGTTPLTIDMQEHCGAT

LKKAIPLSAIPFIEEN*

>CYP76Z1 Picea sitchensis

MMNSMAIESVYDPLVFGVVLSFIFLLLLHWKKKNSRLPPGPPGWPIIGNVLQLGDKPHES

LFGLAQKYGPLMSLRLGCKLTMVVSSPSMAKEVLKDNDQTFSSRSINMAARTFAYQGTSL

VWSPYGPHWRFLRRICNAELFSPKRLDALQHLRREEVNRTIRSIFEVSMEGQSVNIGEIA

FLNSLSLVGMMVCSRNLFNPGSKEVAEFKEMVWEVLKLTGTPNLSDLFPFLERFDLQGLK

KGMKTLARRFDSLFDSIIEERLGEDGAGVHHEGKDFLEIMLGLRKAGTQFTLENIKAVLM

DMFIAGTDTTSVTVEWAMAELLGKPAVIRKAQAELDEIVGQAKRMEESDIAKLPYLQAIV

KEALRLHPAAPLIIPRRSDNSCEIGGYVVPENTQVFVNVWGIGRDPSVWKEPLEFNPERF

LECNTDYRGQDFELIPFGAGRRICIGLPLAHRMVHLVLGSLLHAFNWSIPGATKDDDFVI

DMSEVFGLTLQKKVPLIAVPTPRQPINLLY

>CYP76AA1 Cycas rumphii (cycad)

MAMPACSSWKFLLYSTVFEIILLSFLLIILRDKKKRGKLPPGPPGWPIVGNLFQLGKKPN

ESLLQLAKKYGPLMSLRLGMKTAIVVSSPAMAREVFKNHDHLFAGRTVIQAAKCASHDKS

SLVWSQYGPRWRMLRKICNTELFGVRRLNALQHLRRDQIFQTIRSIYEENYLKRNTVNVG

HTAFLTSLNVLGNMIFTQNIFGRDSQAAEELKQTISKVMEISGTPNLADYFPFLQIGDPQ

GITRAKTLYLKRVYALLDKFVEDRLSSTSTPQNGSSAEKDFLDVLIDCYRNADDGEGAGI

SRTDITPLIYDLIVAGSETTSTTIEWGLAEVIRNLQAMKRTQAELDDVVGRDRQVEESDI

GHLPYLSAVVKEVFRLHPPAPLLLPHRADSCCEIAGFFIPKDAQVIVNVWGMGRDPSTWN

DPMEFVPERFTESEVDFKGNNMELIPFGAGRRICPGLPLANRMLHFLLAALLHSFDWSLP

DGHNSQQMEMTGKFGLTLQKASPLMAVPSPRLPANLY*

>CYP76AB1 Vanda coerulea

MEELTQYLLWSIFFFIAIAMLLRRRSSRNLALPPGPRPLPVLGNLLELGQNPHRSLALLA

RIHGPVMYLKLGSITQSSSPLQPPQKKSLKQKITPPPPDKSQILSQAVGHHQVSVIWLSP

NQSWRYLRTLMKANLFNAKSLNATELLRRRKVRELIAYIKGKNGEAVHVARAAFCTVLNL

ISTTFLSIDMVDIFQSESAQELKDLMSGIMEEVGRPNVSDFFPFLAPIDLQGCRRRFAAY

IKKLSDFFDEVIENRLAGGGGRNKHDILNALLQLSREENSKLSRNTIISFLIDSFAAGSE

TSSATLEWAMVELLRSPEQMATAREEIATVIGLEREVEESDMSRLPFLQAVLKETLRLHP

PGPLLVPHKTEESTEINGYAVPKNSQFLVNVWAIGRDERLWENPDCFMPERFVAGGEIDF

RGHHFELLPFGSGRRICPGMPLGVRMVQLMLASLLQSFEWGLPDGMKPEDLDLTEKHGLS

TVLAAPLKAIATPTKHN

>CYP76AC2 Ceratopteris richardii (fern)

AAIHTTALTTEWGIAELLKHPHCITRLRQEMEEVLGDKKGQLIVEADIAKLTYLQCVIKE

ILRLHPVVSLLLPRMSSQECEVGGYTIPAKTLTFVNVWAIGRDEDVWENALEFRPERFES

NKDIDVKGHHYELLPFGSGRRICAGLPVALSMVSLTLANLVHCFDLELPHGQTPDSMNME

ERKGIAANKAVPTVLVPKPRFSMNFC

>CYP77A4 Arabidopsis thaliana

MFPLISFSPTSLDFTFFAIIISGFVFIITRWNSNSKKRLNLPPGPPGWPVVGNLFQFARS

GKPFFEYAEDLKKTYGPIFTLRMGTRTMIILSDATLVHEALIQRGALFASRPAENPTRTI

FSCNKFTVNAAKYGPVWRSLRRNMVQNMLSSTRLKEFGKLRQSAMDKLIERIKSEARDND

GLIWVLKNARFAAFCILLEMCFGIEMDEETIEKMDEILKTVLMTVDPRIDDYLPILAPFF

SKERKRALEVRREQVDYVVGVIERRRRAIQNPGSDKTASSFSYLDTLFDLKIEGRKTTPS

NEELVTLCSEFLNGGTDTTGTAIEWGIAQLIANPEIQSRLYDEIKSTVGDDRRVDEKDVD

KMVFLQAFVKELLRKHPPTYFSLTHAVMETTTLAGYDIPAGVNVEVYLPGISEDPRIWNN

PKKFDPDRFMLGKEDADITGISGVKMIPFGVGRRICPGLAMATIHVHLMLARMVQEFEWC

AHPPGSEIDFAGKLEFTVVMKNPLRAMVKPRI

>CYP77B1 Arabidopsis thaliana

MDLTDVIIFLFALYFINLWWRRYFSAGSSQCSLNIPPGPKGWPLVGNLLQVIFQRRHFVF

LMRDLRKKYGPIFTMQMGQRTMIIITDEKLIHEALVQRGPTFASRPPDSPIRLMFSVGKC

AINSAEYGSLWRTLRRNFVTELVTAPRVKQCSWIRSWAMQNHMKRIKTENVEKGFVEVMS

QCRLTICSILICLCFGAKISEEKIKNIENVLKDVMLITSPTLPDFLPVFTPLFRRQVREA

RELRKTQLECLVPLIRNRRKFVDAKENPNEEMVSPIGAAYVDSLFRLNLIERGGELGDEE

IVTLCSEIVSAGTDTSATTLEWALFHLVTDQNIQEKLYEEVVGVVGKNGVVEEDDVAKMP

YLEAIVKETLRRHPPGHFLLSHAAVKDTELGGYDIPAGAYVEIYTAWVTENPDIWSDPGK

FRPERFLTGGDGVDADWTGTRGVTMLPFGAGRRICPAWSLGILHINLMLARMIHSFKWIP

VPDSPPDPTETYAFTVVMKNSLKAQIRSRT*

>CYP77C1v1 Selaginella mollendorffii (lycopod moss)

MVAFLAVAVAILAASLLLLSRRRTHLPPGPRGFPLLGNLLQMRSVLGSPMNLQNLARQHG

AIMTLRVGSVPLVVISSSQLAHEALIEKGSIFSSRPSLSERQVRLSNYRRSINAAPYGHH

WRTVRRNMVSHVLSPHRVHAFEPARQRVISELVEKLRQTSQRADSPDGPSAVPVLATLRF

TVFSLLSYMCFGQWLDKDAVNGVERMLRHLITSAGRGGRMSDFVPLLKIVQRSPRDLKLE

ELVGERRELLLPLIQRAKLLAAEDKLDQNSYLSSLFSLQRQEDHQLKLTDEDLIVLCSEF

LNAGADTTANTLEWSLANVIKHPAVQKKLLEEIHSSVGDKPVTEKDIDKLVYLKAVVKET

LRKHPPGYTTLPHAVTEPCKLGGYDIPVHATLLFNIYAINNDPELWTNPDEYKPERFLEG

PGASADFTASSGALNLIPFGAGRRICPGLGLATLHVHLVLARLVQEFEWNTVPGETAVDL

TPIQEFTVVMKQPLRATLKARRL*

>CYP77D1 Selaginella mollendorffii (lycopod moss)

MDLAALVATIALLALASWSLFFLQHLNLRRRMPPGPIGWPVLGSMREIPRLLSDPQKFQQ

LVARYGPIVTLWNGSVATILISSPDIAREALVEKGSVLASRPDVPSMRLLTSGFKTINSS

PYGVHWRATRKNLVSGILSPRVMSGFAPVQEQAAEDLVRKLASEAKQSGGTVESLSVSVR

CVLFQILSFVCFGRKLEEAKLEELNALMKEATTMLHPVLGDLVPFLKVFTSHTKQKSFLV

RQNQLLKGLLSRECPAASESYVQTLLSLQGKNLEDVDLDLAVLVRELFIAGADTTTNCVE

WSMANLIKYPGIQERVFRELAENVGQKSGVKVADLPKLPYLHAVVKEALRKHPPVYLSAP

RTPVHATKLAGYDIPKESTVVVHLQSLSNDAGVWKNPDKFLPERFFEQTELSKRMSMIPF

GAGRRDCPGKHLGMLHVHLIVANLVQAFEWRAEGKEVDLSPRTVFTVQMKNPLRASICQR

RS*

>CYP78A5 Arabidopsis thaliana

MSPEAYVLFFNSFNLVTFEAFASVSLIIATVAFLLSPGGLAWAWTGSSKSRVSIPGPSGS

LSVFSGSNPHRVLAALAKRFKASPLMAFSVGFSRFVISSEPETAKEILSSSAFADRPVKE

SAYELLFHRAMGFAPYGEYWRNLRRISSTHLFSPRRIASFEGVRVGIGMKMVKKIKSLVT

SDACGEVEVKKIVHFGSLNNVMTTVFGESYDFDEVNGKGCFLERLVSEGYELLGIFNWSD

HFWFLRWFDFQGVRKRCRALVSEVNTFVGGIIEKHKMKKGNNLNGEENDFVDVLLGLQKD

EKLSDSDMIAVLWEMIFRGTDTVAILVEWVLARMVLHQDIQDKLYREIASATSNNIRSLS

DSDIPKLPYLQAIVKETLRLHPPGPLLSWARLAIHDVHVGPNLVPAGTIAMVNMWSITHN

AKIWTDPEAFMPERFISEDVSIMGSDLRLAPFGSGRRVCPGKAMGLATVHLWIGQLIQNF

EWVKGSCDVELAEVLKLSMEMKNPLKCKAVPRNVGFA*

>CYP78D1 Oryza sativa (rice)

MRNEVLSTIFLLLIFFTTTINPSSSQLPWLFSLLYLSLAMAVVALPPLLAKRHGHARRVN

GGGAAIPGPRGWPLLGSLPAVSGPLMHRRLAALAYAHGGGARRLMSLTLGATPVVVSSHP

DTAREILAGAAFRDRPARAAARELMFLRAVGFAPAAGDDGGAYWRRLRRAAGAGMLSPRR

AAALAALRARVARRTSEAVSRGMAVPPGRVAMRALLHAASLDNMVGSVLGLEHHDHHGGV

ISDMGDMVREGYELVGKFNLGDYYSTTQYQCLWGLLDFHGVGPRCQRLAARVREQFGRVM

EERRKVSDLHKRDDLLSYMLSMPQEERIEDSDVIAVLWEMIFRGTDVVAILLEWAMARMV

LHPDIQSKVQEELDRAVGHRPMTDSDIPSLRFLHCVIKETLRMHPPGPLLSWARLAVHDT

YVGKHLVPAGTTAMVNMWAISHDETIWGDPWVFRPERFMEEDINVLGSDLRLAPFGSGRR

VCPGRMMGLSTSYLWFGRMLQEYKWSPAQPVKLTECLRLSMEMKKPLVCHAVPRSKTG*

>CYP78D2 Populus trichocarpus (cottonwood)

MKSIPANLSSILFCLAVITHQTPWPVALLLFSLSSFFAFSLNYWLVPGGFAWRNHHDNQN

PSRFRGPIGWPIVGTLPQMGSLAHRKLASMAASLGATKLMAFSLGSTRVIISSHPDTARE

ILCGCSFADRPIKESARLLMFERAIGFAPSGDYWRHLRRIAANYMFSPRKISALEPLRQR

LANEMVAEVREEMKERRVVVLRDILQKGSLSNVLESVFGSDVSIEREELGFMVKEGFDLI

AEFNLDDYFPLRFLDFHGVKRRCCQLAGKVNSVVGQIVKERKGAGDSRSGSDFLSALLSL

PEEDQLNESDMVALLW15448966EMIFRGTDTVALLLEWIMARMVVHPEIQAKAQEELD

TCIGGHREVQDSDIPNLPYLRAIVKEVLRLHPPGPLLSWARLAIHDVHVDKTFIPAGTTV

MVNMWAITHDPSIWRDPWSFNPDRFIEEDVLIMGSDLRLAPFGAGRRVCPGKALGLATVH

LWLARLLHEYRWLPAKPVDLSECLRLSLEMKRPLECHVVQRRSKVTQ*

>CYP78E1 Physcomitrella patens (moss)

MAERPARLWPLTDFPIFISKGDIVCKDSCIGRFQKYQNVGRAVAKKFREFLSALTKSKAC

KPVNSVIKALAAPLILIAIAQEFSRDAVKQFLLDGFLTQPLRWLFQYISPFIQQVGTVDT

ATWTDVHASSILVFFIAAISLIISIVGWCGPGGPAWSFSRIFSPSNKLPTPNGPRGCPVI

GSWTLMQGSEMHRELARQAWAGGPSTRNLMALSVGTTLIVLTSDANVAKEILRSAVFGER

PLKQAALDLGFERAIGFALQGPYWRHLRKVAVTHMFSHRQIVTHSELLQRETLRMISAMV

HSIRTDCVKDYRVGLCARPFLQRAAVNNIMTIVFGRHFDFGNSCDEAEALEAMIREGFEL

LGGFNWADHLPLVRHIPFLSFSRRCRNLTMKVRAFVQSILDERRRCHHQSHSATSSVLNT

SFVDALLSLEGDQKLQDEDIISILWEMVFRGTDTIAVLTEWALAEVILNQGIQARIHEEL

DAVVGSNRLVQQKDIENLPYLQAVLKETLRSHPPGPLLSWARLANEDTQIAGCHIPRGTT

TMVNMWAITHDSSVWPNPEVFDPSRFLKSEGGSDLDVLGTDLRLAPFGSGRRVCPGRALG

IATAQLWLASLLHHFSWSQDLSHPIDLTDNLTLSCEMASPLHGCPTVRFPL*

>CYP79A2 Arabidopsis thaliana

MLDSTPMLAFIIGLLLLALTMKRKEKKKTMLISPTRNLSLPPGPKSWPLIGNLPEILGRN

KPVFRWIHSLMKELNTDIACIRLANTHVIPVTSPRIAREILKKQDSVFATRPLTMGTEYC

SRGYLTVAVEPQGEQWKKMRRVVASHVTSKKSFQMMLQKRTEEADNLVRYINNRSVKNRG

NAFVVIDLRLAVRQYSGNVARKMMFGIRHFGKGSEDGSGPGLEEIEHVESLFTVLTHLYA

FALSDYVPWLRFLDLEGHEKVVSNAMRNVSKYNDPFVDERLMQWRNGKMKEPQDFLDMFI

IAKDTDGKPTLSDEEIKAQVT*ELMLATVDNPSNAAEWGMAEMINEPSIMQKAVEEIDRV

VGKDRLVIESDLPNLNYVKACVKEAFRLHPVAPFNLPHMSTTDTVVDGYFIPKGSHVLIS

RMGIGRNPSVWDKPHKFDPERHLSTNTCVDLNESDLNIISFSAGRRGCMGVDIGSAMTYM

LLARLIQGFTWLPVPGKNKIDISESKNDLFMAKPLYAVATPRLAPHVYPT*

>CYP79B2 Arabidopsis thaliana

MNTFTSNSSDLTTTATETSSFSTLYLLSTLQAFVAITLVMLLKKLMTDPNKKKPYLPPGP

TGWPIIGMIPTMLKSRPVFRWLHSIMKQLNTEIACVKLGNTHVITVTCPKIAREILKQQD

ALFASRPLTYAQKILSNGYKTCVITPFGDQFKKMRKVVMTELVCPARHRWLHQKRSEEND

HLTAWVYNMVKNSGSVDFRFMTRHYCGNAIKKLMFGTRTFSKNTAPDGGPTVEDVEHMEA

MFEALGFTFAFCISDYLPMLTGLDLNGHEKIMRESSAIMDKYHDPIIDERIKMWREGKRT

QIEDFLDIFISIKDEQGNPLLTADEIKPTIKELVMAAPDNPSNAVEWAMAEMVNKPEILR

KAMEEIDRVVGKERLVQESDIPKLNYVKAILREAFRLHPVAAFNLPHVALSDTTVAGYHI

PKGSQVLLSRYGLGRNPKVWADPLCFKPERHLNECSEVTLTENDLRFISFSTGKRGCAAP

ALGTALTTMMLARLLQGFTWKLPENETRVELMESSHDMFLAKPLVMVGDLRLPEHLYPTV

K

>CYP79C1 Arabidopsis thaliana

MDYYLNNVIFSVVVTFSITLNIVFLIKSVVARFLGRRKKLPPCPRGFPIIGNLVGMLKNR

PTSKWIVRVMNDMKTDIACFRFGRVHVIVITSDVIAREVVREKDAVFADRPDSYSAEYIS

GGYNGVVFDEYGERQMKMKKVMSSELMSTKALNLLLKVRNLESDNLLAYVHNLYNKDESK

TKHGAVVNVRDIVCTHTHNVKMRLLFGRRHFKETTMDGSLGLMEKEHFDAIFAALDCFFS

FYVADYYPFLRGWNLQGEEAELREAVDVIARYNKMIIDEKIELWRGQNKDYNRAETKNDV

PMIKDWLDILFTLKDENGKPLLTPQEITHLSDLDVVGIDNAVNVIEWTLAEMLNQREILE

KAVEEIDMVVGKERLVQESDVPNLNYVKACCRETLRLHPTNPFLVPHMARHDTTLAGYFI

PKGSHILVSRPGVGRNPKTWDEPLIYRPERHITGNEVVLTEPDLRLVSFGTGRRGCVGAK

LGTSMIVTLLGRLLQGFDWTIPPGTTDRVELVESKENLFMANPLMACVKPRLDPNMYPKL

WTGPA*

>CYP79D1 Manihot esculenta (cassava)

MAMNVSTTIGLLNATSFASSSSINTVKILFVTLFISIVSTIVKLQKSAANKEGSKKLPLP

PGPTPWPLIGNIPEMIRYRPTFRWIHQLMKDMNTDICLIRFGRTNFVPISCPVLAREILK

KNDAIFSNRPKTLSAKSMSGGYLTTIVVPYNDQWKKMRKILTSEIISPARHKWLHDKRAE

EADNLVFYIHNQFKANKNVNLRTATRHYGGNVIRKMVFSKRYFGKGMPDGGPGPEEIEHI

DAVFTALKYLYGFCISDFLPFLLGLDLDGQEKFVLDANKTIRDYQNPLIDERIQQWKSGE

RKEMEDLLDVFITLKDSDGNPLLTPDEIKNQIAEIMIATVDNPSNAIEWAMGEMLNQPEI

LKKATEELDRVVGKDRLVQESDIPNLDYVKACAREAFRLHPVAHFNVPHVAMEDTVIGDY

FIPKGSWAVLSRYGLGRNPKTWSDPLKYDPERHMNEGEVVLTEHELRFVTFSTGRRGCVA

SLLGSCMTTMLLARMLQCFTWTPPANVSKIDLAETLDELTPATPISAFAKPRLAPHLYPT

SP*

>CYP79E1 Triglochin maritima

MELITILPSVLPNIHSTATVLFLLLLTTALSFLFLFKQHLTKLTKSKSKSTTLPPGPRPW

PIVGSLVSMYMNRPSFRWILAQMEGRRIGCIRLGGVHVVPVNCPEIAREFLKVHDADFAS

RPVTVVTRYSSRGFRSIAVVPLGEQWKKMRRVVASEIINAKRLQWQLGLRTEEADNIMRY

ITYQCNTSGDTNGAIIDVRFALRHYCANVIRRMLFGKRYFGSGGEGGGPGKEEIEHVDAT

FDVLGLIYAFNAADYVSWLKFLDLHGQEKKVKKAIDVVNKYHDSVIESRRERKVEGREDK

DPEDLLDVLLSLKDSNGKPLLDVEEIKAQIADLTYATVDNPSNAVEWALAEMLNNPDILQ

KATDEVDQVVGRHRLVQESDFPNLPYIRACAREALRLHPVAAFNLPHVSLRDTHVAGFFI

PKGSHVLLSRVGLGRNPKVWDNPLRFDPDRHLHGGPTAKVELAEPELRFVSFTTGRRGCM

GGPLGTAMTYMLLARFVQGFTWGLRPAVEKVELEEEKCSMFLGKPLRALAKPRQELLQSF

*

>CYP79F1 Arabidopsis thaliana

MMSFTTSLPYPFHILLVFILSMASITLLGRILSRPTKTKDRSCQLPPGPPGWPILGNLPE

LFMTRPRSKYFRLAMKELKTDIACFNFAGIRAITINSDEIAREAFRERDADLADRPQLFI

METIGDNYKSMGISPYGEQFMKMKRVITTEIMSVKTLKMLEAARTIEADNLIAYVHSMYQ

RSETVDVRELSRVYGYAVTMRMLFGRRHVTKENVFSDDGRLGNAEKHHLEVIFNTLNCLP

SFSPADYVERWLRGWNVDGQEKRVTENCNIVRSYNNPIIDERVQLWREEGGKAAVEDWLD

TFITLKDQNGKYLVTPDEIKAQCVEFCIAAIDNPANNMEWTLGEMLKNPEILRKALKELD

EVVGRDRLVQESDIPNLNYLKACCRETFRIHPSAHYVPSHLARQDTTLGGYFIPKGSHIH

VCRPGLGRNPKIWKDPLVYKPERHLQGDGITKEVTLVETEMRFVSFSTGRRGCIGVKVGT

IMMVMLLARFLQGFNWKLHQDFGPLSLEEDDASLLMAKPLHLSVEPRLAPNLYPKFRP

>CYP80A1 Berberis stolonifera

MDYIVGFVSISLVALLYFLLFKPKHTNLPPSPPAWPIVGHLPDLISKNSPPFLDYMSNIA

QKYGPLIHLKFGLHSSIFASTKEAAMEVLQTNDKVLSGRQPLPCFRIKPHIDYSILWSDS

NSYWKKGRKILHTEIFSQKMLQAQEKNRERVAGNLVNFIMTKVGDVVELRSWLFGCALNV

LGHVVFSKDVFEYSDQSDEVGMDKLIHGMLMTGGDFDVASYFPVLARFDLHGLKRKMDEQ

FKLLIKIWEGEVLARRANRNPEPKDMLDVLIANDFNEHQINAMFMETFGPGSDTNSNIIE

WALAQLIKNPDKLAKLREELDRVVGRSSTVKESHFSELPYLQACVKETMRLYPPISIMIP

HRCMETCQVMGYTIPKGMDVHVNAHAIGRDPKDWKDPLKFQPERFLDSDIEYNGKQFQFI

PFGSGRRICPGRPLAVRIIPLVLASLVHAFGWELPDGVPNEKLDMEELFTLSLCMAKPLR

VIPKVRI

>CYP80B2 Coptis japonica

MEVLSIAIVSFSFLLFLFFILRDSRPKNLPPGPRPSPIVGNLLQLGDKPHAEFAKLAQKY

GELFSLKLGSQTVVVASSPAAAAEILKTHDKILSGRYVFQSFRVKEHVENSIVWSECNDN

WKLLRKVCRTELFTPKMIESQSEIREAKAREMVKFLRGKEGEVVKIVEVVFGTLVNIFGN

LIFSKDVFDLEDPTGGSVELKEHLWKLLDMGNSTNPADYFPIMGKLDLFGQRRAVAEVLQ

QIYDVWGVMLKERRGTKGSESKNDFVDVLLNAGLDDQKINALLMELFGAGTETSASTIEW

AITELTKKPLVVSKIRLELVNVVGDNTVKESDLPHLPYLQAFVKETLRLHPPTPLLLPRR

ALETCTVMNYTIPKECQIMVNAWAIGRDPKTWDDPLNFKPERFLSSDVDYKGNDFELIPF

GGGRRICPGLPLASQFSNLIVATLVQNFEWSLPQGMSTSELSMDEKFGLTLQKDPPLLIV

LKARASNI

>CYP80C1 Populus trichocarpus (cottonwood)

MDQRFLQRLFSLVSSAEILFLLLLPLTFIILKNIIRSCSESKYLPPGPKPWPIIGNLLHV

GNQPHVSLAEIAKIHGPLISLRLGTQLLVVGSSAKAAAEILKTHDRFLSARHVPQVIPRE

SHVLRRVALVWCPESIDTWKLLRGLCRTELFSAKAIESSATLREKKVGELMDFLVAREGK

VVSIGEVVFSTVFNTISNLLFSNDLAGLEEKGMSSGLKSHVRKLMLLVATPNIADFYPIF

AGLDPQGLRRKLSKLVEETFAIWAINIKERRNSYVHDSPKRDFLDVFLANGFDDDQINWL

AAELFSAGTDTTATTIEWAVAEILKNKEVMKKVDEELEREITKNTISESDVSGLPYLNAC

IKETLRLHPPVPLLVPHRATETCEVMKYTIPKDSQVLVNVWAISRDPSTWEDPLSFKPDR

FLGSNLEFKGGNYEFLPFGAGRRICPGLPMANKLVPLILASLIRCFDWSLPNGEDLAKLD

MKDKFGVVLQKEQPLVLVPKRRL*

>CYP80D1 Populus trichocarpus (cottonwood)

MVSISVLANSYPSFPMLFLLAILLLLSLVLKHKSSKVPAIPPGPKSWPIIGNVLQMGNKP

HISLTKLAQVYGPLMSLRLGTQLVVVGSSREAASEILKTHDRELSGRCVPHASFAKDPKL

NEDSIAWTFECTDRWRFFRSLMRNELFSSKVVDGQSRTRETKAREMIDFLKKKEGEGVKI

RDIVFVYTFNVLANIYLSKDLIDYDQTGECQRVCGLVREMMELHTTLNISDLYPILGSLD

LQGVSRKCNECESRIQELWGSVIKERREGRNDTGDDDDNSSKRKDFLDVLLDGEFSDEQI

SLFFVQELLAAVSDSTSSTVEWAMAELMRNPQAMKQLREELAGETPEDLITESSLAKFPY

LHLCVKETLRLHPPAPFLIPHRATEDCQVLDCTIPKDTQVLVNVWAIARDPASWEDPLCF

KPERFLNSDLDYKGNHFEFLPFGSGRRICAGLPMAVKKVQLALANLIHGFDWSLPNNMLP

DELNMDEKYGITLMKEQPLKLIPKLRK*

>CYP80E1 Populus trichocarpus (cottonwood)

MATIVTEISSNTLFTILFLLPLIYLIAKQLKALYSSRFAPLPPGPYSWPILGNALQIGNS

PHITLASLAKTYGPLFSLRLGSQLVIVAASQEAATEILKTQDRFLSGRFVPDVIPAKWLK

LENLSLGWIGEVNNEFKFLRTVCQSKLFSNKALLSQSCLREKKAADTVRFIRTMEGKVLK

IKKVAFAAVFSMLTNILISSDLISMEQESTEGEMTEIIRNIFEVGAAPNISDLFPILAPF

DLQNLRKKSKELYLRFSTMFEAIIEERRERKMSSDNASGKEDFLDTLISNGSSNEHINVL

LLELLVAGSDTSTSAIEWAMAELLRNPQCMKKAQAELASEINQDLIQESDLPRLKFLHAC

LKESMRLHPPGPLLLPHRAVNSCKVMGYTIPKNSQVLVNAYAIGRDPKSWKDPLDYKPER

FLTSNMDFRGSNIEFIPFGAGRRACPGQPMATKHVPLVLASLLHFFDWSLPTGHDPKDID

MSDKFHTSLQKKQPLLLIPKIKN*

>CYP80F1 Hyoscyamus niger (black henbane)

MYIEDTSEIFTIFFTHILLPLLSFFIIRCVISSRKKLPLPPGPFPWPIIGHLFYLGNKPH

VSLAKLANVHGPHLMSIRLGGRLVIVASSPMATAEVLKTHDRLLSGRFVSHPMRVEGSYI

RNLATETLEECDENWKKVRSMYQIVLFSHKAVESQVNIREKKVMELVKFVASKEGELVNI

KGIAFVTILNILSNSTISNDLVDFEGKGIGEGMREWIRNYTKLEGVPQLADLFPILDGCT

WDFQGTYKKLKDTFERISDVWRDIINKKRMEISNKYYEGEDFADALIRNGFEDKQINALL

MELYSAGTETTITTVEWTLVELLKNPEAMKRLRNEIKKELTTIDDREIMIVKDSNLPNLP

YLEACMKETLRLHPPAPLLFPHRAVQTCEVMGYRIPQDTQIIVNVWKMARDSEYWNDDPW

SFKPDRFLDSSTDYKGHDFEFIPFGSGRRICAGQSLALRMLPMIVGSLVHNFELILPNNM

NPMEMNMDDIIDVTMAKKDPLFIIPKIRNS

>CYP80G1 Aquilegia formosa x Aquilegia pubescens (Ranunculales)

MDLQVALFSLIPIILVCILFFKSKHKNLPPGPHAWPLIGSLPVLFTNTEVPLHITLTNMA

RTHGPMMILWLGTQPTLVASTAEAAMEILKTKDRVCSGRHIRMSFRLKHHIKYSLVWSDC

TDYWKLLRKIARTEIFSPKMLQAQSHVREQKVGELVEYLRSKEGQVVKLTQFVFGTLLNI

LGNVVFSRDVFVYSDDDGNKDGIQNLIREMLMIGAEPNIAEFYPILEELDLQGLKRKCDE

RFLRVMKLWKGTVNERKEKRNEETKDMLDVLLANDFNDAQINALFLETFGPGSETSSATI

EWVMSELIKNPKEMAKVRKELDEVVGTSTVKESHLPQLPYLQACIKETMRLHPAAPFLLP

RRAIEACELMGYTIPKDCQILVNAYAIGRDPNSWTDPLTFRPERFFESDVDYHGGHYQFI

PFGSGRRTCVGMPLATRTIPLIVGSLVHTYDWGLPDGKRPEELELKEMLSLSLAIDPSLC

VVPKMRV*

>CYP80G2 Coptis japonica (Ranunculales)

MDLQIALFSLIPVILVFILLLKPKYKNLPPGPHPWPLIGNLPILFTNTEVPLHITLANMA

RTHGPIMILWLGTQPTVMASTAEAAMEILKTHDRIFSARHIRMSFRLKHHIKYSLVWSDC

TDYWKLLRKIVRTEIFSPKMLQAQSHVREQKVAELIDFLRSKEGQVVKISQFVFGTLLNI

LGNVVFSKDVFVYGDETDKGGIQNLIREMLMIGAEPNVAEFYPSLEELDLQGLKKKCDER

FIRVMKMWEGTVKERKANRNEESKDMLDVLLANDFNDAQINALFLETFGPGSETSSATIE

WVIAELIKSPKEMAKVRKELNEVVGTSTIKESDLPQLPYLQACIKEAMRLHPAAPFLLPR

RAAETCEVMGYTIPKNSQVLVNAYAIGRDPKSWKDPSTFWPERFLESDVDFHGAHYQFIP

FGSGRRTCVGMPLATRTIPLIVGSLVHNYDFGLPGGNRPEDLKMNEMLSLTLAIDPSLCV

VPKARA

>CYP80H1 Aquilegia formosa x Aquilegia pubescens (Ranunculales)

MEQSNLFIFILLLILFVLFLVLHHMRQKFSKYPPGPYPLPILGNFLHLRKTPHISLANLA

KVHGPLISLRLGAKLLVVASSQEAATEILKIHERVMSGRPVPLTIEAVSKGLDFFTLIGA

TSCTKNWKILRTIVKAELLSTEVVDKTTAAREEKVKELINLLSSKQGKVVNLGDYVFATA

ANTVSRLLFSKDCISLENQGMVGGFSKENIKTIVKMASTPNLGDHYAIFNGLDIQGLGKK

SVECLGRLYASWNSLIDGRHVSKCRDHVGKDKDFLDVLLANGFSKDQINLILSEMFITGI

DTTSTAVEWAMAELIKNQDAMTKLSEELAKEIGGNIIRDSDLPRLPYLNACVKETLRLHP

SVAMIPRRAVETCQVMNYTIPKNAEIWVNLWALGRDPTKWEDPLAFKPSRFLQSHLGFMG

SHFEYIPFGSGRRMCPGLPLAVRLVPLVLASLVHSFDWYLPENLSPGELNMDAKLGLTLQ

REKPLYLIPRPK*

>CYP80J1 Carica papaya

MQSLNLESILNNADSSATLPLLLLLFSIVFLVAIKQKSSRKLPLPPGPRPWPIVGNLPQL

GKKMHVSMADLAKTHGGLMSLRLGTQLVIVGSSPEAATEILKTHDKELCGRHVPMVSFAT

DPKLNKDSIAWSYECGKEWMAFRALMRTELFSTKIVETQYHVREKNVRAMVDYLSRKQGE

LVAMRDVVYIYTFNTLGNVYFTRDFMDFDGEEGRRVSALVREMMELWSAPNISDLYPILS

RFDLQGLRKKADVCVKKMCDLWDHSIRERREIRGKSRHDSSDLAPKNKDFLDILLESGFD

DEQISYIFLELLAAVSDGSTSTVEWALSEMVKNPEAMKKARQELSEQISESFVTESQLSK

LPYLHACIKETLRLHPPAPVLLPRRAAQDLEIMNHTIPKNAQVLVNVWAIARDEKIWKDP

MSFKPERFINSSVDYKGNDFEYLPFGAGRRICAGLPMATRQVLLALANLIHQFEWSLPDN

LRPEQLDMEELFGVTLLKENPLALIPKRKF*

>CYP80K1 Vitis vinifera (Pinot noir grape)

MDPDTVTADISIFSFLYALLLLPFLVILKHIFLKPPPLPPGPYPWPIIGNLLQMGKNPHA

KLANLAKLHGPLMSLRLGTQLMVVASSPAAAMEVLKTHDRALSGRYLSXSVPVKNPKLNH

LSIVFAKDCNTNWKNLRAICRMELFSGKAMESQVELRERKVTELVEFLATKEGEVVKVMD

LVFTTICNILSNKFFSMDLCDFEDEGRVGGALKDLIHKNAEFGATPNLSDYYPILGGLDI

QGINRKAKEMFERIPTTWEDILKERRTQRSNRSSHRDFLEALLEIGFEDDQINQVILELF

SAGAETSSLTVEWAMAELIRNQDAMDKLRGELRQIVGESPVRESHLPRLPYLQACVKEAL

RLHPPAPLLLPHLAAETCQVMGYTIPKDSQIFVNIWAMARDPKIWDDPLSFKPERFLDSK

LDFKGNDFEYIPFGAGRRICPGLALGGRQVPLILATFVHLFGWSLPGNMDSAQLDMEEWL

VITLRKEQPLRLVPRVRK

>CYP81A1 Zea mays

MERFYYVAVATFVLVFLLHHLLTRKKQQRLPPGPRFAYPILGHLPLLKKPLQTSFADLVS

RHGPIIHLRLGRRHAVVVGSAAVAKECFSGELDVAIANRPHFPSAREVTFDYSVLTAVNY

GALWRTMRRVSTVHLLSAHRVNVMSDTVIARELRVMVRRLARASASAPGDAARVELKRRL

FDLSHSVLMEIMAQTRNTYSDDPREEMSREARDMKDIIEEIIPLVGAANLWNYVPLLRWL

DLYGAKRKLADVVNRRDLIFDNMIGAERQKLRQLERKKGEAHASESDKMGMIGVMLSLQK

TEPDVYTDTFINALVSNLLAAGTETTSTTLEWAMSLLLNHPDVLKRAQEEIESNVGRDRL

LDKNDLPRLPYLHCIISETLRLYPPTPMLLPHEASTDCKIHGYDVPAGSMVLVNAYAIHR

DPAMWEDPEEFRPERFELGRAEGKFMMPFGMGRGRCPGENLAMRTMGLVLGALLQCFDWT

RVGDREVDMATATGTIMSKAVPLEAQCKPRANMSAVLQKI*

>CYP81B1v1 Helianthus tuberosus (Jerusalem artichoke)

MEIPYLLTTTLLLLFTTLYLLLRRRSSTLPPTIFPSLPIIGHLYLLKPPLYRTLAKLSAK

HGQILRLQLGFRRVLIVSSPSAAEECFTKNDIVFANRPKMLFGKIIGVNYTSLAWSPYGD

NWRNLRRIASIEILSIHRLNEFHDIRVEETRLLIQKLLSACNSGSSQVTMKFSFYELTLN

VMMRMISGKRYFGGDNPELEEEGKRFRDMLDETFVLAGASNVGDYLPVLSWLGVKGLEKK

LIKLQEKRDVFFQGLIDQLRKSKGTEDVNKKKTMIELLLSLQETEPEYYTDAMIRSFVLV

LLAAGSDTSAGTMEWVMSLLLNHPQVLKKAQNEIDSVIGKNCLVDESDIPNLPYLRCIIN

ETLRLYPAGPLLVPHEASSDCVVGGYNVPRGTILIVNQWAIHHDPKVWDEPETFKPERFE

GLEGTRDGFKLLPFGSGRRSCPGEGLAVRMLGMTLGSIIQCFDWERTSEELVDMTEGPGL

TMPKAIPLVAKCKPRVEMTNLLSEL

>CYP81C3 Populus trichocarpus (cottonwood)

MEFLYYHLALLFFLFIVVKNLFHRKRNLPPAPFALPVIGHLYLLKQPLYKSLHALLSRYG

PALSLRFGSRFVIVVSSPSVVEECFTKNDKIFANRPKSMAGDRLTYNYSAFVWAPYGDLW

RKLRRLAVAEIFSSKSLRKSSTVREEEVSCLIRRLLKVSTSGTQNVELRLLFSILASNVV

MIVSAGKRCVEEEHAGTKMEKQLFQDFKDKFFPSLAMNICDFIPILRVIGFKGLEKNMKK

LHGIRDEFLQNLIDEIRLKLKKTTSLKTDEVTDGEERRSVAEILLCLQESEPEFYTDEVI

KSTVLMMFIAGTETSAITLEWAMTLLLNHPKVMQKVKAEIDEHVGHGRLLNESDIVKLPY

LRCVINETLRLYPPAPLLLPHFSSEACTAGGFDIPQGTMLVVNAWTMHRDPKLWEEPNEF

KPERFEAGLGEGDGFKYIPFGIGRRVCPGASMGLQIVSLALGVLVQCFEWDKVGTVEDTS

HGLGMILSKAKPLEALCSPRRDLITLLSHL*

>CYP81D1 Arabidopsis thaliana

MEETNIRVVLYSIFSLIFLIISFKFLKPKKQNLPPSPPGWLPIIGHLRLLKPPIHRTLRS

FSETLDHNDGGGVMSLRLGSRLVYVVSSHKVAAEECFGKNDVVLANRPQVIIGKHVGYNN

ANMIAAPYGDHWRNLRRLCTIEIFSTHRLNCFLYVRTDEVRRLISRLSRLAGTKKTVVEL

KPMLMDLTFNNIMRMMTGKRYYGEETTDEEEAKRVRKLVADVGANTSSGNAVDYVPILRL

FSSYENRVKKLGEETDKFLQGLIDDKRGQQETGTTMIDHLLVLQKSDIEYYTDQIIKGII

LIMVIAGTNTSAVTLEWALSNLLNHPDVISKARDEIDNRVGLDRLIEEADLSELPYLKNI

VLETLRLHPATPLLVPHMASEDCKIGSYDMPRGTTLLVNAWAIHRDPNTWDDPDSFKPER

FEKEEEAQKLLAFGLGRRACPGSGLAQRIVGLALGSLIQCFEWERVGNVEVDMKEGVGNT

VPKAIPLKAICKARPFLHKIIS*

>CYP81E1v2 Glycyrrhiza echinata AB022732

MDILSLLSYSVFYLALFFIFNIVIRARKFKNLPPGPPSLPIIGNLHHLKRPLHRTFKGLS

EKYGHVISLWFGSRLVVVVSSASEFQQCFTKNDVVLANRPRFLSGKYIFYNYTTLGSTSY

GEHWRNLRRITALDVLSNHRINSFSGIRRDETQRLITRLADDSSTNFAEIELSYRFYDMT

FNNIMRMISGKRYYGEDCDMSDLQEASQFRDMVSELLQLSGANNKTDFMPLLRFLDFENL

EKRLKDISGKTDAFLRGLIQEHRAKKERANTMIDHLLNLQDSQPEYYTDQIIKGLALAML

LAGTDSSAVTLEWSMSNLLNHPEVLKKVKDELDTHVGQDRLVDESDLPKLSYLKNVINET

LRLYTPAPLLLPHSTSDECNIGGYKVPQDTIVLINAWAIHRDPELWTEATTFKPERFEKK

GELEKLIAFGMGRRACPGEGLAIRAISMTLALLIQCFDWKLTNGDKIDMAERDGFTLTKL

VPLKAMCKSRPVINKVFKQ

>CYP81F1 Arabidopsis thaliana

MLYFILLPLLFLVISYKFLYSKTQRFNLPPGPPSRPFVGHLHLMKPPIHRLLQRYSNQYG

PIFSLRFGSRRVVVITSPSLAQESFTGQNDIVLSSRPLQLTAKYVVYNHTTVGTAPYGDH

WRNLRRMCSQEILSSHRLIIFQHIRKDEILRMLTRLSRYTQTSNESNDFTHIELEPLLSD

LTFNNIVRMVTGKRYYGDDVNNKEEAELFKKLVYDIAMYSGANHSADYLPILKLFGNKFE

KEVKAIGKSMDDILQRLLDECRRDKEGNTMVNHLISLQQQQPEYYTDVIIKGLMMSMMLA

GTETSAVTLEWAMANLLRNPEVLEKARSEIDEKIGKDRLIDESDIAVLPYLQNVVSETFR

LFPVAPFLIPRSPTDDMKIGGYDVPRDTIVMVNAWAIHRDPEIWEEPEKFNPDRYNDGCG

SDYYVYKLMPFGNGRRTCPGAGLGQRIVTLALGTLIQCFEWENVKGEEMDMSESTGLGMR

KMDPLRAMCRPRPIMSKLLL

>CYP81G1 Arabidopsis thaliana

MIDLFLLALMAGFIAVAYVFRSKQKKNLPPNPVGFPVIGHLHLLKEPVHRSLRDLSRNLG

IDVFILRLGSRRAVVVTSASAAEEFLSQQNDVVFANRPLATLTEYMGYNNTLVSTAPYGE

HWRRLRRFCAVDILSTARLRDFSDIRRDEVRAMIRKINVELVTSGGSVRLKLQPFLYGLT

YNILMSMVAGKREEDEETKEVRKLIREVFDFAGVNYVGDFLPTLKLFDLDGYRKRAKKLA

SKLDKFMQKLVDEHRKNRGKAELEKTMITRLLSLQESEPECYTDDIIKGLVQVMLLAGTD

TTAVTLEWAMANLLNHPEVLRKLKTELNEVSKEGRVFEESDTGKCPYLNNVISETLRLFP

AAPLLVPHASSTDCEVAGFDIPRRTWLFINAWAIQRDPNVWDDPETFKPERFESETHRGK

FLPFGIGRRACPGMGLAQLVLSLALGSLIQCFDWERDNDVAVDMSEGKGLTMPKSVPLVA

KCKSLPILDKLVL*

>CYP81H1 Arabidopsis thaliana

MDCILLILTTLVAIFIVKIVLLVTKPNKNLPPSPNICFPIIGHLHLLKKPLLHRTLSHLS

HSLGPVFSLRLGSRLAVIISSPTAAEECFLTKNDIVLANRPRFIMGKYVAYDYTSMVTAP

YGDHWRNLRRITALEVFSTNRLNASAEIRHDEVKMLLQKLHDLSVERPAKVELRQLLTGL

TLNVIMRMMTGKRFFEEDDGGKAGISLEFRELVAEILELSAADNPADFLPALRWFDYKGL

VKRAKRIGERMDSLLQGFLDEHRANKDRLEFKNTMIAHLLDSQEKEPHNYSDQTIKGLIL

MMVVGGTDTSALTVEWAMSNLLNHPQILETTRQNIDTQMETSSSRRLLKEEDLVNMNYLK

NVVSETLRLYPVAPLMVPHVPSSDCVIGGFNVPRDTIVLVNLWAIHRDPSVWDDPTSFKP

ERFEGSDQFGHYNGKMMPFGLGRRACPGLSLANRVVGLLLGSMIQCFEWESGSGGQVDMT

EGPGLSLPKAEPLVVTCRTREMASELLFFGSEPSNKNV*

>CYP81J1 Hemerocallis hybrid cultivar (daylily)

STEIFSPVRIRSLAAVRQEEVKLMITGILASTSTDNSVKVNMKVVFSELMFNVIMKIIAG

KRYFGVNTDSEVEEGQKFRVVFDEMFSTLEVASPQDFLPFLKWFGFKRMENRLTKLAKEL

DQLFQKLIEERRSERGKVQSTVIDVLLSLQETDREQYSDKLIKGMILSLIAAGTHTTAGT

MEWAMSLLLNHPEALLKVRDEIDKKVGQDRLIDHSDLQNLSYLNNAIKESLRLFPTAPLL

LAHESSAECTVGGFTIPSNTILFANAYALHRDPKVWTDPVSFKPERFENNGQQGSRIYVP

FGLGRRSCPGEGLATQVVGLALGTLIQCFEWDRNGEEKVDMTDGSGLAMHMEKPLEAMCK

PRQSIVDVINRL

>CYP81K1 Arabidopsis thaliana

MEDLWFIFFSFLTATLIFFITKKFLWSLNSKLPPSPTPLPIIGHLHLIKKYPLPQALRHL

SSNYGPVLFLKFGCRNVLTLSSPDSIEECFTNHDVTLANRPKTITSDHFSYGYKNFGFAP

YGDLWRTLRRLSTLEVFSSASLQKNSSIRNEEVSNLCLIIFRLSRDSRIVDLKYQFTLLT

AHIMLRLVSGKRGVKKSDPESEKRFLDDFKLRFFSSMSMNVCDYFPVLRWIGYKGLEKRV

IDMQRMRDEYLQRLIDDIRMKNIDSSGSVVEKFLKLQESEPEFYADDVIKGIIVLMFNGG

TDTSPVAMEWAVSLLLNHPDKLEKLREEIKSNVKHKGLIQDSDLSSLPYLRCVIYETLRL

YPAAPLLLPHCSSKRFNLGNYEIPENIMLLVNAWAVHRDGELWEEANVFKPERFEGFVGD

RDGFRFLPFGVGRRACPAAGLGMRVVSLAVGALVQCFEWEKVEAGDIDMRPVFGVAMAKA

EPLVALPKPWSEMVPILSQL

>CYP81L6 Zea mays (maize)

MDVALSTIVFVIAIFIPALLTLVQRSRSRHAGHNPPPPPEPRAIPLVGHLHHLLRKKPLH

RCLAHLAERHGDVLGLRFGSSRVAVVSSASVAQQCLVALDTSFGNRPRLPSARILSYEWS

TMGHSNCGPYWRQARRTTSTEFSSVERVQHFADVHEQEARAMARRLCRVAHASGGRALVD

VKSRLLEMLMNGLLDMLFRRTTSRSRSSDEKDEAVEVSEEARCFMAMAEETMELTLTVWD

FLPPLARWLDVDAVGRRLQRLQANRTEFLQRLIEEHKEMEKSGQVTRRTLVGVMLELQDK

DPEAYTDQLIRSLCVSALEAGTLSTGYTIEWVMSLLLNNPHIMKKARDEIDACVGEPKRL

LDATDLPKLPYLRCIILETLRLYPVVPLLVPRESSTNCTVNGFNIAKGTMLLVNTFAIHR

DPRTWDDPETFLPERFEDGSNQSGKTTMDLSFGMGRRRCPAENLGMQLAGIALGTMIQCF

NWERVGTELVDMAEGSGLTMAKKVPLEAFCQPRASMVDLLANI*

>CYP81M1 rice

MANTTLSSLLFLSMASALFLLTLLRILRSKKQQRPPPPPAEPAVPPRHRGHLHLFKKPLH

RALSGLAATHGPVLLLHFGSRAVLHVTDPAVAEECLTDHDVTFANRPRLPSSCHLSNGYT

TLGSSSYGPNWRNLRRIATVEVFSAHRLLRSADVRGGEVPHMARWLYLAAPAAGPSEPAR

ADVKARAFELVLNVVARMVAGKQYYGGEGDAEAETEEAARFREMVREYFAMHGASNLQDF

VLLLGLVDIGGAKRRAVKLSRERNTWAQRLIDEHRATATAAAATEARTMVGDLLKMQASE

PEAYSDKVITALCLSILQTGTDTSSSTIEWGMALLLNHPAAMAKARAEIDRFVGTGRVVE

EADLPNLPYLQCIIRENLRLYPVGPLLAPHESSADCSVSVAGGGRYAVPAGTMLLVNVHA

MHRDARFWGPDPESFSPERFEGGRSEGKWMLPFGMGRRRCPGEGLAVKVVGLALATLVQC

FEWRRVGDEEVDMTEGSGLTMPKAVPLEALYWPRPEMVPALSGIFFIYNFFY*

>CYP81N1 rice

MTGGLEVAMVAGGGNGGAAVLVGITVLLFVVVVVVVVLVRWWSGGEGGAAPSPPALPVLG

HLHLLKKPLHRSLAAVAAGVGAPVVSLRLGARRALVVSTHAAAEECFTACDAALAGRPRT

LAGEILGYDHTIVLWTPHGDHWRALRRFLAVELLSAPRLAALAADRHAEAASLVDAILRD

AAGGAKVTLRPRLFELVLNVMLRAATTRRRHASVDARKLQEIIEETFSVNGTPSVGDFFP

ALRWVDRLRGKVGSLKKLQARRDAMVTGLIDDHRQWRSGSAGDGDQDKEKKGVIDALLAL

QETDPDHYTDNVVKGIILSLLFAGTDTSALTIEWAMAQLVTHPETMKKARAEIDANVGTA

RLVEEADMANLPYIQCVIKETLRLRTAGPVIPAHEAMEDTTVGGFRVARGTKVLVNAWAI

HRDGDVWDAPEEFRPERFVDSDAGGAVTAPMMPFGLGRRRCPGEGLAVRVVGVSVAALVQ

CFDWEVGDDDVVDMTEGGGLTMPMATPLAAVCRPREFVKTILSTS*

>CYP81P1 rice

MEISQAFVFASLLLLLLLTWLLFHLLSYQAPPPNGDGGRRIPSPPALPVVGHLHLLKKPL

HRSLAALAARYGGGAGLLLLRFGARPVVLVSSQAAADECFTAHDAALAGRPGLASRRLLT

DGCPTIATAGHSARWRHLRRLATVHALCARRLAATSPARDAEARAMAARLYSSSSSSSAA

SAVVVGVKPAAYGFVASVIMSMVAGERMAEEDVLRFKAITEAGLAAAGAANRQDFLPFLR

LLDFGRARRRLAGIAKERHDFGQRIVDEYRRRHRRRLAVAADDFSSSPPRRTVIGDLLRQ

QESSPESYADEVIRTVCLSLLQAGTDTSASTIEWAMALLLNNPDVLRKATDEINSVVGMS

RLLQEPDLANLPYLRCIITETLRLYPLAPHLVPHEASRDCMVAGHVIARGTMVLVDVYSM

QRDPRVWEDPDKFIPERFKGFKVDGSGWMMPFGMGRRKCPGEGLALRTVGMALGVMIQCF

QWERVGKKKVDMSEGSGLTMPMAVPLMAMCLPRVEMESVLKSL*

>CYP81Q1 Sesamum indicum

MEAEMLYSALALTFAIFMVYRILSNSQDKRSLTKLPPSPPGWLPVIGHAHLMKNLLHRTL

YDFSQKLGPIFSIRFGSRLVVVVSSSSLVEECFTKYDIVLANRPQASVDRRSLGFSTTSV

IGAPYGDHWRNLRKLCDLEVFAPTRLASFLSIRLDERDRMISALYKISSAGFAKVNLEAK

IVELTFNNIMRMVAAKRYYGEEAEDDEEAKRFRDLTKEALELTSASNPGEIFPILRWLGC

NGLEKKLAVHSRKTDEFMQGLLDEHRRGERQNTMVDHLLSLQESQPEYYTDEIITGLIVA

LIIAGTDASVVTTEWAMSLLLNHPKVLEKARKELDTLVGHERMVDEHDLPKLRYLHCIVL

ETLRLFPSVPTLVPHEPSEDCKIGGYNVPKGTMVLVNAWAIHRDPKVWDDPLSFKPDRFE

IMEVETHKLLPFGMGRRACPGAGLAQKFVGLALGSLIQCFDWERTSPEKIDLNEGSGITL

PKAKTLEAMCKPRHVMEKVLRQVSNV

>CYP81Q7 Vitis vinifera

MEITWLSTSLCLLFLSFAFNIFLQRRRIHPHLPPSPPAIPILGHLHLLLKPPIHRQLQSL

SKKYGPIFSLRFGSSPVVIISSPSTVEECFTKNDIIFANRPRWLIGKYIGYNYTTIASAS

YGEHWRNLRRLSALEIFSSNRLNMFLGTRRDEIKILLHRLSQNSRDNFARVELRPMFTEL

TCNIIMRMVTGKRYYGEDVDSEEAKRFQKIMRGIFELAGASNPGDFLPLLRWVDFGGYEK

KLVKLNREKDVIFQGLIDEHRSPDQGLVNKNSMIDHLLSLQKSEPEYYTDEIIKGLALIL

TFAGTDTTATTIEWAMSLLLNHPDVLKKARAELDTHVGKDRLMEESDFPKLQYLRSIISE

TLRLFPATPLLIPHISSDNCQIGGYDIPRGTILLVNAWAIHRDPKSWKDATSFKPERFEN

GESEAYKLLPFGFGRRACPGAGLANRVIGLTLGLLIQCYEWERVSEKEVDMAEGKGVTMP

KLEPLEAMCKARAIIRKVL*

>CYP81R2 Populus trichocarpus (cottonwood)

MNYMYYCLAFFLSSFLVFKLVFQRSRNLPPSPFGFPIIGHLHLVSKPPMHKVLAILSNKC

GPVFTLKLGSRNIVAVCSLSAAEECYIKNDIVFANRPQSIFVHYWSYNYAAFLFAPYGHL

WRTLRRFSVTELFSRSCLDRSAAISEEVRTLVRLILSKVSDDGAKKVDLNYFFTITSLNV

IMKMNAGKKWVEEEKAACIDSGKQCIEDVQKIFPSNPGTTVLDFFPFLKWFGYRGEEESV

IKVYKERDEFLQGLIEEVKRKETSSVTSNPAEGVKDQTTVIGSLLALQKSDPELYTDEVV

KGTMATLYLAGVDTVDFTTEWAMTFLLNHPERLERVKAEIDREVGHERLVQESDLPKLRY

VRCVVNETLRLYPPAPLLLPHAPSEDCIVGGYKIPRGTIVMVNAWAIHRDPKLWEDPESF

KPERFEGLNNEGEKQGFIPFGIGRRACPGNHMAMRRVMLALAALIQCFEWERVGKELVDM

SIVDALISVQKAKPLEAICTPRPFTTTLISPP*

>CYP81S1v1 Populus trichocarpus (cottonwood)

MEEDYSASLWLRYSFLLPCMVFLVLSTKFLLHKRKQGKINHLPPSPFALPIIGHLYLLKQ

PIHRTLHSLSKKYGPIFSIKLGSRLAVVISSPSAVEECFTKNDIVLANRPYFLSSKYLNY

NNTTMGSVEYGEHWRNLRRISALEIFSPPRLTSLFSIRREEVMALLRRLHGVSKHGNYAK

VELRSMLLDLTSNIIMRMVAGKRYYGEDVKEIEEARIFKEIMEEFAECIAVRNLGDMIPL

LQWIDFTGHLKKLDRLSKKMDVFLQGLVDEHRDDRDRNTMINRFLALQEEQPEYYTDEVI

KGHVLVLLIGGTETAATSMEWALANLLNHPNVLKKAKAELDAQVGDRLIDESDFAKLHYL

QSIISENLRLCPVTPLIPPHMPSSDCTIGGYHVPAGTILFVNAWSLHRDPTLWDEPTSFK

PERFESAGRVDACKFIPFGMGRRACPGDGLANRVMTLTLGSLIQCFEWERVGENKIDMTE

KTAMTMFKVEPLELMCRARPILDMLLSLSGQKI*

>CYP81T1 Populus trichocarpus (cottonwood)

MEVSHWFNFAALFFFFVLASKLVIYKLGNPKNLPPSPPSRPIIGHLHLLKQPIHRTLCEL

SKKYGDILFLRFGARKVLVISSPSAVEECFTRKDVIFANRPRTLAGKHLNYNSTTMGFSS

YGEHWRNLRRLTTIELFSASRVASFSDIRKEEVQLLLNQLFRDSSKQQAKVGLTASFMEL

TFNVMMRMIAGKRYYGKEVVDEEAGQFQNIIKEMEALRGSSNMNDFFPVLQWIDFQGLEK

RMMGLKKKMDKFLQDLIEEHQKVRSQSSQSTKITGLGNQKRNMTLIDVMLSLKETEPEFY

TDQTIKGVIMSTLTAGSQTSAATLEWAMSLLLNNPETMRKASEEIDAIVGTEHILDEVDV

TKLSYLQNILNETFRLFPPAPLLLPHESSEDCTISGFHVPRGTMLLVNTWSIHRDTKLWV

EPTKFMPERFEGGEGEGYKLLPFGAGRRACPGAGLAKRIIGLTLGVLIQCFEWDRVSKEE

INLTEGTGLTIPKAEPLEALCRPRQSMVNLLSSM*

>CYP81U1 Iris hollandica (Iris, monocot)

MVGILVYVALFILSLLLFLTAAAKLHNSKSKIKNQAPSPPSLPVVGHLHLLKKPLHRSIS

LLSARHGPILLLRFGSRPALAVSSLPLAEECLSGKNDLAFANRAHFPHEAAPLQLLTLGS

ANYGPHWRMLRRISAVELLSSHRINSFSQLRSEEVHSMISTLFRESSDKELNRVELKSKL

FELAMNNMMRMIFGKDLASSEGAGRFREMVKESHSLLGASTRLGDFFPFLGWMDWRARRM

VLRLVRRRDEFLQSLIDAHARKMEEVEEKTMIRVLVELQKSNRESNNDEGFMLKPLIIGL

LQAGTDTSSDTIEWAMSLLLNNRDKLKKARDEIDARVGKERLLRESDLPNLPYLQCVITE

TLRLYPAAPLLVPHESAEECTVGGYAVPQGTMLLVNAYAIRVVGIVLGTLIQCFEWERVG

EEEVDMTEGSGLTLPRANPLEAICRPRQSMISVLAGL

>CYP81W1 Vitis vinifera

MGNLYHYAVILLPIILIIKFLFHGRQRQRYRLPPSPFALPVIGHLHLLKPPLYQGLQALS

SQYGPILFLRFGCRPFVVVSSPSAVQECFTKNDVVLANRPRSMIGDHVTYNYTAFAWASY

GHLWRVLRRLTVVEILSSNKLLLLSTVREEEVRYLLRQLFKVSNDGAQKVDMRLYLSLFS

FNFIMKTITGKRCIEEEAEGIETNRQFLERLKRIFVPTTTTNLCDFFPILRWVGYKGLEK

SVIQFGKERDGYLQGMLDEFRRNNSAVEWQKKRTLIETLLFLQQSEPDFYTDDVIKGLML

VISAGTDTSSVTLEWAMSLLLNHPEALEKARAEIDSHVKPGHLLDDSDLAKLPYLRSVVN

ETLRLYPTAPLLLPHLSSEDCSVGGFDIPRGTTVMVNVWALHRDPRVWEEATKFKPERFE

GMENEEKEAFKFAPFGIGRRACPGAALAMKIVSLALGGLIQCFEWERVEAEKVDMSPGSG

ITMPKAKPLEIIFRPRPTMTSLLSQL*

>CYP81X1 Soybean

MEEEADYRLIVITASVGFLLLFLYVLKSILLKSKNLPPSPPYALPLIGHLHLIKEPLHLS

LHKLTDKYGPIIFLCLGTRKVLVVSSPSAVEECFTKNDITFANRPQTLAAKHLNYNKTTI

GVASYGHYWRNLRRLTTVELFSTTRLAMLTSVRVEEVQLMVKQLFEECKGRQQIMIDLRA

RLLEVSFNIMLRMISGKRYYGKHAIAQEGKEFQILMKEFVELLGSGNLNDFFPLLQWVDF

GGVEKKMVKLMKKMDSFLQKLLDEHCTRRNVMSEEEKERRKSMTLIDVMLDLQQTEPEFY

THETVKGVILAMLVAGSETSATTMEWAFSLLLNHPKKMNKVKEEIDTYVGQDQMLNGLDT

TKLKYLQNVITETLRLYPVAPLLLPHESSNDCKVCGFDIPRGTMLLVNLWTLHRDANLWV

DPAMFVPERFEGEEADEVYNMIPFGIGRRACPGAVLAKRVMGHALGTLIQCFEWERIGHQ

EIDMTEGIGLTMPKLEPLVALCRPRQSMIKVLSNI*

>CYP82A6 Pisum sativum

MDFVLNYLNTTTIAFISLISLLFFLFRFSKVSHTKEPPIVSGSWPLLGHLPLMRNTQTPH

KTLGALVDKYGPIFTIKLGATNALVLSNWELAKECFTKNDIVVSSRPKPVAVELMSYNQA

FIGWAPYGTYWRQLRKIVTLEILSNRRIELLSHIRVSEVQTSIKELVNVWSNQMSSQYGL

LDDTKSSSTNDYASVELKKWFAQLTLNMVLRMVVGKRCFGDVDVENKEEAKKFLENIRDF

MRLIGTFTVGDGVPFLKWLDLGGHEKEMKKCAKKFDEMLNEWLEEHREKKGLGSEDKVVG

ERDFMDAMLLVLKDKPIEGFDVDTIIKATTLELILGGSDTTAGTLTWAMCLLLKHPHVLE

KLKEELNTYIGKERCVKESDINKLVYLHAIIKETLRLYPPAPFSSPREFTEDCTIGGYHI

KKGTRLMPNLWKIHRDPNVWPDPLEFKPERFLSTHKDVDVRGQNFELLPFGSGRRMCAGM

SLGLHMVHYILANFLHSFEILNPSPESIDVTEVLEFVTTKATPLEVLVKPCLSFKCYESM

>CYP82B1 Eschscholzia californica (California poppy)

MEKPILLQLQAGILGLLALICFLYYVIKVSLSTRNCNQLVKHPPEAAGSWPIVGHLPQLV

GSGKPLFRVLGDMADKFGPIFMVRFGVYPTLVVSTWEMAKECFTSNDKFLASRPPSAASS

YMTYDHAMFGFSFYGPYWREIRKISTLHLLSHRRLELLKHVPHTEIHNFIKGLFGIWKDH

QKQQQPTGREDRDSVMLEMSQLFGYLTLNVVLSLVVGKRVCNYHADGHLDDGEEAGQGQK

LHQTITDFFKLSGVSVASDALPLLGLFDLGGKKESMKRVAKEMDFFAERWLQDKKLSLSL

SSETNNKQNDAGEGDGDDFMDVLMSILPDDDDSLFTKYSRDTVIKATSLSMVVAASDTTS

VSLTWALSLLLNNIQVLRKAQDELDTKVGRDRHVEEKDIDNLVYLQAIVKETLRMYPAGP

LSVPHEAIEDCNVGGYHIKTGTRLLVNIWKLQRDPRVWSNPSEFRPERFLDNQSNGTLLD

FRGQHFEYIPFGSGRRMCPGVNFATLILHMTLARLLQAFDLSTPSSSPVDMTEGSGLTMP

KVTPLKVLLTPRLPLPLYDY

>CYP82C2 Arabidopsis thaliana

MDTSLFSLFVPILVFVFIALFKKSKKPKHVKAPAPSGAWPIIGHLHLLSGKEQLLYRTLG

KMADQYGPAMSLRLGSSETFVVSSFEVAKDCFTVNDKALASRPITAAAKHMGYDCAVFGF

APYSAFWREMRKIATLELLSNRRLQMLKHVRVSEISMVMQDLYSLWVKKGGSEPVMVDLK

SWLEDMSLNMMVRMVAGKRYFGGGSLSPEDAEEARQCRKGVANFFHLVGIFTVSDAFPKL

GWFDFQGHEKEMKQTGRELDVILERWIENHRQQRKVSGTKHNDSDFVDVMLSLAEQGKFS

HLQHDAITSIKSTCLALILGGSETSPSTLTWAISLLLNNKDMLKKAQDEIDIHVGRDRNV

EDSDIENLVYIQAIIKETLRLYPAGPLLGHREAIEDCTVAGYNVRRGTRMLVNVWKIQRD

PRVYMEPNEFRPERFITGEAKEFDVRGQNFELMPFGSGRRSCPGSSLAMQVLHLGLARFL

QSFDVKTVMDMPVDMTESPGLTIPKATPLEILISPRLKEGLYV

>CYP82D1 Medicago sativa (alfalfa)

MDVTIEYLYTIVAGVICIILISYSKFFRGDARAQPKLPPLASGGWPLIGHLHLLGSSNQP

PYITLGNLADKYGPIFTLRVGVHNAVVVSTWELAKEIFTTHDVIISSRPKFTAAKILGHD

YANFGFSPYGDYWQMMRKVTASELLSTRRFETLRDIRDSEVKKSLMELCNSGFDGDLKVE

MKRFLGDMNLNVIMRMIAGKRYSNNESGDEREVRKVRWVFREFFRLTGLFVVGDAIPFLG

WLDLGGHVKEMKKAAREMDSVVCGWLEDHRHKNVIGETKMEQDFIDVLLSVLHGVHLDGY

DVDTVIKATCLTLIAGATDTTTVTITWALSLLLNNRHTLKKVQDELDEKVGKDRLVNESD

INNLVYLQAVVKETLRLYPAGPLSGARQFTKDCTVGGYNIRAGTRLILNLWKMHRDPRVW

SEPLEFQPERFLNTHKDVDVKGQHYELLPFGGGRRSCPGITFGLQMTNLALASFLQAFEV

TTPSNAQVDMSATFGLTNIKTTPLEVIAKPRLPYHLLFVKEH

>CYP82E1 Nicotiana tabacum (tobacco)

MYHLLSPIEAIVGLVTFAFLLYLLWTKKQSKILNPLPPKIPGGWPVIGHLFYFNNNGDDD

RHFSQKLGDLADKYGPVFTFRLGFRRFLAVSSYEAMKECFSTNDIHFADRPALLYGEYLC

YNNAMLAVAKYGPYWKKNRKLVNQELLSVSRLEKFKHVRFSIVQKNIKQLYNCDSPMVKI

NLSDWIDKLTFDIILKMVVGKTYNNGHGEILKAAFQKFMVQAMEIELYDVFHIPFFKWLD

LTGNIKAMKQTFKDIDNIIQGWLDEHIKKRETKDVGGENEQDFIDVLLSKRSNEHLGDGY

SHDTTIKATVFTLVLDATDTLALHIKWVMALMINNKNVMKKAQEEMDTIVGRDRWVEEND

IKNLVYLQAIVKEVLRLHPPAPLSVQHLSVKDCVVNGYHIPKGTALLTNIMKLQRDPQIW

VDPDTFDPERFLTTNAAIDYRGQHYELIPFGSGRRACPAMNYSLQVEHLSIAHLIQGFNF

ATTTNEPLDMKQGVGLTLPKKTDVEVLITPRLPPTLYQY

>CYP82F1 Arabidopsis thaliana

MDLIMLFLLSALFIFPVLILIKSRLRPKNKKSTAPMVPGAWPLLGHLHLFDTVNPTHVTF

GAMADVYGPVFMAKLGSIKVMIINSKEVAKEIYTVHDKLLERPELTASKLLGYNDSFLTF

SPYGLYWREIRKIAVSELFSTSGVDMHMVSRAREADLAFRALYVRWEKRGKPKEGVLVDM

KQEFIDLTANISLMMVSGKRYFGENPNCEVKEARRCGKLIREFLDYFALFLLSDVAPVLG

FLDWKTKRGMKRTAKGLDKVAEGWIEEHKNKRSDHGRSENDYLDILIKILGQDKIPGLSD

THTKIKALCLNLVLAGSETAIVVLVWAVSLLLNNPHVLRKAQEELDSKIGKERVVEELDI

KDLVYLQAIVKETFRLYPPVPLVAYRAVVEDFDIAFCKCHVPAGTQLMVSAWKIHRDPNV

WSNPEQFEPERFLTSNRELDVGGQSYKFFPFGLGRRSCPAIPLGMRMVHYLLVRFLHSFD

LARPSSQDVDMTESNGLVNHKATPLEVNIIPRLHKSLYEVDHIGTDN

>CYP82G1 Arabidopsis thaliana

MTFLFSTLQLSLFSLALVIFGYIFLRKQLSRCEVDSSTIPEPLGALPLFGHLHLLRGKKL

LCKKLAAMSQKHGPIFSLKLGFYRLVVASDPKTVKDCFTTNDLATATRPNIAFGRYVGYN

NASLTLAPYGDYWRELRKIVTVHLFSNHSIEMLGHIRSSEVNTLIKHLYKGNGGTSIVKI

DMLFEFLTFNIILRKMVGKRIGFGEVNSDEWRYKEALKHCEYLAVIPMIGDVIPWLGWLD

FAKNSQMKRLFKELDSVNTKWLHEHLKKRSRNEKDQERTIMDLLLDILPEDIVISGHVRD

VIVKATIL*ALTLTGSDSTSITLTWAVSLLLNNPAALEAAQEEIDNSVGKGRWIEESDIQ

NLKYLQAIVKETHRLYPPAPLT*GIREAREDCFVGGYRVEKGTRLLVNIWKLHRDPKIWP

DPKTFKPERFMEDKSQCEKSNFEYIPFGSGRRSCPGVNLGLRVVHFVLARLLQGFELHKV

SDEPLDMAEGPGLALPKINPVEVVVMPRLDPKLYSLL*

>CYP82H1 Ammi majus L., Apiaceae (Bishops weed)

MITCEMGIYLQMQDIILFSLVFFSTLILWRIFSTYVIRKKTCSGPPEPAGRWPLIGHLHL

LGGSKILHHILGDMADEYGPIFSLNLGINKTVVITSWEVAKECFTTQDRVFATRPKSVVG

QVVGYNSRVMIFQQYGAYWREMRKLAIIELLSNRRLDMLKHVRESEVNLFIKELYEQWSA

NGNGSKVVVEMMKRFGDLTTNIVVRTVAGKKYSGTGVHGNEESRQFQKAMAEFMHLGGLL

MVSDALPLLGWIDTVKGCKGKMKKTAEEIDHILGSCLKEHQQKRTNISNNHSEDDFIYVM

LSAMDGNQFPGIDTDTAIKGTCLSLILGGYDTTSATLMWALSLLLNNRHVLKKAQDEMDQ

YVGRDRQVKESDVKNLTYLQAIVKETLRLYPAAPLSVQHKAMADCTVAGFNIPAGTRLVV

NLWKMHRDPKVWSDPLEFQPERFLQKHINVDIWGQNFELLPFGSGRRSCPGITFAMQVLH

LTLAQLLHGFELGTVLDSSIDMTESSGITDPRATPLEVTLTPRLPPAVYQ

>CYP82J1 Populus trichocarpus (cottonwood)

MDFSFHLLAVSTVLALVLWYTLRRVRETRRKTEKGLQPPEPSGALPLIGHLHLLGAQKTL

ARTLAAMADKYGPIFTIRLGKHPTVVVSNLEAIKECFTTHDRILSSRPRSSHGEHLSYNY

AAFGFNNSGPFWREMRKIVTIQLLSSHRLKSLRHVQVSEVNTLINDLYLLSKSNKQGSTK

IDISECFERMTINMITRMIAGKRYFSSTEAEKEDEGKRIGKLMKEFMYISGVFVPSDVIP

FLGWMNNFLGSVKTMKRLSRELDSLMESWIQEHKLKRLESTENTNKMEDDDFIDVMLSLL

DDSMFGYSRETIIKATAMTLIIAGADTTSITLTWILSNLLNNRRSLQLAQEELDLKVGRE

RWAEDSDIGNLVYIQAIIKETLRLYPPGPLSVPHEATKDFCVAGYHIPKGTRLFANLWKL

HRDPNLWSNPDEYMPERFLTDHANVDVLGHHFELIPFGSGRRSCPGITFALQVLHLTFAR

LLQGFDMKTPTGESVDMTEGVAITLPKATPLEIQITPRLSPELYYEC*

>CYP82K1 Populus trichocarpus (cottonwood)

MIIWRILSTSHKRNKTLPPPEPSGAWPLIGHLRILNSQIPFFRILGDLAVKHGPVFSIRL

GMRRTLVISSWESVKECFKTNDRKFLNRPSFAASKYMGYDDAFFGFHPYGEYWLEMRKIA

TQELLSNRRLELLKHVRVSEIETCIKELHTTCSNGSVLVDMSQWFSCVVANVMFRLIAGK

RYCSGIGKDSGAFGRLVREFFYLGGVLVISDLIPFTEWMDLQGHVKSMKRVAKELDHVVS

GWLVEHLQRREEGRVRKEEKDFMDVMLESLAVGDDPIFGYKRETIVKATALNLILAGTDT

TSVTLTWALSLLLNHTEVLKRAQKEIDVHVGTTRWVEESDIKNLVYLQAIVKETLRLYPP

GPLLVPRESLEDCYVDGYLVPRGTQLLVNAWKLHRDARIWENPYEFHPERFLTSHGSTDV

RGQQFEYVPFGSGRRLCPGISSSLQMLHLTLSRLLQGFNFSTPMNAQVDMSEGLGLTLPK

ATPLEVVLTPRLENEIYQH*

>CYP82L1 Populus trichocarpus (cottonwood)

MILEALILVFLYGFWKILARNSEGKKSTRAPEPSGAWPLFGHLPSLVGKDPACKTLGAIA

DKYGPIYSLKFGIHRTLVVSSWETVKDCLNTNDRVLATRAGIAAGKHMFYNNAAFALAPY

GQYWRDVRKLATLQLLSNQRLEMLKHVRVSEVDTFIKGLHSFYAGNVDSPAKVNISKLLE

SLTFNINLRTIVGKRYCSSTYDKENSEPWRYKKAIKKALYLSGIFVMSDAIPFLEWLDYQ

GHVSAMKKTAKELDAVIRNWLEEHLKKKIDGELGSDRESDFMDVMISNLAEGPDRISGYS

RDVVIKATALILTLTGAGSTATTLVWTLSLLLNNPTVLKAAQEELDKQVGRERWVEESDI

QNLKYLQAIVKETLRLYPPGPLTGIREAMEDCSIGGYDVPKGTRLVVNIWKLHRDPRVWK

NPNEFKPDRFLTTHADLDFRGQNMEFIPFSSGRRSCPAINLGLIVVHLTLARILQGFDLT

TVAGLPVDMIEGPGIALPKETPLEVVIKPRLGLELY*

>CYP82M1v1Nicotiana tabacum var. 4407 (tobacco)

MDYHISFHFQALLGLLAFVFLSIILWRRTLTSRKLAPEIPGAWPIIGHLRQLSGTDKNIP

FPRILGALADKYGPVFTLRIGMYPYLIVNNWEAAKDCLTTHDKDFAARPTSMAGESIGYK

YARFTYANFGPYYNQVRKLALQHVLSSTKLEKMKHIRVSELETSIKELYSLTLGKNNMQK

VNISKWFEQLTLNIIVKTICGKRYSNIEEDEEAQRFRKAFKGIMFVVGQIVLYDAIPFPL

FKYFDFQGHIQLMNKIYKDLDSILQGWLDDHMMNKDVNNKDQDAIDAMLKVTQLNEFKAY

GFSQATVIKSTVLSLILDGNDTTAVHLIWVMSLLLNNPHVMKQGQEEIDMKVGKERWIED

TDIKNLVYLQAIVKETLRLYPPVPFLLPHEAVQDCKVTGYHIPKGTRLYINAWKVHRDSE

IWSEPEKFMPNRFLTSKANIDARGQNFEFIPFGSGRRSCPGLGFATLVTHLTFGRLLQGF

DFSKPSNTPIDMTEGVGVTLPKVNQVEVLITPRLPSKLYLF.

>CYP82Q1 Stevia rebaudiana (Stevia, Asterales)

MEFHLSLLTTVTAILVVTAFFLQISKRKKVDTTKNKLLPPKAKGAWPLIGHLPLLGKNRI

AHRVLGDLADKYGPIFTIKLGVYQVLVVSSADAVKDCFTTSDKAFASRPKSTAVEIMGYN

YAMFGLAPYGEYWRQVRKIAVLEILSQKRVDMLEWARVSEVRTSTNEVYDAWRVNKENEG

SDMVLVDMKQWFTNLILNVLVRIISGKRFPFKSVEGIRFQKMEKKLFELLGAFVVSDLIP

SMKRFDIGGYQKQMKMAAEEINDIMEGWLNNRKTQKESGEQKEGDQYFMDVLISVLKDAS

DADFPGYDHDTVIKATCMALLAAGSDTTSVTIIWALALLLNHPEKMKIAQDEIDKHVGRD

RLVEESDLKNLVYINAIIKETMRLYPAAPLSVPHEAMEDCVVGGYHIPKGTRLLPNFWKI

QHDPNIWPEPYEFKPERFLSTHKDVDVKGKHFELLPFGTGRRMCPAITFVLQILPLTLAN

LIQQFEIRKPSNDPIDMTESAGLTTKRATPLDVLIAPRLSLKMYPVDV

>CYP82R1 Coptis japonica (Japanese goldthread, Ranunculales)

MHNPTLKQWIRTSTLPAPIIVSFIVILLFYFFKKRSSNMIRTKKAPEVVGAWPVIGHLNL

LSVPKPAYIVLGELADQYGPAFSIQFGVHPILVVSSWELVKACFTTNDKFFSSRLVNKAI

KYMFYDQKTISFAPHGPYWRELRKMITLNLLSNERLKMLKHQRISEMDACLKKLYELSTK

RKDENAGVLVDMSKWFGDISFNVVTRIVAGKHIFGPKTERYMNVMEEARRLMDVMVFSDV

IPYLGWLDRLRGVDSEIKRTAKELDSALESWVDEHRQKRVSISAGIGGIVNITEEEEIDF

IDIMLSIIAKNKLLGDDPGTLIKAIVQEMYLAAWDNTTVTLTWALCLLLNNKQVLKRAQC

ELDAQVGKERQVEDSDINTLPYIQAIVKESMRLYPPGPIIERETTEDCDVGDFRIPAGTR

LWINLWKLQRDPNVWPNDPQEFQPERFLNGHADIDMKGQHFELIPFGSGRRMCPGVSFSL

QVMHLVLARIIHGFELKTPTDADIDMSTTLGMISWKATPLEVLLTPRFPPVFYM

>CYP82S1 Vitis vinifera

MDLPSHFLAIAGLILGLVLWYNHWRGKTLTHKSKGMSPPEPSGAWPFVGHLHLLHGKVPV

FRTLGAMADKVGPVFVIRLGMYRTLVVSNREAAKECFTTNDKIFASRPNSSAAKILGYNY

AAFAFAPHGPYWREMRKLSMLEILSTRRLGDLMHVQVSELHAGIKDLYILGKDNNWVNPK

KVVISEWFEHLTFNVVLRMVAGKRYFNNVVHGGEEARSAIAAIKKLLLLVGASVASDVIP

FLEWVDLQGHLSSMKLVAKEMDSLIESWVKEHTGRLNSEASSSQDFIDIMLTKLKDDSLF

GYSRETIIKATVLTMIVAGSDTTSLTSTWLLSALLNNKHVMKHAQEELDLKVGRDRWVEQ

SDIQNLVYIKAIVKETLRLYTTFPLLVPHEAMEDCHVGGYHISKGTRLLVNAWKLHRDPA

VWSNPEEFQPERFLTSHANVDVFGQHFELIPFGSGRRSCPGLNMGLQMLHLTIARLLQGF

DMTKPSNSPVDMTEGISVALSKLTPLEVMLTPRLPAELY

>CYP83A1 Arabidopsis thaliana

MEDIIIGVVALAAVLLFFLYQKPKTKRYKLPPGPSPLPVIGNLLQLQKLNPQRFFAGWAK

KYGPILSYRIGSRTMVVISSAELAKELLKTQDVNFADRPPHRGHEFISYGRRDMALNHYT

PYYREIRKMGMNHLFSPTRVATFKHVREEEARRMMDKINKAADKSEVVDISELMLTFTNS

VVCRQAFGKKYNEDGEEMKRFIKILYGTQSVLGKIFFSDFFPYCGFLDDLSGLTAYMKEC

FERQDTYIQEVVNETLDPKRVKPETESMIDLLMGIYTEQPFASEFTVDNVKAVILDIVVA

GTDTAAAAVVWGMTYLMKYPQVLKKAQAEVREYMKEKGSTFVTEDDVKNLPYFRALVKET

LRIEPVIPLLIPRACIQDTKIAGYDIPAGTTVNVNAWAVSRDEKEWGPNPDEFRPERFLE

KEVDFKGTDYEFIPFGSGRRMCPGMRLGAAMLEVPYANLLLSFNFKLPNGMKPDDINMDV

MTGLAMHKSQHLKLVPEKVNKYM*

>CYP83A2/CYP83B1 Arabidopsis thaliana

MDLLLIIAGLVAAAAFFFLRSTTKKSLRLPPGPKGLPIIGNLHQMEKFNPQHFLFRLSKL

YGPIFTMKIGGRRLAVISSAELAKELLKTQDLNFTARPLLKGQQTMSYQGRELGFGQYTA

YYREMRKMCMVNLFSPNRVASFRPVREEECQRMMDKIYKAADQSGTVDLSELLLSFTNCV

VCRQAFGKRYNEYGTEMKRFIDILYETQALLGTLFFSDLFPYFGFLDNLTGLSARLKKAF

KELDTYLQELLDETLDPNRPKQETESFIDLLMQIYKDQPFSIKFTHENVKAMILDIVVPG

TDTAAAVVVWAMTYLIKYPEAMKKAQDEVRSVIGDKGYVSEEDIPNLPYLKAVIKESLRL

EPVIPILLHRETIADAKIGGYDIPAKTIIQVNAWAVSRDTAAWGDNPNEFIPERFMNEHK

GVDFKGQDFELLPFGSGRRMCPAMHLGIAMVEIPFANLLYKFDWSLPKGIKPEDIKMDVM

TGLAMHKKEHLVLAPTKHI

>CYP83D1 Glycine max (soybean)

LVLLSLLSIVISIVLFITHTHKRNNTPRGPPGPPPLPLIGNLHQLHNSSPHLCLWQLAKL

HGPLMSFRLGAVQTVVVSSARIAEQILKTHDLNFASRPLFVGPRKLSYDGLDMGFAPYGP

YWREMKKLCIVHLFSAQRVRSFRPIRENEVAKMVRKLSEHEASGTVVNLTETLMSFTNSL

ICRIALGKSYGCEYEEVVVDEVLGNRRSRLQVLLNEAQALLSEFFFSDYFPPIGKWVDRV

TGILSRLDKTFKELDACYERSSYDHMDSAKSGKKDNDNKEVKDIIDILLQLLDDRSFTFD

LTLDHIKAVLMNIFIAGTDPSSATIVWAMNALLKNPNVMSKVQGEVRNLFGDKDFINEDD

VESLPYLKAVVKETLRLFPPSPLLLPRVTMETCNIEGYEIQAKTIVHVNAWAIARDPENW

EEPEKFFPERFLESSMELKGNDEFKVIPFGSGRRMCPAKHMGIMNVELSLANLIHTFDWE

VAKGFDKEEMLDTQMKPGITMHKKSDLYLVAKKPTT

>CYP83E1 Medicago sativa (alfalfa)

MLPMLLLLVLCLTLPLLMFFHKHKTNTNNPPGPKGLPIIGNLLQLDISNLHLQFSQFSKI

YGPLFSLQLGLRPAIVVSSAEIAKEVFKNNDHVFSNRPISYGQNILSYNGSEIVFAPYGD

FWREIRKICAIHIFSSKRVSYYSSIRIFEVKKMIKNISVHADSSNVTNLSELLISLSSTI

ICRTAFGKSYEDDGIEKSRFHGLLHEFQALLAASFFADYIPFTGWIDKLRGLHGRVDRNF

KEFDEFYQEIIDEHLDPNREQITDEEDIVDVLLELKKKRSFSFDINFDHIKGILTDMLVA

ATDTTSAASVWAMTALIKNPRVMSKVKGEIRNLGVKKDFLYEDDIQNCPYLKAVVKETLR

LHLPAPLLVPRESIENCTINGYNIPAKTILYVNAWAIQRDPDIWINPEEFYPERFLESSI

NFIGQDFELIPFGAGRRICPSIPMAVASLELILANLLYSFDWKLPHGLVKEDIDTSMLPG

ITQHKKNPLCLIAKVPK*

>CYP83F1v1 Populus trichocarpus (cottonwood)

MALLIFVILFLSIIFLFLLKKNKISKRACFPPGPNGLPLIGNLHQLDSSNLQTQLWKLSQ

KYGPLMSLKLGFKRTLVISSAKMAEEVLKTHDLEFCSRPLLTGQQKFSYNGLDLAFSPYG

AYWREMKKICVVHLLNSTRVQSFRTNREDEVSHMIEKISKAALASKPFNLTEGMLSLTST

AICRTAFGKRYEDGGIEGSRFLALLNETEALFTMFFLSDYFPYMGWVDRLTGRAHRLEKN

FREFDVFYQQIIDEHLDPERPKPDHEDILDVLLQIYKDRTFKVQLTLDHIKAILMNIFVG

GTDTAAATVIWAMSLLMKNPEAMRKAQEEVRKVIGDKGFVYEDDVQQLPYLKAVVKETMR

LQPTAPLLVPRETTTECNIGGYEIPAKTLVYVNAWAIGRDTEVWENPYVFIPDRFLGSSI

DLKGQDFELIPFGAGRRICPGIYMGIATVELSLSNLLYKFDWEMPGGMKREDIDVDHTQP

GLAMHTRDALCLVPKAYAVMGNDA*

>CYP83G1v1 Medicago truncatula (barrel medic, Fabales)

MNKNMSPLILLPFALLLFFLFKKHKTSKKSTTLPPGPKGLPFIGNLHQLDSSVLGLNFYE

LSKKYGPIISLKLGSKQTVVVSSAKMAKEVMKTHDIEFCNRPALISHMKISYNGLDQIFA

PYREYWRHTKKLSFIHFLSVKRVSMFYSVRKDEVTRMIKKISENASSNKVMNMQDLLTCL

TSTLVCKTAFGRRYEGEGIERSMFQGLHKEVQDLLISFFYADYLPFVGGIVDKLTGKTSR

LEKTFKVSDELYQSIVDEHLDPERKKLPPHEDDVIDALIELKNDPYCSMDLTAEHIKPLI

MNMSFAVTETIAAAVVWAMTALMKNPRAMQKVQEEIRKVCAGKGFIEEEDVEKLPYFKAV

IKESMRLYPILPILLPRETMTNCNIAGYDIPDKTLVYVNALAIHRDPEVWKDPEEFYPER

FIGSDIDLKGQDFELIPFGSGRRICPGLNMAIATIDLVLSNLLYSFDWEMPEGAKREDID

THGQAGLIQHKKNPLCLVAKKRIECV

>CYP84A1 Arabidopsis thaliana

MESSISQTLSKLSDPTTSLVIVVSLFIFISFITRRRRPPYPPGPRGWPIIGNMLMMDQLT

HRGLANLAKKYGGLCHLRMGFLHMYAVSSPEVARQVLQVQDSVFSNRPATIAISYLTYDR

ADMAFAHYGPFWRQMRKVCVMKVFSRKRAESWASVRDEVDKMVRSVSCNVGKPINVGEQI

FALTRNITYRAAFGSACEKGQDEFIRILQEFSKLFGAFNVADFIPYFGWIDPQGINKRLV

KARNDLDGFIDDIIDEHMKKKENQNAVDDGDVVDTDMVDDLLAFYSEEAKLVSETADLQN

SIKLTRDNIKAIIMDVMFGGTETVASAIEWALTELLRSPEDLKRVQQELAEVVGLDRRVE

ESDIEKLTYLKCTLKETLRMHPPIPLLLHETAEDTSIDGFFIPKKSRVMINAFAIGRDPT

SWTDPDTFRPSRFLEPGVPDFKGSNFEFIPFGSGRRSCPGMQLGLYALDLAVAHILHCFT

WKLPDGMKPSELDMNDVFGLTAPKATRLFAVPTTRLICAL*

>CYP85A1 Arabidopsis thaliana

MGAMMVMMGLLLIIVSLCSALLRWNQMRYTKNGLPPGTMGWPIFGETTEFLKQGPNFMRN

QRLRYGSFFKSHLLGCPTLISMDSEVNRYILKNESKGLVPGYPQSMLDILGTCNMAAVHG

SSHRLMRGSLLSLISSTMMRDHILPKVDHFMRSYLDQWNELEVIDIQDKTKHMAFLSSLT

QIAGNLRKPFVEEFKTAFFKLVVGTLSVPIDLPGTNYRCGIQARNNIDRLLRELMQERRD

SGETFTDMLGYLMKKEGNRYPLTDEEIRDQVVTILYSGYETVSTTSMMALKYLHDHPKAL

QELRAEHLAFRERKRQDEPLGLEDVKSMKFTRAVIYETSRLATIVNGVLRKTTRDLEING

YLIPKGWRIYVYTREINYDANLYEDPLIFNPWRWMKKSLESQNSCFVFGGGTRLCPGKEL

GIVEISSFLHYFVTRYRWEEIGGDELMVFPRVFAPKGFHLRISPY

>CYP86A1 Arabidopsis thaliana

MEALNSILTGYAVAALSVYALWFYFLSRRLTGPKVLPFVGSLPYLIANRSRIHDWIADNL

RATGGTYQTCTMVIPFVAKAQGFYTVTCHPKNVEHILKTRFDNYPKGPMWRAAFHDLLGQ

GIFNSDGDTWLMQRKTAALEFTTRTLRQAMARWVNGTIKNRLWLILDRAVQNNKPVDLQD

LFLRLTFDNICGLTFGKDPETLSLDLPDNPFSVAFDTATEATLKRLLYTGFLWRIQKAMG

IGSEDKLKKSLEVVETYMNDAIDARKNSPSDDLLSRFLKKRDVNGNVLPTDVLQRIALNF

VLAGRDTSSVALSWFFWLVMNNREVETKIVNELSMVLKETRGNDQEKWTEEPLEFDEADR

LVYLKAALAETLRLYPSVPQDFKYVVDDDVLPDGTFVPRGSTVTYSIYSIGRMKTIWGED

CLEFRPERWLTADGERFETPKDGYKFVAFNAGPRTCLGKDLAYNQMKSVASAVLLRYRVF

PVPGHRVEQKMSLTLFMKNGLRVYLQPRGEVLA

>CYP86B1 Arabidopsis thaliana

MNFNSSYNLTFNDVFFSSSSSSDPLVSRRLFLLRDVQILELLIAIFVFVAIHALRQKKYQ

GLPVWPFLGMLPSLAFGLRGNIYEWLSDVLCLQNGTFQFRGPWFSSLNSTITCDPRNVEH

LLKNRFSVFPKGSYFRDNLRDLLGDGIFNADDETWQRQRKTASIEFHSAKFRQLTTQSLF

ELVHKRLLPVLETSVKSSSPIDLQDVLLRLTFDNVCMIAFGVDPGCLGPDQPVIPFAKAF

EDATEAAVVRFVMPTCVWKFMRYLDIGTEKKLKESIKGVDDFADEVIRTRKKELSLEGET

TKRSDLLTVFMGLRDEKGESFSDKFLRDICVNFILAGRDTSSVALSWFFWLLEKNPEVEE

KIMVEMCKILRQRDDHGNAEKSDYEPVFGPEEIKKMDYLQAALSEALRLYPSVPVDHKEV

VQEDDVFPDGTMLKKGDKVIYAIYAMGRMEAIWGKDCLEFRPERWLRDGRFMSESAYKFT

AFNGGPRLCLGKDFAYYQMKSTAAAIVYRYKVKVVNGHKVEPKLALTMYMKHGLMVNLIN

RSVSEIDQYYAKSFDEGYIN*

>CYP86C1 Arabidopsis thaliana

MNVLISAVVWVYTHLRLSDVALALVGLFLLSYLREKLVSKGGPVMWPVLGIIPMLALNKH

DLFTWCTRCVVRSGGTFHYRGIWFGGAYGIMTADPANVEHILKTNFKNYPKGAFYRERFR

DLLEDGIFNADDELWKEERRVAKTEMHSSRFLEHTFTTMRDLVDQKLVPLMENLSTSKRV

FDLQDLLLRFTFDNICISAFGVYPGSLETGLPEIPFAKAFEDATEYTLARFLIPPFVWKP

MRFLGIGYERKLNNAVRIVHAFANKTVRERRNKMRKLGNLNDYADLLSRLMQREYEKEED

TTRGNYFSDKYFREFCTSFIIAGRDTTSVALVWFFWLVQKHPEVEKRILREIREIKRKLT

TQETEDQFEAEDFREMVYLQAALTESLRLYPSVPMEMKQALEDDVLPDGTRVKKGARIHY

SVYSMGRIESIWGKDWEEFKPERWIKEGRIVSEDQFKYVVFNGGPRLCVGKKFAYTQMKM

VAAAILMRYSVKVVQGQEIVPKLTTTLYMKNGMNVMLQPRDW*

>CYP86E2 Zea mays (maize)

MTTRALVAMALRFLREYVRASDLAVAAAVLFACSAARSRLSSRPGEPMLWPVVGIIPTLF

AHLAAGDVYDWGAAVLLGRSRGTFAYRGTWGGGSSGVVTSVPANVEHVLKANFDNYPKGP

YYRERFAELLGGGIFNADGDSWRAQRKAASAEMHSARFVQFSAGTVERLVRRELLPLLES

LSGREGPESAAAAVDLQDVLLRFAFDNICAAAFGVEAGCLADGLRDVPFARAFERATELS

LTRFYTPPFVWKSKRLLGVGSERALVESARAVREFAERTVADRRAELRKVGDLAGRCDLL

SRLMSSPPAAGYSDEFLRDFCISFILAGRDTSSVALTWFFWLLASHPHVEARVLDDVARG

GGDVSAMDYLHAALTESMRLYPPVPVDFKEALEDDVLPDGTLVRARQRVIYYTYAMGRDK

ATWGPDCLEFRPERWLSKSGAFAGGAESPYKYVVFNAGPRLCVGKRFAYMQMKTAAAAVL

ARFRVEVLPGQEVKPKLNTTLYMKNGLMVRFVRREQRHELGHPLPAAAADAGED*

>CYP86F1 Physcomitrella patens (moss)

MQYLVMSRGDNCTHFHSQNQGQTGLGMCTGPNRMDSWMLTQVMLAGVVTFLVWHVIKYSR

IKGPIVWPVFGTTPQFLWNLPRMHDWTTDMLVKHDGTYTSIAPKCTCLTAVATCRPENLE

YVLKTNFANYPKGRSFTYPSHDLLGQGIFNTDHDLWKMQRKTASLEFSTRTLRDLMVKAN

RSSVGQRLLPVLADVARNRAPIDFQDLFLRYTFDNICMVGFGVDPGCLAPGLPTVPFAQA

FDLATEGTLTRMVVPEIFWRITRALGWGMEGRLAKAISTIDKFAADVITERRRELNMLKT

LNATEYPCDLLSRFMQTTDHEGNPYTDRFLRDVTTNFILAGRDTTAIALSWFFYLITQNP

AVEEKILNEIREILQSRRQSGGVGEPDDDDAGRTTQEASLSFEELKQLHYLHAALSESMR

LYPSVPIDNKDVTADDFLPDGTFVRKGTRLMYSIYSMGRMESIWGKDCLEYKPERWLRNG

VFTPESPFKYAVFNAGPRLCLGKELAYLQMKSVASAILRNYHVKLVPEHKVEYKLSLTLF

MKYGLHVTLHPRVTVAY*

>CYP87A2 Arabidopsis thaliana

MWALLIWVSLLLISITHWVYSWRNPKCRGKLPPGSMGFPLLGESIQFFKPNKTSDIPPFI

KERVKRYGPIFKTNLVGRPVIVSTDADLSYFVFNQEGRCFQSWYPDTFTHIFGKKNVGSL

HGFMYKYLKNMVLTLFGHDGLKKMLPQVEMTANKRLELWSNQYSVELKDATASMIFDLTA

KKLISHDPDKSSENLRANFVAFIQGLISFPFDIPGTAYHKCLQGRAKAMKMLRNMLQERR

ENPRKNPSDFFDYVIEEIQKEGTILTEEIALDLMFVLLFASFETTSLALTLAIKFLSDDP

EVLKRLTEEHETILRNREDADSGLTWEEYKSMTYTFQFINGTARLANIVPAIFRKALRDI

KFKDYTIPAGWAVMVCPPAVHLNPEMYKDPLVFNPSRWEGSKVTNASKHFMAFGGGMRFC

VGTDFTKLQMAAFLHSLVTKYRWEEIKGGNITRTPGLQFPNGYHVKLHKKRD*

>CYP87B6 Vitis vinifera

MLPIGLCVVSLVIIWITYWIRRWKNPRCNVTLPPGSLGFPLIGESIQFLISCSNSLDLHP

FFRKRIQKRYGPLFKTSMLGRQVVVTADPEANHFILEQEGKSVEMCYLDSVAQLCGHDES

SAGATGHIHKYLRTLILNHFGYERLRYKLLKKVEAMAHKSLGAWSSQPSVELNRATSQIM

LDFISKELFSYDPKGCTESMGDAFIDFLDSLASVPLNIPGTTFHKCLKNQKKTMKILREI

VDERCASPEIRRGDFLDYFLEGMKKEAFITKDFIAFVMFGLLFASFESIPIMLSLALKLI

MEHPLVLQELEEHEAILRNKDTSNFTLTWEDYKSMTFTVIDETLRMANVGLGNFRKALED

IKIKGHTIPAGWTILVVSSVLHMDPNIYPDPLVFNPWRWKGSXKITTKNFTPFGGGIRFC

PGAELSKLTMAIFLHVAVTKYRFTKIKGGNLVRNPVLKFKDGFHIKVSKK

>CYP87C6 Sorghum bicolor

MSMHYLAALSVTLLGAILLRWAFKWMNYGRTGGEEGMLLPPGSRGLPFLGETLEFFAASP

TLELVPFFKRRLERFGPIFRTNIVGEDMIVSLDPELNARVLQQEERGFQIWYPSSFMRIL

GADNMVSMLGPLHRHIRNLVLRLFGPEALRLVLLRDVQRSARDELRSWLDRPEVEVRTAT

SRMIFGVTAKKLISHDDVASGGSLWKCFDAWTKGLMSFPICVPGTAFYRCMQGRKNVMKV

LKQQLDERRNGAERKTVDFFDLVIDELDKPNSIMSESIALNLLFLLLFASHETTSMGLTV

ILKFLTDNPKSLQELTEEHEKIMERRVDPDSDITWEEYKSMKFTSHVIHESLRLANIAPV

VFRQANQDVHIKGYTIPEGSKIMICPSAAHLNSKVYEDPLAFNPWRWKDTPEPVGGSKDF

MAFGGGLRLCVGAEFAKLQMAMFLHYLVTNYRWKALSKGTMMLYPGLRFPDGFHIQLHKK

T

>CYP87D1 Populus trichocarpus (cottonwood)

MWAIGLVVVALVVIYYTHMIFKWRSPKIEGVLPPGSMGWPLIGETLQFISPGKSLDLHPF

VKKRMQKYGPIFKTSLVGRPIIVSTDYEMNKYILQHEGTLVELWYLDSFAKFFNLEGETR

VNAIGAVHKYLRSITVNHFGVESLKESLLPKIEDMLHTTLAKWASQGPVDVKQVISVMVF

NFTANKIFGYDAENSKETLSKNYTKILNSFISLPLNIPGTSFHKCMQEREKMLKLLKDTL

MERLNGPSKRRGDFLDQAIDDMKTKKFLTVDFIPQLMFGILFASFESMSTTLTLTFKFLT

ENPRVVEELRAEHEAIVKKRENPNSRLTWEEYRSMTFTQMVVNETLRISNIPPGLFRKAL

KDFQVKGYTVPAGWTVMLVTPAIQLNPDTFKDPVTFNPWRWKDLDQVTISKNFMPFGGGT

RQCAGAEYSKLVLSTFLHVLVTSYSFTKVKGGDVSRTPIISFGDGIHIKFTARN*

>CYP88A3 Arabidopsis thaliana

MAETTSWIPVWFPLMVLGCFGLNWLVRKVNVWLYESSLGENRHYLPPGDLGWPFIGNMLS

FLRAFKTSDPDSFTRTLIKRYGPKGIYKAHMFGNPSIIVTTSDTCRRVLTDDDAFKPGWP

TSTMELIGRKSFVGISFEEHKRLRRLTAAPVNGHEALSTYIPYIEENVITVLDKWTKMGE

FEFLTHLRKLTFRIIMYIFLSSESENVMDALEREYTALNYGVRAMAVNIPGFAYHRALKA

RKTLVAAFQSIVTERRNQRKQNILSNKKDMLDNLLNVKDEDGKTLDDEEIIDVLLMYLNA

GHESSGHTIMWATVFLQEHPEVLQRAKAEQEMILKSRPEGQKGLSLKETRKMEFLSQVVD

ETLRVITFSLTAFREAKTDVEMNGYLIPKGWKVLTWFRDVHIDPEVFPDPRKFDPARWDN

GFVPKAGAFLPFGAGSHLCPGNDLAKLEISIFLHHFLLKYQVKRSNPECPVMYLPHTRPT

DNCLARISYQ*

>CYP88B1 tomato

MDFYNLALFFIALILGIFTFYAILMRINGWYYAIKFCSNKYNIPNGYMGLPYFGNTLSYF

KASMCGDPKSFIDFFATRFGEGGMYRAYIFGKPTIMVTKPEIIRKVLMDEEYLERGLPNY

MKKLIGLTTSIEEDKYFRRLTAPVKSHGLLSDYFDYIDKTVSSTLEKYATTEEPVEFLHK

MHKLTFEVFMRLLIGDEVNQELFDEMFEEITAVISGVHNLPINLPGFAYHKGLKARKVLX

EVFKKLIDERREAMKDGKSMPKANIIDMLLSNNNQDYEANMLSDKKIIEILVLFSFAGFE

PVALMSVKAIFHLQKHPHFLEKAKEEQEEIVKRRASSNAGLSFDEIRQMTFVSKIINETL

RIATDQSVFLRDTSTTFNINGYTIPKGWKFFAVVWNIHMNPDVYVQPKEFNPSRWDDIET

KPGIFLPFSMGPKSCPGSNLAKLQISVILHYYLLHYRVEQINPEARCYPPENCLVKFKKL

SISSNGN*

>CYP88C1 Petunia hybrida

MEYDSMFLYTALAVGILTIWSILKNGNGWFYTFKFSSNKCRLPPGDMGWPFFGNMLHFVK

CLSNYDLASFVSYFVTRFGKGGLYKAYMFGKPTILVTSPELCRKVVMDDENFDLGFPQYI

LELLRKEPIGGTTNQEDKLARRLTTPIKSHGLVSFFFDFLSENVKTSFEKWSASEKPIEL

LAEMKKPTFAVLMRVLLGGEELVARELLDVIFKENNFRFAGLRSLPINIPGFAFHRAMKG

RKEIIKVFERVINERKVLIAKDKTRAKSNILDIMLSTQDDDGKGLRDGNILKTLLWYTFS

GYESVAKVATQTMMLLQNHPECLKKAKEEQEEIVKRRTSPNEGLNFSEIGQMKYVTNVIN

ETLRLGSTETVLFRDARTDVNLNGYTIPEGWKCLALLGNFYKDPDTYVKPNEFIPSRWDD

LEVKPASFLPFGVGLRMCPGANLVRLEVAVVLHYFLLNYRLEMLDPDSTPEKCLARFKKL

SA

>CYP88D3 (Medicago truncatula)

MEMQWVYICTAALFACYVFVNKFLRRFNGWYYHLKLRNKEYPLPPGDMGWPLIGNLLSFN

KNFSSGQPDSFTTNLILKYGRDGIYKTHVYGNPSIIICDPEMCKRVLLDDVNFKIGYPKS

IQELTKCRPMIDVSNANHKHFRRLITAPMVGHKVLDMYLERLEDIAINSLEELSSMKHPI

ELLKEMKKVSFKSIIHVFMGTSNQNIVKNIGSSFTDLSKGMYSIPINAPGFTFHKALKAR

KKIAKSLQPVVDERRLIIKNGQHVGEKKDLMDILLEIKDENGRKLEDQDISDLLIGLLFA

GHESTATGIMWSVAHLTQHPHILQKAKEEQEEILKIRPASQKRLSLNEVKQMIYLSYVID

EMLRFANIAFSIFREATSDVNINGYLIPKGWRVLIWARAIHMDSEYYPNPKEFNPSRWKD

YNAKAGTFLPFGAGSRLCPGADLAKLEISIFLHYFLLNYRLERINPDCPVTTLPQCKPTD

NCLAKVIKVSRA

>CYP88D4 (Lotus japonicus)

MELYWAWVCAATLATCYVLRRLNGWYYDVKLRKKQYPLPPGDMGWPLIGNLIPFYKDFSS

GRPNSFINNLLLKYGEGGIYKTHLFGNPSIIVCEPEICMRVLTDDVNFRVGYPTTIKELI

RLKHISRAEHKQYRRLVNTLPILDHQALATLYLERIENIVTNSLEELSSMKHPVELLKEM

KKVTFKVFIHILMGSSIHHMIIENMDTSFAELTNGILSAPINAPGFVFHKALKARKKLAK

ILQSVVDERRLRSKNGQEGKDKVFLDNLLEAKDENGRKRDDEYIVDVLIAQLFAGHETSA

TALMWTILYLTQHPHILEKAKKEQEEIMKARVSSQGRLNLQEIKQMVYLSQVIDETLRCA

NIVFTTFREAISDVNINGYVIPNGWRVLVWARAVHMNPKYYPNPEEFNPSRWDDYHGKAG

TFLPFGAGSRLCPGKDLAKLEISVFLHYFLLNYKLERINAECPITFLPILKPVDNCLAKV

IKVS

>CYP88D5 Sequence3 (Lotus japonicus)

MELYWAWVSAATLATCYVFVDIFLRRLNGWYYDLKLCKKQHPLPPGDMGWPLIGNLISFY

KDFSSGHPNSFTNNLLLKYGQSGMYKTHLFGKPSIIVCEAEICRRVLTDDVNFKFAYPES

LRQLIPVQSISRAEHRQFRRLINTPIMNHQALAVYLERIENIMINSLEELSSMKHPVELL

KEMKKVTFKVIIDILMGTSIPHMITQNMESFFAELCNGMLSAPINAPGFVYHKALKARKK

LAKTVQSVVDERRLKSKNGQEGKDKAFIDSVLEVNDENGRKLEDGYIIDLLIAILFAGHE

TSATTMMWTIVYLTQHPHILNKAKEEQEKIMKVRVSSQTRLNLQEIKQMVYLSQVIDETL

RCANIVFSMFREATSDVNMSGYVIPKGWRVLIWGRAVHMDPENYPNPEEFNPSRWDDYHG

KAGTSLPFGVGSRLCPGKDLAKLEISIFLHYFLLNYKLERINPDCPITFLPIPKPVDNCL

AKVIKVSCN

>CYP88D6 (Glycyrrhiza uralensis)

MEVHWVCMSAATLLVCYIFGSKFVRNLNGWYYDVKLRRKEHPLPPGDMGWPLIGDLLSFI

KDFSSGHPDSFINNLVLKYGRSGIYKTHLFGNPSIIVCEPQMCRRVLTDDVNFKLGYPKS

IKELARCRPMIDVSNAEHRLFRRLITSPIVGHKALAMYLERLEEIVINSLEELSSMKHPV

ELLKEMKKVSFKAIVHVFMGSSNQDIIKKIGSSFTDLYNGMFSIPINVPGFTFHKALEAR

KKLAKIVQPVVDERRLMIENGPQEGSQRKDLIDILLEVKDENGRKLEDEDISDLLIGLLF

AGHESTATSLMWSITYLTQHPHILKKAKEEQEEITRTRFSSQKQLSLKEIKQMVYLSQVI

DETLRCANIAFATFREATADVNINGYIIPKGWRVLIWARAIHMDSEYYPNPEEFNPSRWD

DYNAKAGTFLPFGAGSRLCPGADLAKLEISIFLHYFLRNYRLERINPECHVTSLPVSKPT

DNCLAKVIKVSCA

>CYP88E1 Selaginella moellendorffii

MNLKWAIAIATVAAATFFELLRNFNRFWYEPKLKPGQAPLPPGSLGWPIFGNMASFLRAF

KSHNPDSFITKYLHKYDRTGVYKAFLFWQPTVLATTPETCKVVLSRDSLFETGWPSSTRR

LIGTRSFAGVTGEEHLKLRRLTEPALSNPKALEDYIPRMSSNIKSCLEEWSCQERTLLLR

EMRKYAFRTIHDILFSKDSGLDVEEVSSLYYEGNQGIRSLPINLPGTSYNRALKARKKLD

VLLHRVLNKRRFSEKPEKTDTLSLLMDATDENGKHLDDKQIVDLLVMYLNAGHDSTAHLI

LWLLIFLLKHEIVYDKVKEEQELIASQKPLGDSLSLSDVKKMSYLSRVINETLRVANISP

MVFRRAVTDVEVNGFTIPKGWYVEPWLRQVHMDPAVHSNPQNFDPDRWAVIRPFTHLPFG

LGSRTCPGNELAKLEACIIVHHLVLGYEVKPLNPDCEVTFLPHPRPKDYFPVQVRRRR*

>CYP88F1v1 Selaginella moellendorffii

MDLWLPSIAVALIIVLISCILNFNSWFYAPKLRPGSPPLPPGSLGWPVFGNMGDFLQAFK

SSNPESFVGGFISKYGCGGLYKAFLFRQPTILATSAEVCKTVLCNHDVFEIGWPERVVKE

LLGLKVLSAVTGDDHLKLSKLVKPALSSPKAIQHQMPCIEENVKKLLDEWADRGNIVFLD

EARMFTLKTIHEILVGEDTGIDFKQVSGLFHTMNKGLRALPLNFPGTAYSNAVKARATLA

NDFWRIFYERKESKKRGGDTLSMLLDATDEGGQPLEDDQIVDLIMSFMNGGHESTAHLVT

WLAILLKEHPAVYQRLKAEQDEIALKKMPGESLTLADMRSMTYMSRVIDETLRLINISPF

VFRKVLSDVQLNGYTIPRGWFVEAWLRQVHMDPLVHKNPREFDPDRWINEKPQPHTYVAF

GLGNRKCPGSNLSKIQSSIIIHHLITKYNWEPLNPHYKLVYLPHPRPADHYPVKITKRAL

V*

>CYP89A2 Arabidopsis thaliana

MEIWLLILASLSGSLLLHLLLRRRNSSSPPLPPDPNFLPFLGTLQWLREGLGGLESYLRS

VHHRLGPIVTLRITSRPAIFVADRSLTHEALVLNGAVYADRPPPAVISKIVDEHNISSGS

YGATWRLLRRNITSEILHPSRVRSYSHARHWVLEILFERFRNHGGEEPIVLIHHLHYAMF

ALLVLMCFGDKLDEKQIKEVEFIQRLQLLSLTKFNIFNIWPKFTKLILRKRWQEFLQIRR

QQRDVLLPLIRARRKIVEERKRSEQEDKKDYVQSYVDTLLDLELPEENRKLNEEDIMNLC

SEFLTAGTDTTATALQWIMANLVKYPEIQERLHEEIKSVVGEEAKEVEEEDVEKMPYLKA

VVLEGLRRHPPGHFLLPHSVTEDTVLGGYKVPKNGTINFMVAEIGRDPVEWEEPMAFKPE

RFMGEEEAVDLTGSRGIKMMPFGAGRRICPGIGLAMLHLEYYVANMVREFQWKEVQGHEV

DLTEKLEFTVVMKHPLKALAVPRRCH

>CYP89B17 Zea mays (maize)

METSWLLLSGALLLSLLVLRLHAKNRRLPPGPPAVPLFGNLLWLRNSAVQVEPLLLKLFK

RYGPVVTLRMGSQLTIFVADRRLAHAALVGAGAVTMANRPQAATSSLLGVSDNIITRTDY

GPVWRLLRRNLVAETLHPSRVRLFAPARAWVRGVLMDKLRAGGAAGDDEPRDVMEAFRYT

MFCLLVIMCFGERLDEPAVRAIQDAERKWLLYISQQMSVFFFFPSVTRHVFRGRLQTARA

LHRRQTELFVPLINARREYKRLAKDGQAPERETTFQHSYVDTLLDITLPDEEGHRPLTDD

EIVRLCSEFFTAGTDTTSTGLQWIMAELVKNPAVQDRLYAEIKATCGDGDAEAVSEEAVH

GMPYLKAVILEGLRKHPPGHFVLPHKAAEDMDVGGYLIPKDATVNFMVAVMGRDEQEWER

PMEFVPERFLEGGDGAEVDLTGIKGIRMMPFGVGRRICAGMSIAMLHLEYFVASMVREFE

WKEAPGH

>CYP89C3 Zea mays (maize)

MEDWLFYSLTTLLCLLCSLILRARTPGKKARNADSSSPLPPLPPGPTPLPVLGPLLFLAR

RDFDIEPVLRRIARDHGKVFTFAPLGRARPGIFVADRGAAHRALVQRGAAFASRPPSTAS

SAVLTSGGRNVSSSPYGATWRALRRNLASGVLNPARLRAFSPARRWVLGVLARRVRADGR

HGEAPVAVMEPFQYAMFCLLVHMCFGGDRLGDDARVRDIEATQRELLGSFLSFQVFSFLP

WVTKLVFRRRWEKLVSLRRRQEELFVPLIQARREAGGDGDSYVDSLVKLTIPEDGGRPLT

DGEIVSLCSEFLSAGTDTTATALQWILANLVKNPAMQDRLRDEVSSAGAGADGEVLEEEL

QAMPYLKAVVLEALRRHPPGHYVLPHAVHEDTTLDGYRVPAGAPVNFAVGDIGMDEEVWR

APAEFRPERFLPGGEGDDVDLTGSKEIKMMPFGAGRRVCPGMALALLHLEYFVANLVREF

DWRQADGEEVDLTEKLEFTVVMKRPLRARAVPLRPPPPAVAAA*

>CYP89D1 rice (japonica cultivar-group)

MEVILLPLVVIITSTMLLLLIISTAKKRHHGTANLPLPPAPPSVPVVGPLLWLVRARSNL

EPAIRELHRRHGPILSLTFLSPRAAIFVSSREVTHRALVQRGHTFASRPPAIAPFAVLTS

GQCTVSSAPYGPLWRSLRRNLTSGVLGHGSRAPLYAPARRWALHLLTSDLAAASGNTGGG

VAVAVVDCLQFAMFSLLTYMCFGKRLDRRGVREIEAVQRELFSSYISFQVFAFCPTVTKR

LFFRRWQKVLSIRRRQEDIFLPLIEERRKRIKISSMDNDGSMVCCYVDTIISHKLPKEAG

DRRLTDGELVSLCTEFLTASVDTIVTALQWIMARVVEQPEIQAKLLDEINRVVSSDKEHV

DEEDIKSMAYLKALVLEGLRRHPPAHFLLSHAAVEETSLDGHRIPAGRSVNFSVADVAHD

ENVWSRPEEFLPERFLDGGEGAGTDLTGSREIKMMPFGVGRRICPGLGLALLQLEYFVAN

MVREFEWGMVDGDCGGGINLAERPEFTVIMEQPLRALVVPRRRE

>CYP89E1 rice

MEETWLFLLFSISLVAVLLATARRRRSSSIKARLPPGPSPLLFLAKFLRLRRSIFDLGPL

LRDLHARHGPVISIRLFGTTLVFVADRRLAHRALVQGGSTFADRPPLPELGRLFTSDTRD

INSSPYGPYWRLVRRNLASEALSPARVALFAPARRRARDVLVRGLRDRGGDGSRPVELRP

LLRRAMFELLLYMSLGARLAPEALEEVERLELWMLRAFTSFPVFSFFPAITKRLFRNQWA

AHVAVRRRVGEIYVPLINARRAGDGDGDDPPCYTDSLLQLRVAEEGDRPLTDDEIIALCS

EFLNAGTDTTVTLVEWIMAELVNRPDIQAKVHDEVRRRPELTEADLQAMPYLKAVVLEGL

RLHPPAQFLLPHGVQSDAEVGGYVVPRGAELNVWVAELGRDEVVWTAAREFMPERFMDGG

EVEVDVTGSREITMMPFGVGRRMCPGYTVGTLHAEYLVGSLVRELEWLPETEGEAADMAE

ELDFTTVMKHPLRARVLPRPSSLY*48655

>CYP89F1 rice

MHAELTSTTTMEMGSLLPHAASLFAVSMASLMIAAVLSIVRRPWPWKTAAISREAVLRLL

GVRLGDVPTTVVRDGAVAVDALVRRADAFSDRPAGGGATSIVSGGRAHNINTVPHGPLWV

ALRRNLTSEAFHPVHGLARAAPGRSSRTSRPPRRAPPAEGQAVRDCLYAALFALNVATCF

GDGVDGELVGAMRAAQQEFLRFLPRARVFSTFQKAARLVYPDRWKQLLRHRRRQEEMYLP

LIRAINEQRRTRGTPSPPPPTTYVDTLLYLEVPADDGRRRRKLSDGEMVGLVSEYLGAAT

GTVVAQLEWALANLVRRPDIQTRLCGEVEAAAGGEPCAYLRAVVMECLRRHPPVSSVQRH

MVRDVMLGGAHVARGNVVSFAIEEIGRWTSSEEFSPERFMEGGEEGVRLAIGSKQEATTK

VKMMPFSAGRRTCPGMGYAILHLEYFLANLVTAFEWRRVPWEEEVDLTADYGFITTMQHP

LRALVVPLSNDRSTVV*

>CYP89G1 rice

MALLLLLVSSSCLVVAASIAVLCYVNNDADERLPPGPRVRLPLIGNLFLHAPTMAFLPSA

LRRLRRSHGPVVTLWAGNRPAVFVIGRDFAHRTLVLAGAALAHRPPSPFASSRALSFNRH

GVNAAEYGDRWRRLRSNICSCLAATEALRRRSVDRLVATLELEARAGAG1055ATGVVAP

TDAFRHGVFSFFAVLCFGEWVRDGEHDAVLRDLRRAHADILALTVELGAFHLVPAVLMVP

YLHRWWKLSGLQRSHRDIVAALISVRRLRREKADGDVADSATFCYVDTLLELELGEDEMV

SLCWEFMNAAAKTTSTALEWTMARLVHHSDIQRKLRHDIAKTTNSGGVGVSPSPYLKAVV

QESLRLHPPAHYLLAHTVDRDVPLGAGGYVIPKGAIVNYAVAEIGRDATAWTDPDEFVPE

RFMEGGEGAMVDAVSCGGAEIRMMPFGAGRRACPGASFAVSVLHLFVGRLVEQFEWWPVA

EDEKAAAVDFSEKTGLVTVMKTPLRALLVPITSS

>CYP89H1 Zea mays

MDDLVTMFPHLSHSRSVTLLFLFLTTAFLLVGCSRKSGAVMLAVLRWLAAPVLTLPWHRA

SGGRGTRRGLSVQVTDRAVARRALVQHSAAFLDRPTGAVPSTILTRNRHYNILSSPYGPY

WRAARRNVATGVLHPSQLRMLGGTRARVLGDLVRALKSGAPAGESLYFAVYSVLAGMCFG

EDVVAELGETRLRAMQKFQRDILLALPSFGVFVRYPRIGRFLYRSRWHRLLALRRQQEES

FLPLVAAIRNRREASRGNTTLTTYVESLLDLRIHEDGGRAVTDGELVSLISEFLGAGTES

TAAALEWTMANLVKSPDLQQKLRLEANAMACGKRVIEEEDLARMPYLRAVVLESLRRHPP

VPFVIRRVDGDDAKKVIGVSRLPDGGATVNFLVGKIGRDPAAWSDPMSFKPERFMPGGEG

DGTDLTCTTELKMMPFGAGRRVCPGLATAMLHLKYFVANLLTEFEWWEAEDDKVDLTEFR

GFFFTVMNRPLQARLVPTDAAAAPWLSN*

>CYP89J1 Triticum monococcum

MQHVLVILTVTLVLLVVVVRRYAPSKAVYTRLAASIKSTMARRFRPPAIVIKDRATAHRL

LVRGCAGGNFCNRPASLTPTAVVSQLRHHNIITAPYDPFWRVTRRNLTSEVLHPLRLHQY

AAARREALRVLVADLRAQCTSNPDGLVLAAESIRNAMFGLLATMCFGDGIDKGLVRAMAD

AQYEFMQLFPDLRLFARVPALARLIHRKRWSKIIALRRKQEDMYLPLIHARRTRQRQSGE

TPAYVDTLIDLWVPDEHNAGKRRRQRRLANGELVGLCSEFVGAGTETVAAELQWIMANLV

KHPHLQEAVRRETDAAVDANAEEVGEEVLPKLEYLNAVVMEALRLYPTVTLVIRQVMEED

DVVHDSRRIPAGTNVIFRPLSLGRDKTAWANPDEFRPERFLACRGGQSVNLVAAAGSRGG

EMSMMPFGAGRRVCPGMGVAMLHTAYFLANLVKEFEWRDAEGELAVDLRPRFAFFTVMER

PLRARLLLRSRTQNGQVN*

>CYP90A1 Arabidopsis thaliana

MAFTAFLLLLSSIAAGFLLLLRRTRYRRMGLPPGSLGLPLIGETFQLIGAYKTENPEPFI

DERVARYGSVFMTHLFGEPTIFSADPETNRFVLQNEGKLFECSYPASICNLLGKHSLLLM

KGSLHKRMHSLTMSFANSSIIKDHLMLDIDRLVRFNLDSWSSRVLLMEEAKKITFELTVK

QLMSFDPGEWSESLRKEYLLVIEGFFSLPLPLFSTTYRKAIQARRKVAEALTVVVMKRRE

EEEEGAERKKDMLAALLAADDGFSDEEIVDFLVALLVAGYETTSTIMTLAVKFLTETPLA

LAQLKEEHEKIRAMKSDSYSLEWSDYKSMPFTQCVVNETLRVANIIGGVFRRAMTDVEIK

GYKIPKGWKVFSSFRAVHLDPNHFKDARTFNPWRWQSNSVTTGPSNVFTPFGGGPRLCPG

YELARVALSVFLHRLVTGFSWVPAEQDKLVFFPTTRTQKRYPIFVKRRDFAT*

>CYP90B1 Arabidopsis thaliana

MFETEHHTLLPLLLLPSLLSLLLFLILLKRRNRKTRFNLPPGKSGWPFLGETIGYLKPYT

ATTLGDFMQQHVSKYGKIYRSNLFGEPTIVSADAGLNRFILQNEGRLFECSYPRSIGGIL

GKWSMLVLVGDMHRDMRSISLNFLSHARLRTILLKDVERHTLFVLDSWQQNSIFSAQDEA

KKFTFNLMAKHIMSMDPGEEETEQLKKEYVTFMKGVVSAPLNLPGTAYHKALQSRATILK

FIERKMEERKLDIKEEDQEEEEVKTEDEAEMSKSDHVRKQRTDDDLLGWVLKHSNLSTEQ

ILDLILSLLFAGHETSSVAIALAIFFLQACPKAVEELREEHLEIARAKKELGESELNWDD

YKKMDFTQCVINETLRLGNVVRFLHRKALKDVRYKGYDIPSGWKVLPVISAVHLDNSRYD

QPNLFNPWRWQQQNNGASSSGSGSFSTWGNNYMPFGGGPRLCAGSELAKLEMAVFIHHLV

LKFNWELAEDDQPFAFPFVDFPNGLPIRVSRIL

>CYP90C1 Arabidopsis thaliana

MQPPASAGLFRSPENLPWPYNYMDYLVAGFLVLTAGILLRPWLWFRLRNSKTKDGDEEED

NEEKKKGMIPNGSLGWPVIGETLNFIACGYSSRPVTFMDKRKSLYGKVFKTNIIGTPIII

STDAEVNKVVLQNHGNTFVPAYPKSITELLGENSILSINGPHQKRLHTLIGAFLRSPHLK

DRITRDIEASVVLTLASWAQLPLVHVQDEIKKMTFEILVKVLMSTSPGEDMNILKLEFEE

FIKGLICIPIKFPGTRLYKSLKAKERLIKMVKKVVEERQVAMTTTSPANDVVDVLLRDGG

DSEKQSQPSDFVSGKIVEMMIPGEETMPTAMTLAVKFLSDNPVALAKLVEENMEMKRRKL

ELGEEYKWTDYMSLSFTQNVINETLRMANIINGVWRKALKDVEIKGYLIPKGWCVLASFI

SVHMDEDIYDNPYQFDPWRWDRINGSANSSICFTPFGGGQRLCPGLELSKLEISIFLHHL

VTRYSWTAEEDEIVSFPTVKMKRRLPIRVATVDDSASPISLEDH

>CYP90D1 Arabidopsis thaliana

MDTSSSLLFFSFFFFIIIVIFNKINGLRSSPASKKKLNDHHVTSQSHGPKFPHGSLGWPV

IGETIEFVSSAYSDRPESFMDKRRLMYGRVFKSHIFGTATIVSTDAEVNRAVLQSDSTAF

VPFYPKTVRELMGKSSILLINGSLHRRFHGLVGSFLKSPLLKAQIVRDMHKFLSESMDLW

SEDQPVLLQDVSKTVAFKVLAKALISVEKGEDLEELKREFENFISGLMSLPINFPGTQLH

RSLQAKKNMVKQVERIIEGKIRKTKNKEEDDVIAKDVVDVLLKDSSEHLTHNLIANNMID

MMIPGHDSVPVLITLAVKFLSDSPAALNLLTVEENMKLKSLKELTGEPLYWNDYLSLPFT

QKVITETLRMGNVIIGVMRKAMKDVEIKGYVIPKGWCFLAYLRSVHLDKLYYESPYKFNP

WRWQERDMNTSSFSPFGGGQRLCPGLDLARLETSVFLHHLVTRFWIAEEDTIINFPTVHM

KNKLPIWIKRI*

>CYP90E1v2 Selaginella moellendorffii

MISSSTAWAWTSLAGVAGVFWLAALVYWRSWRFRKLQRLPPGSMGWPLIGELVPYVTIVR

SETPFRFTRERESKYGPVFKTSLLTGKTVMITDVEGVKFVLHNEGVLFETGYPRSLKDVL

GEHAMLFQHGDLQKRMHAMLKRFVSSTPLKKHLTREMELLTMQGMSTWSRGQRILLQDEI

QRITHDFLMKQLFGLEPGKLSTTILKEFHTLMAGIIGIPMMIPGTPYFKAMKAREKLSKI

IMDMVATRRAKPDIEHKDILNALIEEVKQEDGDMEKIIIDNVLVNIANAENVPAVVIALA

VKNLSETPKALEQIREENLAIRKGKDPSEGLSWNEYMSLEFTQAVFNETLRLANGAQGVM

RKALKDVEYRGYIIPKGWTVLPYFLNIHFDENMFPNSAKFHPWRWLEKNIPPSYVLPFGG

GSRLCPGQELAKVQTAVFLHHLVTQFKWDAEPEKVINFPMISTRNHVPVVLYDLN*

>CYP90F1v1 Selaginella moellendorffii

MNLPPGRMGWPLVGETLEYLATRPIGVPQPFIAKRVARYGSIFKTHLFGCPTIVTTDPDF

NRFVLANEGKLFQSSYPAGVDRVLGKFSMVQASGELHKRMRALTVSFMQAQSLKDNFLQT

IQARVISLLSTWEGRVVKIQDEAQSLSFDCIVGHVLGMDPGAENTKTIKEDFFNLVYGLT

IPLRIPGTRYWTAMKGRQNIVRLVEQMVAERTTKPCTARKDFLQQLLQDDNGKNLTLEQI

SDFIVFMLFAAHDTTATAMTMAIKYLLANPQALNQLQEEHLEIRRNKRSPDEPLEWNDYL

QMTFTQHVINETLRLTNVLTSAHRIALQDVQTEEGYVIPKGWKVVSSWTTIHLNPKLYAE

PLEFNPWRWKTQSVKYFTPFSGGPRFCTGSELARLEIALLLHFIITKYSLHPAEDDEAVY

FGTVKMRKGLPVTVTKLSQIL*

>CYP92A1 Zea mays

MEPATWAVFLGIALCAAAALFLSRGRRPVYNPPPGPKPWPIIGNLNLMGELPHRSMNELS

KRYGPLMQLRFGSLPVLVGASVEMAKLFLKTNDAAFSDRPRFAIGKYTAYDFSDLLWAPS

GPYLRQARRICATELFSATRLESFEHIRDEEVRVMLRQLRQAAGRTVRLRDYLQMLALGV

ISRIVLGNKYVMEEVADGEGDSAPAITPAEFREMVDEFFALHGAFNIGDYIPWLDWLDLQ

GYVARMKRMKARFGRFLERVLDVHNERRLREGGNFVAKDMLDVLLQLADDTSLEVQLSRD

NVKAITQDLIIAGTDSNANTLEWAVSELLKNPKILAKAMEELNHVIGPDRLVTESDLPRL

PYIEAVLKETMRVHPAAPMLAPHVAREDTSVDGYDVLAGTVLFINVWAIGRDPGLWDAPE

EFRPERFVESKIDVRGHDFQLLPFGSGRRMCPGINLALKVMALSLANLLHGFEWRLPDGV

TAEELSMDEAFKLAVPRKFPLMVVAEPRLPARLYTGA

>CYP92A15 Oryza sativa (rice)

MEVQELVPSPWSSSSSFLVLVLATLLFVAAFLRRRQGARRKYNIPPGPRPWPVIGNLNLI

GALPYRSIRDLSRRYGPLMSLRFGSFPVVVGSSVDMARYFLRANDLAFLDRPRTAAGRYT

VYNYAGVLWSHYGEYWRQARRLWVTELLSARRLASTEHVRAEEVRAMLRGLSRRAGAGTA

VVLKEHMLMVTLNVISRMVFGKKYIVEEGEGSSPTTAEEFRWMIEEIFFLNGVFNIGDMV

PWLGWLDPQGYIGRMKRLGGMFDRFLEHILDEHVERRRREGDGFAARDMVDLLLQFADDP

SLKVPIQRDGVKAFILELITGSTDTTSVSVEWAMSEVLRNPSVLARATDELDRVVGRRRL

VAEGDIPNLPYLDAVVKESMRLHPVVPLLVPRVSREDAFSVSVAGAAASYDIPAGTRVLV

NVWAIGRDPAVWGDDAEEFRPERFAAGGERGGVDVKGQDFELLPFGSGRRMCPGFGLGLK

MVQLTLANLLHGFAWRLPGGAAAEELSMEEKFGISVSRLVQLKAIPEPKLPAHLYDE*

>CYP92B3 tobacco

MENSWVVLALTGLLTLVFLSKFLHSPRRKQNLPPGPKPWPIVGNIHLLGSTPHRSLHELA

KRYGDLMLLKFGSRNVLILSSPDMAREFLKTNDAIWASRPELAAGKYTAYNYCDMTWARY

GPFWRQARRIYLNEIFNPKRLDSFEYIRIEERHNLISRLFVLSGKPILLRDHLTRYTLTS

ISRTVLSGKYFSESPGQNSMITLKQLQDMLDKWFLLNGVINIGDWIPWLAFLDLQGYVKQ

MKELHRNFDKFHNFVLDDHKANRGEKNFVPRDMVDVLLQQAEDPNLEVKLTNDCVKGLMQ

DLLAGGTDTSATTVEWAFYELLRQPKIMKKAQQELDLVISQDRWVQEKDYTQLPYIESII

KETLRLHPVSTMLPPRIALEDCHVAGYDIPKGTILIVNTWSIGRNSQHWESPEEFLPERF

EGKNIGVTGQHFALLPFGAGRRKCPGYSLGIRIIRATLANLLHGFNWRLPNGMSPEDISM

EEIYGLITHPKVALDVMMEPRLPNHLYK

>CYP92C5 Zea mays (maize) EU955931

MELASTMSVAMALAAAIFVVLCSVVASARGRREKALKLPPGPRGWPVLGSLGALAGALPP

HRALAALAARHGPLMHLRLGSYHTVVASSADAARLVLRTHDSALADRPDTAAGEITSYGY

LGIVHTPRGAYWRMARRLCATELFSARRVESFQDVRAQEMRALARGLFGCAAGRRAVAVR

EHVAGATMRNILRMAVGEKWSGCYGSPEGEAFRRSLDEAFAATGAVSNVGEWVPWLGWLD

VQGFKRKMKRLHDLHDHFYEKILVDHEERRRLAQASGGEFVATDLVDVLLQLSEESTKLE

SESEARLPRDGVKALIQDIIAGGTESSAVTIEWAMAELLRHPEAMAKATDELDRVVGSGR

WVAERDLPELHYIDAVVKETLRLHPVGPLLVPHYARERTVVAGYDVPAGARVLVNAWAIA

RDPASWPDAPDAFQPERFLGAAAAVDVRGAHFELLPFGSGRRICPAYDLAMKLVAAGVAN

LVHGFAWRLPDGVAAEDVSMEEHVGLSTRRKVPLFXVXEPRLPVHF*

>CYP93A4 Populus trichocarpa (black cottonwood)

MADIQGYIILFLLWLLSTILVRAILNKTRAKPRLPPSPLALPIIGHLHLLAPIPHQALHK

LSTRYGPLIHLFLGSVPCVVASTPETAKEFLKTHENSFCDRPKSTAVDFLTYGSADFSFA

PYGPYWKFMKKICMTELLGGRMLDQLLPVKHEEIRQFLQFLLKKANARESIDVGSQLIRL

TNNVISRMAMSQRCSDNDDEADEVRNLVHEVADLTGKFNLSDFIWFCKNLDLQGFGKRLK

EVRKRFDTMTERIIMEHEEARKKKKETGEGDPVKDLLDILLDISEDDSSEMKLTRENIKA

FILDIFAAGTDTSAVTMEWALAELINNPNILERAREEIDSVVGQSRLVQESDIANLPYVQ

AILKETLRLHPTGPIILRESSESCTINGYEIPARTRLFVNVWAINRDPNYWENPLEFEPE

RFLCAGENGKSQLDVRGQHFHFLPFGSGRRGCPGTTLALQMVQTGLAAMIQCFDWKVNGT

VDMQEGTGITLPRAHPLICVPVARLNPFPSF*

>CYP93B1v2Glycyrrhizaechinata

MEPQLVAVSVLVSALICYFFFRPYFHRYGKNLPPSPFFRLPIIGHMHMLGPLLHQSFHNL

SHRYGPLFSLNFGSVLCVVASTPHFAKQLLQTNELAFNCRIESTAVKKLTYESSLAFAPY

GDYWRFIKKLSMNELLGSRSINNFQHLRAQETHQLLRLLSNRARAFEAVNITEELLKLTN

NVISIMMVGEAEEARDVVRDVTEIFGEFNVSDFIWLFKKMDLQGFGKRIEDLFQRFDTLV

ERIISKREQTRKDRRRNGKKGEQESGDGIRDFLDILLDCTEDENSEIKIQRVHIKALIMD

FFTAGTDTTAISTEWALVELVKKPSVLQKVREEIDNVVGKDRLVEESDCPNLPYLQAILK

ETFRLHPPVPMVTRRCVAECTVENYVIPEDSLLFVNVWSIGRNPKFWDNPLEFCPERFLK

LEGDSSGVVDVRGSHFQLLPFGSGRRMCPGVSLAMQEVPALLGAIIQCFDFQVVGPKGEI

LKGDDIVINVDERPGLTAPRAHNLVCVPVERRSGGGPLKIIEC

>CYP93C4 Glycine max (soybean)

MLLELALGLCVLAWFLHLRPTPSAKSKALRHLPNPPSPKPRPPFIGHLHLLKDKLLHYAL

IDLSKKHGPLFSLSFGTMATVGGSTPELFKLFLQTHEGTSFNTRFQTSAIRRLTYDNSVA

MVPFGPYWKFVRKLIMNDLLNATTDNKLRPLRTQQIRKFLRVMAQSAEAQKPLDVTEELL

KWTNSTISMMMLGEAEMIRDIAREVLKIFGEYSLTDFIWPLKYLKVGKYEKRIDDILNKF

DPVVERVIKKRREIVRRRKNGEVVEGEASGVFLDTLLEFAEDETMEIKITKEQIKGLVVD

FFSAGTDSTAVATEWALAELVRRSTAVVGKDRLVDEVDTQNLPYIRAIVKETFRMHPPLP

VVKRKCTEECEINGYVIPEGALVLFNVWQVGRDPKYWDRPSEFRPERFLETGAEGEAGPL

DLRGQHFQLLPFGSGRRMCPGVNLATSGMATLLASLIQCFDLQVLGPQGQILKGDDPKVS

MEERAGLTVPRAHSLVCVPLARIGVASKLLS

>CYP93D1 Arabidopsis thaliana

MVDLQYFSVIILVCLGITVLIQAITNRLRDRLPLPPSPTALPIIGHIHLLGPIAHQALHK

LSIRYGPLMYLFIGSIPNLIVSSAEMANEILKSNELNFLNRPTMQNVDYLTYGSADFFSA

PYGLHWKFMKRICMVELFSSRALDSFVSVRSEELKKLLIRVLKKAEAEESVNLGEQLKEL

TSNIITRMMFRKMQSDSDGGEKSEEVIKMVVELNELAGFFNVSETFWFLKRLDLQGLKKR

LKNARDKYDVIIERIMEEHESSKKNATGERNMLDVLLDIYEDKNAEMKLTRENIKAFIMN

IYGGGTDTSAITVEWALAELINHPEIMKKAQQEIEQVVGNKRVVEESDLCNLSYTQAVVK

ETMRLHPGGPIFVRESDEECAVAGFRIPAKTRVIVNVWAIGRDSNQWEDPLEFRPERFEG

SEWKVMSEKMMSFGAGRRSCPGEKMVFRFVPIILAAIIQCFELKVKGSVDMDEGTGSSLP

RATPLVCVPVAKEATQSFSLLEPNVNF*

>CYP93E1 Glycine max (soybeans, Fabales)

MLDIKGYLVLFFLWFISTILIRSIFKKPQRLRLPPGPPISVPLLGHAPYLRSLLHQALYK

LSLRYGPLIHVMIGSKHVVVASSAETAKQILKTSEEAFCNRPLMIASESLTYGAADYFFI

PYGTYWRFLKKLCMTELLSGKTLEHFVRIRESEVEAFLKRMMEISGNGNYEVVMRKELIT

HTNNIITRMIMGKKSNAENDEVARLRKVVREVGELLGAFNLGDVIGFMRPLDLQGFGKKN

METHHKVDAMMEKVLREHEEARAKEDADSDRKKDLFDILLNLIEADGADNKLTRESAKAF

ALDMFIAGTNGPASVLEWSLAELVRNPHVFKKAREEIESVVGKERLVKESDIPNLPYLQA

LLKETLRLHPPTPIFAREAMRTCQVEGYDIPENSTILISTWAIGRDPNYWDDALEYKPER

FLFSDDPGKSKIDVRGQYYQLLPFGSGRRSCPGASLALLVMQATLASLIQCFDWIVNDGK

NHHVDMSEEGRVTVFLAKPLKCKPVPRFTPFAA

>CYP93F1 rice

MDHQLVARGLFKPLLLFVAGLIVLYALRRRRRHRRSSGLRLPPSPFGLPILGHLHLLAPL

PHQALHRLAARHGPLLFLRLGSVPCVAACSPDAAREVLKTHEAAFLDRPKPAAVHRLTYG

GQDFSFSAYGPYWRFMKRACVHELLAGRTLDRLRHVRREEVARLVGSLRASADGGERVDV

DAALMGLTGDIVSRMVMGRRWTGDDNDAEEMRSVVAETAELTGTFNLQDYIGVFKYWDVQ

GLGKRIDAVHRKFDAMMERILTAREAKRKLRRQAAADGEDDEKDLLDMLFDMHEDEAAEM

RLTRDNIKAFMLDIFAAGTDTTTITLEWALSELINNPPVLRKLQAELDAVVGGARLADES

DIPSLPYLQAVAKETLRLHPTGPLVVRRSLERATVAGYDVPAGATVFVNVWAIGRDAAWW

PEPTAFRPERFVSGGGGGGTAADVRGQHFHLLPFGSGRRICPGASLAMLVVQAALAAMVQ

CFEWSPVGGAPVDMEEGPGLTLPRKRPLVCTVSPRIHPLPAAASASLT*

>CYP93G1 rice

MASLMEVQVPLLGMGTTMGALALALVVVVVVHVAVNAFGRRRLPPSPASLPVIGHLHLLR

PPVHRTFHELAARLGPLMHVRLGSTHCVVASSAEVAAELIRSHEAKISERPLTAVARQFA

YESAGFAFAPYSPHWRFMKRLCMSELLGPRTVEQLRPVRRAGLVSLLRHVLSQPEAEAVD

LTRELIRMSNTSIIRMAASTVPSSVTEEAQELVKVVAELVGAFNADDYIALCRGWDLQGL

GRRAADVHKRFDALLEEMIRHKEEARMRKKTDTDVGSKDLLDILLDKAEDGAAEVKLTRD

NIKAFIIDVVTAGSDTSAAMVEWMVAELMNHPEALRKVREEIEAVVGRDRIAGEGDLPRL

PYLQAAYKETLRLRPAAPIAHRQSTEEIQIRGFRVPAQTAVFINVWAIGRDPAYWEEPLE

FRPERFLAGGGGEGVEPRGQHFQFMPFGSGRRGCPGMGLALQSVPAVVAALLQCFDWQCM

DNKLIDMEEADGLVCARKHRLLLHAHPRLHPFPPLL*

>CYP93H1 Aquilegia formosa x Aquilegia pubescens Ranunculales

MAFEILFYIILIFSLSSIFGRKFLSAKSQKCISNRLPPGPKGLPIIGHLHLLNNTPHQTF

HNLCSRYGPFIHVRLGSVFCIVASSAEYAKETLVTNGLAFASRSVNIASDLLTYGSAGFG

FAPYGPQWKFMKKLVTTELLSDKNMTQLKYVRSDEASQLVQLLLDNATSGTVVNVSNEVT

MLSNNIISRMMWNIRCSGEDEDGKEIISIIRECTEILAQFNLSDFIPFLGKIDLQGVRKR

AMNIHLRYDAILEIIIKKRHEERRKNKERNMQDAGGDNGDDHNSNNFLNILLDAMEDENA

KTPVTIENIKALMFDFLNAGTDTSATVVEWSLSELINHPTIMAKARQEIDTIVGKDRLVQ

ESDLPNLPYLQAIFKESLRLHPPVTLFGRESIQDCKIGGYDIPAKTVLFLNIWSINRDPN

YWKTPLEFRPERFMPHSDQKEGDDNEYLLEYRGQHFNYLPFGAGRRGCPGMSLAALISPR

VLALLIQCFDWKIACNDKGVAPKLVDLTERPGLTVPKLHPLMLIPSVRLNPFPISL

>CYP94A1 Vicia sativa

MFQFHLEVLLPYLLPLLLLILPTTIFFLTKPNNKVSSTSTNNNIITLPKSYPLIGSYLSF

RKNLHRRIQWLSDIVQISPSATFQLDGTLGKRQIITGNPSTVQHILKNQFSNYQKGTTFT

NTLSDFLGTGIFNTNGPNWKFQRQVASHEFNTKSIRNFVEHIVDTELTNRLIPILTSSTQ

TNNILDFQDILQRFTFDNICNIAFGYDPEYLTPSTNRSKFAEAYEDATEISSKRFRLPLP

IIWKIKKYFNIGSEKRLKEAVTEVRSFAKKLVREKKRELEEKSSLETEDMLSRFLSSGHS

DEDFVADIVISFILAGKDTTSAALTWFFWLLWKNPRVEEEIVNELSKKSELMVYDEVKEM

VYTHAALSESMRLYPPVPMDSKEAVNDDVLPDGWVVKKGTIVTYHVYAMGRMKSLWGDDW

AEFRPERWLEKDEVNGKWVFVGRDSYSYPVFQAGPRVCLGKEMAFMQMKRIVAGIVGKFK

VVPEAHLAQEPGFISFLSSQMEGGFPVTIQKRDS

>CYP94B1 Arabidopsis thaliana

MEMLNAIILILFPIIGFVLIFSFPTKTLKAKTASPSNPTSYQLIGSILSFNKNRHRLLQW

YTDLLRLSPSQTITVDLLFGRRTIITANPENVEHILKTNFYNFPKGKPFTDLLGDLLGGG

IFNSDGELWSSQRKLASHEFTMRSLREFTFEILREEVQNRLIPVLSSAVDCGETVDFQEV

LKRFAFDVVCKVSLGWDPDCLDLTRPVPELVKAFDVAAEISARRATEPVYAVWKVKRFLN

VGSEKRMREAIKTVHLSVSEIIRAKKKSLDIGGDVSDKQDLLSRFLAAGHGEEAVRDSVI

SFIMAGRDTTSAAMTWLFWLLSQNDDVETKILDELRNKGSLGLGFEDLREMSYTKACLCE

AMRLYPPVAWDSKHAANDDILPDGTPLKKGDKVTYFPYGMGRMEKVWGKDWDEFKPNRWF

EEEPSYGTKPVLKSVSSFKFPVFQAGPRVCIGKEMAFTQMKYVVGSVLSRFKIIPVCNNR

PVFVPLLTAHMAGGLKVKIKRREQCDSMYI*

>CYP94C1 Arabidopsis thaliana

MLLIISFTIVSFFFIIIFSLFHLLFLQKLRYCNCEICHAYLTSSWKKDFINLSDWYTHLL

RRSPTSTIKVHVLNSVITANPSNVEHILKTNFHNYPKGKQFSVILGDLLGRGIFNSDGDT

WRFQRKLASLELGSVSVRVFAHEIVKTEIETRLLPILTSFSDNPGSVLDLQDVFRRFSFD

TISKLSFGFDPDCLRLPFPISEFAVAFDTASLLSAKRALAPFPLLWKTKRLLRIGSEKKL

QESINVINRLAGDLIKQRRLTGLMGKNDLISRFMAVVAEDDDEYLRDIVVSFLLAGRDTV

AAGLTGFFWLLTRHPEVENRIREELDRVMGTGFDSVTARCDEMREMDYLHASLYESMRLF

PPVQFDSKFALNDDVLSDGTFVNSGTRVTYHAYAMGRMDRIWGPDYEEFKPERWLDNEGK

FRPENPVKYPVFQAGARVCIGKEMAIMEMKSIAVAIIRRFETRVASPETTETLRFAPGLT

ATVNGGLPVMIQERS

>CYP94D1 Arabidopsis thaliana

MALLIFIFLLCFPISIFFIFFTKKSSSEFGFKSYPIVGSFPGLVNNRHRFLDWTVETLSR

CPTQTAIFRRPGKQQLIMTANPSNVEYMLKTKFESFPKGQQFTSVLEDFLGHGIFNSDGD

MWWKQRKTASYEFSTKSLRDFVMSNVTVEINTRLVPVLVEAATTGKLIDLQDILERFAFD

NICKLAFNVDCACLGHDGAVGVNFMRAFETAATIISQRFRSVASCAWRIKKKLNIGSERV

LRESIATVHKFADEIVRNRIDQGRSSDHKEDLLSRFISKEEMNSPEILRDIVISFILAGR

DTTSSALSWFFWLLSMHPEVEDKILQELNSIRARTGKRIGEVYGFEHLKMMNYLHAAITE

SLRLYPPVPVDIKSCAEDNVLPDGTFVGKGWAITYNIFAMGRMESIWGKDCDRFDPERWI

DETNGCFRGEDPSKFPAFHAGPRMCVGKDMAYIQMKSIVAAVLERFVVEVPGKERPEILL

SMTLRIKGGLFARVQERS*

>CYP94E4 Zea mays (maize)

MEAIHLAYVLVFLLPILLLRLRRRGPPPVKRPRTTTAHCPHPSPVLGNTLHFIRNRRRFF

DWYADMLRAAPSGAIEAWGPLGAGHAVTTASPADVDHLLRSSFDKYAKGALFRDATADLI

GDGLFAADGRLWSLQRKLASHAFSSRSLRRFTDGVLDVHLRRRFLPLLDAAARDGGAVDL

QDALRRFGFRTICHVAFGVEGLDDDARRQDALFAAFDAAVEISFRRALTPATFVRRLTKL

LDVGKSRRLREAVHAIDDYAMSVVESKVARRRNSLDDGAADLLSRFMAAMDDGGGSDSEL

GAMFPTPAAKLRFLRDVVVTFVLAGKDTTSSALTWFFWLLAANPRCERRAHEEAASCCGD

GGDVKGMHYLHAAITEAMRLYPPVPFNGRVAVRDDVLPSGAALRAGWYANYSAYAMGRME

KLWGKDCLEFVPERWLGEGGEFVPVDAARFPVFHAGPRVCLGKEMAYVQMKTVAAAVLRR

FRLDVVAPVANMEAPPAYEMTATMKMKGGLLVRLCSRED*

>CYP94F2 Vitis vinifera

MMDLNAAALFFCLCLSFIFFRSLRSPPTKNSCPHSYPIIGNLIALLRNRHRFHDWVADML

SRTPSLTLQVNTFLNASHGVCTANPLNVNHLLVSNFPNYIKGSRFHDFFHELLGDGIFNV

DGHLWTVQRKISSHEFNTKSLKHFISDTVQSELSTRLIPFLSSACENNQVIDLQDVLRKA

MFDNICNLAFGADPACLSSEAVGENSLNLSFVQAFDDAVEISASRSLLPIHVIWKIKRFF

NIGSEKRLKEAVGIINEYANMILKSKEDQIGSGDCGNLDLLSRFMSSSSNFGLGFDDQEH

KRKFLRDIVISFILAGKDSTSTALTWFFWLMAGNPRCEGLILAELSEASPAPATSPVIFS

YDDLKGLNYLHAAISESLRLFPPVPIDSRLAVDDDILPDGTHVRKGWFADYSAYAMGRMH

QVWGPDCREYRPERWLDDDGRFRPSDQFRFPVFHCGPRLCLGKEMAYVQMKSIAASVMRE

FEIVAVDGGGCAGKMADPPYTPSIVLKMRGGLPVRVKRRRQPNAIDFC*

>CYP94G1 Physcomitrella patens (moss)

MMDRELVTLLYTAGILLVVTLWCIWYHHPKYGKNRGPKVYPLLGSYLSLLHNKSRILDWM

VDLIRDSPTMTVRTVRPGGRQFHITAGPANVEHILKTNFENYPKGENSYANLHDLLGNGI

FNIDGKSWKLQRKVASHEFTTQSLKNFMVGAVHDELRGRFIPVLQECCNTGRTVDLQDLL

ARFTFDTICKLGFGVDPACLDLCFPSVRFANAFDTATSITANRFITFASVWKTMRALNVG

SEKKLRAAVADIDDFAMFVIQNRRKQVAGQSNRQTDNSSDADDAAHLDLLSRFMGLTAad

qdrrdfdtqdpscdqNEGPQLGYSDEFLRDIVISFILAGRDTSTSSLTWFFWNLEHHRQV

EDAICKEVSEILKNRLVEDKDHNKHVPTSFFSFEELKKMHYLHAAVSESLRLYPPVPIEM

KLAHSSDEWPDGTRIDPNSTIIYHPYAMGRMERIWGPDCMKFKPERWLKDGVFVQESPYK

HAVFQAGPRMCLGRELALMQIKMVVAVLLQRFRFSSQKGFTPEYDLNLTLPMKNGFPVSV

QSKVPM*

>CYP94H1 Physcomitrella patens (moss)

MSTPNYMSPEMGRFEKWALLLREESEEHTLAFVATILFVAVNALIFIWWHHPLYGKNIGP

RVYPFVGSLPSAIQHAHRLLDFSVETLRKSPTLTIRYVQSGYTAYSTANVENVEYVLKTK

FDNFVKGERMGDVLFDLLGRGIFNADGNLWKLQRKLASHEFSSRSLREFGVECVQKELQN

RLVPVLSQFSENGNVVDLQDLLMRFSFDNICQLGFGVDPNCLEPSLPPVKFAEAFDKANE

CTLLRFRTFPIMLRLYKFFNIGIERGLKESMAVVHNFAQEVIEARRKEFNENHGDIGHAR

QDLLSRDAKEKQKASDIFLRDMVISFVLAGRDTTSLGLSWFFYALGHNPHVEAKIYDEIK

EQLQLQAQEDDSLPSSRPPGQLFTFEQLKKLHYLHAALHESLRLFPPVPWDSKHAVRDDV

LPDGTVILKGERVTFNIYAMARMEANWGPDCNEFKPERWLKDGVFVPESPFKFATFQAGP

RICLGKEMALIQMKLVASSLVYCFKFTLLEDPPRTCLSFVFKMLNGFPGDVHKRAVST*

>CYP94J1v1 Selaginella mollendorffii

MEQGTLAAALLISCVGFFVWFYLKNRHENGETVPRMYPLLGTMPELLKNKDRILEWTTEY

LAKSPGHTITLKRWGAKPFKLTSNAQNVEYILKTNFDNYPKGEYVCDTLRDLLGDGIFNA

DAGLWKLQRKLASYEFTTRSLHDYLMDSVAEKIEKRLLPTIASICGRRVDLQDVFMRFAF

DSICKLAFGVDPMSLDPSFPTIAFARAFDESTRLSTERFYQVHPLLWKIKRYFNLGSEKH

LKEYLAIVNEFAAMVIKNRRKKTGARENQDLLSRFMALEMEDTASSYSDKFLRDIIISFV

LAGKDTTSVTLSWFFWLLSKHPKVENKIIQEIVDVAERNHEPGRRMKHFAYSELREMNYL

QAALSESLRLYPAVPFDSKGAKGPDVLPDGSRIEKGTRVTYQIYAMGRMESLWGKDCLEF

KPERWLSSTGSFVNESPYKFTAFQAGPRICIGKEMAMLQMKSLVAALLPKFKFEMASDTE

PRYSINMTLAIKNGLPVIPRARE*

>CYP96A1 Arabidopsis thaliana

MALITLLEVSISLLFFSFLYGYFLISKKPHRSFLTNWPFLGMLPGLLVEIPRVYDFVTEL

LEASNLTYPFKGPCFGGLDMLITVDPANIHHIMSSNFANYPKGTEFKKIFDVLGDGIFNA

DSELWKDLRKSAQSMMTHQDFQRFTLRTIMSKLEKGLVPLLDYVAEKKQVVDLQDVFQRF

TFDTSFVLATGVDPGCLSTEMPQIEFARALDEAEEAIFFRHVKPEIVWKMQRFIGFGDEL

KMKKAHSTFDRVCSKCIASKRDEITNGVINIDSSSKDLLMCYMNVDTICHTTKYKLLNPS

DDKFLRDMILSFMLAGRDTTSSALTWFFWLLSKNPKAITKIRQEINTQLSPRTNDFDSFN

AQELNKLVYVHGALCEALRLYPPVPFQHKSPTKSDVLPSGHRVDASSKIVFCLYSLGRMK

SVWGEDASEFKPERWISESGRLIHVPSFKFLSFNAGPRTCLGKEVAMTQMKTVAVKIIQN

YEIKVVEGHKIEPVPSIILHMKHGLKVTVTKRSNLL*

>CYP96B2 rice

MAFSSILQLTLCFLCFSVFYYYHIKSKRKNPAIPVCWPLVGMLPDLLVNRHQLHDWITSF

LTASQLNFRFIGPTMSSNMRFFFTCDPANVRHIFTSNFANYPKGPDFAEIFDDTLGDGIF

NVDGDSWRRQRAKTQLLMYNHRFQSFVSRCSSDKVENALLPLLSHFAGTGERCNLQDVFM

RLTFDMSTMLASGEDPGCLAISLPMPKVPFVRAVDYTTRVLLVRHIIPLSLWKLARRLGV

GFERKMAEALRTINQFIYETIVKRRAKKATEGIEDSEDLLSSYLKDDDENADTFLRDTTM

TLIAAGRDTIGSALSWFFYLLTKNPHVASKILEELDSVERATTTPDGMVTFDPDELKSLV

YLHAAVCESLRLYPPVPLDHKGVVAADVMPSGHKVRPGDKIVVSIYAMGRTESVWGSDCM

EFRPERWISDHGKLRYVPSYKFTPFITGPRTCLGKDMALVQLKVVAATVVKNFEAEAVPG

HIVEPKLSMVLHMKNGLMVRVKRR*

>CYP96C1 Catharanthus roseus (Madagascar periwinkle)

MALASIEKVATGLICFILLIFSLKRIGHPRDWALVGILSVWFPSLGHIYEKLAKSLAKND

KTFVLKGSFLSKQDVIFTCDPANMHHVMSTNFSNYPKGPENRNVFDVYGEMLFTADHEKW

KSHRKVTNAYFHDQRFNGFSQKVNKEVIEKELFPFLDHAAEEALVFDLQDVFQRLMLDSS

SILTTGQNHRSLRVGLPYDETLEAINIANYQIFVRHILPAKIWKFQKWLGIMGEKKIKKA

WRILDDISVEYMNRRKKEISSTISSQEDMDVVKFSEEDHVVLKSVDASDNLLRDTVKGIL

LAGTDTTATVLSWFFWLILKNPRVEQKIREEIELYLKQKNGEHGLYTNPEELNELMYLHA

AIYETMRLYPAAPFTSRKSIQADVFPTGHQVNPNTTIVMAYYAVGRMKSIWGEDCLEFKP

ERWLSDKGKLIPVQTNKFLAFGTGPRICPGKELGLNRVKAVAAAIIPKYSFKIMRNKPVM

PAACATLYLKDGLIVRVNKII

>CYP96D1 rice

MGPLWTFILLYPEIFLAIICFFWFSLFRPIRQRQKSNLPVNWPVFGMLPFLVQNLHYIHD

KVADVLREAGCTFMVSGPWFLNMNFLITCDPATVNHCFNANFKNYPKGSEFAEMFDILGD

GLLVADSESWEYQRRMAMYIFAARTFRSFAMSTITRKTGSVLLPYLDHMAKFGSEVELEG

VFMRFSLDVTYSTVFAADLDCLSVSSPIPVFGQATKEAEEAVLFRHVIPPSVWKLLRLLN

VGTEKKLTNAKVVIDQFIYEEIAKRKAQASDGLQGDILSMYMKWSIHESAHKQKDERFLR

DTAVGFIFAGKDLIAVTLTWFFYMMCKHPHVEARILQELKGLQSSTWPGDLHVFEWDTLR

SAIYLQAALLETLRLFPATPFEEKEALVDDVLPNGTKVSRNTRIIFSLYAMGRIEGIWGK

DCMEFKPERWVSKSGRLRHEPSYKFLSFNTGPRSCLGKELSLSNMKIIVASIIHNFKVEL

VEGHEVMPQSSVILHTQNGMMVRLKRRDAA*

>CYP96E1 rice

MELLPWLLGFVVKYPEIMASAACFLLLFCRFRRRSKRIPTNWPVVGALPAIVANAGRVHD

WVTEFLRAAAMSHVVEGPWGSPGDVLITADPANVAHMFTANFGNYPKGEEFAAMFDVLGG

GIFNADGESWSFQRRKAHALLSDARFRAAVAASTSRKLGGGLVPLLDGVAASGAAVDLQD

VFMRLTFDLTAMFVFGVDPGCLAADFPTVPFAAAMDDAEEVLFYRHVAPVPWLRLQSYLK

IGHYKKMAKAREVLDASIAELIALRRERKAADANATGDADLLTAYLACQDEIGMDGAAFD

AFLRDTTLNLMVAGRDTTSSALTWFFWLLSNHPGVEARILAELRAHPPSPTGAELKRLVY

LHAALSESLRLYPPVPFEHKAAARPDTLPSGAAVGPTRRVIVSLYSMGRMEAVWGKGCEE

FRPERWLTPAGRFRHERSCKFAAFNVGPRTCLGRDLAFAQMKAVVAAVVPRFRVAAAAAP

PRPKLSIILHMRDGLKVKVHRRQED*

>CYP96F1 Populus trichocarpus (cottonwood)

MAILEYFHIIIALVCILLFCHWCRNTVTPVTNWPVVGMLPGLLFKAQNIHEYATQLLKQS

GGTFEFKGPWFANMNILLTADPLNVRHISTTNFVNYPKGPEYKKIFEPYGDGVLNSDFES

WKSFRKLIHSMIKDNKFQVSLERSLREKIVEGLIPVLEHASRQEIELDMQDVFQRFTFDN

ICLLVLGFDPQSLSVDLPEIAYKTAFDDVEEAVFYRHIVPESIWKLQKWLNVGEEKKLSQ

AMDTIDNFLEQCISSKKEEIRQRKAQNMVQVEDNDQDDYDLITACIKEGEEAEQMDASKR

TDKYLRDIGFNFIAAGKDTVNAALTWFFWLVATHHEVEEKIVEEIRANMKSKGDHTINGM

FFNLEELNKLVYLHGAICETLRLYPAVPFNYRVSVEADTLPSGHLVKENTKVMFSLYSMG

SMEEIWGDDCLAFKPERWISERGGIIHVPSYKYIAFNTGPRSCLGKEITFIQMKTIATAI

LLNFHLQVVEGHPVSPGLSVMLHMKHGLKLRVTKRCV*

>CYP96G1 Populus trichocarpus (cottonwood)

MAILTLIILFVATIFSIFFLPFIKKNSSPWILVRSLVNFYRMHDNQAEMLEQNNGTILVK

RSWFGGKDILLTSDPANVRHIMSTNFSNYPKGPEWKKQFEFFGDSVFTLDFEEWKHHRKV

IRSYISHRSFQQFAGKIVRDCIEIELSSVLDRVSNQQIVVDFEGLLRRYIYYFACCISTG

YKPKFIDLAFSEDKFLKATDDACDAITVRYLVPESIWKLQRWFGLGKEKRLSEARKTIDK

IIDDYISMKQEEMSKGEIRNDEEDFSALKSYTTGNEIFEQADHKVIKDGTMSLIFAIEDT

TSSSLSRFFWIFTKNPKVETKIRQELEKIRPVTEARKSSSFFSEEEVSKMVYLQAALLET

LRLFPPASMVSKTAVEADTLPSGHHISQNTMVVISAYAMGRMRTIWGQDCLEFKPERWIM

EDGRIRHEPPHKFSAFGSGPRICPGKDLGLTLLKTFAATIIYNYHIQVVEDRVGAPKNNF

MLHLDHGLMVRLNKRWT*

>CYP96H1 Populus trichocarpus (cottonwood)

MVAESILAIAFFLVLICLFRSKNGLPWNWPLLGMSPTLLLNSHRLYELADEILEISGGTY

LFKGLWFSNMDMWFTSDPENVHYITTTNYWNYPKGPESMQVFDTLGNSLFNLDFEEWTYY

RGLLHGFFSHQKFHQFVPKVLVDNVNKGLVPFLEDVAKQALVVDLQDMFKRHIYDAACAI

ATGYNPKTLSIGFEENAFVRAMDDACVAMLTRHILPGRCWKLLRWLQIGSEKRLSVAKGT

LRQIVTNYMATKREELSAGAKTKEDEETFDVLRSFLTINDVNDKEHPDEIVRDSTIGIIF

AAYDTSSATLSWFFWLLSKNPHVETKIREELDSNFSVKEGQNWQLNSRKELSKLVYLHAT

LCETLRLYPPVPLQRRTPVRPDIFPSGHHVDPKAIVVLSGYAIGRMARVWGQDCHEFKPE

RWINEKGDLKYERSAKFFTFNAGPRICPGKEMAFSIMKAAATTILYNYHVQVVETRPVTP

KASIILQMKHGLRARICSRWT*

>CYP96J1 Medicago truncatula

MQFANIHFSKTTMSIIQCIGGFVTILIFLYIYYSRRNRDELLLINWPIIGILPSFLCHLS

DYHDYSTIVLKRYGGTCRFQGPWFTNTSFIALADQMNVNYITKKNCGNYRKGSKYHDIFE

VLGGSIFNSDSDDVWKQEKTMFHLVLGRKSFKNMFEQSIQKKVENYLIPFLNDVSEAGAH

VDLQDAFNRFTFDSSCMILFGFDPNCLPNKFNQLRKIPYKESLPVMEEVILYRHFIPSSL

WKLQKWLNVGQEKKFKVAQEYLDRFLYESITFSHGEEQSKCSDEEMDQCFLGMVKALKKE

GHGKGEISEKYLRDTAFTMIFAGNGTISSALSWFFWLLSTYPIVEEKIIQEIKDNWLTQE

GNRITLRHVDLDKLVYLHGAICETLRLYPPIPFEHICSIKSDILPSGYHVSPNTTLIYSL

YSMGRMEQIWGEDCMEFKPERWISERGDII

>CYP96K1 Gossypium hirsutum

MMAVLFVYLGFMVFGIIVLRFLYRFIDNNGLPRNWPFVGMIPTLLLNIHRPHDKVAQVLR

RSNGTFFYRGLWFTNTSFLATSDPENVRYILSSNSSVYLKGPEWLKQFDIFGEALFNSDG

EAWKCHRRVFHAFLNHPQYRQSLSKVLHQRIEEALVKVLEYVSGREMVVNLQDLLAGHAF

DIGCITGVGFDPGLLSIEFPENRFQKAMSDTLEAAFYRYVVPDSLWKLQSWPQIGKGKKR

SDAWKALDDLLTQFISTQRHKSTKSVASSGSNEEHDFNFLNCYLTGHEITGPTPKESLIR

DNLIHFLFASDGTYSLTLTWFFYLISKAPVVENKIREEIKRHLSMKQVEGSLQIPSNYDE

LSKLTYLHAALCETLRLYPPIPFDFRTCTKQEYLPSGHRVDQNTRIIIGIHAMGRMESLW

GEDCYAFKPERWIGEDGKIKRESPTKFSAFLAGPRICPGKEVSFLLMKATATAIIHNYNV

HVVEGQNIGPKNSVVYQMKKGLMVRIKKRWS

>CYP97A3 Arabidopsis thaliana

MAMAFPLSYTPTITVKPVTYSRRSNFVVFSSSSNGRDPLEENSVPNGVKSLEKLQEEKRR

AELSARIASGAFTVRKSSFPSTVKNGLSKIGIPSNVLDFMFDWTGSDQDYPKVPEAKGSI

QAVRNEAFFIPLYELFLTYGGIFRLTFGPKSFLIVSDPSIAKHILKDNAKAYSKGILAEI

LDFVMGKGLIPADGEIWRRRRRAIVPALHQKYVAAMISLFGEASDRLCQKLDAAALKGEE

VEMESLFSRLTLDIIGKAVFNYDFDSLTNDTGVIEAVYTVLREAEDRSVSPIPVWDIPIW

KDISPRQRKVATSLKLINDTLDDLIATCKRMVEEEELQFHEEYMNERDPSILHFLLASGD

DVSSKQLRDDLMTMLIAGHETSAAVLTWTFYLLTTEPSVVAKLQEEVDSVIGDRFPTIQD

MKKLKYTTRVMNESLRLYPQPPVLIRRSIDNDILGEYPIKRGEDIFISVWNLHRSPLHWD

DAEKFNPERWPLDGPNPNETNQNFSYLPFGGGPRKCIGDMFASFENVVAIAMLIRRFNFQ

IAPGAPPVKMTTGATIHTTEGLKLTVTKRTKPLDIHPYRYFQWILTG*

>CYP97B3 Arabidopsis thaliana

MAFPAAATYPTHFQGGALHLGRTDHCLFGFYPQTISSVNSRRASVSIKCQSTEPKTNGNI

LDNASNLLTNFLSGGSLGSMPTAEGSVSDLFGKPLFLSLYDWFLEHGGIYKLAFGPKAFV

VISDPIIARHVLRENAFSYDKGVLAEILEPIMGKGLIPADLDTWKLRRRAITPAFHKLYL

EAMVKVFSDCSEKMILKSEKLIREKETSSGEDTIELDLEAEFSSLALDIIGLSVFNYDFG

SVTKESPVIKAVYGTLFEAEHRSTFYFPYWNFPPARWIVPRQRKFQSDLKIINDCLDGLI

QNAKETRQETDVEKLQERDYTNLKDASLLRFLVDMRGVDIDDRQLRDDLMTMLIAGHETT

AAVLTWAVFLLSQNPEKIRKAQAEIDAVLGQGPPTYESMKKLEYIRLIVVEVLRLFPQPP

LLIRRTLKPETLPGGHKGEKEGHKVPKGTDIFISVYNLHRSPYFWDNPHDFEPERFLRTK

ESNGIEGWAGFDPSRSPGALYPNEIIADFAFLPFGGGPRKCIGDQFALMESTVALAMLFQ

KFDVELRGTPESVELVSGATIHAKNGMWCKLKRRSK*

>CYP97C1 Arabidopsis thaliana

MESSLFSPSSSSYSSLFTAKPTRLLSPKPKFTFSIRSSIEKPKPKLETNSSKSQSWVSPD

WLTTLTRTLSSGKNDESGIPIANAKLDDVADLLGGALFLPLYKWMNEYGPIYRLAAGPRN

FVIVSDPAIAKHVLRNYPKYAKGLVAEVSEFLFGSGFAIAEGPLWTARRRAVVPSLHRRY

LSVIVERVFCKCAERLVENVAALCKRRNSCKYERNFSSQMTLDVIGLSLFNYNFDSLTTD

SPVIEAVYTALKEAELRSTDLLPYWKIDALCKIVPRQVKAEKAVTLIRETVEDLIAKCKE

IVEREGERINDEEYVNDADPSILRFLLASREEVSSVQLRDDLLSMLVAGHETTGSVLTWT

LYLLSNSSALRKAQEEVDRVLEGRNPAFEDIKELKYITRCINESMRLYPHPPVLIRRAQV

PDILPGNYKVNTGQDIMISVYNIHRSSEVWEKAEEFLPERFDIDGAIPNETNTDFKFIPF

SGGPRKCVGDQFALMEAIVALAVFLQRLNVELVPDQTISMTTGATIHTTNVCQWIVYEGE

PKVKTRIYVFMIIDWCEWTCFIVLLRNNHKKMNYGK*

>CYP97E5 Ostreococcus tauri, Prasinophyte green algae

MVARARVHASRGVDARRVRARGRARVDVIARAVKEPSSAPEEALPDENFKPEQLKFQDIV

SLWVTQILQTYGGKESKDNAPVCEGVIDDLVGGPIFLALYPYFRRYGGVFKLAFGPKVFM

VLSDPVIVREVLKEKPFSFDKGVLAEILEPIMGQGLIPAPYAVWKNRRRQLVPGFHKAWL

DHMVGLFGHCSNELVRNLDKSAEDGEVVDMEERFCSVSLDIIGLAVFNYDFGSVTKESPI

ISAVYNCLQEAAHRSTFYFPYWNIPFATDIVPRQREFKQNMKIINETLNGLIQKAQKFEG

TEDLEELQNRDYSKVKDPSLLRFLVDIRGADVTDSQLRDDLMTMLIAGHETTAAVLTWGL

FCLMQNPELMKRIQADIDEVMGDDDRTPTYDDIQKLESVRLCIAEALRLYPEPPILIRRC

LEDVTLPKGAGDAEVTLIKGMDIFISVWNLHRSPECWENPEEFDPFRFKRPFANPGVKDW

AGYNPELFTGLYPNEVASDFAFIPFGAGARKCIGDQFAMLEATIAMAMVLRRYDFELTTD

PKDIGMTMGATIHTEKGLPCRVRRRQPVTTATAAAV*

>CYP98A3 Arabidopsis thaliana

MSWFLIAVATIAAVVSYKLIQRLRYKFPPGPSPKPIVGNLYDIKPVRFRCYYEWAQSYGP

IISVWIGSILNVVVSSAELAKEVLKEHDQKLADRHRNRSTEAFSRNGQDLIWADYGPHYV

KVRKVCTLELFTPKRLESLRPIREDEVTAMVESVFRDCNLPENRAKGLQLRKYLGAVAFN

NITRLAFGKRFMNAEGVVDEQGLEFKAIVSNGLKLGASLSIAEHIPWLRWMFPADEKAFA

EHGARRDRLTRAIMEEHTLARQKSSGAKQHFVDALLTLKDQYDLSEDTIIGLLWDMITAG

MDTTAITAEWAMAEMIKNPRVQQKVQEEFDRVVGLDRILTEADFSRLPYLQCVVKESFRL

HPPTPLMLPHRSNADVKIGGYDIPKGSNVHVNVWAVARDPAVWKNPFEFRPERFLEEDVD

MKGHDFRLLPFGAGRRVCPGAQLGINLVTSMMSHLLHHFVWTPPQGTKPEEIDMSENPGL

VTYMRTPVQAVATPRLPSDLYKRVPYDM*

>CYP99A1 Sorghum bicolor

RLISAVILAVCSLISRRKPSPGSKKKRPPGPWRLPLIGNLLHLATSQPHVALRDLAMKHG

PVMYLRLGQVDAVVISSPAAAQEVLRDKDTTFASRPSLLVADIILYGSMDMSFAPYGGNW

RMLRKLCMSELLNTHKVRQLAAVRDSETLSLVRKVVYAAGAGGGGRGQRGEAPVVNLGRLVLSCSMAITGRATLGKLCGDEIMSVVDVAVLYGSGFCAGDLFPSLWFVDVVTGLTRRLWT

ARRRLDAIFDRILAECEARQRQEEKMTGDDGFLGVLLRIRDDDGEPETGGISTTSIKAILFDMLAGGTETTSSAAEWIMSELMRKPEAMAKAQAEVRGALDGKSPEDHEGQMDKLSYTRM

VVKEGLRLHPVLPLLLPRSCQETCDVGGFEVTKGTKVIVNAWALARSPERWHDPEEFRPERFADDDGSSAAVAVDYRGSQFEYIPFGSGRRMCPGNTFGLAALELMVARLLYYFDWSLPDGMRPEELDMDTVVGSTMRRRNHLHLVPSPYKETELTVGI

>CYP701A3 Arabidopsis thaliana

MAFFSMISILLGFVISSFIFIFFFKKLLSFSRKNMSEVSTLPSVPVVPGFPVIGNLLQLK

EKKPHKTFTRWSEIYGPIYSIKMGSSSLIVLNSTETAKEAMVTRFSSISTRKLSNALTVL

TCDKSMVATSDYDDFHKLVKRCLLNGLLGANAQKRKRHYRDALIENVSSKLHAHARDHPQ

EPVNFRAIFEHELFGVALKQAFGKDVESIYVKELGVTLSKDEIFKVLVHDMMEGAIDVDW

RDFFPYLKWIPNKSFEARIQQKHKRRLAVMNALIQDRLKQNGSESDDDCYLNFLMSEAKT

LTKEQIAILVWETIIETADTTLVTTEWAIYELAKHPSVQDRLCKEIQNVCGGEKFKEEQL

SQVPYLNGVFHETLRKYSPAPLVPIRYAHEDTQIGGYHVPAGSEIAINIYGCNMDKKRWE

RPEDWWPERFLDDGKYETSDLHKTMAFGAGKRVCAGALQASLMAGIAIGRLVQEFEWKLR

DGEEENVDTYGLTSQKLYPLMAIINPRRS*

>CYP701B1 Physcomitrella patens (moss)

MLNESTSGHSSDTCVQTSLGCRDGKRRLNEMLETKVIAHHVSHSPCAAIPGGLPVLGNLL

QLTEKKPHRTFTAWSKEHGPIFTIKVGSVPQAVVNNSEIAKEVLVTKFASISKRQMPMAL

RVLTRDKTMVAMSDYGEEHRMLKKLVMTNLLGPTQVHDHRvqqnppclkmchvyashskg

tpeekivcspafyrrispsgmefgcaeqkpiVEVLELGTCVSTWDMFDALVVAPLSAVIN

VDWRDFFPALRWIPNRSVEDLVRTVDFKRNSIMKGLIRAQRMRLANLKEPPRCYADIALT

EATHLTEKQLEMSLWEPIIESADTTLVTSEWAMYEIAKNPDCQDRLYREIVSVAGTERMV

TEDDLPNMPYLGAIIKETLRKYTPVPLIPSRFVEEDITLGGYDIPKGYQILVNLFAIAND

PAVWSNPEKWDPERMLANKKVDMGFRDFSLMPFGAGKRMCAGITQAMFIIPMNVAALVQH

CEWRLSPQEISNINNKIEDVVYLTTHKLSPLSCEATPRISHRLP*

>CYP701C1v2 Selaginella moellendorffii

VAEVPGLPFVGNLLQMTVERPHRKLTSWSNEYGPIYTIRTGQKSQVIVSSPELAREAVVA

KYSSISNRDLGSNLTILTRNRKIVAMSDYGDRYRMLKRMVVNNLLGQTSQKALHVQRENY

LRIALDGLFDELGRFPGSTGQVNARDCIANFLFRLGMHQASVFGRDIESVRVPELGAEVT

RWEIYRVLVQDVMKAAVQIDWRDFFPSLKWIPNRKFEDGIYKVERKRSAVTKALMEQHRQ

LSRSQQRDKCYCDVLLDNESHYSEDELLLAAWEPIIESSDTTLVTSEWALYELASGPKLQ

EKLYNEIKRVVGDERMVSEDDLPNLPFLNAVIKETLRKYSPVPILPPRYIHEQVELGGYT

IPAGYQLIVNIFGIHHDPKRWSNPETWDPSRFLGVEGGSFDMGLTDMRLMPFGGGKRICA

GMAQVFYVVPMIIATLVQHFEWTLPQGDMDKRNVVEDTVYLTTQKLEPLQACAKPRVPRR

LPSKTLNAVPSNNKVPEHKH*

>CYP702A1 Arabidopsis thaliana

MVEVYELLTVMVSLIVVKLFHWIYQSKNPKPNEKLPPGSMGFPIIGETFEFMKPHDAFQF

PTFIKERIIRYGPIFRTSLFGAKVIISTDIELNMEIAKTNHAPGLTKSIAQLFGENNLFF

QSKESHKHVRNLTFQLLGSQGLKLSVMQDIDLLTRTHMEEGARRGCLDVKEISSKILIEC

LAKKVTGDMEPEAAKELALCWRCFPSGWFRFPLNLPGTGVYKMMKARKRMLHLLKETILK

KRASGEELGEFFKIIFEGAETMSVDNAIEYIYTLFLLANETTPRILAATIKLISDNPKVM

KELHREHEGIVRGKTEKETSITWEEYKSMTFTQMVINESLRITSTAPTVFRIFDHEFQVG

SYKIPAGWIFMGYPNNHFNPKTYDDPLVFNPWRWEGKDLGAIVSRTYIPFGAGSRQCVGA

EFAKLQMAIFIHHLSRDRWSMKIGTTILRNFVLMFPNGCEVQFLKDTEVDNSSGSNPDCC

NG*

>CYP703A2 Arabidopsis thaliana

MILVLASLFAVLILNVLLWRWLKASACKAQRLPPGPPRLPILGNLLQLGPLPHRDLASLC

DKYGPLVYLRLGNVDAITTNDPDTIREILLRQDDVFSSRPKTLAAVHLAYGCGDVALAPM

GPHWKRMRRICMEHLLTTKRLESFTTQRAEEARYLIRDVFKRSETGKPINLKEVLGAFSM

NNVTRMLLGKQFFGPGSLVSPKEAQEFLHITHKLFWLLGVIYLGDYLPFWRWVDPSGCEK

EMRDVEKRVDEFHTKIIDEHRRAKLEDEDKNGDMDFVDVLLSLPGENGKAHMEDVEIKAL

IQDMIAAATDTSAVTNEWAMAEAIKQPRVMRKIQEELDNVVGSNRMVDESDLVHLNYLRC

VVRETFRMHPAGPFLIPHESVRATTINGYYIPAKTRVFINTHGLGRNTKIWDDVEDFRPE

RHWPVEGSGRVEISHGPDFKILPFSAGKRKCPGAPLGVTMVLMALARLFHCFEWSSPGNI

DTVEVYGMTMPKAKPLRAIAKPRLAAHLYT*

>CYP703B1 BJ976025 Physcomitrella patens (moss)

MNILSPELLVPLITEWIQGGRLIFATCSVLVALLSSVFLVAHFRTPMNLPPGPKAMPLLG

NLLQMGSHPHRTMTAMHKKYGHILYIRLGCIPTVVVDSPQLIAEITKEQDNVFSSRPHMT

FTDIVAYDAHDFAMAPYGPHWRYVRRICVHELLTPKRLEITMKERIEESRCMIMAVAEAA

QKGEIVDMRDVFAGVSMTVMCRMLLGRREFAATGKKAKDFKHLIHELFRLMGALNLRDFV

PALGWLDLQGFERDMYKLRDEFDEVFDAVIQEHRDLASGKLPGGKPNDFISVLLDLPGEN

GAPHLDDKTIKAITPDMMAGATDTSAVTNEWAMAEIIRNTEIQRKLQEEIDSVVGLERNV

QESDINKLPYLMCVVKETFRLHPAGPFAIPRETMADTKLSGYRIPKGTRVLINIFSLGRS

SETWKDPLKFQPERWANENLSAIHDMGFRILPFGYGRRQCPGYNLGTTMVLLTLARLLHG

FKWSFPPGVTAENIDMEELYGCTTPLRTRLRTIATPRLAPHLYSQ*

>CYP703C1v2 Selaginella moellendorffii (lycopod moss)

MQLGPLPHRTMAGWCQKYGPLVYCRLGSTPTITASSPQMIRELLWTQDETFASRPRTTAG

KLMAYHDQDVGLAPYDAHWKLMRRICVENLLTTRRMEGFQKSRAEEARDLVETMLKEAKA

GNTINIREHLGGFTMNVITRMLIGKRYFGTESTGEKEATDIRELLHEAVALIGVFFIPDH

VPLLKWMDPKSYRHQFKKIGKRMDDYYSYIIEEHRKRQEVVDGPKDFVDVLLGENSELND

VEIKALIQDMVVGGTDTASFTMEWFMLEMIRNPKVMRKVQKEIDSVLEKKATNKHLVEES

DLASLDYLKAAVKETFRMHPVGGFLIPHESIRDTNVAGYHVPKGSLILINTHGLGRNSAV

WDNVDEFRPERFLRTDDKVHLRDSEYRVIPFGSGKRACPGAQLGQSMLLLGLGRLFHGFD

WYPPPGMSTGDIDVMEAYGLTTPPRTPLRAVAKARLDESFYCLH*

>CYP703D1v1 Selaginella moellendorffii (lycopod moss)

MDSSVFQAIVAAFFLFLSLIHFLFFRPIRPGAKSNVDPGVKGRDRRQRLHLPPGPKPWPV

IGNLLQIGPFPHKSMMEFTRRHGPLVYLKLGVVPTIVTDSPAIIRDILIKQDHIFASRPE

NIACQYFTYNGRDIAFAPYGQHWRAMRKICTLELLSPRKIASFRDGRCQELDLMVESVFQ

DLGREEGSSPTTHKINLRDKFASLSCNILTRMLLGKRHFGPGAAGPEDAAEHKQMIYEGF

ALVNAFNVADYLPFLRAFDLQGHERKMRRIMQRADEVYDEIIEEHRQKLAKNSGGSCQEQ

QGASFVDVLLSVPGANGEKQLSTTTIKAIMIDMLSAGTDTSSVISEWAMAELARHPREMA

KVREELDAAVGVDRPVDESDVVNLNYLKAVVKEIFRLHPVGAFLIPHFSTEDTRIGGYDI

PKNTRILVNTYSLGRSRSVWGDDVHLFRPDRFLASPGDLSSQIVELMDSECRVVPFGAGR

RSCPGASLGSCMVVMGLARLIQRFDWSAPPGEEIDVSERVGFTVLDKPLELVAKPRECVN

F*

>CYP703E1 old CYP789A1v1 Selaginella moellendorffii (lycopod moss)

MVEIPQLLSSSTPELYLKLAVAGSLLVLLLLLLNLPSSRGARRKSSSSSSSSGSSSSSSP

PLPPGPRGWPIIGNLLDVGTVPHEGMMKLTRAYGPLVYLRLGAIPHVVSDDPAIIKEFLK

IQDHIFASRPGNVILAELLTYGGKDIGFAPYGAHWRNMRKICTLELFSAKSVDSFQRLRR

MEMIHTLGLILDAAVDRRAVDLRDAFNGLTSNMMTRMLLGKRYFGPGDPGPEVGAELKAM

IAEGILMMNGFNISDYLPFLRFLDLQGQERRMKQIMRHIDGLATALLLELAPRIGKKPES

FVDILVNLRGENGEPHLPEDVMKAVMVDMMAAGTDTPGVSCEWAMAELLRDPALLARVRE

EVDRVVCVDRLVDESDLAHFRLLRAVLKESFRLHPVGAILIPHLAMEDAVVAGYGIPKDT

RVLINVFALNRNAQVWERPHEFDPERHLRGLGEGAVVEFGDPECRLIPFGSGRRMCPAAS

LGLTMVLLALANLVHAFDWEVPANLSMERAPGKMVKAQALTALARPRLPRHLYSQQI*

>CYP704A1 Arabidopsis thaliana

MAIIVVTTIFILLSFALYLTIRIFTGKSRNDKRYTPVHATIFDLFFHSHKLYDYETEIAR

TKPTFRFLSPGQSEIFTADPRNVEHILKTRFHNYSKGPVGTVNLADLLGHGIFAVDGEKW

KQQRKLVSFEFSTRVLRNFSYSVFRTSASKLVGFIAEFALSGKSFDFQDMLMKCTLDSIF

KVGFGVELGCLDGFSKEGEEFMKAFDEGNGATSSRVTDPFWKLKCFLNIGSESRLKKSIA

IIDKFVYSLITTKRKELSKEQNTSVREDILSKFLLESEKDPENMNDKYLRDIILNVMVAG

KDTTAASLSWFLYMLCKNPLVQEKIVQEIRDVTSSHEKTTDVNGFIESVTEEALAQMQYL

HAALSETMRLYPPVPEHMRCAENDDVLPDGHRVSKGDNIYYISYAMGRMTYIWGQDAEEF

KPERWLKDGVFQPESQFKFISFHAGPRICIGKDFAYRQMKIVSMALLHFFRFKMADENSK

VSYKKMLTLHVDGGLHLCAIPRTST*

>CYP704B1 Arabidopsis thaliana

MSLCLVIACMVTSWIFLHRWGQRNKSGPKTWPLVGAAIEQLTNFDRMHDWLVEYLYNSRT

VVVPMPFTTYTYIADPINVEYVLKTNFSNYPKGETYHSYMEVLLGDGIFNSDGELWRKQR

KTASFEFASKNLRDFSTVVFKEYSLKLFTILSQASFKEQQVDMQELLMRMTLDSICKVGF

GVEIGTLAPELPENHFAKAFDTANIIVTLRFIDPLWKMKKFLNIGSEALLGKSIKVVNDF

TYSVIRRRKAELLEAQISPTNNNNNNNNKVKHDILSRFIEISDDPDSKETEKSLRDIVLN

FVIAGRDTTATTLTWAIYMIMMNENVAEKLYSELQELEKESAEATNTSLHQYDTEDFNSF

NEKVTEFAGLLNYDSLGKLHYLHAVITETLRLYPAVPQDPKGVLEDDMLPNGTKVKAGGM

VTYVPYSMGRMEYNWGSDAALFKPERWLKDGVFQNASPFKFTAFQAGPRICLGKDSAYLQ

MKMAMAILCRFYKFHLVPNHPVKYRMMTILSMAHGLKVTVSRRS

>CYP704C1 Pinus taeda (Loblolly pine)

MDVNILTMFVTVSALALACSLWIASYLRNWRKKGVYPPVVGTMLNHAINFERLHDYHTDQ

AQRYKTFRVVYPTCSYVFTTDPVNVEHILKTNFANYDKGTFNYDIMKDLLGDGIFNVDGD

KWRQQRKLASSEFASKVLKDFSSGVFCNNAAKLANILAQAAKLNLSVEMQDLFMRSSLDS

ICKVVFGIDINSLSSSKAESGPEASFAKAFDVANAMVFHRHMVGSFWKVQRFFNVGSEAI

LRDNIKMVDDFLYKVIHFRRQEMFSAEKENVRPDILSRYIIISDKETDGKVSDKYLRDVI

LNFMVAARDTTAIALSWFIYMLCKHQHVQEKLLEEIISSTSVHEDQYSTECNDIASFAQS

LTDEALGKMHYLHASLSETLRLYPALPVDGKYVVNEDTLPDGFKVKKGDSVNFLPYAMGR

MSYLWGDDAKEFKPERWIQDGIFHPKSPFKFPAFQAGPRTCLGKDFAYLQMKIVAAVLVR

FFKFEAVKTKEVRYRTMLTLHMNEDGLNVQVTPRLNSD

>CYP704D1 Physcomitrella patens (moss)

MDFAQGWGSESIAGMMKTIVTAFSGILSLLLAYLLWAAADNWVLHRERKGPVQWPILGVT

LEALKNYQTLNDWVVYYFLRDGLTFSCKMMHLDLTFTADPVNVKHILKTNFANYDKRKFF

HENFEIFLGDGIFNVDGEIWRTQRKTASFEFASRKLRDFSTVVFRDYSVKLASILARAAT

AQQSMDMQDLFMRFTLDSIFKVTFDYDVGTLQPGLPNIPFAQAFEITNEITSSRLINPIW

KLNRALKIGSERVLLQSAKDVDEFIYGVIEAKKAEMANSKSDLFSRFMRLEEDDSDIQFT

DKNFRDTLLNFIIAGRDTTAVSLSWFVYRMAQNPEMTARLQQELRDFDTVRNWKQQPEGD

EGLRRRVLGFAELLTFDNLVKLQYLHACILETLRLHPAVPQDPKHAINDDILSDGTQIKK

GSLIYYTPYAMGRMPALWGPDAMEFNPQRWFVDGVVQTEQPFKFTAFQAGPRICLGKDSA

MLQLRMVLALLYRFFTFQIVEGTDIRYRQMATLLLANGLPAKIIKQKN*

>CYP704E1 Physcomitrella patens (moss)

MLSGGPLETFSVWFAMNGESSFPVKTCLSLTWVTAESFLVAAIAWSFAAWIWWHWREQRK

LPGPFAWPLIGCLPELSANWDRLHDWVLEQFSDDRRTIYVQFGYPDVAVFTVDPANVEHL

LKTNFSNYPKGESNCNLMRELFGVGIFTTDGELWKEQRRMASYEFSSASLRDFSTDVFRE

YALKLVFILSRFASTGADFDLQEMCMRMTLGTTCKIGFGVVLDCLSPSLPKIQFAQCFDD

ANFISYHRFVDPLWHVKRALNIGRERKLKHCVKVLNTFTYNVIEKRRQEMASFNTKVWSW

AAQSDLLSRLTDLCNRGGEISHYVDTALRDMILNFIVAGRDTTAGTLTWFFYMMSSHPEI

ADKIFDELSTVVAVAGKHIVEFSKLLTYEKLGKLHYLHAALSETLRLYPAVPLDSKQAAE

DDVLPDGTVVKKGSMVGYVPYSMGRMKCLWGDYAAEFKPERWIQEGEFVPQSLFKFTAFQ

AGPRTCLGKDSAYLQMKMTAALVMRFFTIRVVPGHSMQYRTMLTLNMKHGLRAVVSRR*

>CYP704F1 Physcomitrella patens (moss)

MGDEGATLFGAFKSGNVLPAGVGQQEVWIMAAVSLVVVTASMWLWLLSLRRRPPGPMIWP

WLGSMLEIAPQFDTMNDWYLNYFSADVKTFSFGMPGFPSCTKFVATVDPVIVEHILTNVY

KYGKGDQLRDRLGDFLGRGIFLADGEDWRRHRKIASTEFSTRKLRGHSASVFRGEGVKLA

NCLKVAMAADQPVEIQDLFLRMTLDSICKVAFGVEIGSLSPDLPDVQFAKDFDNAQAHIS

KRVVRPMFKILRALDIGEEHHFRIATNSVHSFAMDVIAKRRKEIAAAHDAGEEYHRDDLL

SKFMANLTQDENSYDDKELRDVIISFMLAGRDTTAVTLSWFTYEMCCHPEIADKIYEEGV

AVIGKHTVVESAVEHLTHEALGQMHYLHAALSESLRLHPAVPRDGKCVLEEDVLPNGIKV

KKGDFVQYVPYSMGRMPFLWGPDALEFKPERWLKDGVYQSVSPYIHSAFQAGPRICLGKD

SAYLQLKVTAALITHFFKFHLVPGQEIAYTTTLVMPIKKGLKVTLSPRQ*

>CYP704G1 Medicago truncatula

MDVLYTILTLIAFSLLAIFLAICFILMTIFKGKSIGDPKYAPVKGTVFNHLFYFNKLYDY

QAQMAKIHPTYRLLAPNQSDQLYTIDVRNIEHVLKTNFDKYSRGKYSQDVMTDLFGEGIF

AVDGDKWRQQRKVASYEFSTRVLRDFSCSVFRKNAAKLVRVISVFYHEGLVFDMQDLQMR

CALDSIFKVGFGTELNCLEGSSKEGTEFMKAFDESNALIYWRYVDPIWNLKRFLNIGGEA

KLKHNVKLIDDFVNGVINTKKEQLALQQDSNVKEDILSRFLMESKKGQTTITDKYLRDII

LNFMIAGKDTTANTLSWFFYMLCKNPIVEDKIVQEIRDVTCFHESELSNIDEFATNLTDS

ILDKMHYLHAALTETLRLYPVVPVDGRTADAPDILPDGHKLQKGDAVNYMAYAMGRMSSI

WGEDAEEFRPERWITDGIFQPESPFKFVAFHAGPRMCLGKDFAYRQMKIVAMCVLNFFKF

KLANGTQNVTYKVMFTLHLDKGLPLHAIPRS*

>CYP704H1v1 Selaginella moellendorffii (lycopod moss)

MASKLFSFLPSSSSPWLWKWSSSSQSFSSGAWTWIITWALILAWWIFLHRFRQRGLRGPK

SWPLVGCLFEQIANFDRLHHWLLDYHHKTWTFSAPVLGVNNTFTAHPANVEYILKTNFVN

YPKGELLRQRFRDMMGYGIFNVDGEMWMHQRKVATVEFASSKLRDYSTFAFRDLTLKLAG

ILADRSGTGQALDLQDLFLRLTLDSICKIGFGVEIGCLRPDLPLIPFAHAFDYGNTLIIR

RYIDMFWKIKRSFNAGSERELKRCIRVMDDFLYRVIERRRQELKQSKDVGRPDILSRFLS

LDEEEAYTDKMLRDVVINFVIAGRDTTALTLSWLFSELAKRPEVVEKILAEVDRVFGVDE

ELEGKDSMSKKEQVLAKVANFSRKLDYQGLNRLHYLQATLTEALRLYPAVPLETKTVVAD

DVLPDGFSVKGGQFVSYSSWAMGRLEEIWGPDVLEFKPDRWLRSDNIFQPQSPFKLTAFQ

ARREFGKAGPRICLGKDSAYLQMKITTILLLRFFKFELLDEKPVNYRMMVVLYMANGLLS

RVSFR*

>CYP704J1v1 Selaginella moellendorffii (lycopod moss)

MNSTDEVRNISGVFEGLSYPNPLVFITGIAAAVFLAVLLSAEKRDHKGPKRWPLVGSYFQ

VVKNFPVLHDWFLSYFSSECRTIAVDWGTFYNILTVDPANVEHILKTNFANYPKGRVSHA

RNYDFMGDGIFNSDGEMWKRHRKLASYEFSSKKVNEYSGQVFRKAAVRMMEVLENIASKK

TSFDFQDISMRMTLDSICEVAFGVELNTLSPSLPAVPFAASFDRVNELIVRRLIGPVWKI

LRALNLGSERELKNQIQVLDSFTFQVIENRRQEIEACEKSGKEYERQDLLSRFMTSTGAS

DAYHDRELRDAILNFIIAGRDTTAITLSWFIYCICNNPRVAKEIRLELDRTFGSESNTLT

FSAFAQLLSTENLRTLHYLHACISETLRLYPPVPRDGKYAANDDVLPDGTKVKRGDSVAY

VQYSMGRMEFLWGPDALEFKPERWIKNSEYQPQSPFVYTAFQAGPRICLGKDAAYLQAKI

TAAMLMRFFNFELVKDHVVHYRLLMVLAMVNGIKVNVSTL*

>CYP705A1 Arabidopsis thaliana

MDAIVVDSQNCFIIILLCSFSLISYFVFFKKPKVNFDLLPSPPSLPIIGHLHLLLSTLIH

KSLQKLSSKYGPLLHLRIFNIPFILVSSDSLAYEIFRDHDVNVSSRGVGAIDESLAYGSS

GFIQAPYGDYWKFMKKLIATKLLGPQPLVRSQDFRSEELERFYKRLFDKAMKKESVMIHK

EASRFVNNSLYKMCTGRSFSVENNEVERIMELTADLGALSQKFFVSKMFRKLLEKLGISL

FKTEIMVVSRRFSELVERILIEYEEKMDGHQGTQFMDALLAAYRDENTEYKITRSHIKSL

LTEFFIGAADASSIAIQWAMADIINNREILEKLREEIDSVVGKTRLVQETDLPNLPYLQA

VVKEGLRLHPPTPLVVREFQEGCEIGGFFVPKNTTLIVNSYAMMRDPDSWQDPDEFKPER

FLASLSREEDKKEKILNFLPFGSGRRMCPGSNLGYIFVGTAIGMMVQCFDWEINGDKINM

EEATGGFLITMAHPLTCTPIPLPRTQNSLISHL*

>CYP706A1 Arabidopsis thaliana

METASSNFSLSQILNIEEPYSSVMLGVAALLAVVCYFWIQGKSKSKNGPPLPPGPWPLPI

VGNLPFLNSDVLHTQFQALTLKHGPLMKIHLGSKLAIVVSSPDMAREVLKTHDITFANHD

LPEVGKINTYGGEDILWSPYGTHWRRLRKLCVMKMFTTPTLEASYSTRREETRQTIVHMS

EMAREGSPVNLGEQIFLSIFNVVTRMMWGATVEGDERTSLGNELKTLISDISDIEGIQNY

SDFFPLFSRFDFQGLVKQMKGHVKKLDLLFDRVMESHVKMVGKKSEEEEDFLQYLLRVKD

DDEKAPLSMTHVKSLLMDMVLGGVDTSVNASEFAMAEIVSRPEVLNKIRLELDQVVGKDN

IVEESHLPKLPYLQAVMKETLRLHPTLPLLVPHRNSETSVVAGYTVPKDSKIFINVWAIH

RDPKNWDEPNEFKPERFLENSLDFNGGDFKYLPFGSGRRICAAINMAERLVLFNIASLLH

SFDWKAPQGQKFEVEEKFGLVLKLKSPLVAIPVPRLSDPKLYTA

>CYP706B1 Gossypium arboreum (cotton, Malvales)

MLQIAFSSYSWLLTASNQKDGMLFPVALSFLVAILGISLWHVWTIRKPKKDIAPLPPGPR

GLPIVGYLPYLGTDNLHLVFTDLAAAYGPIYKLWLGNKLCVVISSAPLAKEVVRDNDITF

SERDPPVCAKIITFGLNDIVFDSYSSPDWRMKRKVLVREMLSHSSIKACYGLRREQVLKG

VQNVAQSAGKPIDFGETAFLTSINAMMSMLWGGKQGGERKGADVWGQFRDLITELMVILG

KPNVSDIFPVLARFDIQGLEKEMTKIVNSFDKLFNSMIEERENFSNKLSKEDGNTETKDF

LQLLLDLKQKNDSGISITMNQVKALLMDIVVGGTDTTSTMMEWTMAELIANPEAMKKVKQ

EIDDVVGSDGAVDETHLPKLRYLDAAVKETFRLHPPMPLLVPRCPGDSSNVGGYSVPKGT

RVFLNIWCIQRDPQLWENPLEFKPERFLTDHEKLDYLGNDSRYMPFGSGRRMCAGVSLGE

KMLYSSLAAMIHAYDWNLADGEENDLIGLFGIIMKKKKPLILVPTPRPSNLQHYMK

>CYP706C9 Zea mays (maize)

MDAPGASTTTLLLYGALLLVGFLFIAVARRSNAGLPPGPTGLPLLGSLPSLDPQLHVYFA

RLAARYGPIFSIRLGSKLGVVVTSPELAREVLREQDLVFSGRDVPDAARSISYGGGQNIV

WNPVGPTWRLLRRVCVREMLGPAGLDNVQGLRAREFGATLAHLHAQARAAAPVDVGAQMF

LTVMNVITGTLWGGNVGSEGERVALGREFRHLVAEITDMLGAPNVSDFFPALARFDLQGI

RKKSDALRERFNQMFARIIEQRVHAERAGGEPPAPDFLEYMLQLEKEGGDGKASFTMTNV

KALLMDMVVGGTETTSNTVEWAMAELMQKPELLAKVRQELDAVVGRDAVVEESHLPQLPY

LHAVVKETLRLHPALPLMVPHCPSADATVGGYRVPAGCRVFVNVWAIMRDPAVWKDPRDF

VPERFLDGAGEGRKWDFTGSEMEYLPFGSGRRICAGVAMAQRMTAYSLAMLLQAFDWELP

AGARLELDEKFAIVMKKATPLVAVPTPRLSKPELYYSA*

>CYP706D1 Populus trichocarpus (cottonwood)

MSSSTICGPWSWFCKGDQDNEDILLPIILLAVSVTILGTCLFQWGFKKQRETADKLPPGP

RGLPIVGYLPFLGPNLHQLFMELAQTYGPIYKLSIGRKLCVIISSPALVKEVVRDQDITF

ANRNPTIAAKTFSYGGKDIAFQPYGPEWRMLRKILLREMQSNANLDAFYSLRRNKVKESV

NETYRKIGKPVNIGELAFSTVISMISGMFWGGTLEVDTEIDIGSEFRAAASELIEILGKP

NVSDFFPVLARFDIQGIERKMKKATQRIEKIYDFVMDEWIEKGSARVESEAKNDQRKDFM

HFLLGFKEQDSRRSISREQIKALLMDIVVGGTDTTSTTVEWAMAEMMLHPEVMKNAQKEL

TDAVGTDEIVEERHIDKLQFLHAVVKETLRLHPVAPLLLPRSPSNTCCVGGYTIPRNAKV

FLNVWAIHRDPKFWDNPSEFQPERFLSDVSRLDYLGNNMQYLPFGSGRRICAGLPLGERM

LMYCLATFLHMFKWELPNGERADTSEKFGVVLEKSTPLIAIPTPRLSNLNLYA*

>CYP706E1 Artemisia annua (sweet wormwood, Asterales)

MILVTDFKDKIYYTMINYWSWWWEVDNESDNVARTILTILVPILVLLWYKWTVSYTKKPR

SRLPPGPYGLPVIGYLPFLSSNLHERFTEMSHKYGPIFSLYLGSKLNVVVNSIDLAKVVA

RDLDQTFANRNPPVTAITVTYGLLDIAWSNNNTHWRNTRKLLVSQVLSNANLDACQGFRT

DEVRKTVGHVYAKIGEIVDINEISFETELNVVTNMLWGRNESGSLLEGFREVELKMLELL

GAPNISDFIPMLSWFDLQGRKREMQKQHERLDRILDNVIKARMEGVLHDDGKKDFLQIML

ELKDQKDGPTSLNMVQIKALLFDILTASTDTTSTMVEWVMAEILHNPDVMRRVQEELTIV

IGMNNIVEESHLQKLVYLDAVVKETFRVHPPLPLLIQRCPNESFTVGGYTIPKGSIVYIN

VMAIHHDPKNWINPLEFKPERFLNGKWDYNGYNLKYLPFGSGRRICPGIPLGEKMLMYIL

ASLLHSFEWSLPKEEELELSEEFGFVTKKRRPLIAIPSQRLPEASLYS

>CYP706F2 Carica papaya

MMLEGWSLWMEAEHTTIMVSILVATVTIFVGYKWLMKIAMRGKPPLPPGPRGLPLLGNLP

FVEPDLHIYFSKLSKKFGPIFKIQMGTKIYVVINSASLARKVLKEDDEIFANRDPPAAAI

AETYGGGNILWRPNGPEWRNLRKVLIREMMSKTYFDASYGLRRREVREMVKEIYAKVGSP

IKVRNHMFLISLNVVMGMLWGAPLDEDKKHNVGLELVPFIEEAVDLLGKPNISDFFPILA

PLDLQGIVSKMLKIRLRFDNIFESVIAIKKSTKTSNQSKDFLQILLELMQQEDEKMLFSM

TNIKALLLDIISATTETSSTTVEWVMTELLKNSDIMKKVHEELERVVGNEKIVEECDINQ

LHYLQSVVKETMRLHPVGPLLIPHSPSVSTTIAGYTIPKGSSVFINVWSIMRDHETWKNP

LKFQPERFFEHPEIGDYRGNNFNYLPFGSGRRICAGINLAEKMVMNVLATLLHSFDWKVE

NATNLNSSEKFGIVVRKLDPLMAIPTIRLPTLEQYY*

>CYP706G1 Vitis vinifera

MISYHLNKATETICSLWSWWWAGSNKKLDVVAGAVLTVSVAVLAILWYLRTSRKGTAPWP

PGPRGLPVVGYLPFLGSNLHHSFAELAHLYGPIFKLWLGNKLCVVLSSPSLAKQVVRDQD

IIFANRDPPVAAFAYTYGGLDIAWSPYGSYWRNLRKVFVREMLSNTSLEACYPLQRSEVR

KAITNAYSKIGTPMDIGQLSFQTVVNVTLSSLWGNTPEAHNDGKIGAEFREAASEITELL

GKPNISDFFPTLAGFDIQGVERQMKRAFLSAEQVIDSIIDRKMKKSTAKEEGASDNGEKK

DFLQFLLDLKVQEDTETPITMTQIKALLMDILVGGTDTTATMVEWVMAEMIRNPVIMTRA

QEELTNVVGMGSIVEESHLPKLQYMDAVIKESLRLHPALPLLVPKCPSQDCTVGGYTIAK

GTKVFLNVWAIHRDPQIWDSPSEFKPERFLSEPGRWDYTGNNFQYLPFGSGRRICAGIPL

AERMIIYLLASLLHSFNWQLPEGEDLDLSEKFGIVLKKRTPLVAIPTKRLSSSDLYL*

>CYP706H1 Vitis vinifera

MSSMVHRLLAMASSFWSQWCNIGYGGAGLFVSFAVIAVGAISWHILRKESLALPPGPRGM

PVLGNLPFLHPDLHSCFAKMAQKYGPVMRLWLGNKLTVVLSSPSLAKEVLRDNDAIFADR

DTPIAMLTMTYGGSGLIWARCDQNWRMLRKVWVGEMLSKVSLDRLYALRHREVWDSMRRI

YANAGTSVNVNEHTFSAMINVITSMLWGRTLEGEERRHADKEFRQVVWEMFDLLTKPDVS

DLFPVLAPLDIQGKNSKMKKLGSRLDRIFDFIINHHEVKGLSMEGIENKDREHKDLLQIF

LQNEEGSKGILDKTQLKALFLDMVAAGTDTASSAVEWAMAELMNKPEKMERAQKELEQVV

GMNNMVEETHLPKLPFLNAVVKEVLRLHPPGPFLVPRRTREPCVLRGYTIPSGTQVLVNA

WAIHRDPEFWDSPSEFQPERFLSGSLKCDYSGNDFRYLPFGSGRRICAGVPLAERIVPHI

LASMLHLFDWRLPDGVNGVDLTEKFGLVLRKATPFVAIPKPRLSNLDLYT*

>CYP706J1 Vitis vinifera CAN80272.1

MIAVLLRNLVNLVSEGWSWWWDGLTENTIFRVVAALSTAAIAISWYAWLIKKASRGLPPL

PPGPRGLPLLGNLLFIEPDLHRYFSKLSQLYGPIFKLQLGSKTCIVISSSSVAKEILKDH

DVIFANRDVPISALALTYGGQDIAWSHYSPEWRKLRKVFVQEMMSSASLDACSALRRREV

QEMVRDVYGKVGTPINMGDQMFLTVLNVVTSMLWGGTLHGEDRSRIGMEFRRVIVEMVGL

MGKPNISDLFPALAWFDLQGIESRVKKLVLWFDRIFESLIAQRTQLDGADGGGKNKSKES

KDFLQFMLELMHQGDDKTSVSITQLKALFMDIVVAATDTSSTTVEWAMAELLQHPQTMQK

AQEELEKVVGNENIVEESHLFQLPYLGAVIKETLRLHPPLPLLVPHSPSTSCIISGYTIP

KGSRILFNAWAMQRNPEVWEHPLEFIPERFLEDAASADYKGNNFNFMPFGSGRRICAGLP

LAEKMLLYVLASLLHSFDWKLPDGRTSVDLEERFGIVLKKSEPLLAIPTARLSN*GHYDP

SERIF

>CYP706K1 Glycine max (soybean, Fabales)

MLLSSFCVTWPYLWCWDSTSLHPILTILVTLISVLCLLRWFKNSSYEATLPSPLPPGPLG

LPLLGYLPFLGTNPHLKFHKLAQVYGPIYKLMLGTKTFIVVSSPSLVKEIVRDQDTVFAN

RDPPISVLVALYGGTDIASLPLGPRWRKARKIFVSEMLSNTNISSSFSHRKIEVKKSIRD

VYEKKIGCPISISELAFLTATNAIMSMIWGETLQGEEGAAIGAKFRAFVSELMVLVGKPN

VSDLYPALAWLDLQGIETRTRKVSQWIDKFFDSAIEKRMNGTGEGENKSKKKDLLQYLLE

LTKSDSDSASMTMNEIKAILIDIVVGGTETTSTTLEWVVARLLQHPEAMKRVHEELDEAI

GLDNCIELESQLSKLQHLEAVIKETLRLHPPLPFLIPRCPSQTSTVGGYTIPKGAQVMLN

VWTIHRDPDIWEDALEFRPERFLSDAGKLDYWGGNKFEYLPFGSGRRICAGLPLAEKMMM

FMLASFLHSFEWRLPSGTELEFSGKFGVVVKKMKPLVVIPKPRLSKPELYQ*

>CYP707A1 Arabidopsis thaliana

MDISALFLTLFAGSLFLYFLRCLISQRRFGSSKLPLPPGTMGWPYVGETFQLYSQDPNVF

FQSKQKRYGSVFKTHVLGCPCVMISSPEAAKFVLVTKSHLFKPTFPASKERMLGKQAIFF

HQGDYHAKLRKLVLRAFMPESIRNMVPDIESIAQDSLRSWEGTMINTYQEMKTYTFNVAL

LSIFGKDEVLYREDLKRCYYILEKGYNSMPVNLPGTLFHKSMKARKELSQILARILSERR

QNGSSHNDLLGSFMGDKEELTDEQIADNIIGVIFAARDTTASVMSWILKYLAENPNVLEA

VTEEQMAIRKDKEEGESLTWGDTKKMPLTSRVIQETLRVASILSFTFREAVEDVEYEGYL

IPKGWKVLPLFRNIHHSADIFSNPGKFDPSRFEVAPKPNTFMPFGNGTHSCPGNELAKLE

MSIMIHHLTTKYRCVCVYYLLITFSFTHFVLFFQIYV

>CYP707B2v2 Selaginella mollendorffii (Lycopod moss)

MWDALSQWGILISLARAWRREKNAKLPRGNWWLPWLGESLDFFWRSPDDFYKTRFSRYGS

IFLSHIFGSTTIVTSTPEEAKFILGTRHKLFRAKYPTSIDRVLNHPFWEGDFHCRVRKIV

QAPMMPEVLKSQISKFDSLATWTLNTWSHGDHVITHAETRKFSFHVALYLVCSLEPSAES

MKMLDDYECVAKGAICFPLNVPGTGFHLALKKSKVILEALDNIIARRRMERSVHNDILSS

LLNSSDENGIKLTTDQVKNVLITLLFAGHETTGVLLVWIVKYLTENPQVLHLVKEEQEIV

RQSMADDKQPLTWANVRNMPYTLKVVQETLRLANVAPFSPREILEDVEYNGILFPKGWRV

QVYYRHFHLNPEYYKEPLKFDPSRFEVPPKPMVYTPFGNGIRLCPGSELVKLEVLIFIHR

LVTNYSWHAVGADKGIQYWPTPRPKGGYKIKVHSHAHSSFSQEQL*

>CYP707C1v1 Selaginella mollendorffii (Lycopod moss)

MVKQSRVAVEVSSTSGAKTPPGPAWRIPLVGETLSFLRDPHRFYLTRIARYGEIFSTSLF

GDKCIIVTTPEASKWLLQSAQKFFKPAYPESANSLIDPTRSFGSEQLHNYVRRIVGSSLY

PESLQSHIPAIEALACSVLDSWTKQKSVNVYSEMAKYTFEVAMKILCGMEPGKQMDALFQ

NMQDFEKAFLTLNINLPFTTYRRGLKARDSMFKAVEEMIQQRRKKKRDWSGREQQQRLDM

LDSMICVETKDEKFANAVTDIHVRGIIMTILFAGHETSAAQLVWAIKNLHDNPELLHGVK

EEHEAIRRKREPGSPLTWSQVMKEMPLTLRVINETMRTSYVGLFLPREALDDLEYDGYYF

PKGWKVYASPSMVHLNPKLYTEPYKFDPTRFQDGGPKPNTFIPFGNGQRLCLGGELAKVE

MLVLIHHLVTTYSWKIKEDHGGIRWWPVPIPKGGLVIQVEREVERGLEKETRQGQTGELG

SGEFQH*

>CYP708A1 Arabidopsis thaliana

MNNLISLDIMKEMWGVALSFVIALVVVKISLWLYRWANPNCSGKLPPGSMGFPVIGETVE

FFKPYSFNEIHPFVKKRMFKHGGSLFRTNILGSKTIVSTDPEVNFEILKQENRCFIMSYP

EALVRIFGKDNLFFKQGKDFHRYMRHIALQLLGPECLKQRFIQQIDIATSEHLKSVSFQG

VVDVKDTSGRLILEQMILMIISNIKPETKSKLIESFRDFSFDLVRSPFDPSFWNALYNGL

MVRSARSNVMKMLKRMFKERREEATSDDSKYGDFMETMIYEVEKEGDTINEERSVELILS

LLIASYETTSTMTALTVKFIAENPKVLMELKREHETILQNRADKESGVTWKEYRSMMNFT

HMVINESLRLGSLSPAMFRKAVNDVEIKGYTIPAGWIVLVVPSLLHYDPQIYEQPCEFNP

WRWEGKELLSGSKTFMAFGGGARLCAGAEFARLQMAIFLHHLVT*TYDFSLIDKSYIIRA

PLLRFSKPIRITISENPLSSSHQNANLF*

>CYP709B1 Arabidopsis thaliana

MGLVIFLALIVLILIIGLRIFKAFMILVWHPFVLTRRLKNQGISGPNYRIFYGNLSEIKK

MKRESHLSILDPSSNDIFPRILPHYQKWMSQYGETFLYWNGTEPRICISDPELAKTMLSN

KLGFFVKSKARPEAVKLVGSKGLVFIEGADWVRHRRILNPAFSIDRLKIMTTVMVDCTLK

MLEEWRKESTKEETEHPKIKKEMNEEFQRLTADIIATSAFGSSYVEGIEVFRSQMELKRC

YTTSLNQVSIPGTQYLPTPSNIRVWKLERKMDNSIKRIISSRLQSKSDYGDDLLGILLKA

YNTEGKERKMSIEEIIHECRTFFFGGHETTSNLLAWTTMLLSLHQDWQEKLREEIFKECG

KEKTPDSETFSKLKLMNMVIMESLRLYGPVSALAREASVNIKLGDLEIPKGTTVVIPLLK

MHSDKTLWGSDADKFNPMRFANGVSRAANHPNALLAFSVGPRACIGQNFVMIEAKTVLTM

ILQRFRFISLCDEYKHTPVDNVTIQPQYGLPVMLQPLED*

>CYP709C1 Triticum aestivum cultivar Darius

MGLVWMVAAAVAAVLASWAFDALVYLVWRPRAITRQLRAQGVGGPGYRFFAGNLAEIKQL

RADSAGAALDIGDHDFVPRVQPHFRKWIPIHGRTFLYWFGAKPTLCIADVNVVKQVLSDR

GGLYPKSIGNPHIARLLGKGLVLTDGDDWKRHRKVVHPAFNMDKLKMMTVTMSDCAGSMM

SEWKAKMDKGGSVEIDLSSQFEELTADVISHTAFGSSYEQGKKVFLAQRELQFLAFSTVF

NVQIPSFRYLPTEKNLKIWKLDKEVRTMLMNIIKGRLATKDTMGYGNDLLGLMLEACAPE

DGQNPLLSMDEIIDECKTFFFAGHDTSSHLLTWTMFLLSTHPEWQEKLREEVLRECGNGI

PTGDMLNKLQLVNMFLLETLRLYAPVSAIQRKAGSDLEVGGIKVTEGTFLTIPIATIHRD

KEVWGEDANKFKPMRFENGVTRAGKHPNALLSFSSGPRSCIGQNFAMIEAKAVIAVILQR

FSFSLSPKYVHAPMDVITLRPKFGLPMILKSLEM

>CYP709D1 rice

MLKSTIELYIFTTAIAKKSLHSQTKHKSKMDVPSVVIPILVVLVSRLLTSALVHLLWKPY

AITKLFRGQGITGPKYRLFVGSLPEIKRMKAAAAADEVAAGAHSHDFIPIVLPQHSKWAT

DHGKTFLYWLGAVPAVSLGRVEQVKQVLLERTGSFTKNYMNANLEALLGKGLILANGEDW

ERHRKVVHPAFNHDKLKFMSVVMAESVESMVQRWQSQIQQAGNNQVELDLSRELSELTSD

VITRSAFGSSHEEGKEVYQAQKELQELAFSSSLDVPALVFLRGNTRAHQLVKKSRTMLME

IIEGRLAKVEAAEAGYGSDLLGLMLEARALEREGNGLVLTTQEIIDECKTFFFAGQDTTS

NHLVWTMFLLSSNAQWQDKLREEVLTVCGDAIPTPDMANRLKLVNMVLLESLRLYSPVVI

IRRIAGSDIDLGNLKIPKGTVLSIPIAKIHRDRDVWGPDADEFNPARFKNGVSRAASYPN

ALLSFSQGPRGCIGQTFAMLESQIAIAMILQRFEFRLSPSYVHAPMEAITLRPRFGLPVV

LRNLQG*

>CYP709E1 rice

MATLLLLAVAAAAAAWVWWGRYAWRARAVARRLAAQGVRGPRRGGLLRGCNDEVRRRKAE

AEADGVAMDVGDHDYLRRVVPHFVAWKELYGTPFLYWFGPQPRICVSDYNLVKQILSKKY

GHFVKNDAHPAILSMIGKGLVLVEGADWVRHRRVLTPAFTMDKLKVMTKTMASCAECLIQ

GWLDHASNSKSIEIEVEFSKQFQDLTADVICRTAFGSNSEKGKEVFHAQKQLQAIAIATI

LNLQLPGFKYLPTKRNRCKWKLENKLRNTLMQIIQSRITSEGNGYGDDLLGVMLNACFST

EQGEKRDELILCVDEIIDECKTFFFAGHETTSHLLTWTMFLLSVYPEWQDRLREEVLREC

RKENPNADMLSKLKEMTMVLLETLRLYPPVIFMFRKPITDMQLGRLHLPRGTAIVIPIPI

LHRDKEVWGDDADEFNPLRFANGVTRAAKIPHAHLGFSIGPRSCIGQNFAMLEAKLVMAM

ILQKFSFALSPKYVHAPADLITLQPKFGLPILLKALDA*143643

>CYP709F1 Populus trichocarpus (cottonwood)

MMVYTYVGYFAAGLAVQVLVAKILKLCWIVLWRPYALIKSFEKQGIKGPSYSILHGTLPE

MKTLLKAANEVILDTNCHDIAQRVQPHYNRWSAEYGEVFLFWRGVQPAIRIADPKLAKQI

LSDKSGAYAQPQFDHRLLSFAGNGVGQLNGPDWVRHRSILTPAFTKDKLKLMTKRMASCT

IDMIDDWKNRARIADHQNITIEMSEEFKKLTCDVITHTAFGSNYVEGGEVFKAQDELIHH

CVATMADLYIPGSRFLPTPSNRQMWKMENNVNNSLRRLIQGRLESAQARGNLDGCYGDDV

LGLLVEASKTTNKSLKLTMDEIIDECKQFFFSGHETTAKLLTWTIFLLSLHQEWQERLRE

EVLTECGMGIPDADMVSKLKLLNMVLLETLRLYCPVLETLRETSRATKLGDFLIPKGVFI

TIQLVQLHRSKEYWGEDANDFNPLRFKNGVSQAAKHPNAFLGFGMGPRTCLGQNFAMLEV

KLVLSLLLQRFSFFLSPEYKHAPANYLTMEAQYGVPTIVKPLLSK*

>CYP709G1Picea sitchensis (Sitka spruce)

MDCVIRSSVEAFVAILVVLFGSIVVKLFRDLIWRPYAFHKAYAGQGIRGTPYRILAGSVP

EYTELLREAHAQPMQNISHDIVPRITPEYHKWCQIYGEPFFYWYGIHSRLYISEPELIKE

VLSNKFGHYDKPTPRPILLALLGRGLVFADGLRWVKHRRIVSPVFNVDKLKPMVKKMAAC

TSSMLENWQEMMAQADSHGKEIDVHHDFRALTADIISHTAFGSSCNEGKEVFELQRQLQE

MAAKAEQSVFIPGSQYIPTRKNSHAWKIDRRVKEILNSIIQSRLEPRTTTRAHVGYGSDL

LGIMMTANQKELGGSQRNLSMTIDEIMNECKTFFFAGHDTTSNLLTWAVFLLSINPEWQE

ILRKEVISVCGTDIPDADMLSKMKSMTMVLNETLRLYPPASKIIRKAYKAIKLGQFSLPK

GAVLSFSILAMHHNEKFWGLDANLFKPERFAAGVSKAAIHPNAFFPFSLGPRNCVGQNFA

MLEAKTVLAMILQRLSFSLSPAYKHAPIAVLTLQPQYGMQIIFKSIEVQT*

>CYP709H1 Zea mays

MVVAVQLAALLALLLALWRLVWRPHAVARSFARQGIRGPPYTFLAGSLPEAKRLLMAGRR

GTAPLDAGCHDIMPVLLPQFLRWVADYGRTLLFWIGPIPAVLSTDLQLIKQVLTDRTGLY

QKDFMIPVLKFLFGNGVILINGDDWKRHRKVVLPAFNHETIKSMSAVTAEVTEQMMQQWR

GQIHGSEEESAEIDMIHAFNDLTSKVNGRVAFGTSHREVEEVIVLMREMQKLATAATLDA

PILWYLPTRRNLHVRRLNKQLRSKIMSIMQARLAADGADRRGGRGGAVSGGGDLLGLLLE

AWTPQPQQHGNGGETLTTDEVIDECKTFFAAGQETTATLLVWAMFLLAVHPEWQDKVREE

VVREFCTGDDGEVPHADVLAKLKLLYMVLLETSRLYPPIVYIQRRAAWDAVLGGIKVPQG

TVISIPIAMLHRDKQVWGPDADEFNPMRFEHGLTKAAKDPKALLSFSLGPRVCTGQSFGI

VEVQVVMAMILRRFSFSLSPKYVHKPKYLLSLTPKLGMPLIVRNVDG*

>CYP710A1 Arabidopsis thaliana

MVFSVSIFASLAPYLISAFLLFLLVEQLSYLFKKRNIPGPFFVPPIIGNAVALVRDPTSF

WDKQSSTANISGLSANYLIGKFIVYIRDTELSHQIFSNVRPDAFHLIGHPFGKKLFGDHN

LIYMFGEDHKSVRRQLAPNFTPKALSTYSALQQLVILRHLRQWEGSTSGGSRPVSLRQLV

RELNLETSQTVFVGPYLDKEAKNRFRTDYNLFNLGSMALPIDLPGFAFGEARRAVKRLGE

TLGICAGKSKARMAAGEEPACLIDFWMQAIVAENPQPPHSGDEEIGGLLFDFLFAAQDAS

TSSLLWAVTLLDSEPEVLNRVREEVAKIWSPESNALITVDQLAEMKYTRSVAREVIRYRP

PATMVPHVAAIDFPLTETYTIPKGTIVFPSVFDSSFQGFTEPDRFDPDRFSETRQEDQVF

KRNFLAFGWGPHQCVGQRYALNHLVLFIAMFSSLLDFKRLRSDGCDEIVYCPTISPKDGC

TVFLSRRVAKYPNFS*

>CYP710B1 Chlamydomonas reinhardtii (green algae)

MNATGLLNDGLASLGMSGFGDNLASGPALVAAGGALALGYALWEQMKFRWYRSDKNGNML

PGPASVTPIIGGIVEMVKDPYGFWERQRLYSFPGMSWNSIVGIFTVFVTDPALSRYVFSH

NSSDSLLLALHPNAEWILGKTNIAFMSGPEHKALRKSFLALFTRKALGLYVLKQDDVIRK

HFNEWMQTAGPREIRPFIRDLNAYTSQEVFVGPYLDDPTEREKFSDAYRAMTDGFLAFPL

LLPGTGVWKGRQGRQFIVKTLTRAAARSKVRMAAGQEPECLLDFWTKQILSDIKDAADAG

QEAPFYADDKKIAETVMDFLFASQDASTASLVWTITLMAEHPEVLARVRDEQYRLRPNPE

EKVTGDMLNEMHYTRQVVKEILRFRPAAPMVPMRAKAPFKLTETYTAPKGALIVPSLVAA

CKQGYSNPDSFDPDRFSPERAEDIKYASNFLVFGHGPHYCVGKEYAMNHLTVFLALLATS

LDFPRIRSKVSDDIIYLPTLYPGDSIFDLSWSAKK*

>CYP711 Arabidopsis thaliana

MKTQHQWWEVLDPFLTQHEALIAFLTFAAVVIVIYLYRPSWSVCNVPGPTAMPLVGHLPL

MAKYGPDVFSVLAKQYGPIFRFQMGRQPLIIIAEAELCREVGIKKFKDLPNRSIPSPISA

SPLHKKGLFFTRDKRWSKMRNTILSLYQPSHLTSLIPTMHSFITSATHNLDSKPRDIVFS

NLFLKLTTDIIGQAAFGVDFGLSGKKPIKDVEVTDFINQHVYSTTQLKMDLSGSLSIILG

LLIPILQEPFRQVLKRIPGTMDWRVEKTNARLSGQLNEIVSKRAKEAETDSKDFLSLILK

ARESDPFAKNIFTSDYISAVTYEHLLAGSATTAFTLSSVLYLVSGHLDVEKRLLQEIDGF

GNRDLIPTAHDLQHKFPYLDQVIKEAMRFYMVSPLVARETAKEVEIGGYLLPKGTWVWLA

LGVLAKDPKNFPEPEKFKPERFDPNGEEEKHRHPYAFIPFGIGPRACVGQRFALQEIKLT

LLHLYRNYIFRHSLEMEIPLQLDYGIILSFKNGVKLRTIKRF*

>CYP712A1 Arabidopsis thaliana

MDLKLNTKLIILITSLAFPFMLYALFKWFLKEQGSLAATKLPQSPPALPFIGHLHLIGKV

LPVSFQSLAHKYGPLMEIRLGASKCVVVSSSSVAREIFKEQELNFSSRPEFGSAEYFKYR

GSRFVLAQYGDYWRFMKKLCMTKLLAVPQLEKFADIREEEKLKLVDSVAKCCREGLPCDL

SSQFIKYTNNVICRMAMSTRCSGTDNEAEEIRELVKKSLELAGKISVGDVLGPLKVMDFS

GNGKKLVAVMEKYDLLVERIMKEREAKAKKKDGTRKDILDILLETYRDPTAEMKITRNDM

KSFLLDVFMAGTDTSAAAMQWAMGQLINHPQAFNKLREEINNVVGSKRLVKESDVPNLPY

LRAVLRETLRLHPSAPLIIRECAEDCQVNGCLVKSKTRVLVNVYAIMRDSELWADADRFI

PERFLESSEEKIGEHQMQFKGQNFRYLPFGSGRRGCPGASLAMNVMHIGVGSLVQRFDWK

SVDGQKVDLSQGSGFSAEMARPLVCNPVDHFNTF

>CYP712B1 Arabidopsis thaliana (thale cress)

MFTLNLDMNYTSCSYLFFTLVTIFLLHRLFSSSSRRGLPPGPRGLPVLGHMHLLRSSLPR

SLQALAHTYGPLMTIRIGSLRVLVVSDSDTAKLILKTHDPDFASKFVFGPRQFNVYKGSE

FFNAPYGSYWRFMKKLCMTKLFAGYQLDRFVDIREEETLALLSTLVERSRNGEACDLGLE

FTALTTKILSKMVMGKRCRQNSNIPKEIRKIVSDIMACATRFGFMELFGPLRDLDLFGNG

KKLRSSIWRYDELVEKILKEYENDKSNEEEEKDKDIVDILLDTYNDPKAELRLTMNQIKF

FILELFMASLDTTSAALQWTMTELINHPDIFAKIRDEIKSVVGTTNRLIKESDLQKLPYL

QAAIKETLRLHPVGPLLRRESNTDMKINGYDVKSGTKIFINAYGIMRDPTTYKDPDKFMP

ERFLVVEQDTERKMGYYQQYMLELKGQDVNYLAFGSGRRGCLGASHASLVLSLTIGSLVQ

CFNWTVKGDEDKIKIKLPTGFSASGTAGGSSLMCSPELCFDPFGYQTK*

>CYP712C1 Populus trichocarpus (cottonwood)

MAPSDHSFAYYCCLCITWSTIIIIVVHLFIKTCTSFCNKTRHPPSPLGLPIIGHLHLLSS

DLPNSLKTLASRYGPLMKIRFGSTPIYVVSDAKTAKEILKIHDVDFASKYTLGFGLSKFD

IYDGYTFFNAPYGTYWRFMKKLCMTKLFRGPQLDRFVHIREQETLKLLKSLVDKSREGKP

CDLGEELSVFSSNIICRMVIGNICVEDPNLPIEIRKLVGDIMENAAKFSFNEVFGPLNRF

DLLGKGKRLVSATRKYDKLLEQLMKKYEDNFDKLINSGDEEQKDVMIILMEAYKDTNAEL

KLTRTHIKKFFLEIFFAGVETTATAMQSAITELINNPKAFMKLREEIHSVFGSNYRLLKE

SDVPKLPFLQAVVKETLRLNPIATLRARQCDVDTRINGYDIKAGTRILINAYAIMRDSDS

WEKPDDFFPERFLADSMDTNFDHHPTMDFKGDHDFHFLPFGSGRRACIAASHGLIVTHAT

IGALVQCFDWEVKDDAKIDNEMATGYSGSRVLPLACYPITRFDPTNA*

>CYP712D1 Vitis vinifera (grapevine)

MGTFTDLQYYTIFFILSFISTLLLRSFLNRITTPTTRLRLPPSPPALPIIGHLRLHFLSS

SIYKSFHSLSTQYGPLLYLHFGASRCLLVSSAAMAAEIFKTNDLAFASRPRLAFADKLPY

GTSSFITAEYGDYWRFMKKLCVTELLGVKQLERSRVVRREELDCFLKKLVESGENGEAVD

VRAEVMKLTNHSTCRVILSARCSEDNDEAERLIEMVTEWVELAVKMSFGDVFGPLKRLGF

WIYGRKAVELTLRYDEILEKMLKEHEERGKREDKDLMDVLLEVYQDDKAGMKLTRTHIKA

FILDLFMAGTNTSAESMQWTIAELINHPDVFKKVREEIDLAVGRTRLVEESDIPNLPYLQ

AVVKETLRLHPPAPVATRECRKNCKIGGFNIPEKTAVAINLYAIMRDPEIWDDPTEFRPE

RFLVPSKEQVDLDQTKGQNFNFVPFGGGRRGCPGTLLAFAMMNTTVAAIVQCFDWKLGGD

GDGGKVDMQSGPGLTLSMLHPLKCHPIVHFNPFEG*

>CYP712E1 Populus trichocarpus (cottonwood)

MIAIQYVLAIFVLWVITVFLQFIFKRPGKKPAGYCPPPSPPTLPLIGHLHLLTPVAYKGF

HALNNKYGPLLYLRLATYPAVLVSSAPLATEIFKALDVHFTSRIKSPFEDNLLFGSSTSF

FNAPYGDYWKFMKKICTTELLGTRQMKKLKNVRREEVVRFLSKMLEIGQKHEVANVSAEV

LTLANNSTCRMIMSARCSGEDNQAEKCRGLVSESFDLAAKLALFSVFGPLKRIGTWYLRK

KIADVPRRYDELFENVLVEHEEKAKREGPHMENKDLMDILLEVYHDKNAEIRITRKQMKT

FFLDLFTGGTNTTSDAILWILAELVNHPAAFKKLREEIDSAVGTERLVDEEDIPNLPYFQ

ACVKEAMRLNPPVPLFDRICGENCKLGGYDIPKGITMIMNAYSIMRDPKIWENPNDFIPE

RFLTEQDNAEGQNLQVYVPFGGGRRMCPGTNMTSSLINCSVTAMVQCFDWKVLGGDGPDG

SKVNMDSKSGVVKSMDKPFVAIPVLRRNLFSA*

>CYP714A1 Arabidopsis thaliana

MENFMVEMAKTISWIVVIGVLGLGIRVYGKVMAEQWRMRRKLTMQGVKGPPPSLFRGNVP

EMQKIQSQIMSNSKHYSGDNIIAHDYTSSLFPYLDHWRKQYGRVYTYSTGVKQHLYMNHP

ELVKELNQANTLNLGKVSYVTKRLKSILGRGVITSNGPHWAHQRRIIAPEFFLDKVKGMV

GLVVESAMPMLSKWEEMMKREGEMVCDIIVDEDLRAASADVISRACFGSSFSKGKEIFSK

LRCLQKAITHNNILFSLNGFTDVVFGTKKHGNGKIDELERHIESLIWETVKERERECVGD

HKKDLMQLILEGARSSCDGNLEDKTQSYKSFVVDNCKSIYFAGHETSAVAVSWCLMLLAL

NPSWQTRIRDEVFLHCKNGIPDADSISNLKTVTMVIQETLRLYPPAAFVSREALEDTKLG

NLVVPKGVCIWTLIPTLHRDPEIWGADANEFNPERFSEGVSKACKHPQSFVPFGLGTRLC

LGKNFGMMELKVLVSLIVSRFSFTLSPTYQHSPVFRMLVEPQHGVVIRVLRQ*

>CYP714B1 rice

MVVVVAAAMAAASLCCGVAAYLYYVLWLAPERLRAHLRRQGIGGPTPSFPYGNLADMRSH

AAAAAGGKATGEGRQEGDIVHDYRQAVFPFYENWRKQYGPVFTYSVGNMVFLHVSRPDIV

RELSLCVSLDLGKSSYMKATHQPLFGEGILKSNGNAWAHQRKLIAPEFFPDKVKGMVDLM

VDSAQVLVSSWEDRIDRSGGNALDLMIDDDIRAYSADVISRTCFGSSYVKGKQIFDMIRE

LQKTVSTKKQNLLAEMTGLSFLFPKASGRAAWRLNGRVRALILDLVGENGEEDGGNLLSA

MLRSARGGGGGGGEVAAAAEDFVVDNCKNIYFAGYESTAVTAAWCLMLLALHPEWQDRVR

DEVQAACCGGGGRSPDFPALQKMKNLTMVIQETLRLYPAGAVVSRQALRELSLGGVRVPR

GVNIYVPVSTLHLDAELWGGGAGAAEFDPARFADARPPLHAYLPFGAGARTCLGQTFAMA

ELKVLLSLVLCRFEVALSPEYVHSPAHKLIVEAEHGVRLVLKKVRSKCDWAGFD*

>CYP714C1 rice

MEKLLALIVVLVILLSLALFYLCNILWLRAVKIRKKLRRQGIRGPKPTFLYGNTKEIKRI

RQELKFSQKQGTNNFISTLFPHFLLCRETYGMHMLGPVFLYSTGALEILQVSHPDMVKDI

GRWTPSELGKPNYLKKSRKALFGGGLFTENGDEWAYQRKIIAPEFFMDKIKMIQLIEDAT

VTVLEAWEDMIDDVGGCREIVVDDYLRNLSADVIARACFGSSFTEGEEIFCKLRQLQKAI

AQQDSFVGLSALWKYLPTKSNQEIQMLDEQVRLLILDVAKEQHHYQDSHNSLLNAIIDGA

QDGRSAAEAEDFIVGNCKTIYFGGHESTVVTAIWCLMLLATHSEWQERARSEAMEVCRGR

STLDVDALRRLKIVTMVIQETLRLYPPASVMMQEALTDVKLGNIEVPRGTIVQVPRLMLH

LDKEAWGAHADEFRPDRFANGVAAACRAAHMYVPFGHGPRTCIGQNLAMAELKVVLARLL

TKFAFSPSPRYRHSPAFRLTIEPGFGLPLMVTKLP*

>CYP714D1 rice

MESFFVFFTAAALPVVVAAAVIAGLCITAAWLARPRRVAEVFRRQGIDGPPPSSFLAGNL

PEMKARVAAAASAAAPTADGEETASAGGGGGGRDFEKDGFDDYCTRIFPYFHKWRKAYGE

TYLYWLRRRPALYVTDPELIGEIGRCVSLDMGKPKYLQKGQEPLFGGGVLKANGACWARQ

RKVIAPEFYMARVRAMVQLMVDAAQPLIASWESRIDAAGGAAAAEVVVDGDLRSFSFDVI

SRACFGSDYSRGREIFLRLRELSGLMSETSVIFSIPSLRHLPTGKNRRIWRLTGEIRSLI

MELVRERRCAARAAREHGGKAAPPSPPERDFLGSIIENSGGQPRPDDFVVDNCKNIYFAG

HETSAVTATWCLMLLAAHPEWQDRARAEVLEVCGGDGAAAPAAPDFDMVSRMRTVGMVVQ

ETLRLFPPSSFVVRETFRDMQLGRLLAPKGTYLFVPVSTMHHDVAAWGPTARLFDPSRFR

DGVAAACKHPQASFMPFGLGARTCLGQNLALVEVKTLVAVVLARFEFTLSPEYRHSPAFR

LIIEPEFGLRLRIRRAGGQDATSQVDTSTAPVHSSHN*

>CYP714E1 Medicago truncatula (barrel medic, Fabales)

MSIVVEVMVALVAVLVALIHFLHVLVLRLRSLRAKLHRQGIHGPSPDFYFGNIKEMKTLL

LQQQTQVKQIKQEHEDEDVCASISHSWTSTVFPHIHKWRKQYGPTFLYSTGSIQWLLVTD

VEMVKEILLNTSFNLGKPSYLSRDMGPLLGQGIVSSSGLIWSHQRKIIAPELYLDKVKAM

VDRVIYSTNILIRSWESRIERDGVVSEIKVDEDLRSLSADIIARVSFGSNYVEGKEIFTK

LRDLIKLLSKIYVGIPGFRYLPNKSNRQIWRLEKEINSNISKLVKQRQEEGHEQDLLQMI

LEGAKNCEGSDGFFSNSISQDRFIIDNCKTIFFAGHDTTAITSSWCLMLLAKYQDWQDRA

RAEVLEVCGNGNPDASILRTMKTLTMVIQETLRLYPPAVFITRTSFQDINLKGIKVPKGI

NMQIPIAILQQDIDIWGPDAHEFNPERFANGVLGACKIPQAYMPFGIGSRVCPGQHLSMI

ELKVFLSLILSKFRVSLSSSYCHSPAFRLLIEPGHGVVLNMTRI*

>CYP714E4 Populus trichocarpus (cottonwood)

MELVLLISLAVVITFLGLLELLYSGLVLKPERLRSVLRKQGIRGPSPSLLLGNISEIRKS

QSTTVKASTNEPPVFHNCAATLFPFFEQWRKQYGPVFVFSLGNTQILYVSRADVVREIST

CTSLEFGKPSYQQKELGSLLGQGILTSNGKVWAHQRKIIAPELYGDKVKGMMSLIIESTT

VLLNSWKSRIDKEGGVAEIKIDEGMRSFSGDVISRACFGSNYSEGAEIFSRLRDLQEAMS

KKSLSTGIPGMRYIPTKNNREAWALEKYVRNLILEIVKERKETAHEKDLLQMVLESAKTS

NVGQDAMDRFIVDNCKNIYLAGYETTAVSATWCLMLLAANQEWQDRVRAEVLEVCGSGCL

PDADMLRKMKQLNMVIHESLRLYPPVAVVSREAFKEMKFGGITVPKGVNVWTMVLTLHTD

PEVWGPDAYRFNPDRFAKGITGACKLPHLYMPFGVGPRMCLGQNLAIAELKILIALILSQ

FSLSLSPKYIHSPALRLVIEPERGVDLLIKTL*

>CYP714F1 Populus trichocarpus (cottonwood)

MEGFPISYNFTCLVLLLTWSLIVYLYYSFWWRPELRLRKQGIRGPPPNFLLGNIPEIKQA

TVQNRSESTPSMESDSFSGFPSFKQWCKKYGNTYMFKLGALHFLYATNPFMVKEIKLFRS

LDLGKPAYLQKDRGVLLGKGVITSNGPAWSHQRKILSPQLYVDKXXDTLNIIVESGITVI

KSWERILMESKDGLDADIMVDSHMRSFTSCIISKLMFGHDHCRGMNVTARCHTLFKAMGT

PTTIGIPFLRYLPTKANRNAWRLAKEIHSMILDIAKNRCGSSTTKDILQVILEGSENGGP

GPSSAHEFIVDNCKDMLLAASEGTAISAMWGLMLLASNPEWQARARSEVKQVCGGHLPNF

NMLGKMKLKMVILEVLRLYPPVALVSRRALQDVKLCNMQVPKGVNIWIWAPALHRDPDLW

GPDADKFNPERFIDGVSGACKSSHAYIPFGVGARLCPGNKLGMIQLKVVLLAMILSSFNL

SISPNYRHSPTLGLLLEPEHGVNLVIQKI*

>CYP714G5 VITIS VINIFERA

MEAFTVKVFISLALAGVLGLFFRLYNALVVKPEKLRSILRSQGISGPPPSFLLGNIREIK

KSRSTAVKDSSTDTHNCAAALFPFFEQWRNKYGQVFMFSLGNTQILHANQPDIVREITTC

SSLDFGKPSYLQREFGPLLGQGILTSNGAVWAHNRKILAPELYMEKVKGMIGLITESVDT

LLNSWRSKIEAEGGIADIRIDEHMKSFSGDVISRACFGGSYTKGEEIFLKLKALQEAMSK

KAFSFIPGMRYIPIKRNRDAWALQKDVRNLILKVVSERKELAAHEKDLLQMVLEGAKKSE

LSQEATDNFIVDNCKNIYLAGYETTAVSAVWCLMLLAANPDWQARVREEVVEICKGRTPD

ADMIRKMKQMTMVIYESLRLYPPVPVMSREALADMKFGGIHVPKGVNVWNLVVTLHTDPE

NWGPDALKFNPERFKNGITGACKLPHLYMPFGVGPRVCLGQNLAMVELKILISLILSNFS

FSLSPNYKHSAALRLLIEPENGVNLLVKKL*

>CYP714H1 soybean

MEAEGQWQISEREICWSVVFIATCSIIILLYVKLWYRPQRIRSVLQKQGINGPKPSFPFG

NISEMQQLPNQLAPVSLEALDEWAYSIFPYFHTWRQLYGPMFMYSTGTNEHLYVETPELV

KWIGMHKSLHLGRPSYLTKTLKPLLGNGIIRSNGLHWAFQRNLLAPEFFHSKIKDWVDIM

EESTMAINKKWENHITESEGGIAELVIDGDMKALTADVISKACFGSTYAQGNLIFAKLAS

MQTALAKPNHIFGFLNLRFLPTKENKEIWKLQKEVEAMILKMIKEREAENQKSSTHGNQT

QKDLLQIILEGATSATSTESSGKGIFGPGYNIYQSIVDICKNMYFAGSESTALAITWTLF

LFALHPEWQQLVRSEIMETYGNMLPHSFRDMDRLRNLKALTMVIQESLRLYGPAVTTARG

VLAEMKLGEHVLPKGINMWLYIPALHRDPDNWGPDAREFKPERFAGGVSAACKYPQAYIP

FGLGSRICLGQNFALLEIKEALCLLLSNFSFAVSPNYHHCPQYRMLLTPKYGMRLLVSKV

HKTRT*

>CYP714J1 soybean

MEMNTVQVVLVAVVVVLIHVFNVLLLRSRSLRAKLHRQGIHGPSPHFYFGNIPEMKTLLL

QVQSAPITQVKDKDDHDSLSHKWPFTLFPHIQKWISQYGPIYLFSSGTIQWLMVSDIEMV

KEIIMYTSLNLGKPSYLSKDMGPLLGQGILTSSGPIWAHQRKIIAPELYLDKVKAMVNLI

VDSTNVTLRSWEARLESEGAVSEIKIDDDLRSLSADIIARTCFGSNYIEGKEIFSKLRDL

QKLLSKIHVGIPGFRYLPNKSNRQMWRLEKEINSKISKLIKQRQEETHEQDLLQMILEGA

KNCEGSDGLLSDSISCDVFMIDNCKNIFFAGHETTAITASWCLMLLAAHQDWQDRARAEV

LEVCGKGAPDASMLRSLKTLTMVIQETLRLYSPAAFVVRTALQGVNLKGILIPKGMNIQI

PISVLQQDPQLWGPDAHKFNPERFSNGVFGACKVSQAYMPFGIGARVCVGQHLAMTELKV

ILSLILLKFHFSLSLSYCHSPAFRLVIEPGQGVVLKMTRI*

>CYP715A1 Arabidopsis thaliana

MEFFEMSRVWYIFFKVFVVVICLMFLKLFLRCWIWPVRAQKKLRGNGFVGPAPSFPFGNL

NDMKKLKMASVVVDNSKSSTIINHDIHSIALPHFARWQQEYGKVFVYWLGIEPFVYVADP

EFLSVMSKGVLGKSWGKPNVFKKDREPMFGTGLVMVEGDDWTRHRHIITPAFAPLNLKVM

TNMMVESVSNMLDRWGIQINSGNPEFDMESEIIGTAGEIIAKTSFGVTGENGTQVLKNLR

AVQFALFNSNRYVGVPFSNILSYKQTVKAKGLGHEIDGLLLSFINKRKISLAEGDDQGHD

LLGMLLKADQKGNFTAKELVDECKTFFFAGHETTALALTWTFMLLAIHPEWQDTIREEIR

EVIGDSKIEYNKLAGLKKMSWVMNEVLRLYPPAPNAQRQARNDIEVNGRVIPNGTNIWID

VVAMHHDVELWGDDVNEFKPERFDGNLHGGCKNKMGYMPFGFGGRMCIGRNLTTMEYKIV

LSLVLSRFEISVSPGYRHSPTYMLSLRPGYGLPLIIRPL*

>CYP715B1 rice

MELVVQALAAAAALLAVFFLSTLYLSPAATARRLRNAGFRGPTPSFPLGNLREIASSLAS

NNDTDESNTKGGDIHAAVFPYFARWRRAFGKVFVYWLGTEPFLYVADPEFLKAATAGALG

KLWGKPDVFRRDRMPMFGRGLVMAEGDEWARHRHIIAPAFSATNLNDMIGVMEETTAKML

GEWGDMVASGRSCVDVEKGVVRNAAEIIARASFGISADDDDATGARVFHKLQAMQAILFR

STRLVGVPLAGLLHIRATYEAWKLGREIDALLLDIIESRRRREGGGGGKKKKKTTSNDLL

SLLLAGSEASAGAERKLTTRELVDECKTFFFGGHETTALALSWTLLMLAAHPEWQAAVRE

EVVEVAGRSGPLDAAALGKLTKMGCVLSEVLRLYPPSPNVQRQALQDVVVVAGDGEKKVV

IPKGTNMWIDVVAMHRDGELWGEEASEFRPERFMREGVQGGCRHRMGYVPFGFGGRICVG

RNLTAMELRVVLAMVLRRFAVEVAPEYRHAPRIMLSLRPSHGIQLRLTPLC*

>CYP716A1 Arabidopsis thaliana

MYLTIIFLFISSIIFPLLFFLGKHLSNFRYPNLPPGKIGFPLIGETLSFLSAGRQGHPEK

FVTDRVRHFSSGIFKTHLFGSPFAVVTGASGNKFLFTNENKLVISWWPDSVNKIFPSSTQ

TSSKEEAIKTRMLLMPSMKPEALRRYVGVMDEIAQKHFETEWANQDQLIVFPLTKKFTFS

IACRLFLSMDDLERVRKLEEPFTTVMTGVFSIPIDLPGTRFNRAIKASRLLSKEVSTIIR

QRKEELKAGKVSVEQDILSHMLMNIGETKDEDLADKIIALLIGGHDTTSIVCTFVVNYLA

EFPHIYQRVLEEQKEILNNKDVNEKLTWEDIEKMRYSWNVACEVMRIVPPLAGTFREAID

HFSFKGFYIPKGWKLYWSATATHKNPEYFPEPEKFEPSRFEGSGPKPYTYVPFGGGSRIC

PGREYARLEILIFMHNLVKRFKWEKVFPKENKLVADPAPIPAKGLPI

>CYP716B1 Picea sitchensis (Sitka spruce)

MVWKEAVSVLQKAQELKEPPLMFTVFLASFIGLAFFFYLISNHRTKAWRGIPPGTFGWPL

IGETLEFLGCQRKGNPRDFFDSRTQKYGNVFTTSLVGHPTVVFCSPEGNRFLFSNENKLV

VNSWPSSVGNLFRSSLITTVGDDAKRLRRILMTFLRPEALREFVGRVDSMTKRHLAEHWI

GKDEVMALPLLKRYTFSLACDLFASINTKDDLDRLWLHFMVFVKGVMQIPIDLPGTRYNK

TKHAANAIRQQLGSIINERKIALEAGNASPEQDLLSFLLSNVDEQGESLTDNEIQDNILL

LLYAGHDTSSSTLTVLLKFLAENPHCYEEVLREQLNIAGSKEEGQLLEWEDLQRMKYSWR

VAQEALRLFPAVQGSFRKAIKEFIYDGFTIPKGWKLHWTVNSTHQKSEYFSNPEKFDPSR

FEGEGPPPYTFVPFGGGPRMCPGNEFARMEILIFLHNIVKNFNWNLVNPLEKVIVDPMPA

PVNGLPIKLVPHD

>CYP716C1Poptr

MEVICLLLSMVLLVLALAISIFAFKHSSHSAKNLPPGSLGWPIFGETLDFLFGKPEKFVS

DRMKRYSSDIFKTKILGEETAVICGPGGHKFLFSNEQKLFTAFRPHAMQKIFRSYQAAAP

AQAQIPREAESKILRSPGFLKPEALVGYLGKMDSITQLHMQTYWEGKDEVKAFALAKTLT

LSLACRFFLGSDDPERIARLVSNFDDMTLGMHSIPLNVYGTTFYRANKAAAAIRKELRII

IDEKRADMSKGAQVQDILCHMILATDPSGKHMAEAEIADKIMGLLVAGYSTVATAMTFFM

KNVGERPDIYAKILAEQIEVAADKKAGELLDWNDIQKMKYSWNVMYEVMRLTPPLQGTFR

EALTDFTYAGYTIPKGWKIYWTVSTTNKDPEYFPDPEKFDPSRYDDEKVFPPFTFVPFGG

GPRMCPGKEYARLAILTFVHNVVKRFRWEVAFPAEKIVGDMMPTPEKGLPIRLRSRQAER

LASL*

>CYP716D1 Poptr

MFSSLDHDSIMILVAIPCLLLLYFAIKTLKERLFPNPHLPPGSLGWPLVGETLQFLPINL

PPEIFVNYRMKKYDSPVFKTSLFGETVAVFVGPAGNKFLFSNENKLVNVWWPTSVKKLMK

LSLANVVGDEAKRLRKILMTSVDRDALKSYIDRMDLVAQNHIRTRWEGKQQVKVHPTANL

YTFELSCRLFASIDDPIHISKLAHHFDIFLKGVIHFPIYIPGTTFYRASKSGDALKEEIR

LVARQRRAALDKKMESHRKDLLSHLLVTADESGKLLSESEIVDNMLMLLFVSHETTTSAM

TCVIKYLAEMPEVYEMVLREQLDIAKSKEAGELLKWEDIQKMKYSWRVVSEVLRMIPPIS

GTFRQAIVDFTYAGYTIPKGWKLYWSPNTTTKDPAHFPNAEDFDPSRYEGAGPAPYTHVP

FGGGPRMCLGYEYVRPKILVFLHNIVKRFKWDLLIPDEKVPYNPLPAPSHGLPIRIRPHQ

SSA*

>CYP716E1 Poptr

MPLIILLVVAAKYMDMDLRLLFSYLLPPAVLCISLYLVVSAYRRKSSNAKFPPGKTGWPI

IGETWDFVRAGRSGTPEKFVNDRMSKYSTDVFHTSLLGDNLAMFCGVSGNKFLFSSENKY

VTTWWPRPIQRILSFPEEIVTSSKDDSTILRRFLPEILKPEALKHYIPVMDSMAKDHLEA

DWSPYKQVRVLPLSKKYTFALACRLFMNIKDPAHVSRLENHFNLVTNGLVSVPINFPGTT

YYRAVKGGKIIREELLAIMKQRKGELASENYEERAEATDLLTLMLLASDDNGQPLNERDI

AYKVLGLLVAGHDTTSSAITMVMYYLAEYPHIYQGVLEEQKEIAMSKAPGELLNWDDVQK

MKYSWSVACEVLRVSPPVSGTFREVIADFSFAGFTIPKGWKAYWSVYSTHKNPKYFPDPE

KFDPSRFEGKGPAPYTFVPFGGGPFMCAGKEYARLEILVFMHNLVNRVKWEKVIPNEKIM

YTSFAMPVKGLPVLLQPLRN*

>CYP716F1 Physcomitrella patens (moss)

MAHQQHGFLEGHAESTPAWAAVAAVVAMLVGWLFWRLFSVSPESQGKLPVPPGSFKWPLL

GETLDYLDCARRNRVADFFNARVAKYGETFKTHILFNPTVSVTAPDGNKFLFANENKLVQ

NHWPPSVSRLLGEHSMATKVGEEHRRARRVYTNFFKPEGLQSFVPRIDELARSHNSKYWE

GKEFILGGPTVRDFTFAVAADLFLSLKHDDPMFRPFELAACDYLAGILQVPINLPGTAYR

KGILGRESQLRVIDMSLKQRRQEMKEGRVPPQQDLMSVLLNTLNEDGTPMSDDQIKDNML

LFVFAGHDTSSSALAGLLKYLSLNPECLKKVLEEQMEIRKEKGGEDIPLSWDDTRKMKYT

WRTIQETLRLQPSVQAAFRTVIEEFEYDGYTIPKGWTIFWSVGRSHRNPKFFPDPEKFDP

SRFEGTGPAPFTFVPFGGGPHICPGNEFARTEILVYIHYLVLNYEWEMVDPTEDVCIDPM

PLFTKQLQLRVRKRFPSL*

>CYP716G1 Medicago truncatula (Fabales)

FLQSLLLILIPLIAFFCFFLKTKQIGTKNMPPGAFGWPLVGETYQLLFKNIENFIQERAE

KHSSEIFKTNLFGEPTVVMFGPAANKFLSINESKLVKVWYMKSQCKLFNLPDQNQNQTQV

GVASPPVKVLGLLKHEGIIRYMGNNNNIESIIQKHFITHWEGKTELKVYPLVKSFSISLA

FQFFLGTDETHYVDKFATKFENLFSGIYSVPMDFPGSTYHRAIKGASEIRKEIQYMIKDK

IEGLSKGKVMDGLLAHIVDAEKSGKYVPKIEISNTIMGLMNASYISIATTLAFMIKHIGL

SPHIYQRIISEHADIKRSSKESGTSQLDWDSIQKLKYTWAVALESMRLYSPAPGAFREAK

TDFTYEGFTIPKGWKIFWAFIGTNKNPKYFDKPESFDPSRFEGNNVLAPYTYIPFGSGPR

SCPGKDYTRLAILTFIHNLVTKFKWEVMLPDEEVSGAMIPIPTEGIPIRLH

>CYP716H1 Solanum tuberosum (potato)

DLFLLSFATILTIIIYVFFKYFFAKPKEKIPPGTFGWPIIGETIQFFISLYYGMVHEFVQ

ERTKKYNSHVFKTSLLGQKVVIFSGPAANKFIFTQGNKLIIGWRPKSVQKLFASTSFVPI

EHDTKRAHNVISYLLNSQNVERLISTMDNMSHLHLKNHWKGKNEVIVYDLVKLFTFSLSI

RAFIGIKESDKILNLYEKFKIFTYGLLAVDINLPGTTFYKAMKAGNELRKQMKVIIKQRR

AELSENPNLSKVDVLTQMINEQDEDGKYMTEVEIEDKVFGFIIGSYDTTATTITLTMKYL

QQMPEFFNEIIQEQNEISMQMMPRKELCWNDIQKMKKTWSFVNEVLRNTPVVQGIFREVI

EDFTYEDFYIPKGWKIYLSFGATQKNGEYFPNPTKFDPSRFEGNGQVPYTSVPFGGGHRM

CPGKEFARILILVFLHHLLKNFRWEPKVPSEKILYPFFLLAIPTDGYPITLSAV

>CYP716J1Selaginella moellendorffii (lycopod, Gemmiferous spike moss)

MMIVVFFLVSTALLILTRSLLQFLRNSSSNSSSSSKGRVPPGSLGVPVIGDSLNFVKALK

RNDPWRFYGEKRAKYGTVFKMSLLGSPVVILPAPAGHKLLFGSEEKLMVNSWPVGFKRLL

GPGSLTSLTGEDFKRMKKVFMSFLKPEALQRYVPRVSQLSLKHLEDHWEAYAGEEFAIYP

AVKSFIFSVACSSFMSLETEEEQLELEEPFAIWTKGLLQLPVNIPGTLFHKALKRREVIH

DLLGRLISKRRQEFLQGRASESSDMLSVMLSYRNEDGKPACTDAEIKDNLLLLLFAAHDT

SSSTLTLSLKFLAENPYWRNQVLQENLAISQEKSGQDGYSLEWDDLRGMKVSWRVLQETL

RLQPPALSGYREVIQDFEFGDYLIPKGWKACWTVVSHRLPEFFPDPEKFDPSRFEGDGPT

PYTYVPFGGGPRMCPGNEYAKMVMLVLLHHLVLRFDWQLADPDEGVTMDPMPMPQNGLNV

KLHKRT*

>CYP716K1v1 Selaginella moellendorffii

MILLLGACFLLLITTIFAIRFSPSSNLPPGNLGWPIVGETLQFLALFRKNKAYSFFHERM

AKHGGVFKTSLLGSPTIVMPGPDGNKFLFSQENKLVVGCWPPSTASLLGPCSLAVQTGQE

HRRLREVFMTFLSSQALGRYLPKLCLLAQSFLQSKWNEEAVVTVAPLVQSFVFSAACNLF

LSMDKESDQELLLVPFYKFVKGMMSLPVHFPGTRYYEALKSREAILRLLDPVISARKKEL

LANPTDDRDMLSVLLTTCDEDGKLISENEIEDNVLLMLFAGHETVFRALTITMKMLTDNP

HWKEELYQEHLEIRASKSKPDYVLEWNDLRKMKLTWCSVQESMRLYPPSPGATRKATQEF

EYAGYRIPEGYKLMWSVNTSRMKDEFFPEPQKFDPLRFQGNGPAPYVFTPFGGGPRTCPG

NEFAKMEMLVFLHYLLLSHDWKPVITNEGIIVETAPLPAHGLPVKLSKR*

>CYP716L1v1 Selaginella moellendorffii

MALLAMVALLFFLVAPLFWIFNLIVASRKETPAQALQIPPGNLGWPLIGETFRYAVQSGS

TFYDERVAKYGAVFKTSLFGSKTVVLPAPEGNRLILMNENKLVSVSYPKSVSVLLGENSL

IALRGDEHRRSKALLMTFLRPEMLQKFVGRVCKVVHDHLQKFWSGGDEVIRVYNLMKMFT

FALACDLLMGLDIGDEEMEFLARDFDTLVRGLFQLPIDLPGTQFCRAKAARKKLDQCFDR

HIREKRRELAGSFRARSHEQDMLEVLLTTRDENGEFSTDLAIKDNIVSLLFAGHDTSSVA

LTWTLKFLADSPSCMDKVVQENLAVRSSRSSSELSWEDLRKLKYTWQVVQESMRMRPPVG

GGFREALVDLEFDGYLNWTTATSYRKPEFFVEPNKFDPSRFDGGNGIAPYTFLPFGAGAR

MCPGSEFAKMEILVFLHYCVLQFDWKLLEPNEQVIIDPMPRPVHGMPVRISKRN*

>CYP716M1v1 Selaginella moellendorffii

MDLPPGSLGLPLLGETLQFIRYTKSNRPWEFIEQREAKYGKIFKTSLFGSPMVMVSPPQG

NKLVFSNHNLLVETAWPSPMKTLVGSNAINFMSGEEAKSFRDVLMTFLSAQAVQSQVVPT

SNMIQDHLHKHWKHGETVLAYSLIKQALFSVTCCAFLSVSDEEEQLELLEPLAKIIKGLI

SLPLDLPWTNFHHAKKGRVELYKMFDKYIARRRIELENGSSSQQDLLSLLLSTKLDNGKL

MNDDQIKDNILSLLFAGHDTSSSSLAMTLKCLAQNPACYQELRREHLDILSAKQPGEELN

QNDLRKMKYTWMVIQETLRVMPTGFGILRKALKDIEMDGFTIPKGWQLLISGYRSYRKPE

FFAEPFKFEPSRFAEGTGPVPYTYIPFGGGPRICPGIQLAKMQVMVFLHHLVTRYEWTLV

EPDEPVSYTPVAMPTKGLPIKLK*

>CYP716N1 Selaginella moellendorffii

MSQALFFVLLFVISVIWFTFTNRNRKRKHASLPPGNMGLPFIGETLPFLRSLVSNQPWEF

FRVREAKYGKVYKTRLFGMPVVVVSPPVGTRFMFADTNHTLITKSWPVPVIKLFPESAFV

RPDASGSRQLITSFLGPECMKRYVTSTSVIVQKHLDSWPTGELVRVYPLIKRCLFSIVCN

MYLGLTDEKEVMELMEPFEKVIHGIISIPVNLPGTAFHRAKLGQKEICNILEKHIAKRRI

NSQLSPARDQDLLSMLLSTRSKEGTAMTDNEITHNILGLLVSGHELSASSISMTIKSLVE

NQTVYKEMKRVHCEIGSFKRPREPLEPLDLKQMKYSWRVVQESLRLRPTAPAVARKTLTD

VELEGYTIPKGWQMFSAVYNSHTTPEFFPDPLKFDPSRFERAGPNPYTYFPFGGGPRICG

GIEQVKMHSLVILHHITTRFDWTLMEPDEPIKISPVAVPAHGLPLELRLV*

>CYP716P7 Selaginella moellendorffii

MPWITVVASFLIPVLGTLWIWRYFSRERYDPPLPPGSMGLPLVGETLHILYAMKTSTLWE

FYGAREKKYGPIYKTHIFGRPTIVVSPPLGFKLLFSNHGKLVESSWPQPMKTLLGDKCLF

FMEGQKAKSFRHILLAFLGPDAIRRYVERASVIIQEHIDKFWMAGSEVKAYPLVKKALFS

LVFSLFLSISDEEEERELLAPFQGFLQGLLELPIDLPGTMFRRAKVGRAKIFKKLDEYIA

KRKIELETGKAWPQQDFLSVLLTTKGEDGEPMTKEEIKQNILMLVMSAHDTTVSSLVSSM

KYIGENPWCYDRLREEHVSIALAKSQKEPLTHSDLQKMDYTWKIVQEAMRLAPPAAGNLR

RATTEFTMDGFTVPKDWQLNWTVFRSHKKKEFFEEPEMFNPDRFDRPLLPNTYVPFGGGP

RICPGYELAKMQDRIFLHYLVTRFKWTLLDPNEAIHMTPLALPVNGLGIKLVSNPVKI*

>CYP718 Arabidopsis thaliana

MVLEPNFVLSWVFLCIAATISSTLFFFRKKHHRFITKKIQKKKKLLPGEMGLPWIGETMD

FYKAQKSNRVFEDFVNPRIIKHGNIFKTRIMGSPTIVVNGAEANRLILSNEFSLVVSSWP

SSSVQLMGMNCIMAKQGEKHRVLRGIVANSLSYIGLESLIPKLCDTVKFHHETEWRGKEE

ISLYRSAKVLTFTVVFECLYGIKVEIGMLEVFERVLEGVFALPVEFPCSKFARAKKARLE

IETFLVGKVREKRREMEKEGAEKPNTTLFSRLVEELIKGVITEEEVVDNMVLLVFAAHDT

TSYAMSMTFKMLAQHPTCRDTLLQEHAQIKANKGEGEYLTVEDVKKMKYSWQVVRETMRL

SPPIFGSFRKAVADIDYGGYTIPKGWKVILWTTYGTHYNPEIFQDPMSFDPTRFDKPIQA

YTYLPFGGGPRLCAGHQLAKISILVFMHFVVTGFDWSLVYPDETISMDPLPFPSLGMPIK

ISPKVS*

>CYP719A1 Coptis japonica (Japanese goldthread, Ranunculales)

MEMNPLLVCATVAIVFATTTIIRILFSSSSLPQMKWPSGPRKLPIIGNLHQLGDDVLHVA

LAKLAKVHGSVMTIWIGSWRPVIVISDIEKAWEVLVNKSADYGARDMPEITKIASASWHT

ISTSDAGSFWQNVRKGLQSGAMGPLNVAAQNQYQERDMKRLIKAMSDEAANNNGIVKPLD

HIKKNTVRLLTRLIFGQAFDDNKFIESMHYEIEDIIRISGYARLAEAFYYAKYLPSHKKA

EREAFLVKCRVEELVRPLLSSKPPTNSYLYFLLSQNFEEEVIIFCIFELYLLGVDSTSST

TTWALAYLIREQGAQEKLYQDIRMTLGDVDLVKIEDVNKLKYLQGVVKETMRMKPIAPLA

IPHKTAKETTLMGTKVAKGTRIMVNLYALHHNQNIWPDPYKFMPERFLEGETGTAYNKAM

EQSFLPFSAGMRICAGMDLGKLQFAFALANLVNAFKWSCVEEGKLPDMGEELSFVLLMKT

PLEARIAGRNV

>CYP719B1 Papaver somniferum (opium poppy, Ranunculales)

MAPINIEGNDFWMIACTVIIVFALVKFMFSKISFYQSANTTEWPAGPKTLPIIGNLHQLG

GGVPLQVALANLAKVYGGAFTIWIGSWVPMIVISDIDNAREVLVNKSADYSARDVPDILK

IITANGKNIADCDSGPFWHNLKKGLQSCINPSNVMSLSRLQEKDMQNLIKSMQERASQHN

GIIKPLDHAKEASMRLLSRVIFGHDFSNEDLVIGVKDALDEMVRISGLASLADAFKIAKY

LPSQRKNIRDMYATRDRVYNLIQPHIVPNLPANSFLYFLTSQDYSDEIIYSMVLEIFGLG

VDSTAATAVWALSFLVGEQEIQEKLYREINNRTGGQRPVKVVDLKELPYLQAVMKETLRM

KPIAPLAVPHVAAKDTTFKGRRIVKGTKVMVNLYAIHHDPNVFPAPYKFMPERFLKDVNS

DGRFGDINTMESSLIPFGAGMRICGGVELAKQMVAFALASMVNEFKWDCVSEGKLPDLSE

AISFILYMKNPLEAKITPRTKPFRQ

>CYP720A1 Arabidopsis thaliana

MAESAGESYRLLSVSSSTTFLAFIIIFLLAGIARRKRRAPHRLPPGSRGWPLIGDTFAWL

NAVAGSHPSSFVEKQIKRYGRIFSCSLFGKWAVVSADPDFNRFIMQNEGKLFQSSYPKSF

RDLVGKDGVITVHGDQQRRLHSIASSMMRHDQLKTHFLEVIPVVMLQTLSNFKDGEVVLL

QDICRKVSIHLMVNQLLGVSSESEVDEMSQLFSDFVDGCLSVPIDLPGFTYNKAMKVSFK

HLSQLLIQARKEIIRKINKTIEKRLQNKAASDTAGNGVLGRLLEEESLPNESMADFIINL

LFAGNETTSKTMLFAVYFLTHCPKAMTQLQEEHDRLAGGMLTWQDYKTMDFTQCVIDETL

RLGGIAIWLMREAKEDVSYQDYVIPKGCFVVPFLSAVHLDESYYKESLSFNPWRWQKRNW

RTSPFYCPFGGGTRFCPGAELARLQIALFLHYLIACFRWTQLKEDRISFFPSARLVNGFK

IQLNRRDSDPPNQ*

>CYP720B1 Pinus taeda (Loblolly pine)

MADQISLLLVVFTAAVALLHLIYRWWNAQRGQKRTSNEKNQELHLPPGSTGWPLIGETYS

YYRSMTSNRPRQFIDDREKRYDSDVFVSHLFGSQAVISSDPQFNKYVLQNEGRFFQAHYP

KALKALIGDYGLLSVHGDLQRKLHGIAVNLLRFERLKFDFMEEIQNLVHSTLDRWVDKKE

IALQNECHQMVLNLMAKQLLDLSPSKETNEICELFVDYTNAVIAIPIKIPGSTYAKGLKA

RELLIRKISNMIKERRDHPHIVHKDLLTKLLEEDSISDEIICDFILFLLFAGHETSSRAM

TFAIKFLTTCPKALTQMKEEHDAILKAKGGHKKLEWDDYKSMKFTQCVINETLRLGNFGP

GVFRETKEDTKVKDCLIPKGWVVFAFLTATHLDEKFHNEALTFNPWRWELDQDVSNNHLF

SPFGGGARLCPGSHLARLELALFLHIFITRFRWEALADEHPSYFPLPYLAKGFPMRLYNR

E*

>CYP721A1 Arabidopsis thaliana

MAVFFILVLVFFFLVFRFIYSNIWVPWRIQSHFKKQSVTGPSYRIFSGNSGEVSRLTAEA

KSKPIPSGRNPHEFVHRVAPHYHEWSRVYGKTFLYWFGSKPVVATSDPRLIREALTTGGS

FDRIGHNPLSKLLYAQGLPGLRGDQWAFHRRIAKQAFTMEKLKRWVPQMVTSTMMLMEKW

EDMRNGGEEIELEVHKEMHNLSAEMLSRTAFGNSVEEGKGIFELQERMMRLFYLVRWSVY

IPGFRFFPSKTNREIWRIEKQIRVSILKLIENNKTAVEKSGTLLQAFMSPYTNQNGQEEK

LGIEEVTDECKTFYFAAKETTANLMTFVLVLLAMNQEWQNIAREEVICVLGQTGLPTLDI

LQDLKTLSMIINETLRLYPPAMTLNRDTLKRAKLGDLDIPAGTQLYLSVVAMHHDKETWG

DDAEEFNPRRFEDPKKQSALLVPFGLGPRTCVGQNLAVNEAKTVLATILKYYSFRLSPSY

AHAPVLFVTLQPQNGAHLLFTRISS*

>CYP721B4 Zea mays

MAALTSALLFAALLVAAQYVLRLLHSFLWVPFRLERRFRRQGIRWPPRSLVSGNAADYRD

LLAAARSAPLSSFRHNGVVARATPQYAVWLARYGRPFVYWFGPRPRLVISDTELVKAVMT

DSTGGFDKAASGGNNPLARQLIGEGLVGLSGETWARHRRVISPAFNMERVKAWIPEIAAA

ASPVLDKWEAEGGSRTEFEIDVHKAFHTLSADVISCVAFGSSYEEGKRIFQLQEEQMQLA

LLAMRTVYIPGFRFVPTKKNRKRQRLNQEIQCSLRKLIEINGTKCEDSKNLLGLMLSASK

AGSEYKMGIEEIIHECKTFYFAGKETTANLLTWATLLLALHQEWQVKARDEVLKVCGKHE

HPNAENLSDLKIVTMVLKETLRLYPPATFINRTATRDIKLGKLDIPAGTRLDFPIIHIHR

DHEVWGMDAEEFNPSRFADGSSYHLGAYFPFGIGPTICVGQNLAMVEAKVALAMTLQRFA

FTVSASYAHAPMLVFTLQPQFGAQVLVRKI*

>CYP722A1 Arabidopsis thaliana

MEHLCLCLVLCAAMLTLGKFLKIMFQDRKKSTAGVPPGSDGFPVIGETLQFMLSVNSGKG

FYEFVRSRRRYGSCFRTSLFGETHVFLSTTESARLVLNNDSGMFTKRYIKSIGELVGDRS

LLCAPQHHHKILRSRLINLFSKRSTALMVRHFDELVVDALGGWEHRGTVVLLTDLLQITF

KAMCKMLVSLEKEEELGSMQRDVGFVCEAMLAFPLNLPWTRFHKVMQARGRVMEMLEKII

RERRNEINSHNNHHEDFLQQLLAVDNDTPQLTDAEIKDNILTMIISGQDTTASALTWMVK

YLGENQKVLDILIEEQSQITKKASNKPFLELEDLSEMPYASKMVKESLRMASVVPWFPRL

VLQDCEMEGYKIKKGWNINIDARSIHLDPTVYSEPHKFNPLRFEVNKPKANSFLAFGMGG

RTCLGLALAKAMMLVFLHRFITTYRWEVVDEDPSIEKWTLFARLKSGYPIRVSRRL*

>CYP722A1 Vitis vinifera (grapevine)

MEXDEIKRSMLNLLPNATLQLCCYTVVLIVSLMWLMRGIARVLRDSHWESTAKIPPGSRG

LPLIGETLHFMAATSSSKGFYDFVHIRQLRTSIFGQTHVFVSSTESAKVVLNNEVGKFTK

RYIKSIAELVGNESLLCASHQHHKLIRGRLINLFSTASISSFIKQFDQLIVTTLSGWEHK

PTVVVLHEALELICKAMCKMLMSLESGDEVEMLQKDVAHVCEAMIAFPLRLPCTRFYKGL

EARKRVMKMLEKKIEERRRGEAYHEDFLQHLLKDNGSACCDEVPPLTDAEIQDNILTMII

AGQDTTASAITWMVKYLDENQHVLHTLRAEQGRIAEKTSHTSSLTLDDLNEMPYASKVVK

ESLRMASIVAWLPRVALQDCEVQGFKIKKGWNINIDARSIHLDPTLYNNPTMFIPSRFDG

EXKPNSFLAFGTGGRTCLGMNMAKAMMLVFLHRLITTYNWTVVNPDSSIEKWALFSRLKS

GCPIHVSPIAKDAADA

>CYP722B1 Oryza sativa (rice)

MESLAAGAWWVVVLLLLVLTIVASWYRSWWKTTEAGGPLLPPPAAGAGPWWVWVWQWRET

AAFLASHGSGRGFYHFVQERYKLYKGEGEGEATCCFRTALMGRVHVFVSASHPAASQLLT

AEPPHLPKRYARTAADLLGPHSILCSTSHAHHRHARRALATTLFATPSTAAFAAAFDRLV

IRHWTTLLPPHNQNQVVVVLDAALHISYRAICEMLLGAGGGKLRPLQSDVFAVTQAMLAL

PLRWLPGTRFRRGLHARKRIMAALREEMAARNHHHHHHHHHHDLLSVLMQRRQLGHPDAL

TEDQILDNMLTLIIAGQVTTATAITWMVKYLSDNRLIQDKLRAEAFRLELKGDYSLTMQH

LNAMDYAYKAVKESLRMATIVSWFPRVALKDCQVAGFHIKKDWIVNIDARSLHYDPDVFD

NPTVFDPSRFDVQPQKRRLLVFGAGGRTCLGMNHAKIMMLIFLHRLLTNFRWEMADDDPS

LEKWAMFPRLKNGCPILLTPIHNS*

>CYP722C1 soybean

28703503MLNLLREEVLLVVQKYYYDLIMVALFTIGVTYLASKAWKRATTNNREEIPGR

LGLPFIGETFSFLSATNSTRGCYDFVRLRRLWNGRWFKTRLFGKIHIFIPSPEGARTIFA

NDFVLFNKGYVKSMADAVGQKSLLCVPVESHKRIRGLLSEPFSMTSLSAFVTKFDKMLCG

RLQKLEESGKSFKVLDLCMKMTFDAMCDMLMSITEDSLLRQIEEDCTAVSDAMLSIPIMI

PRTRYYKGITARKRVMETFGEIIARRRRGEETPEDFLQSMLQRDSLPASEKLDDSEIMDN

LLTLIIAGQTTTAAAMMWSVKFLHDNRETQDILREEQLSITKMKPEGASINHEDLNSMRY

GLKVVKETLRMSNVLLWFPRVALEDCTIEGYDIKKGWHVNIDATHIHHDSDLYKDPLKFN

PQRFDEMQKPYSFIPFGSGPRTCLGINMAKVTMLVFLHRLTGGYTWTLDDLDTCLEKKAH

IPRLRNGCPITLKSLSKSMPEA*28707854

>CYP723A2 Oryza sativa (rice)

MMLLLTLLLVLTLFLCLAVFRRTTSRARRAPVSLRQPTVEIHDGDVARRALLDHADAFVN

RPAIGAEPRGRRSDNLTTVRYGPQWRVLWRNLTAGFLRPSRVGLLAPLQQKAVDALVADI

AARGADGGEVGVRDVVHDALFPLAVRFCFGDDIDERHVRDLRRVMQELKLDVVVKGFSGS

MLANLVHWWRLRRFIASGRRRAEIFLPLIAQRRRTQHRDADGGVFRPYVDSLLDLRVPVG

HDADADAAGCEDNEGRNSGRALTDDEMVGLVAEFLSGGTETVVSCVEWTLAHLVIEPEIQ

DKLCRQVVAAADHHGGERGTTPAYLRAVILESLRMHPPVPLTMRDVRSPQAVEHLSLPDG

GARVHFILGDIGRDGKAWTDPDEFRPERFMAGGEAEGVGPLPGPREVRMMPFGAGRRYCP

GMGLGVAHACLLVAALVREFEWAPTAVAATGGVDLTEVNGFFKMMRTPLRARATPRGTSA

*

>CYP724A1 Arabidopsis thaliana

MLVLSIFLSLGLFFLSILILYISISKKNETNDHHSSLTGSMGWPFIGETISFFKPHRSDS

IGTFLQQRVSRYGKVFKSNICGGKAVVSCDQELNMFILQNEGKLFTSDYPKAMHDILGKY

SLLLATGEIHRKLKNVIISFINLTKSKPDFLHCAENLSISILKSWKNCREVEFHKEVKIF

TLSVMVNQLLSIKPEDPARLYVLQDFLSYMKGFISLPIPLPGTGYTNAIKVRSNRNIHQN

AIIEDMNNAIREEDFLDSIISNEDLNYEEKVSIVLDILLGGFETSATTLSLVVYFLAKSP

NLLHKLKEEHAAIRAKKGDGELLNWEDYQKMEFTQCVISEALRCGNIVKTVHRKATHDIK

FNEYVIPKGWKVFPIFTAVHLDPSLHENPFEFNPMRWTDKAKMNKKTTAFGGGVRVCPGG

ELGKLQIAFFLHHLVLSYRWKIKSDEMPIAHPYVEFKRGMLLEIEPTKFLED

>CYP724B1 Oryza sativa

MVGGELVLAALVILLALLLTLVLSHFLPLLLNPKAPKGSFGWPLLGETLRFLSPHASNTL

GSFLEDHCSRYGRVFKSHLFCTPTIVSCDQELNHFILQNEERLFQCSYPRPIHGILGKSS

MLVVLGEDHKRLRNLALALVTSTKLKPSYLGDIEKIALHIVGSWHGKSKDKGMVNVIAFC

EEARKFAFSVIVKQVLGLSPEEPVTAMILEDFLAFMKGLISFPLYIPGTPYAKAARARIS

STVKGIIEERRNAGSSNKGDFLDVLLSSNELSDEEKVSFVLDSLLGGYETTSLLISMVVY

FLGQSAQDLELVKREHEGIRSKKEKDEFLSSEDYKKMEYTQHVINEALRCGNIVKFVHRK

ALKDVRYKEYLIPSGWKVLPVFSAVHLNPLLHGNAQQFQPCRWEGASQGTSKKFTPFGGG

PRLCPGSELAKVEAAFFLHHLVLNYRWRIDGDDIPMAYPYVEFQRGLPIEIEPLCSES*

>CYP725A1 Taxus cuspidata (Japanese yew)

MDSFIFLRSIGTKFGQLESSPAILSLTLAPILAIILLLLFRYNHRSSVKLPPGKLGFPLI

GETIQLLRTLRSETPQKFFDDRLKKFGPVYMTSLIGHPTVVLCGPAGNKLVLSNEDKLVE

MEGPKSFMKLIGEDSIVAKRGEDHRILRTALARFLGAQALQNYLGRMSSEIGHHFNEKWK

GKDEVKVLPLVRGLIFSIASTLFFDVNDGHQQKQLHHLLETILVGSLSVPLDFPGTRYRK

GLQARLKLDEILSSLIKRRRRDLRSGIASDDQDLLSVLLTFRDEKGNSLTDQGILDNFSA

MFHASYDTTVAPMALIFKLLYSNPEYHEKVFQEQLEIIGNKKEGEEISWKDLKSMKYTWQ

AVQESLRMYPPVFGIFRKAITDIHYDGYTIPKGWRVLCSPYTTHLREEYFPEPEEFRPSR

FEDEGRHVTPYTYVPFGGGLRTCPGWEFSKIEILLFVHHFVKNFSSYIPVDPNEKVLSDP

LPPLPANGFSIKLFPRS

>CYP726A1 Euphorbia lagascae

MEQKNLSFPSILISFLLVLILVVVMRLWKKQNPPPGPWKFPIIGNLPHLLLTSDLGHERF

RALAQIYGPVMSLQIGQVSAVVISSAEAAKEVMKTQADAFAQRPIVLDAQIVFYNRKDVL

FASYGDHWRQMKKIWILEFLSAKKVQSSRLIREEEMEDAITFLRSKAGSPVNITKIIYGI

IISIMIRTSVGNCKQKERLLSVADAVNEAATSFGTADAFPTWKLLHYIIGAESKPRRLHQ

EIDDILEEILNEHKANKPFEADNLMDVLLNLQKNGNVPVPVTNESIKASVLQMFTAGSET

TSKATEWVMAELMKNPTELRKAQEEVRQVFGEMGKVDESRFHDLKFFKLVVKETLRLHPP

VVLIPRECRETTRIDGYEIHPNTRIVVNAWAIGRDPNTWSEPGKFNPERFKDCAIDYKGT

TFELVPFGAGKRICPGITSAITNLEYVIINLLYHFNWELADGITPQTLDMTEAIGGALRK

KIDLKLIPIPYQVSLGSNIS

>CYP727A1 Oryza sativa rice (japonica cultivar-group)

MASPCEHHVPYTLLGALLSGGGPHAAACGGAAFLRDYAERGTNALLWAALLAVTWLLVLR

LAALLRLWALGARLPGPPAFPADPGLAAGDITGYLSKLHGSYGPVVRLWLGPSQLLVSVK

DSRVIKELLTKAEDKLPLTGKTYNLACGKLGLFISSFEKVKSRRESLKSFLDEKLSVGTG

GSSFKIIQIVLDRVDSIMARDFLDCRYFSQHMAFNIVGSALFGDAFFDWSDASAYEELMM

TVAKDACFWASYAVPPFWKPDYRRYRTLCARLKLLTQGIVAKSRNQNGVLSLIDLSSCQR

SERMIKDPCRGFSLLDGVISSRCLNEAAEGPLSSEEEICGNIMGLMLHGISTCANLIGNI

LTRLALYPNLQCQLHSEIVSGHSESSELKIDDVLRMKFLLATVCESARLLPAGPLLQRCS

LQQDVNLNSSITIPAGAILVIPLHLVQMEASTWGNDACQFNPNRFLKKEINFEEILAAAH

KGSNGINLFTDECDKTESFLPFGSGSRACVGQKFAVLGIAMLIASLLRSYEVQPHPALSQ

EMESLVDSNSLHHIPNPKIILKKRSI*

>CYP727B1 Populus trichocarpa (black cottonwood)

MNSVLNSVNDFVSSKSTKEYAKKELNAILWVALITITVFSLEKVFKLFRLWSKASQIPGP

PCNSFFGHGNLGSRENFIDLLSVSHDKYGSVFKLWLGPTQLLVSIKDPALIKEMLLKAED

KLPFIGKAFRLAFGRSSLFFCSYDQKRRESLALELNGKMLGRANVIPKNVVDCIMERVDA

IMSKGSVDCKSVSQHMAFTILGTALFGDTFLAWSKATFYEELLMMIAKDASFWASYRVTP

FWKRGFWRYQSLCTKLKCLTLDIVQQCGKNYGLFSHMDQNSHIGIEKVGIKAASGAPPSN

GVEMQDKLFSPELDGHLNEREEPCGNIMGVMFHGCIATASLIGSILERLVTDVEMQDKIY

SEIIKVKQGSVKEDQNVEKMLLLLATIYESARLLPAGPLLQRCSLKDDLIFKSGVVIPAG

AVLVVPAQLLQMDDSSWGSDASKFNPYRFLSKAGKDSDLVQDTSFTEEAVDPIQCSFILN

DPNDNAAFLPFGSGMRACVGQKFAIHGVATLFASLLQRYEVRLDPQLANNPKSTTGPQIV

FVRRNS*

>CYP728A1 Oryza sativa (rice)

MDASAMLVALLTILATAAAVASSSLRRRKNQPPGSLGLPVVGHTLALLRALRSNAARAAA

YGPVSTISLFGRPTAFLAGASCNKLLFSSDKLAAMSSASFLRMVGRRNIREVAGDDQRRV

RAMMARFLRLDAVKNYVSAMDDEVRRHLRAEWGGRAAVAVMPSMKSLTFDVMCTVLFGLE

RRGDHAAVRRELSSEFQQLVRGIWAVPVNLPFTTFGKCLAASRRGRRAVARIVEERRRAM

PRGGGGGGSAGDLVTHMLAEGMDEEEIIDNVVFLMVAAHDTTAVLLTFLLRHLDGNRAAY

ERVAAEQEAIATQRRRRGGSGSGSGSALTWDDLAGMRYTWAAAMETLRMVPPTFANMRKA

VADVEVGGYVIPKGWQVITAATMTHLDPTIFPDPGRFEPARFEAAAAKSAPPPFSYVPFG

GGARACPGNEFARAETLVAMHYIVTGFRWRLAAGCDGGFSRHPLPCPNQGLLLDIEPKE

>CYP728B1 Oryza sativa (rice)

MALAVVVVALLVAFLTPLAVYLAGRSTRTKPPPRRNLPPGSLGLPLVGQSLSLLRAMRRN

TAERWLQGRIDRYGPVSKLSLFGAPTVLLAGPAANKAVFLSEALAPKQPRSLAAIIGRRN

MLELVGDDHRRVRGALAQFLRPEMLRRYVGRIDGEVRRHLAGRWAGRRTVAVLPLMKLLT

LDVIATLLFGLARGAVRERLAAAFADMLEGLWAVPLDLPFTAFRRSLRASARARRLLAAT

VREKKANLEQGESSPSDDLISYLVSLRDGDGGGGRPLLTEEEIIDNSIVCLTAGHDTSAI

LLTFMVRHLADDPAILAAMHEEIARSKRDGEALTWEDVARMKLTWRVAQETLRMVPPVFG

SFRRALEDVELDGGYVIPKGWQVFWAPCVTHMDPAIYHDPDKFDPSRFDAQAAASAPPPY

SFVAFGGGPRICPGMELARVETLVTMHYLVRHFRWRLCCGGEENTFVRDPLPSPANGLPV

ELDHIAPLRCDEFNS*

>CYP728C1 Oryza sativa (rice)

MDSSMPFALLLALLIPTLLRFVIRRKYSSYNLPPGSLGFPLIGQSISLLRALRSNTDYQW

YQDRIKKYGPVFKMSLFGSPTVLMAGPAANHFVFSNQDLIFTQTKAINTILGRSILTLSG

EELKRVRSALQGYLRLEMVTKYMRKMDEEVRRHIDLNWVGHKTVKAAPLAKRLTFDIICS

VVFGQGIGPIREALATDFETLVQALLSLPVNIPFTKFIKGLRASRRIRKVLRQIAREREA

ALQQGHSSSADDFFTYMLVLRSEGTHSLTVEDIVDNAIVLLTAGYGNSAVLITFLLRYLA

NDPDILGKITEEQEEIARRKGPNEPLTWDDVSRMKYTWKVALETLRTVPPIFGSFRTAVK

DIEYHGYHIPKGWQVFTAQRITHLDGNFFNDPVKFDPTRFDNHTSIPPYCFVPFGGGPRM

CPGNEFARTEILVTMH*LVRQFRWKLCCEEEGYRKDPVPIPVLGLPIELETRSPPEYAHA

*

>CYP728D2 Populus trichocarpus (cottonwood)

MNPEILFALLLFLLPLYFLLTRRSSKRLPPGSLGLPIIGQTLSFLNAMRKNTAEKWLQNR

TRKYGPVSKMNLFGTPTVFLQGQAANKFIYTCDGDTLSSQQPLSVKRICGERNIFELSGL

EHRRVRGALVSFLKPEVLKQYVGMMDERIRKHFEMHWHGKQKVMAMPLMKTLTFNLMSSL

IMGIEQGSKRDVPVKLFQQLMEGLISVPINLPFTRFNRSLQASEKIREIVMDLIREKRVA

LDHQNASPQQDLITSLLSLRNDHNSVALSDEEIVDNAIIIMIGGHDTSSILLAFLIRLLA

KDPSVYAGVVQEQEEIAKNKASNELLTWDDLGRMKYTWRVAMESLRMNPPVFFSFRKVLK

DFNYEGYLIPKGWQVMWAACMTHMDGSIFPNPSDFDPKHFERQSSIPPYSFMGFGGGPRI

CPGYEFARLETLITVHYLVNMFTWKLCCPEISFSRDPMPTFKDGLEIEIEPKILGEII*

>CYP728E1 Populus trichocarpus (cottonwood)

MDLMILFEHPSFESKVSSPTIILVTLLALVAGFYYKLKASKLAGKKLPPGSLGFPLVGES

ISLVRAQKRDKIDEWMWKRIDKFGPIFKTSIFGTKTVVLTGQAGNRFLFSGGDGISYKQP

KTIASILGKYSLFEISGSRHKLIRGAIVGFLKPERIQKIVGEINSLVQQQLSKELDGVDS

VKIVPFMKRIAFNITCNIFFGIPDGKEKDTLFEEFSVAVKGCWAVPLDIPGTVFHRAMQA

RASLCKILSKIIDERKRQMEEGTVDVNENIIYSFLSLRDENDEPLIEEEILDMVLSLIMA

SHDSTTILLCLLVRLLSRDAEIYNKVLEEQREVIKVKGGSDGKITWNEIQMMKYSWRVAQ

EVMRFYPPIFGNFRQITKDIEFDGFHIPKGWQVLWVASGTHMDKSIFEDPEKFDPSRFDT

SSKTFPPYTYVPFGAGLRICPGADFVRIESMLVIHHFITKYQWKEIIPDEPIIRDPMPYP

AMGLPVKFYPRSGDLAIAGNDI*

>CYP728F1 Populus trichocarpus (cottonwood)

MEAVFGLDKLSSTTVISLATLTTLVAVIWTYRFSLIQRKKLPPGKLGLPFIGESISFFRA

HKHNNIGKWIEERTIKYGPVFKTSLMGENVVVMTGEASHRFIFSGRDNGIAAKLATSALA

ILGKNNIFDLYGSPHKLVRSAIMSFLNSECIQRYVSKMDSLVKEQVLQELNDKETVQVVL

LMKKISFIATASLLFGLPEAKERDGLFKDFTIAVKGMWSIPLNLPGSTFRKAVQARGRIF

KLFTNLIAERKRGLEDGSMGSHDDVILCLLSLRDENGKTLPDEEIINNLIALMMASHDTT

SVLLSLIVRELAKNASVYDKVLEEQNEIAKVRSIASDGQLGWREIQKMRYTWNVAQELMR

LTPPIIGNFRHAWRDTTFNGYDIPKGWQVFWLATSTHLDNKVFEDPVKFNPSRFDTNSKS

SVPPYTYIPFGAGPRVCPGAEFARTEVLLIIHHLITNYKWTAMVEDEIVVRDPMPFPNKG

LPVKIYPKHNI*

>CYP728G1 Vitis vinifera (grapevine)

MEVLLSSLEESSFEWKLPLISTILVTLIALLAGSIKLKFSPPVDKKLPSGSLGFPFIGET

ISFLRAQRQDKTVEWIESRIAKYGPVFKTSLMGSKVVVLTGQAGNRFLFSGSDNGILSNQ

PMSVAKILGKHSIFELAGTRHKLVRGAIMNFLKPESIQRSVSRMDSVVQQQLFQELEGKD

SVQMVGLMKKITFKVTCSLLFGLPDGKETEELLEDFTTALKGAWTVPWDLPGTVFRKALQ

ARGRICKQLAQLVRERKAKIEEGRVDSHEDIISSLITLRQENGQPLSEEEIIDNLISVVI

ASHDTSTVLLGLLIRHLARDTEVCKKVLEEQKQVAKAKEGKGNGKLTWGEVQMMKYTWRV

AQELMRMTPPVLGNFKCAWRDTTFGGFDIPKGWQVFWVAPGTHMDKKVFEEPEKFDPSRF

ENPSTSVPPYAYLAFGAGPRACPGADFSRVEVLLMIHNLITKYHWAEMIIDEPIVREPMP

YPAMGLPVKLYQRSTT

>CYP728H1 soybean

MVYGILFFVLFAFTLSLAFLLSKCLSKSQTKNVPKGSLGYPIIGETLSFLKAQRQDKGSV

WLEERISKYGPIFKTSLMGFPTVFVIGQEGNKFVLGSPDDLLSSKKPLTLRKILGRQSLV

ELTGPRYRLVKGEMLKFLKPECLQNYVKEMDELVNATLLREFRENEIIRAVVFMKKLSYE

IACNLLFDIKDEHTKEALFVDFTLAFKAIHSLPINLPGTTFWRGQRARARIVDRMIPILN

KRREELSKGVLSSTNDMLSCLLALRDENHQPLDDDLITDNFIFLFVASHDTSATLMSLMI

WKLSRDQEVYNKVLEEQMEIIKQREGTEERLTWAEIQKMKYTWRVAQELMRMIPPLFGSF

RKALKDTNYKGYDIPKGWQVYWAAYGTHMNDDIFENPHKFDPSRFENPTKPIPPYSYLPF

GAGLHYCIGNEFARIETLAIIHNFVKMYEWSQVNPEEAITRQPMPYPSMGLPIKIKPRSC

SIS*

>CYP729A1 rice

MSGATADWAWWLGLVAGAVPLLALAVWHCTDAFHSAAFAFRRRGTRARLPPGHMGLPFVG

ETLALIWYFNLARRPDAFIEAKRRRYCYGDGDDDGGIYRTHLFGSPAVLVCSPASNGFVF

RSAPPGSFGVGWPVPELVGASSLVNVHGGRHARLRRFVLGAINRPGSLRTIARVAQPRVA

AALRSWAAKGTITAATEMKNVTFENICKIFVSMEPSPLTEKIHGWFTGLVAGFRSLPLDM

PGTALHHARKCRRKLNSVFREELERRKVKMVTGEGGDDDDDGDLMSGLMHVEDEQGRRLD

DDEVVDNIVSLVIAGYESTASAIMWATYHLAKSPSALAKLREENLAIAKEKNGDGFITLE

DVSKMKYTAKVVEETIRLANIAPMAHRVALRDVEYRGYTIPKGWKVIVWIRSLHVDPAHY

DNPLSFNPDRWDKSAELGTYQVFGGGERICAGNMLARLQLTIMLHHLSCGYKWELLNPDA

GIVYLPHPRPTDGAVMSFSEL*

>CYP729A6 Lotus japonicus

MDAVSGWFVTVVICGLLWWWNVLWYVVPLSLRGKLPPGNMGLPFVGDMISFLWYFKFLRR

PDDFINAKRRKYGDGAGMFRTHLFGTPSIIVYTPAVSKFIFRSEDKFMQEWPTIELMGRT

SMVAVHGKAHARVRSFVMNAINKPEALRRLAALVQPRMISALESWAKMGKIKAQFETQKM

TFENISKSFMSMEPGPFLLSMDKLYKGLLEGVRAYPIDFPGFAYHRSIQCRKKLEEIFWT

EFDNRKKESYKLKPNNDLMDGLMQIEDAEGDKLSDTEVVDNIVSLVVAGYMSTSLVSMWA

ISLLAKYPNVLKKLREENMALEKGSPGDLITANDVSNLKYTNKVVDEVIRVANVAAFVFR

KSVEEAEYKGYKIPKGWNVLVFIRYIHTNPEHFHDPMYFNPERWNEPLKPGTNQVFGGGQ

RLCPGNMLAKIQLALLLHHLSLGYKWELLNPNADTIYLSHPAPSDGVEVNFSKL*

>CYP733A1 Oryza sativa (rice)

MAVFGAAVLVALAVTCGLIWSRSRRLSKEMRDIPGTMGWPVVGETFSFISGFSSPAGILS

FMRDRQKRFGKVFKTYVLGRMTVFMTGREAAKILLSGKDGVVSLNLFYTGKQVLGPTSLL

TTNGDEHKKLRRLIGEPLSIDALKKHFDFINDLAVQTLDTWLDRRVLVLEEASSFTLKVI

ANMLISLEPEGEEQEKFRANFKIISSSFASLPLKIPGTAFHRGLKARNRMYAMLDSVIAR

RRDGGEVRNDFLQTLLRKHAKDDADKLTDAQLKDNILTLLVAGHDTTTAGLTWLIKFLGE

NPEALQKLREEHMEIKERLDGSSHLRWSDVNSMPYTNKVMNETLRRATILPWFSRKAAQD

FSIDGYEIKKGTSVNLDVVSIHHDPSVFADPYKFDPNRFDGTLKPYSFLGFGSGPRMCPG

MSLARLEICVFIHHLVCRYSWTPLEDDDSVQPTLVRMLRNKYPIVAAAI*

>CYP734A2 Oryza sativa (rice)

MEEDGGGGAGWGWATWRVAAVAAAAAVWVTMHVAARMADALWWRPRRLEAHSRGAGVRGP

PVPVLLGSVREMVALMAEASSKPMSPPTSHNALPRVLAFYHYWRKIYGHRFLIWFGPTPR

LTVAEPELIREIFLTRADAFDRYEAHPVVRQLEGDGLVSLHGDKWALHRRVLTDAFYPDN

LNRLIPHVGKSVAALAAKWGAMAEAGGSGEVEVDVAEWFQAVTEEAITRATFGRSYDDGR

VVFAMQGQLMAFASEAFRKVLVPGYRFLPTKKNRLSWRLDREIRRSLMRLIGRRSDEAEQ

GEKADDGSFRDLLGLMINAGAAAATRGNAGGEKNSPAAAIPVEDMLEECKTFFFAGKQTT

TNLLTWATVLLAMHPDWQERARREVFDVCGAGELPSKEHLPKLKTLGMIMNETLRLYPPA

VATIRRAKVDVQLSDGCMIPRDMELLVPIMAIHHDTRYWGPDASQFNPARFANGASKAAK

HPLAFIPFGLGSRMCVGQNLARLEAKLTMAILLQRFEIRTSPNYVHAPTVLMLLYPQYGA

PLIFRPLSSHPPDSTGP*

>CYP735A2 Arabidopsis thaliana

MVTLVLKYVLVIVMTLILRVLYDSICCYFLTPRRIKKFMERQGITGPKPRLLTGNIIDIS

KMLSHSASNDCSSIHHNIVPRLLPHYVSWSKQYGKRFIMWNGTEPRLCLTETEMIKELLT

KHNPVTGKSWLQQQGTKGFIGRGLLMANGEAWHHQRHMAAPAFTRDRLKGYAKHMVECTK

MMAERLRKEVGEEVEIGEEMRRLTADIISRTEFGSSCDKGKELFSLLTVLQRLCAQATRH

LCFPGSRFLPSKYNREIKSLKTEVERLLMEIIDSRKDSVEIGRSSSYGDDLLGLLLNQMD

SNKNNLNVQMIMDECKTFFFTGHETTSLLLTWTLMLLAHNPTWQDNVRDEVRQVCGQDGV

PSVEQLSSLTSLNKVINESLRLYPPATLLPRMAFEDIKLGDLIIPKGLSIWIPVLAIHHS

NELWGEDANEFNPERFTTRSFASSRHFMPFAAGPRNCIGQTFAMMEAKIILAMLVSKFSF

AISENYRHAPIVVLTIKPKYGVQLVLKPLDL*

>CYP735A3 Oryza sativa (rice)

MAMAAAVLVAIALPVSLALLLVAKAVWVTVSCYYLTPARIRRVLASQGVRGPPPRPLVGN

LRDVSALVAESTAADMASLSHDIVARLLPHYVLWSNTYGRRFVYWYGSEPRVCVTEAGMV

RELLSSRHAHVTGKSWLQRQGAKHFIGRGLLMANGATWSHQRHVVAPAFMADRLKGRVGH

MVECTRQTVRALRDAVARSGNEVEIGAHMARLAGDVIARTEFDTSYETGKRIFLLIEELQ

RLTARSSRYLWVPGSQYFPSKYRREIKRLNGELERLLKESIDRSREIADEGRTPSASPCG

RGLLGMLLAEMEKKEAGGNGGGEVGYDAQMMIDECKTFFFAGHETSALLLTWAIMLLATH

PAWQDKARAEVAAVCGGGAPSPDSLPKLAVLQMVINETLRLYPPATLLPRMAFEDIELGG

GALRVPSGASVWIPVLAIHHDEGAWGRDAHEFRPDRFAPGRPRPPAGAFLPFAAGPRNCV

GQAYAMVEAKVALAMLLSSFRFAISDEYRHAPVNVLTLRPRHGVPVRLLPLPPPRP*

>CYP736A1 Pyrus communis (pear)

MSPPEIAILILVFLTFLWSLLRLINVSSRQSRTLPPGPAALPIIGNLHMLGDLPHRSLQN

LAKKYGPIMSMRLGSVPTIVVSSPKTAKLFLKTHDTIFASRPKLQASEYMAYGTKAMAFT

EYGPYWRHIRKLCTLQLLCPSKIESFAPLRREEVGLLVQSLKVAAEAGEVVDFSEKVGEL

VEGITYRMVLGRKNDDMFDLKGIIEEALFLTGAFNISDYVPFLSPLDLQGLTKRMKRVSK

TVDQLFEKIIQDHEQVSRSEQGNHHKDFVDVLLSSIHQTLKPNDEEVYMLERTNAKATLL

DMIAGAFDTSATAIIWTLAELLRHPKVMKRLQEELQSVIGMDRMVEESDLPKLDYLSMVV

KESFRLHPVAPLLVPHQSMEDITVDGYHTPKKSRIFINIWTIGRDPKSWDNAEEFYPERF

MNRNVDLRGHDFQLIPFGSGRRGCPAMQLGLTTVRLALGNLLHCSNWELPSGMLPKDLDM

TEKFGLSLSKAKHLLATPTCRLYNES

>CYP737A1 Chlamydomonas reinhardtii

SWPAATVAMLGTDSVTFSTGAYHRSLRRLLGPCFSPQAVEGYLPSIQAICERYCAEWAAE

TTAAAAAAAPAATGGDSSAVIEQLPKLQKGARMLTFEVMSHVVAGFHFSPQQLASLSDAF

DVFVRGIFAPVALAIPGSNYAKASAARKVMVAALTQQLELLKGGSGGGGNGGGANGGGDG

DSDLAINLLFAGHETTATSIVRLMLVLRSRPDVVSRLREEQAAAVRQHGAAISGSSIRDM

PYLDAVVKETWRCHPVVPMVPRRAVRDFTLGGHDVPQGWGVVLGLVEPMRDLPAWSGLTP

DSPLHPSHFNPDRWLSGRSSASGNSSNSASSSALQQQDGTATADGDDVASAAAAASVGGG

GGAAGSGTLSSPMGMLPPQMLTFGGGGRYCLGANLAWAELKVFVAVLLRGYDFTSPLPEL

EVKLFPALTVAQGFPIEVRAR*

>CYP738A1 Chlamydomonas reinhardtii

MRSSSRGAKIGRAYPTAHHIDGRASGGRPLHFGLHPCHRPCLRAKAAQSGLAELPLPEGS

LGLPVVGETLELITNGDTFGTSRRERYGDVYKTNILGAPTVMVAAPMARRYACICFRFSC

QVTSTLVGPDSLNLLTGPRHGAVKRALSDAFADRALRRHVPAIAELVQAVFDRVVLGGAG

SRDRAAQLQAVMSALQAGFNTPPVQLPFTAYGKAVAARQEFGQLVSQSIQRSRQHTAASA

TVSVSPSSAPAFDCAMSDVVAAAAAAAATGTALPDSLLVDNAAAAFFGNASTGPSLAKAL

QHLATNAAGPNGGATGGVMAALRQEQDIVSRHGPAITAEALDEMSYGTAVARELLRITPA

VPAVFRLALVDFELQGRRIPKGWRVWCHVGDSVTRYNKDQFQPERWLGSSGMAAGGCPMH

AGGGGAARGAQPEYSLPFGSGVRTCLGRNLVMTELLVVLAVLARGYEWEAVNPAEQWGVV

PSPAPKEGLRVRLHRRL*

>CYP739A1 Chlamydomonas reinhardtii C_

MAVFGFRELFASMYIPGLSPVLSTITCLAGVLLFLAWQRHSRATSVPRLGPLLTIPLLGD

VAWLAADPTRFVFGRFQRYGPTFILNLMGVPLYVLTQPADLRGPYRDQGAEPDVPFSSFR

RLMEVAPGRPYDVQADKAAHGPWRRMFLSALGPAGLQALLPRAQAVMQAHLAQWEAAGTA

AGGRSGGGCIPSLFRQVRLLSVDLAIEVIAEVPLPPGVERIAFREQLLCFLDGLFGLPLA

LPGSSVARALAAKEELVAALGPLVAADRQRMAKRWRAAGSSYAALVDTLTAASAAVGGSA

AAEAAAGVQAAEPSAAAAARVTVRDAVISGFMALGRAAAVSVLHAVVAGADTTRFALFNT

LALVAMSARVQEEIFAEQERVVAEHGPELSARVLGSAAITPYLDAVVREAMRLLPATPGN

MRRLTADLRVGAGRGGPASELVIPKGSMVWRFVPLMHCLDPVLWDGDTSVDVPAHMDWRS

NFEGAFRPERWLSEDTKPKYYYTFGSDNHLCVGQNLAYMEVKLLLAMLLRKYRLQLHTPD

MLARASQMFPFVIPRRGTDRVLLEPR*

>CYP740A1 Chlamydomonas reinhardtii C_1080041

MAPLLDAKQLELLGIGMQLAAVLLVLYYLLKWLAGKRGGVPGPAFYLPAIGETLSLFASP

TRYMWKNWLEYGPFFRTHLLGYPLYVVGSPGLLKPVLGDDSAFEFFVPGKTFTMLISDIR

HMQVPEQHAVFRRRLGQALNPGALSRHVMAPLRVVLERHLDAWEAAGRVQLAEACAAASL

DVALEVLTGVPLPAAPETRAEVRRGTGGLFRTALAGLYGVPLPWLPGTAIHSALRAQRRL

MALLGPELDREVAELAGKSRLPTGGTAWHETHLAHARTPRPGSAACPRGPTADAGSRRSH

RHRHHQLLLRHRGAHAHAGGPGRCGPALPHRHAFLRTGTPLSLTKEQIFERALGVVIASD

DTSKHLFFFELVAAAMLPGVWAKLEEEQKQAMRKYGDELSYSILNDMPYLDAVIKETIRV

FPTAVGGFRRALKDVPVEGGQLIPAGSIVFYSTHLLNAADPALLPRSLAPEALEGPTGLP

AHLDYECRLEEAFRPERWLSDETRPRQFAGFGGGQHLCLGMHLAHAEARMLLALVVRRFH

LRLEQPQLLSRVTYFPGPVPRKGADGLVLMPRRLEP*

>CYP741A1 Chlamydomonas reinhardtii)

MDGFWKTLGLGALLSPVLYALYLASLIVIPYLKSLPLRRKLRHLPGPPVTGFFLLGNVPD

LVRTPVHQCMARWAEQYGKIFKLELPTMTAVVLTDPEAVSQVLKVDRFEKLTTSYQNMEK

LTAEQQPNILTEPLSAYYKAVRRAVTPAFSTANLRRFFPLLLDITQQVMTGLAAAGPSAA

LDLDRVAQRLTIDVIGRFAFDRDFGATADIAKTNEALQVVGELMTALQRMLNPLNRWFWW

RKEARGLWASRRRYDALVRRALEDLRSSPPAQHTLLHHLMSLTDPDTGKPLSARRLRSET

ALFWIAGFETTAHAIGWTLMFIAGSPEVESRVAAELEGAGLLAVPGRPEPRQLAWGDLGG

LKYLNAVIHESMRLMPPTSGGTVRVVPRDTQLAGHVLPKGTMLWIPFYAMQRSERVWGPD

AAQFRPERWLAAAAGAGGPGARGFLPFSEGPRNCVGQSLALLELRTALALLCGSFRFRLA

DDMGGVEGAVSEARQHITLKPGDRGLLMHAIPRVPA*

>CYP742A1 Chlamydomonas reinhardtii

MHTAPRRIHAARCRPLHASTGASTPGPAGAPDLPPLQRAPGPPGLPWLGQLPAYLATKFF

PKKMLEWSEQYNGVYAMEIVGRKYLVVTEPSLIAGIVGRGSAGLPKSTGYAMWDSAISPH

AGVQGLFTVAENTTTWRAVRRAYGPAIGPGSMSSGTSTSTSSSSTASINSTTGLTSHEMN

HLAKCLTLDMLGLSAFGIDFRCLDDPAAAQLPSLIESAMHECGERARSVGRRLLPWLYEE

EARAGAADMAAFHALVEDVWRQIRARGAPTEDDNSFGAQLLRLADPSLAPGGAALSDEQI

CAEIATVIIAGYETTANTLTWMLYGLHAHKDASEQLVAELRGAGLVPDTSSSSSPSSVDP

TTASFASLAGAHEALGGLPVLDAYVRECLRLYSTAPNGLIKEVPKNGPPARVGPFAADPG

VVVWIPFWSLHLSNLNWEQPHDFQLSRWLGKDPRTAGSLTASRCPVSGTLNALRAATSSS

SSSSSSSSSSSSSSSSSSSGSDSDGEGGSSSGGRGSKAIRFMPFGDGSRNCVGQHLGMLQ

LKLSLAYLAARFDLVLDEARMGGSAAAALERQRVNLTLEVDGGMYLLGASVHSHARVYWY

QLVSCEPKC*

>CYP743A1 Chlamydomonas reinhardtii

MLRALSCLALLAAGAARLAAAAGATDSAVSRALAVLALLLALHVLADPLQRWRLRHIPGP

PALPLLGSVPAMMRAGGPFFFRQCFAKYGPVFKVAMGRKWVVVVADAELMRQAGQRLRSH

VIIEPNLNRGHLRRLDAEGLFQAHGEFWRLLRGAWQPAFSSAALSGYLPLMSACGLRLAQ

QLQAGGGARPAAGYVDVWRALGGMTLQVVGSTAYGRLAVACGDVFRFGSALHGSSYQRIG

LLLPELVPALVPLAHSLPDPPFKRLQRARSTLLAACMELIRSWRQQHHATT(largeins

erthere)TRTAGGTTATGVAAAAEAPAAMCGAAVPAAAAAVDGAAAPAGPEEADAAARG

GGVGGGGGDGSGVGGSGVAAGSFLDLMLAARDKANGAALTDRMVAAQVQTFLLAGYETTA

NALAFAIYCVATHPEVESRLLAEVDAVLGRDRPPTESDLPRLPYTEAVLNEAMRLFPPAH

ATTRIVEAGAPLQLGGVSLPPRTPLILAIYSAHHDPAVWPRPEDFIPERFLPASPLHSEV

AARVPGAHAPFGYGSRMCIGWKFAMQEAKLVLALLYQRLLFRLQPGQVPLPTATALTLAP

RDGLWVRPVLRRAARAE*

>CYP743B1 Chlamydomonas reinhardtii

MVASASWQLDLLGALSGAPSPQMAAAGLALLLASLLIYLLDPIQRWRLRKVPGPPARPLL

GCLPQLRAQPMPLFLQSCAQTYGPVFKASAEVQGIAVIPHHVSRMQVALGRKWAVVLADA

EMQRQVRGTGAERGGSTWRQLRAAWQPAFAPASLAGYLPLMTGCADQLARRLEAKATAAA

GATASGATAGGGSSVDMWRELGGMTLQVVGSTAYGVDFHSINEEDQAGSGSGSGSAIATA

GATAAAKGRGDDGYGKQLAAACGQIFRYTSSAHGSPYLRVAMLFPELRRLLVPLAHTLPD

KRFAILMQARNRLSGAVFQLMDSWKQQHIAAAGSGAAGKGSSGKADASNGVGAAATSGRG

GMAGVAPGSFLDLMLGHRQGGGSGSGGKKAEGEEGVEHAPLTDEQVAGQVQLFILAGYET

TANALAFAVYCIATHPEVESRLLREVDDVLPGSDQLPGESDLPRLAYTEAVVNEALRLFP

PAHLTSRVVPPGETLTVGGFNIPAGIPIFLPMYIAHRDPAVWPRADVFLPERFLHPRGAA

QQHAHAPFGYGSRMCIGYKFAMQEAKVALATLYRRLTFTLEPGQQPLQVEASLTMAPRGG

LRVTPVPRRKL*

>CYP743C1 Chlamydomonas reinhardtii

MTFLQLLPGVPLVLLGVLALPVVITLVQEVITKRKYRHIPGPKPQPISGNLREFLTSPGG

LLGCLEGWVKQYGDLLTFRLGSRQFVLVADPDAARPVFTARVFLTQIVFPHTARSLRGYQ

ALMDREAVALAGRLRRQAAAGGGGGGGGGGGGGGGDKAGEIEVMSEMSRVTLAVVGTAAY

GCNDFFRTMSPAARSSWSWAVALPCLLPAVRHLAAAAPDPVLALHIQSRQVLRQVSTKLI

TAWRDSHTAASANGSSTNSTSGSSSSTGVAPGSFLGLMLAARDRSRKEGGAAATAKDGMA

PTLTDAQIEAQVQTFLLAGFETTANALTFAVYLLACHPEIQGHRIPAGSTLWLSIAHLHT

RDGVWPEPQAFRPERFLSPDVPGSAPELAARHPHVHLPFGSGPRMCIGWRFAMQEAKTVL

SRLVQAVDFTLAPGQAAPLDTVAGLTLAPRNGVWVRLSPRGGGGSGGGGGRGQEVATAAA

KGAAVRSAAA*

>CYP744A1 Chlamydomonas reinhardtii

MALSSAWALAGLFLAMFVFFGYSLRKRWQLRKIPGALGWPFLGSIPEFSIYGYEYVLGLS

AKLGNAWLGVEPLIIICDPALIRKYAYKCVSKPPSMSEYGHVLTGFNYDVDQASAFVASG

EVWRRGRRVFEASVINGVRLAAHLPAINRCANRFVAQLAQRVAAPAAAHSGKTLGEEGID

MFSIVGGYTMAVTGEVAYGHVPAVTRGVRPFWQVEHSTLYLPLGVMFPWARPLVRWLATH

FPDRAQREHMAARTQIIANISRLLMERWATSKKAAAAAAGTGTGTGTAITADSKAGTASA

PPAEAARADGAAAAGKGAEEAIKEVGGGISSSSFMAAMMEGRRGAPQEERLSDVEVIAQS

FTFVMAGFETTALTLSLVTFMLATHPEAAARLTAEVDGLGPGELTHEVLAEKLPYTEAVI

KETLRLHPPIPYFIREAREDLDLGNGMVAPKGSYLTMYMHAVHLNPDVWPHPERFLPQRF

LPEGSAAFGPADPGAWAPFGIGARMCVGHKLAMMMAKTLLVRMYQRFRIELHPRQPLPLK

MKTGLSRVPVDGVWVTLTER*

>CYP744B1 Chlamydomonas reinhardtii

MELVSGLALAGVALFILGFIWAGFNPIERYLSPLRRFPGPAPLPFLGNLVSVATRDLTAY

LADCRQAYGGIWLGNQPWVCVADPDLIRRVAYRVLSRPFSHTDSIHLLAGEQWEVDCNTL

VFLKNGPTWRLARRAFESSIIHPQSLAGHLPAVWRCVRRYTPRLERHAATGEPLDLSSDL

ADLTLAVVGEAAYGVDFRTTDEQQDGGRPADPSAPGPALVAAVRECFDCLDVNKTTMYGP

LKMIWPGLTPLWRWMAKHLPDAAQTRHMRVADVSRQLMAQWQAAKAKTAAAADTAGATAA

SGAGAEAGAGVGVGAGAQAKPGGGGAVQAFVEVGGGISSSSFMASLLEGRRGAAKEEERL

TDLQIVAQCLTFLLAGFETTAATISFTAFCLATHPEAQARLLAEVDEHFARQAAAEQQQQ

GQQQREGDDALPELPYLDAVLKESMRLYPAGSALIRKSPQPLDLGRDGLVIPGNTFVCLA

THAVMHDPAIWPEPEAFRPERFLPEGSSSLGPMVGGAAASAPAGGGADAAAAAWVPFGMG

PRMCVGSKFATMVSKAVLLQIYRRFTFELHPKQVLPLRTRTALTHAPRDGIWVVVKAR*

>CYP744C1 Chlamydomonas reinhardtii

MQLTWLGWAPVTRWRLRNIPGPFALPFLGHLPAISARDLVHFCHDVARQYGPVWVAARPW

IVVSDPVAARKIAYRSLARPSTVASFTHALVGEPRQVDDESIFWNRGPAWKASRRAFETS

VLRPDRLAAHMPAVRRCTERFLARLAPYADGSTAVDMKDEYGVIALAITGEVAYVSFWPS

DEDAALLAAPTGGSGAATSSSSSSSSSSKSPSSALVRACHECMACFELPLATMYLPLQML

LPALRPLWLALAAALPDAAQRRHMEARQAVADVSRRLMREWQQQAAARANDSGGDGLLLK

DQTPVVNGGSSSSGSGGISSSSFLAAMLKDQTGSNTACASSSGTDGGVISQGLSFILAGY

DTTGTTLALTTFLLAHNPTTQEKLRAELVENRELLDSADGLAQLPYLDAVLKESQRLHPA

VGHFWRDATSDIALPEMGGLVIPKGSFVSISIYNMHRDPAHWKEPERFIPERFLQATGGA

LGPTDPGAYVPFGSGPRMCVGYKMAIMVVKSVLAGLLLRYRVALHPRQPLPLRLKTGLTL

EPADGVWVTLQPLLLPGAK*

>CYP745A1 Chlamydomonas reinhardtii

MASSSSPLEELLAFAGVKDGTISSPRLALVVLGAALAAYALVFAVINVVDYIRIARGLSA

IPSAPGGVPLLGHVIPMLTCVSQNKGAWDIMEDWMDAKGPIVKYNIAGTQGVAVRDPKAM

KRIFQTGYKLYEKDLKLSYRPFLPILGTGLVTSDGALWQKQRMLMGPALRVDVLDDIIRI

AKKAIDRLCEKLSHHAGKGDIVDIEEEFRLLTLQVIGEAVLSLGPEECDRVFPQLYLPVM

NEANRRVLRPYRMYLPTPEWFRFSSRMGQLNGFLIDLFRRRWQARQAAAAAAQGEGSSSS

KPKPADILDRIMEAIEESGAKWDAALETQLCYEVKTFLLAGHETSAAMLTWSTLELAAHS

QAADKVVEEARAAFGPRGESEAGRRAVDEMIYTLAVLKEGLRKYSVVPVVTRVLAEDDPQ

GLLGYPLPRGTMVACHLQGTHRLYESPDEFRPDRFMPGGEYDQFDDADRAYMFLPFIQGP

RNCLGQHLALLEARVVLGLLHARFSFKPAPSVHPDPASLFMRHPTVIPVGPIRGLKVLVE

QRK*

>CYP745B1 Ostreococcus tauri

IFQRHCVVVADPELVKRVMQTNLKNYKKDTEFSYEPFLEILGTGLVTSEGETWRAQRQRI

SSALRIEILDDIIAIATRALEKVRGKGEAVELAEEFRLLTLQVIAEAILSLTPEQSDEVM

PNLYLPIMDECNRRSLEPWRKFLPTREWREHRKRVAALNKYIVDLIRVRWKKRVSGETNP

NPDILDRVLASVEMEEYGSDVEEQMCFEIKTFLLAGHETSAAMLVWTIYELVKNEDKMTE

AVAEANKVLGAVKPGNLPTRDELAHLDYCVSALKETLRLYSVVPVVTRRAVEDDVLGGCK

IPKGTTVIISLQGIHHREDLWPNAMSFEPERFLNGKGDEIGNYAYLPFIQGPRNCLGQYL

ALLEARVVLATLIRRFKFKSASANNGKKHTKAIPIAPADGMWFTVE*

>CYP746A1 Chlamydomonas reinhardtii

MLALAGGLQSMLQVSSPLVTHKITYGSLRLSSPPPPAFPAGPSGDQTLPLLTDPLRFLTD

ATATYGPVVGLLLGGERVALVTGRAEARAVLVEAAGEVYVKEGTAFFPGSSLAGNGLLVS

DGPVWQRQRRLSNPAFRRAAVEAYGGAMVAATEDMMRRVWGPAGGTRDVYADFNELTLQV

TLEALFGFSEDAAQIVAAVEKAFTFFTQRAATGFVIPEWLPTWDNLEFAAAVQQLDRVVY

GMINRRRQELAAAFAGVPSDLLTSLLLARDEDGSGMSDQALRDELMTLLVAGQETSAILL

GWASALLAAHPEVQAAAAAEVAAVCGGPEAGTPTPASVRHMPYLESVVLETLRLYSPAYM

VGRCARRDAALGPYVLPAGTTVLVSPYVMHRDPEVWEEPEVFRPERWQELQRSNLGPNGA

YLPFGGGPRNCIGTGFAMMEALLVLAALLQRYSLALPPAAGSSSGGAFPKPKPLLTLRPE

AVVLRISPRRQ*

>CYP746B1 Physcomitrella patens (moss)

MGSISAANLELIATLASHCLQRTTESVKQGQAVLQQAVQPLPSCFPPGPNGDVALDFARD

PLECLASLKSRYGSLVGFKLASRPIVLVSSPNFSREVFVTQSSTFIKAGTAFFPGSSLAG

NGLLVSDGDIWKRQRRLSNPAFRRAAIQTYAEQAMVNITEKMVDKVWRTGGVRDVYADFN

ELTMEIVASALFGASEASEEMAQVGPAITQAFQFFTRRATSMFIVPEWVPTFDNIQYNNA

VTDLNKVVFRLINERRRQLANSSAPPRKDLLTRLLHVNEDGSGMDNQSLRDELMTFLVAG

QETSAILLTWALLMFALHPHTQELVFQEISEVLNGQLPRQTDVSKLRYLEAFIWETLRLM

PPAYVVGRCACHPTELGGYKIPQGTTILVSPYLLHQDPAFWPRVSEFDPSRWMPGGDATE

HMENDSFWPFGGGPRNCIGMGFAMMEVTLVLAVISSRFRVSLPVGEPIPSPRAMITLRPE

SEVKLRLTSRRQQRQRKSEAADEMKVIVCLN*

>CYP747A1 Chlamydomonas reinhardtii

MKSALSAFVRDSGDQVAETGAPTATRPIPGPAPLSLEALKDVSVIFFEGLHVAQLKFSEK

YGPVCRFANPASLNGATSWVFINSPENIQHVCATNVRNYSRRYLPDIYTYVTHGKGILGS

QDEYNARHRRLCSGPFRNKWQLQRFSSVVVERSKRLVDIFSAAAAADPSGAFTTDVATQT

QRLTLDVVGLVAFSHDFACVEQVQRRDLAGATAGDGRSGVLQDRVLWAVNTFGEVLAQVF

ITPLPLLKAMDRLGAPHLRQLGEAVSVMRAAMLDVIAATEDDGRGLSDEELWEDVHDIMG

AGHETTATTTAALLYCISAHPHVRQRLEEELDAVLADGEAPTYESLERMPYLQACAKEVM

RLYPAIPVFPREAARPDVLPTGHGVAAGDVVFMSSYALGRSEAVWGPDVLEFDPDRFSPE

REARQHRFQWLPFGAGPRMCLGASFAQMSVALMAATLLQRFRFTPLAPCSPLIPVGYDIT

MNFGPSGGLRMRVAPRQRGQQQ*

>CYP748A1 Chlamydomonas reinhardtii

MSSALDELRFYGTLAATLLGPRYDLGRVPGPPGHPLLGNITAVMRPDYHVQMLEWANTYG

GIFKFSLGFQPVVVVSDPAVAVQVLGRAPGRAIPRKCVGYKFFDLATNASGAHSFFTTSD

EGQWAAVRKAAAAAFSSANVKKAFPIALRHLLLLSLLHVFVEALFGVTPEDFPGRQVAAD

MNLVLEEANSRLKVPLSGLARAVTQPVVGWREGGTGHVSRGFGARNSRAWGSGEKEWTEE

NWEPRAVTDLWACLGRVRHPRTGELLGRQGLVPEIGALMMAGFDTSSHSVAWALFALAAN

PEAQQRVRQELDGRGLLRRPGTAAPPRLPVLDDLPQLPYLNACIDEAMRMYPVAATASVR

EVTEPTRVGDFVIPPGVIVWPMLYALHNSVHNWDQPDVFKPERWLQSNAGGSSGKGGGGG

KRYMPFSDGMKSCLGQALGLMEVRTALVVLLGRYAFALDPGHGGEAAVRRSMIMSLTLKI

RGGLRLVATPLG*

>CYP749A1v1 Populus trichocarpus (cottonwood)

MANPLIYFSGCLFLSILIILIKFFNKVWWTPIRIQSLMKSQGIRGPSYRFLHGNTKEIST

MIRKTRSSPQELLHHTLPMVHPHFYSWIKLYGMNFLQWYGPQAQLIITEPELVKQILSNK

DRAYPKTKVSNEIKKLLGDGIVLSEGEKWVKLRKLANHAFHGESIKGMVPEMIASLEIML

ERWRHHHSKEIDIFVEFKILTSEVISRTSFGSSYLEGQHVFDMLTRMTHIISENNYRVRI

PGIGKFVKASYDIEFENLEAKIRKSFMNMMKRREKDAMLGELDGYGHDLFGLLLKAYHDS

DETKKISLDDLIDQCKNFYLAGQETSASALTWIVFLLAVHSDWQDKARKEVLELFGLQIP

SQDRIAKLKIMGMVINESLRLYTPNAILMRRVERETKLGKITVPANTEVYISTLAVHQNP

EIWGEDALLFKPERFADGVVKATNNNIAAFMPFGLGPRNCAGMNFAITETKLALSMILQR

YSFTLSPTYAHCPTEVLTMCPQHGVQVILQRYEHIALKV*

>CYP749B1 Petunia hybrida (petunia) DQ099540.1

MMTAICFVFLVGLALVLARFLYKSWWYPVSLQLLMKSQGIKGPPYKAPNWNYAKGVLDME

VKSTSAPMEISHDIIPRLFPQVYSWINLYGKNFLHWIDTQPQLVVTDINLIKEILSDKEG

SFDKVQLEGVLKKFLGGGIVFEEGKKWSKLRKVANHAFHGQNLKEKVPAMVASVEELLKT

WKSYEGKEIEVFKEFKLLSLEIISKSVFGSDYLTGKTMYHMLDEIVLICYKIIADKFSKS

SHELKVADHILQAFVDSLVGIMKQREDKVKAGQSNNFGSDFLGSLMESHHNTDQNKRISV

VEIIEECKTFYFAGHETVRSVLSWSILLLAVHTDWQDTARKEVLEMLGQGNPNIESISRL

KTVGMILNETLRLYPPLVFLHRKVKRNIKLGELRLPAGMEVYIASLAVHHNSEIWGEDTH

LFKPERFAEGVAKATRDQLMAFLSFGFGLRKCVGFNFAQMEVKIALCMILQRYRFTVSPN

YRHFPTLVMGLWPKHGIQIMLHPL

>CYP750A1 Pinus taeda AY779539

MSFDKLLQALPPPLPLPAILIATFIFFFSCWILHQSQRNERLPPGPYPWPIIGNFHQVRL

PLHRTLKNLAEKYGPILFLRFGSVPTVVVSSSEKAKHFLKTHDLIFASRPPTSVGKYFFY

NFKDIAFSPYGDHWRKMRKICVLELLTSKRIESFKHVRQEELSAMIHSIWEESESGRIAV

NVSKAISTSLANILWRILARKKFSDNDLGADGKGFADLVVEVSIAVGSLNIGDFIPYLDC

LDLQGIKRALKKANARFDAFAEKMIDEHINASTIRNGEADAGCHVKDIIDVLLEMAKNDN

TGAKVTREIIKAITYELFSAGMETSANVLEWAMSELLRHPHAMKKLQQEIESVVGQQGTV

KESDLASIVYLHCVVKETLRLYPSLPLALPHESLEAVTVGGYYIPKKTMVIMNLWAIGRD

PSVWGADASEFKPERFMQMEENGIDLSGGQSDFRMLPFGAGRRTCPGSAMAILTVEFTLA

QLLHTFDWRVEGDPSELDMKEACATKMPRQTPLLAYPRLRLPRCP

>CYP751A1Physcomitrella patens (moss)

MAAWKRESVVSVFATNESAAIGVCDVTPVHETPLWRYVKHEQNTLAWVSLIVVTFLLSRR

LCSVLRLLILGYRLPGPRARAFDGRSQCDDIVELLARLHQEHGPLVKVWTGPAQLLVSVK

DVDILQHVFERAHDRVPVLRMALQLLYGRRSLFTSNYSKVSCRSLINGLVLRQAHISSIE

VAEKMTQLGGLSKNGCHDLDCMTFSKLMAFAALGTSLYGDGYMIWPVAREFERVMMEVME

ALPIWMRYSVPPLWNAKFVVFWKQCLRLRDLARELAAHGNQTSIQESEDRVEGLNILGKL

LEEFVSVLFSRMGMGSSTVAEPGAAGMMSHGSLNTAGVLCNVLAQLARHPHIQTKVHNEI

STISGFDKSLTETDVQKMIYLNATVLEAARLLPTVPFLQRCSDEHDIALLPGVVIPAGAI

LTAPIQLIQRDTVYWGDDAAIFNPDRFLKPRRIASGELASEQNQNIPHPCNFTKEPLQLN

PAFLVFGAGSRSCIGSSLAVKQISILVTVILKRFEVMLYPTLFISKL*

>CYP752A1Physcomitrella patens (moss)

MEKDDDVSFDVALPTIGLETTHSKFPQLVLSAIGLIVVCGGAYVLYNTHYLRVKIKLPPG

PPPWNVFCTSMQSKKPCQALAKICNCEYGGIMTLSLGKFPTILITSTVIATQLHVLKRYK

FKFGHPKNIPRPCEYLHPDNYQNLKCVLPYNWTQWHKLWQIYIDHLLPVAHNMSFQSINQ

LDIQIMLKNLENEMTKEGGMKPFGIGLRPHLRHASFKFIFNICFGRHVDAIAGGVGSHKD

PLMMQLEALFIEVIRLGPAFIISDFVPTSLPFHSPIDIQRAAISGTMKKYKTFYHRANIK

VPRSEPVDLLDHLVCLQKDEQLQDKEIVWLLSELILASTDHVSTILEWTFAHLMANPQVQ

AKLHQEIDIVCSKRTNISTTEFDNMPYLVAIVKESMRVSSPIMLTIPHSTTKELNIGGFQ

LPMNTQIVCHLGALGQDANIYENPSCFDPNRFIGIGVNLNNAFEKQKNIVHLMTQQFCPG

RGLEILHVYIFLVKLLQCFEFSHLYVEIMPFKTSDTVEWGVINVLRKPLVACLNPHL*

>CYP753A1Physcomitrella patens (moss) BJ976877 CYP753A1 BJ975911

MANYIIASNALVLLAFVTFFFVYFLRAFILRDKKLRPKYPPSPWKWPILGNLPQLLRGGP

ACHTTFRLLAKELGPVYNVWLGGSFPMVIVTGEETVHEALIKQSSVFSSRPKLLSWQHIS

AGFKTTMTSPFGPHWQKLRKTISVDLLGPSKLASYKPIRDSEIQKLLARLREQAHANAGL

VSPLDQLRTSAVDVIMRIGFGEEFALMEAVNSNRRHAKIVELDRCFRQLMDAGSIFQLVI

DSSVVARTLLFPLARSANRNIETVADNTVSLVMPIVQQRKRYLQDHPATETRTFVDALIS

CKGESALTDLEIVWNVVELMVGGTDNTSHILEWALANMVKYPHIQEKVYTEVRCAMGPNL

ERRLVEESELDKLPYLQAVVKESMRRHMMTPLAIPKLAAQDCKLSGYDIPKGTMVVFHAG

ALAMDDDIWTDPLNFRPERFLAGTGSSNAPVTQTHKHAFMPFGAGRRSCPGAAMGFLHLH

HLFANLIYAFEWGPESPRKAVDFTEKFRMVVTMKNPLRATIKERTHFRMM*

>CYP754A1 Physcomitrella patens (moss)

MVEESWLWVLFVGALSFSILLQWGLNRKRKLKLPPGPTAWPIVGCVFGLPRLNPPEKLFN

KLSEKYGELMLLQLGSWSIVVTSSARMAMEILKTHDNEFANRPDVISSRLNFNNTGLIQM

HSTNPLFKRTRRMFSAEIVSPRTVLETGVIRRKQLRTLRSIVQDFDAGRSVNFTHEMKTL

AMNLSMSICFGTDYATKVNDEAEALIHTYKIMAIWTRRSLGAIFPALRWLDLDGIESGFA

DVELQLRTNITALIEKKKQEMSMWSAEDIQAGANEGDVMTKFLSMEGEDRCSEDQLISVV

FTILLAGTDTVFNVVTEAMYALLMHPNFYHRAVEELDAVVGKSRLVEEADIPKLPMIQNI

IKETFRIKPAGPSLVPRKNFEACEVAGYHIPANTTVFVNCIPLMRDPSFWDSPDEFNPDR

FIDSKVTVLGSDFNYLPFGYGKRTCPGLNLGMITVQYILAACLQCISWKLSRPRRLDIET

DDDPRKVDDVMVDGKQRVDPALLEFAPQPVK*

>CYP754B1 Physcomitrella patens (moss)

MVGDVWIWVLITMVVAVIVGVGIDKKTKRGLKLPPGPPAWPVVGCLASLPAGHPPEVMFA

KLAEKHGELMLLWLGSKPYVVASSARMAMEFLKRHDQEFANRPMSVVREYVSFKGNSIIS

MSASDPKYQRLRRTFVMELLSPKKIAATRDLRKDQVLKMLRAIREDLDAKHEANFTEAVL

TLGMSLSIGLLFGRDYGGKVFSEEIQTLVLTFKTMVKYLSMINISDLIPSLRWLDLQGIE

RGLGLGEVQLRKSIMALIEQKRLDKIRLSSDEIESGACQRDILSKLLSLEGEDRLDDDQL

MGVVFALMLAGSDSISRGVGRAMQELLKQPLLHQRALDELDEVVGRRRLVEESDISSLPL

INNIIKETLRLHPPAQLLIPHGNVEQCEVAGYHIPARSTVLVNLYALSRDPSFWNSPLEF

APDRFVDSNLTVQGSDFHYIPFGYGRRGCPGLNLGMITVQYALALCLQCILWRLPAGATI

SETYIDWKNSPDLIVDGDLRVDLHLLEGL*

>CYP755A1 Physcomitrella patens (moss)

MVFTVRREVADWSHSFPPMSYGTAVVVGFVLLLLLLVFGYSRRVGKKKTLPPGPFAFPVI

GNLFLVGKHPHVTFAKLAKQYGNIMRLHFGAVPVVIVSDANMARELFSVQDMKFASRPIY

DLMSTAYKYMNYGTDEEVSLAISEYGPKVRDLRQLCTTELFTQRKIDMKKSVRAEEIQRM

FGKIKTMIRDEEPVEIRPIVSEFSLRISCRTTFNKAFLNFENLPWRPGALHPQAFRNMET

ENTKLLGEHQILD594MIPMLKFVLERFDVFGINARWKEVSALKEECTRPVIEWYRKHSS

DDESTLDFVEVLL423RLSEEGKLSKTCVKSLILELLTAGSDTIASVLEWTLLELVRHPH

GMERLSAEIDGFFGINRPVDEDEFTKLPYLQAVAKEVLRLHNPTTLGIPHSNMEEATLAG

YHLPARTTVLANFWAISRDPTTWGQDALTFNPDRFLACDLNVNGTNYEYLPFGAGR(2?)

RICPGRAVAMRVLAAAIGSFVHAFEWSALPGVELNANEGKDGLNIRPETPLVLKLSPRPS

AMLY*

>CYP756A1 Physcomitrella patens (moss)

MAHLHTRLSEEAEAWIATGVDSFSRWQEYAAGFGRATYIVAALGFFAVVILELHNSRKRR

LSKLPPGPFQWPYLGSLPNLLLTVGVTSSFRLREKVSELGRNHGPLMFLQIADTQILIVS

SGTAAKEVLIARDEEFNFRPQCAVGKYLGFGSSDIAFAEGRHHWYLRKLCDTRLFSADSF

VSYGHIPRAEALKMLHSVWEASKKGNGISVRETVTAFVRNSLCGMLLGSAHLDIENVSLQ

FTEKTLITLLDETICVVGEITLSDLAPGLKRVDFHGRTRKLKELHERWEKYLRVILEDRS

HRLEKSAKPEALVDVLLSLDDADMKLSNEAIMGVLLDTLVGGVYSTSATIEWALAELVRH

PGVLEKVQLEMSEVVGPYHIVEDAEISQLPYFQATVKETLRLHPVVPMSLPHMNKVATSI

SRYQIPANTSVVIDYKAIARDPAAWHKPLRFDPSRFLHTSAASQIDNIFKFLPFGYGRRG

CPGANFAAVLLQLALAHLIQAFDWAPTKGQLPHDIDVKESPGLVCFRFSPLVLSSTPRLA

NSLYQVSP*

>CYP757A1Physcomitrella patens (moss)

MMEIGGMRAEWHVVLSACVTIATMVLTIMKLRKKIGKLPPGPRALPLIGNIHQIGDFSRR

NLMQMAEKYGPIMYMRIGSKPLLVVSTAEAAHEFLKTQDKEWADRPTTTADKIFTNDHRN

IVCAPYAAHWRHLRKICTMDLFTPKRLMSFRTPRTEEINQMMTSIHEDVAAGKEVKLHVK

LGHLTTNNITRMLLGKRFFTVDEKGQMEAHRFKELVFELFRASSTPMIGDFIPWLKWVSI

ASGYVKYLKRVKADLDAFLQEFLEIKKAASDQATAERAKDFVDLLLEQKTVSGDGPLEDA

TIRSDMLLAGTDTVSNAMEWTIAELMRHPECMRKLQQELDTVVGKSRIVSETDLPNLPYL

QAVVKEVMRFYPPAPLSLPHQSIVPTTVCGYDLPAGTQLCINLYAIQRDPKYWPNPVQFN

PDRFLNCDVDVGGTHFQLIPFGAGRRQCPGMPLGNLLLQISVARLVQAFEYSLPRGTKRN

YFMNYYSGANKLTSGIMYLI*

>CYP758A1Physcomitrellapatens(moss)MATPD

SSGGAFDLAKWINGLVAHWGSVAVAVVAAAVIAKFIFNSTVGRRKLPPGPAPWPILGNIA

SLAGLPHRSLEKLARKYGSLMYLRLGEVPCIVISSADVAKQLFKTHDILFSNRPGGCFFE

QLTEYRNITASRYGPHWRHLRKTCVHELFTQKRLEAYQATRLEEISISIKELFEESDKKG

PVDLHAWLHRLLFNNLTRVIMNNRYFGTDEKGMKDAMDFNNVTALMFSQAGDVVISDFLP

YLGFLTRLQGKPLLYRKTREIVLEMMRRMTNFDERKKLHAEGRSTGEPEDFVDVLLSSTL

SDGTTPLPDDICLMLLMDVLVAGTDTSATTVEWTITELLRHPEAYKRVREELNSVVGSDQ

LVKEEHLEHLPYLNAVLQESFRLHPATPLGLPRESSEAFEFLGYSLPAGTRLFVNQWAIH

RDPAVYEQPEEFNPERFLGREALKFIGDTQFQLVPFGSGRRNCAGLPMAVIVIPLVLAHL

LHSVEFSLPDGQQPKDLDMTETFGVAAPKASPLMIYATPRESAALY*

>CYP758B1 Physcomitrella patens (moss)

MLATAFLVGFLAWAAMILGKFILEGIQRRNLPPGPWAWPIVGSLFSLGPLPYKTLRVLAK

KHGELMYLRLGSIQSVVVSSASMAKEVVTNHDLQFAYRPTKLFGKLLFNSKDIVHASNGP

AWRHLRMICTSQFFTKKRLASYEATRTFEIHTLMKDILRKSSSEDCVVNLPFQLRNTSTN

FISQMVFNKRYFVEGEESNVEDAKRYQKILKIHFSSYAIFVVSDYIPCLRFITKLQGIRG

KFQQIADKIHKKMDEIIDINGHERRRIDANHKQDADRKKDFVDLLLETTSHDGKGTLDHE

TVRGDMLFAGAETQSSTLEWAMAFLIRNPGVMKQVQAELDGVVGTERVVQESDLEKLPYL

EAVVKEVMRVKPGAPIGINHESREPRQVAGHYLPAKTRLIFNIHAIHRDPSVYDRPDEFD

PTRFLSPGKGNVPTGQELFQLMPYGAGRRICPGMPLAIVNIPHVLAHLVHSFDWSLPAGQ

DHRELDMTEKFDGVTSPRLHPLHLIPHPRKPAFLYK*

>CYP758C1 Physcomitrella patens (moss)

MMDSASYSAPFAALWDTFGRGTVVAVLVVVVVGELLLYARFQAQRRSTLPPGPRPWPILG

NFFVFSDVNHAHHDLRRLAAKFGPLMYLQLGSVPCVVVSTAEAAKELFRGHNDECLISRP

KMLGLEILSDNYQLMAYAPAPGKLWHSLRKFGSMELFSFKRVAFYRSLREEELRHWIKFV

LESREGEAMNLKSCVFELAANMMTRMLVNKRMFDITGADTQQQLLRSEFESFMEEHYKCL

MPNVISDFLPFLRFFCEKLQGWRAYIQDHQEKSVEFWTRIIEVEKHRQRAAERQNDGSYV

PDLVDFMSTAPLDDGKVLSDRNITLQILDFFLGGTDTTPLTLEWAMAELVTHPNFMKRAQ

EELDRVVGLERLVEETDFPNLPFLQAIVKETYRLHPVGPLGGPRESTEPVEALGYKIPAK

TRVILNIFAIHRDPAVYERPDEFDPTRFLDRPLAAFDSYELMPFGVGRRMCPAFNLGNTT

VHLILANLIHNFDWALADGQNIDTFDMTERLHGVTFSLKYALSLIPTARSGILARAL*

>CYP758D1 Physcomitrella patens (moss)

MGFVEMTQNWRLWLQEGSNVSVYGTVLFVMFTTSCILHVLSAIERRKKLPPGPWPWPFIG

NLGVVLRKTGARHKFLQALGAKYGGLMYLGLGQIPCLVVSSVRVVESMFKSHDATFSDRL

QTYFRKVQYGDSAMRSLSSAGYGSYWRQVRRMCNTELFSPGTHASQEGVRREEIQNMLDV

LVHECKRRKPIDLGDWLFGVSTNNMTRMLINKRYYGTGAEIPEKKEEFQGMVKSRTRAAG

TFVISDFIPSLTFIAKLQGLPKRFRESHESAKAQMESVLDVEEHRKNAIARASVDIKSEY

SPDFVDVLLKAPLDDGQPLADSDIKFLLTDLMIAGTETTGITVEWAMVELMLRPELRKQA

QEEIDAVVGADPERFVQESDIQKLPFLVAILKETFRVHPVAPLNVMRSSYEPCEFAGYYL

PAQTRLIVNQYAIHRDPSVYENPDKFEPRRFMENPEVNPLSGRDSYQLIPFGVGRRMCPA

SNLAFTMALLMLANLLHTFDWSFPDGVTADNFDVSEEFLGTVLRKKTPTILMAKPRSHVQ

*

>CYP758E1 Physcomitrella patens (moss)

MASAHSHTRRWWSQEAHGIRVSGEGTIATLLISSLVIYVTVVYQRRKKLPPGPWPWPVVG

NLAVLAGLPHRNLQNLAAKYGGLMYLQLGQVPCLVVSTAAAAKELFRTHDVIFSYRPKRL

DHEIISGKSYKSLTSAPYGPYWRQIRRICNTELFSPAIHASHVSVRSEEIHSMMKVLLAE

SRTEKAIDLKSWLTGVTANNMTRMLINKRFFGTGVSDQQEKKDFEEIFDHIFAAAGTFFI

SDFIPKLRFVEMLQGKIAKLTAFRKFLHSVIGKIFEVEKHRQRALERGNDPIYVPDFVDV

LLNTPLDNGERLTDREIISILSSMIGAGTDTTATTVVWAMSELMVNPKIRKQAQEELDAV

VGDSRLVEESDIPNLPFLRTIVKETFRLHAPVPLSLPRCSEQPCEVAGSQFPANTRLILN

VFAIHRDPIVYENPDSFQPSRFVDHPEVDHMSGKDFYGLIPFGAGRRMCPGYHLGNVMVS

LMLAHLLHSFDWRLPAGVTEENLDMSETYKLVGLRKKPLFLIAKPRSPAYLY*

>CYP758F1 Physcomitrella patens (moss)

MAEGGLLFGFALADVLVAAVLISVVVLYFHAETLQRRRCPPGPWPWPVVGNFSALGDLPH

RNLAGKYGGLMYLRLGAKPCLVISTAAVAKEFYTTVDASFASRPKRFSWTVWNNNDENYR

NIGLAEYGPYYRKLRRLLNTELFSPRRHASHEVTRAQEIQCMMKVLLEESEKGNPVNLQT

WLHGTTSNNMTRMVVGKRFYGVRVDDSEKERQDLQKMTSSVFELLGSVDLSDFVPYLSFI

TKLQGHASKFSKIRDVSDKLTADFFDLDSHRNNYKKMKNDPSYVPDFEDVLMETPFENGT

NLPDQDLLKLLQELLNAGTETSSNTSEWAMAELIRRPELIERAQTEMDSVIGSKRLVEES

DIQQLPFLQAVMKENFRLHPPAPLLLPHESREPTELLGYHFPAGTELLVNAFAIHRDPSV

YDNPDSFDPDRFLARPHVDHMSTSDPYELMPFGKGLRMCPGYRLANTMVALMLANLLYVF

DWSLPEGQTEVDMTETIGISVRKKQPLFLVPKPRFELSLESVAEN*

>CYP758G1 Physcomitrella patens (moss)

MGALDHSNDMWLQILLALTLVSVVLTWILQCSSSAQKVHPPGPTPWPVIGNLFLFFRAPL

PHRMLHNLAEKYGDLMYLRLGFTPCIVVSSPALADYIHKNHDTEFSSRPDGLITGILNGD

SQSVSMAKHGDLWKTLRSICWQILRPANIARYETRRMEEINIMLQSIQIAAEAGETVDLS

SMLYKLSSNSMTQMLINRRYFTAGGNEENLREAVIFKKMISERLKIASQFAIGDYIPYLR

FIDYLFRYNAKAQEIQSMTMRVCDEIMNLEERRRRLTREENGDAQAVREEDFVDDLLSIQ

AEYTADNSRKIKLTDHQIKLLVQDMLVAGTETSATTVDWAMAELLCHPKVLQQLRSEIVT

VVGSRSAVTEQDTKQMPYLNAVVMETLRLHPAAPLNLPRESKGACLFLGRYQLPAKTRVI

FNTHSIHRSLEAYDSPNAFKPERFLGVPQANVSGSSFFQLSPFGFGKRVCPGQALGTISV

CAALANLVHRFAWSLPCGLPPSHLDMIESFGLTAPRRCPLILLPTPRLKV*

>CYP759A1Physcomitrella patens (moss)

MEFNQLVSVAAVVVVLGASLVFLRLFTKKKLNLPPSPKGRMPIIGHLHLMDDNEAAHRTF

ARISEQNGPLTMIYMGNKPTLLVSTAAMAEQVLKHNDQAFASRPFITAGKTLGFDFKSIV

FAPFGNYYRRLRRIYTVELLSPKRVALSQVLRQHEIKHVINSVLAENQAEGRVNMTSILQ

EMGIDNLVRMIFAKPHMGATECLTKEEMATLKSVVKEAVNLAGVIYVGDFIPLLDIYDFT

GYKKKTNKLAAKMLDIATQLIEKHKSDAGTGVDNDKLNLVDILLSQKGEDQLPPHAMAGI

LFDFIIAGSDTTSVSIEWAIAELLHYPHYLKRAQEEIDQVVGKERLVTEQDIKHMPFLQA

VVKELFRLHPAAPLGIPHCNMEETKLAGYDIPAKNTVMMNLWAIGRDPAHWDDALEFKPE

RFLNKDITLLGRDFHLIPFSVGRRQCPGAGLGLAVVQLAVASLLHGFEWSTYNQKPEEID

MREKPGLVTPRKSDLIVTAVPRLPLHVYQGDKNGVQNGH*

>CYP760A1 Physcomitrella patens (moss)

MEDNRMGDGQVEEYSSRVMHLSALTLCAAMAVILLRRVMXSWNADKTLPPGPKGWPIVGS

LYSLGPRTIPACRRFTTLADKYGPVMFFRLGSRPTVIVSNDKMARELLRVHDQTFASRPK

LATGKHFGYNYSSVVFSPSGAHFVRMKKIYTHELLSPKKVELLSALRMEEAHILLVDVLR

NSGTEANGVVNITSLVFKANLNLMGRIVFSKRLFGESATISAPPREVENFKFFVKSATKL

VGLFNIGDYIPALRWLDLQGVEGALLQLKPHQEGLLRPIIQEYRKMSLNLEGGMKQKEDG

RVDFIAALVSNDSGLSDENIMAVAIDVMVGGSDSTSTAVEWSITELLRHPDCLQAAQEEL

DSVVGRDRLVEEADCANLPFLNCIVKETLRLHPPSPLAIPHFSAEECTLGGYRIPANTTA

YVNIYAIGRDAATWENPNRFNPTRFKDSKVNVYGHDFNLLPFSSGRRGCPGVHFALPTYK

LELANLLHCFKWSPPPGVDFKDIDTKEAVGVVCSRLNPLMASVTPRIPRHVILAK*

>CYP761A1 Physcomitrella patens (moss)

MVPAGYGGVSEFPFLKFAVAVLGVYFVAVLIRGASRKLPPGPVGFPIIGSVHLLGPRSHV

SLAQLARKYGAPLMSLYLGQKLFVVASSAEAAMEVLKKQDAVFCSRPPLRGFKVIFPHDV

TFADLTPESNYLRKFIRLHLTTARSIEAFQHIRVDEMLQMVRSIVASPRDVVVNLRTSLE

VMTANVLTRSIIGKRFMGRTGLSESEKKEIMEFIHIAAEIGECLGAKNPGDLIPALKLVD

WNGLDQRMKNLRRKMATFLANIVRERREKSSLGTSNPPGKEMLGVLLDEMENAAAGEKIT

EDILNTIIWESFTAGMETTVLATDWTLAEVLRNPEVLQKCQAELDAVVGRNRRAQESDIP

DLHYIKAVVKESFRLHPVIPLLIPHYSHDPIKVLGYDIPAHTQLLINVWAIGRDPKVWAD

PLKFHPERFLEGPHRETEMFGKSFNLLPFGSGRRACMGITLGTLLVEASVVVLLHSFDWI

LPAEGIDMTEGQGLSVRKNVPACAFATPRLPPHVYAE*

>CYP761B1 Physcomitrella patens (moss)

MPFSGGQGTFMFQGSAIAVVAIFLLARFITTPKNIPPGPFAWPIIGSLHLIGPYPHRSLA

KLAEKYGSLMSVWFGQRLIIFATSPETALEFVKTQDANFCSRPKQQAPSVLLPHDLTFSD

VTSHSKLLRKIFQQQFTTSKKMEATQQLRANEFAHMLRTIPHDTTVNVKFHLEVLAGNIF

SQLVMSRRLLQPSSIEDTTTDSTEKLKDLMKITADLDRIIGTFNPGDFIPAVKRFDLAGI

GCKFKQFRNRMDSFVEKIIQERLEERKSSRAPKELREKDYLDALLDEADQQKEIDLNVVK

TMIWEIFAAGMETNIASSEWAMAELVNAPHTMKKAQAELDAVVGRDRMVKESDLPNLPYI

KAIAKESLRLHPPVPFLAHQCIKSCKAFGYDIKSGTSVFVNVYGLGRLESIYPDPNTFNP

DRFLPGGSNVGLDYQGQNFELLPFGSGRRICAGMPVASLMVQTAVATXLHAFTWIAPKDH

ELMEGLGAASLSKAVPLKAHATPRLPSHVYSL*

>CYP761C1 Physcomitrella patens (moss)

MAAFAVPTLKNVYYVATVLVVILLVRRLLTWPHQPPGPPGLPLVGHMHFLGANPHISLWK

LADKYGPLMSLRLGNKPYVVATSPETAKEFLKTLDANFGSRHYSSQSQYLLYGGQDVAFQ

ESSPSWRNLKKIFTMELASPARLEASRHIREEEMIVLLRTIHSKGELELKSQLIDMISHV

ISRMVINKRFDDSVESDFPTLVQTHFRLAGAFVPGDYIPAVKWLDLGGFEAQMKKQKERM

DAFIDDILVQHRERRAKGPVPMKEYDMVHVLLDRIETKDDQIQLTDTHVKALVLDAFLGA

SETIILTSEWAMAELLRHPSLMAKAQAELDAVVGRDRMVTESDLRHLTYLNTIIKETFRL

HPAAALLLPRESAQPSQAFGYNFPAKTRVLINCYAIHRDPAIWHDPLVFNPDRFLQADLK

DVDVKGRHFQLLPFGAGRRVCPGLSMGILTVQFILASLLHSFDWSLPGDMKPEDVDMTEI

YGLTLPRAAPLPCAAKLRLPSHLLTTAQKP*

>CYP761D1 Physcomitrella patens (moss)

MASSSLDFPTFFIIGATVFAIFIFKKFLTKHSNLPPGPIALPVIGSMHLLGTSPHHNLQK

LSTKYGPLMSIRLGQAQCVVASSTETAMEFLKNQDSNFTSRPALRVGEAVFYGQDLVFQN

STPLWRHLKKIFQVEFTSTKRLDTTRHVREEEIAHLTSTLPHNCEVNLRIHLKSMIGNII

SRMAVGQRLCAKPEECESEEQLREVASLREVMDNVAFCIGAVNLADYIPALKWLDLQGLE

RRFKKTFQIMNSVSGEIIAKHQERRKLSNPTDKQKDLIDVLLDDMEKPQDGSPRVTMDSI

KAVTWNAFAGATDAIAMSLEWAMSEILLHPHVQAKAHAELDVVVGKNRRVEESDIQNLSY

IGAIIKETLRLHPVAPMLAPHAALNPCKAFGFDIPGGTWVIINAWAIARDPAVWKDPTEF

NPDRFMQDDPNALNPRVFEMLPFGAGKRMCPGVAMANVTMQRAIAKLLHEFWGLTSELDM

SEGTMSIVVPRAVPLHAVAKPRLSSEFYT*

>CYP761E1 Physcomitrella patens (moss)

MDFAKSTVARISFEGLKPEDGLSNQRVEIIVFLAAMFILPFVLLKLMRRPKLKLPPSPPA

YPIIGHLHLLGKLPHHSIANIAKTYGEIYSLRLGSVPAIVVTTPEMAKEFLLTHDKIWAS

RTVRDVSGYYLSYNHTGIAFAPFTPVWRNLRKICTSELFTQKRMEASQGVRDVEMQCMIR

SILNDANQRRLIDLKLEVNALTANVVTRMVLNKRFMRCVDSTAEEESRAQQFKEIMKDHF

TLQGIFMIGDYIPWLRPLDLGGKEKRMKALRKRLDAFLNEILDDHEVKRAKGPIAEEDQD

MIDVLLNEMHQQDPNEPHKMDLNNIKSTILNMFAGGTDTATITIEWAMSELLRNPPIMAK

LKAELDALIGQDRRVRETDVPNLPYLQAITKETFRLHPAGPLLVPHESTHDCEVAGYRIP

AGTRLFVNIYAIGRSSKAWDRPLEFDPERFMTGPDASVDTKGKHYRLLPFGTGRRGCPGM

SLGLLLVQFTLAALVHALDWSLPPGMDPEDVDMTEACGLKVPREHALSLNAKPRAAAQFY

*

>CYP761F1PPhyscomitrella patens (moss) Pseudogene

METSQLSDYWAGSQLLGNSSFGPGVRVDSVSGSQYFVVEFFLSAIVFTVFNLVFQRLHEP

SLIPPRLSAWNFLCQTHVLRRNPTVVLHNLVKRYGPVTHVKLWSQDLLVLSSVXAVEEFY

KLHDMEFGDRPSSMNRVTLSNSINSSCFPPLATYWKHLRFVLVSASTIPSFSSSFAFFRM

EWALEALHHHPSIVAQVSEEVERSLGSRSHIEDSDLAKLPYLQAVVKELFRLYPPCAFSF

PHESFDEYCHIFGYEVSPRTQVLINIYTIQRDPAVWTNPNEFNPTRFITHPGIDMHGQHY

QLLPFGGGRQCPATKLAIRYVQSGLARYFHDARSSHMIPHSTCLEDDL*

>CYP762A1 Physcomitrella patens (moss)

MATVSLQEPGLVVGLFLGAPLLLFLYILYYAISLHTTSVEGVRVPRGNFWLLPLLGESIS

ALTVPPKQFIDRQTRKYGAMFTTHIGGDPMIMTTDVDLTRWVYQQTNRLFSVLSPKATYE

LLGHESIFYAKGDHHLRLRKVFAGYLSTQKLVPFTPRIDKMAASIMESWKRKERVIVFDE

AKMYAIHLALAQLISIDTQEYPCMDHIFAHVPGENRLEKLVYLHYDIESGMMSVPLNIPG

TAYHKANKAKILFRKALKVIINERRTGDVKCNDLLEGLLSPLEDGTLLDDEQVMDNVITG

VGAAEVTTTTALVWMVKWIQENPELHRELQNEMDAIKKTKANGEELTYDDIKKMNLTLWT

MYETLRLRKVTGFFIARTADQDVRYKDVVIPKNWVVAMTHGYHLDPNYYPEPEKFNPYRF

QTMPPAHTFTPFGASVRLCPGKEMAKIEILTFMYHMLTSFSWEPAEPEGETIWHLFPHPR

NKLPIKVTPRT*

>CYP763A1 Physcomitrella patens (moss)

MAELGVSEMERMNTFGIGADAQRGLGAGMTLPLLFLATVVWWIWQRHKANLESGLPGTFG

LPFIGETLTYVAKMKSPLGNFVDEKTKRYNGAQAFKSSLFFQPTVIATEVETVKMIVAKE

GRSFVSNYPSSFALLLGRFNGLNMNGENWKRLRKFVISHIMRVDLLKERMADIEDLVVRT

LDSWADDEGRTIYVEDETKTIAFNITALIVLNLKPGKVSQTMQRDYYPLIEGMFSLPINL

PWTIYGKATQARVRILKTLEEFLQSRTVKDDVFDNYVQLLQEELPPGSPPALKHEMGLDL

LTSLLFAGHDTTAATMVFSVKYIGENPKVLAELRREHEELLKRKQPGERISWDDCKTLSF

SNSIITETLRMCNISTTVFRKSLEDVHVGDYVIPKGWLVLPYFRAVHFNPSIYPDPYTFN

PFRYQDAAGSKLPFFGFGGGARLCPGMDLARAELCLFLHHLVMKFESWELLGNDVVSYFP

FPRLSARLPIRVKRRTPPQQPST*

>CYP763B1 Physcomitrella patens (moss)

MDGRLFLQGLETVAFVCVSVLLISQLWPKNEERAKINTRLPRGSYGLPLVGETLKYMASM

MTSAPAFMAEHRQKYGEMFKSKLMGAFCIITTKADTIKWVLNHEGKQFVTGYPKSFRKVL

GEYAALSLHGDQWKSTRRFLVNSLRVELLRERIPTIEQAVLENLNPWAAKESVSIREETK

TLAFNVVAQYLLGSR696LKSGPVNDSLRNDFYTLTEGLFALPINLPGTQYRKGLEARAR

IIETLERDVVSHARPVGD876EDQYADYMDYMRKENLPGTTEELLLEKTRCHVLGMLFAG

HETAASAMLFAVKYIMDNPRVLNELRAEHENIRISKFEGGSLTWDDYKNMRFTQSVITET

LRLANPVALLWREATEDVQLNGYVIPKGWKTVCAIREAHHDPALFDRPSEFNPWRHEQEV

MNPAKKLPLLGFGGGPRYCPGAELARAEICIFLHHLVTKFDLKSCGEETVSFFPVPKFSN

GLQVQVQERDLSTRISHKIRVH*

>CYP765A1 Physcomitrella patens (moss)

MPLAVAILYAANKLALAPALLHTMTIITILTWILGGALTLGLGFIVKEWLWNPLMLIELC

KRQGIKGFPFVPFVGQMPAIDEVLSGRNRRVQKQDNDEVEDEDRLTAVTNCYRNHGSTFY

FTVGRTVRLSIADPPLIKDILIANSESYSKPLHIRKLGVLGDGIFASSGSTWSPQRSLFT

GAFHTKEVKSKIPTMIDCAHSAVEKWSRELNDGYSELDMYQKFAELTLDVIGKTAFGTEE

IGGASEAASVIGSFNRYLLYCRELVFGPPATFPTSLKWLRTYMGRIISARRNSHHSGAAE

TVSDRHDLLDVIIGAVDNIGHSEEGAKKALNEAPDQTISEKRKRAAEMTRLTEKRLLDNA

LTVLLAGHETTASLLTWTIYLLAEHPLWQKRARAEVEEFCPGGVVEPQVLSHLKLLGMIL

LESLRLFPPVPLIGRMCIKDNKVGPDLLIPEGLEIVIPVAVLHRDRTIWGDNADEFAPAR

FGNGISGACGNPLAFLPFGAGPRTCIGQTLALSEAKAVLAVMLPLFSWKLSTSYRHSPDV

TLTMMPEFGMPVVLEKIEK*

>CYP766A1Physcomitrella patens (moss)

MMVEYSQSWTALAVVELSVVAITAVFVPLWNVCSTFLLEPLRLRRVMGKQDVRLAPFNLV

FGNAFEIGAHAQSFPETLPLKFDDLEPTATPQFDLYFSKYGKRFLYHVGSETRLVVRDPE

MAKEVLFNRMGWYERSPLDLHIFSQVIGKGMFVVKGEEWEMQRRMLNPCFSNESLKPMVE

RMVKSAAQEMRNWEEMAAQAGGRVEHDVEHDIHIIAYNIISYTAFNEGFDKGKQIYLMIY

LMQDEIMGHLFAAGNPSFWIPGLRVLAGLLPTKHATAIAQLNGRTEKLIMELVKDRREAV

QKGERDSYGDDLLGRMLTATERTDGSSHKFILDAVINNCKNFFFAGSDSAANLTTFSLLM

LANYPEWQDRARKEVLEVFGDNDPCEMNDISRLKIVGMISQEIARIFAVSPSIARLAVKD

CELGDLLIPKGLVIEIATLAMHRDPELWGKDVAEFRPERFANGASAACTHHQAFLPFGAG

PRSCIAEKISWLEVKVVLCMILRRFRILPSPKYKHHPHFAMVNRPKYGLPLILEILPQSR

SDSIMAEI*

>CYP766B1 Physcomitrella patens (moss)

METVPVNVRNALAVVVASVIVYSVIKFLRVSVWQPLRLRRIMAKQGVSGPPFRFVRGQFV

EMWKFTESFPDALPIDDFANLTPTVTPQNALYYPKYGKIYLYWWGTITRLAVRDPKIVKE

LMVSNHESLTRLQSESQFLAEVVGKGLLTQVGEKWASERRTLGPFFHQKSLEGMVGAIME

GAATELQKWEQEVEERGGTAELDVEPDLHKISGRIISRTAFGDEFEIGEQIFKFQTLLSQ

ELLKGFRSTAYWLVPGYRNLPTKRNRSMNLYGSQVDALVRGIINARREAVQKGVTSSYGD

DLLGRMLTAATEGWSANTKEFNQLAVFNICKFFYFAGQDTVANAIGFMILMLALYPEWQD

RCRQEVTEILGDEQDWRASDISRLKVVGMVFNETLRIFPPASTLTRVAAKDLQLEGLFIP

KGMAIEFSLAAMHQDKDYWGDDVGKFNPERFVNGAASACTHPQAFSPFGLGPKFCIGNNF

AVMEAKIVLAMMLRRFQLVLSPNYKHHPTSIMVQSPKFGLPIILKALKIT*

>CYP766C1 Physcomitrella patens (moss)

MVFTQWVRFAALAIPEDVRNALGVVLLAFVASAIVRVVFSLVKTYLYDPLSIGRIMAKQG

IEGPPFHPIFGTTAELNAYVKSVPESLPLDEDHDSMRTVSPHFHMYFPKFGKRFLYWRGP

HAKLVSKDPGLAKEVLLSQYEFFQRHPQDIKMLSNFVGMGLDNLTGEKWAIERRTLNPFF

YHDPLKGMVEGMVKGAEPVLKSWEEEVARAGGTAEFNLEEDLHTISGNIIAHTAFGTDHE

KAKEIYQTQREYVNLLFQNLHSGWYWIPGFTYLPTQTNVTMARLRSTIDSSLHELITERR

KAAERGDTASYGNDLLGIMLAAASNSTDETATEFNLASVFNNAKLFFFAGQDTVATVLTF

TLLQLARYPEWQDRARQEVLEEVGETEAYDSTTLNRLKIVGMIVNETMRLFPAVISVSKV

ATKDMQINELFIPKGLTVEIPIVSYNQDPEIWGDDAHKFKPDRFEHGVSKACKHPRAFLP

FSMGPKMCIGKEFALMELKLVVAMVLRRFLSVSPHYKHHPYSSLLTRPKYGMKLIFSSRQ

ASKLEH*

>CYP767A1 Volvox carteri (colonial green algae)

MYSGRWWELPRDLSDLARRSRRHAAAHLAIGASAAKRNGQPKYDLDLIPGPWTHALPFIG

NLLQFLRPDFHRVCLRWADKYGGIVRIKFLWHDGLLVTDPPALAAICGRGEGAVDKAANI

YSPINQMCTPHAYPNLLTSLADDRWRAVRKAIALSFAFGNIRKKFPLIRDRTGELLEWLR

GVGPLESVDVDQAALRVTLDVIGLSAFGHDYGCTRLQQVPYNHLLRVLPRAFTEVMRRIA

NPFRSFAPGLVKNGKKGLTSFKDFQRHMQELLGEIKARGPPARGDADIGAQLYRVLEAAR

PAITDERILSEIGILFVEGFETTGHTISWTLFNIATTPGTQEAVAEELSSLGLLVRPKSE

GGRSAARQLELDDLKRLRYLTACVKESMRMYPVVSIMGRTTDKPTRVGPYVVPSGTPVAT

ALFAIHNTIHNWRDPMTFKPERWLGECSLGVLGSFMPFSEGPRSCVGQSLAKLEVMTVLA

MLLANFRIELSDEMGGREGVRQRESTHLTLQTRGTRGIRMHLHPRDQE*

>CYP768A1 Volvox carteri (colonial green algae)

MWDTLRFYYSTHGPLGAWTPAIVLLLNILGIALALAVTKFIGLYFAPSYDLRKIPTPPVG

DAILGHVKFLLRPDYHRVILAWTRKYGKIFRLRILTQWTVVITDPAAAAQVLAVVPGRTH

NYTLVDEGLGGPGKISMFGTRDEAHWRNVRKATAPAFSMANVPDARALPGFDLLVPRILL

LMAEANRQIVDPLWALWYRTPLAPLLSKHVSECRAAVREVRAFHTATAARLLDRPDPPSD

NTLLWACLHRLRHHITGARLTPTQLHPEVGMYTTAGFDTTASTLGWCLYAAALHPDQQQK

VADELQQACVFGNGAVVEDLVKLPYLTAFVNEAMRLYPTTAVAAERVSPDRPVAVGPFTL

PPGVVLWPLVYGIHMSDANWDEPEAFRMERWLEDPRCAFARGERGPGASGAPRRFLPFAD

GPKNCVGQNFGLVVVRAVLALLLSRYRVALHGDMGLERVAVVTKLSKLRLVMTPRD*

>CYP769A1 Volvox carteri (colonial green algae)

MSIDARLDRRLNYRCNLRGRVSRRALQDVHLSTRWTKTAPPPGVPLLGHSLTLRAWPSWT

WWWFRSGGPRGDQLLLRALLRWSEQYDGAFQLRNGWLVLHPNAVPSSATATSSAQWRLLR

RSLLHAFSDSELQLDFEGPGAVVDVNDAALRLSLDVMGLSKLGYDFQVGMAVAVESQGEV

LMLRLLGEVAAEWAVRRRRLLGRWAPWISDGAAEGQTRCRILHHFIEQLLLAHGPTGHSI

AWALGCLAARRGVQEKLVAELKKEGIFNDPLRLTYDMLSKLPYLDCVVREVLRLYPTMPC

PATVRTLKKDVALHGRTLTAASDVWVDVFSMHRSPKWWRDPHHFKPERWTASPPPLAPLC

SPEAFMPFSFGSRSCLGQKLAVAQIKAALAMLLCFLVFEPSVAPWGLGLFLRPEGGMQLL

VAPRKKNS*

>CYP770A1 Chlamydomonas reinhardtii (green algae)

LLVSEGQQWRLMHALATPAFKAELLERGAFAAALRGVMEEWHRRAVALLPLWRLQAAGVA

LTLVGMGHENVSATAAWALLLLAAHPEQQQALYRELRQGCGFPTSRFIQSHPSRTAALLR

LPYLDAVLRETLRLYPPVPMLSRQLMQDTTIGGVMLPKDVELVVSPYVLHRLPRLWGPHA

ACFQPERFMPPPPRPPPAAGGGCTEPAAAGPYLPFGAGPRACPGASFGSAEVKLLVAHVV

MRYSLELLQPPPPSPRQLFVSLRPGPGVRVCFVPRHQQQVE*

>CYP771A1 Chlamydomonas reinhardtii (green algae)

MRAGYVRAKAVSCLWPKCRQLPTRVRFIRHVRWKPKRSPPPLLRPAHVRYLGKRKLLLRE

PDDVAAVLARPGEDAFRKHPRQQRVSAFLGAGLATQPDRQRHAAQRDALAPAFRPDAVRQ

LDAVMAAAAERLAEALMAAAEAEAEEAEAVAAASGSSSGAAGAAAGAGAGAAAGELQVEM

QDLLKRHSLDLLGLAALRSDMGALRRSPVMAAAAAAAAAAGGGYAAVGADVDVVTLMTEI

EAASLWLLMALPVPNELLPGYGTYEANVRRLDELLVTMLLGGTDTSALTVAFAAWHLAAE

PQLQAELRREVLGVLGGRALGELRAEDVKAMPLLAAVVNETLRLHPPLAEITRVATQPNA

FLPFGVGSRSCIGRHFGLLSTQLTLAALVARFEVLPPAPPAPTALDWSQSIVITSRSGVW

LRLRPIRQ*

>CYP772A1 Chlamydomonas reinhardtii (green algae)

MTMVQDSMIQALDALPVPAVAASVVAVIITTVLLAVFRSRPGDAPSVPGLPLLGSAMALG

RHGVAFINKCRQQFGNSFSLSLAGVKMTFLFDPQHIDYFFGAPDSKITFR(PAVEQFTQR

VFGLTSRLFFPLHFKMLTELRHLLVPASIAAHMQALGGRVLALLPLYVHHPQVDLYSLCR

GLVFHCAGGEGGhqRPPEGVHRLARDFFAFEDGFELAASPVPHAFQPEFTAARQRLLALL

AAADARGLFAGTLAGQLLERTAGLPPALRPNLLLAVLWASQANTVPATFWATGFLLLPEN

AHHRAAVLAELQAELKGAVSAAGSPGGSAAYSNEELVAAAARVASSRRSAVSRCVAEALR

LRVQSIDVRIAADHLELPLAGVKGGGGDVLRLPRGRLLAICPFVSHHDTQLYGGAAAAAA

AAAAGCPAVTGAAAAGDVSSPWAFNPDRPELKLGDGTAVVSSVAGLAFGGGPYRCPGRFF

AEQELGLLVQLLLWTYDIQLSYTPQLRQVAGGSWLYGVLSGLVGARALAWGCGWFDGVDG

PLEDFRHSGDPGGLLPPCDLKRLVGVKVPRRPLWVQLGVPHWQARRLGLVGVAPAATSRR

WADIGLG*

>CYP773A1v1 Selaginella mollendorffii

MDLIWNVVTAGVSLIATICVWQLARDFLWRPRRLLQAFKQQGVLGPVPRLFLGNLDQVRE

LMAVEVVKSSTGEIRDDNHGGVVAKVLPYYAAWSRSYGETFLVWWGSQPRLMISDPELMK

EVLCDKSGSLDRDPGQHAARDLFGDGIALLTMNERWSQKRKMVSLAFHNEKLKLMIDAMV

ACVEENLKQWKTTEGPVDVASKLRDITQDVLCRTAFGTSYAAGKEVFEMQIEQQYIHLEW

QGQVHLPGFRFLPTSANRRRWTLKQQIDSKLRKIVVNRLKESSVSGSYGKDLLGLMLAAK

DGVLDFNNGKKLDIQVTMQDVIDECKTFFFTGQETSAALLAWTMLLLALNPDWQTRLRQE

VCQVCGQVSAPNTLEMLGNLKSMTMVINEALRMYPPVPLLNRYTHNKVKLKELVIPKGTL

LLVPLIVINYNEKFWGVDAKSFNPDRFVSQQQRPFLPFSVGPRTCVGQSFAMIETKIILA

MILRKFKFELSETYVHSPFQVLTLQPKFGMPMNLLANQ*

>CYP774A1v1 Selaginella mollendorffii

MEDFLWKILCSCGVFLVVWLLVPAARSLWHAWRFRCFYAKQGIPGPPFRLIVGNIPEIRK

LFNSVPKFETSFHAATKFRVIPDLATFQQTYGRISVHELGSTTRILVADTELVKQVLMSR

SSSYIKADLSRQILRAVVGRGVVVTDGDFWRQQRKILNPAFKLAYLKGLMRHMSGAGEDL

ARKWSSRETTRIDAHREMAALTLDVITRASFGATIGGTNTGYAAFECLDRLLSTGLLYMN

SYKRLIPGYSFLPTRENLHLRRSEQYVNTLLRDIIRNRWAEKTRNPDENGKPVYDLLDMM

LEAVENKSPTMTMDQLLDECKTIFFAGHSTTALTLTWSLIMLSVHQEWQQRARDEIFAAH

KRCGGRDLSAEDLSSLEVVGWIIHEVLRLFPPVSTVTRQCHQAHEIGEFSILPGTLVLCP

LALLLQSKEDWGDDVSEFNPERFINKKTKDISEFMAFGAGPRMCLGMNFALIEARLLLSL

LLAKFSFTLAEDYVHAPGSPVSMKPVYGAPLLVKKL*

>CYP775A1v1 Selaginella mollendorffii

MQGLEGPRPKFLVGNMDEITRMKETAFHQPMEIGDHNLLQRICPYYLEWSKLYGRTFVFW

WGTEPRITVTRPDMIKEILYSKAAHFGKSALQRKGGAVLLGNGLIMANGSDWAHRRAIVC

RAFKMDKIKEMVPSMLESTKNLIRRWDAHLELNGGAPCEVDAYRDLAVVTADIIATTAFG

SSYSDGIKLFHTLTSIQKLFVQSNKYLWLPGSRLLPTRTNRKIRKLQREMQALLQDLIKA

RLSSPSLGTDLLALMLSAVEEDPGNKVQSSKFKFTIQQLIEECQTFFFVGHETTLMLVTW

AMMLLCLHPEWQDLARKEARQVLQESNRVVNADTLAKLKTVGMIINETLRLYPPAPNLVR

AALQDTCVGDLYVPKGTTFWIPILALHQDKHLWGEDAHEFRPQRFSQGVSRACKTYDFLP

FSSGPRICVGQSFAIMEAKLILAMILQHYHLGLSPRYKHSPVSSVTLKPGLGMQLMIKRC

D*

>CYP775B1v1 Selaginella mollendorffii

MLLLAAPMLVFLASLLVILVWSASVAFRYVHLRKSLLEQGLDGPPPKFLIGNMHQVSEMR

ELATSKDMRVGDHDLLPRICPHFTYWSAIYGKRFLFWWGMEPRITVVEPEMIKEILSTKA

EHFGKSLLLKKGGVLLLGNGMVYANGESWAHRRRIVGPAFHAEMLKKMVPEMVASTSQML

GQWSQIIDNKSSRRSGDGSSAEIDINYYLSMATADVIARTAFSSTSSHEKGKRVFQLLTC

LQKVFAQSNRFLWLPCNRMLPTAANRRASRIKRDMERALRELVLERRAGRQKHGYGSDFL

GLMLSESERDKSEAAAAAQQFDTPELVEECKTIFFTGHETTSALLTWTLMLLALNPEWQQ

RGRAEVMEHLPSKSSVPDADVLPKLKILGMILNEVLRLYPPAPALVRESLVDLSIQDVKY

PRGTTFWIPIVALHHSKDVWGDDALHFNPARFADGVAAACKLQHQKLWSFMPFSLGPRAC

LGQSFAMMEAKVVLAMILQRFEFKISPNYRHAPVTAITLKPRYGMQLMLAHYNIEDGEKS

PG*

>CYP776A1v1 Selaginella mollendorffii

MAMEWWSAIIAALALAKSIALNLVIARVVGFVFSLCRLHVFVRRRLAKQGILGPKPSWLA

GNAVEMKRLVASATSADMKSTSNDISARLLPFHHKHAQTYGKRFLAWSVGWEPFVSISEP

ELIHEILNSTDFEKSGIQNRFMMPLFGRGLVMATGKAWDHQRRLLNPAFYVERIKGFLPT

INFCASGLVQEWKGLIRSSSSNVVEVDVHSVLTSVTADIIARTSFGHEFTHREEYVRLER

ELEVCVLNQPAFCLIPGYRYLPTKQNRKLWEITRKIRSYLYELIDARLATGKDHFGDDIL

GLLLAATFSSSPSSTKKVPPMSKDVLIDDCKTLFFAGHESSADLVTWSMMLLALNPEWQA

RARSEVLQVLDGCEVLTSEMLPKLKLIGNILSETLRLYPAAVAIRRKAVKDVVFTKGKLV

IPKGVCAEVPILRVHHDPELWGDDVLEFNPDRFSKSEAVAAGSYLPFGWGPRICIGRNFA

LAEAKVVLSTLLDNFEWEISPSYRHSPRAGVTLYPQHGMQLLLRQLPQN*

>CYP777A1v1 Selaginella mollendorffii

MDRVVWVGLIAAACWIAVLKLVELVVKSWWRRRRICQVMEGQGIRGPPCNLLDGNYSEIK

RMQAEAAAVDMPALTHDIVARVFPFQHKCTQLYGKHFLHWWGQDPIIHITEPELIVEVLS

LKFGHWQKSSQLRRAMEFLFGKGLLVAVGEDWVRQRHAVNSALSAEKIKCFVEVVICCVK

PMVRKWEQRVEEGGEAEVEVKQDMLDMATEIILRSSFGDECYDEARRYPELVYRLLGLTS

KSSPFNSLIPSFVPTKKNQLLKEIEQCFYRVVATHTQQRNTILSSLLGCAARSSLSVQHV

IDECKNIVFAGHETTAHMLTWTMMLLGLHPEWQQRAFEEVAEVCKGRDPTSDTLSKLRVM

NMIVNESLRLYPPGAQTAREALKDMKLGDRITIPAGVSVAINIVEVHRSVEMWGDDALEF

KPQRFAEGVSRACKQPVGGYLPFLLGPRVCVGQGLALMEAKLALVLILQRLSWRLSPNYR

HAPIVALTLQPQHGMQLVISPRRTHDR*

>CYP778A1v1 Selaginella mollendorffii

MDVISVLLVALVLALSVLWMVCSFLMRYYWVPSALRAVMEKQGIRGSPNPPNPIFDVFDG

KPDMKEISHDILPHVLPWAAQNMKFYGNVHLNWWLREPRIVISEPKMIWDLFMKKHKDFV

KSHFIKLLSDDIFHKGLFLANGEAWARQRQIVAPRYYIDEVKAMVRAVNNATSQVMAKWE

AFVKDSGDVERELDVQVEFMCLNVDVVARTTLGLEDNDFQNILKYNITLLKLQNDQETWS

WLPFARLIPFGINVERWKVRKQLNDLVRKQVRERRKKMTEGNNIDFIGKLLDNPDVREDV

IVAELKTLYATGFISLAPLLSFTMLMLALYPSWQEKARQEVDQVLDGEVVSPKDVTKLIT

IEMILQETLRLYPTMPLIARVCIKDSMLGDVFIPKGLGVSVNVVALHHDRDLWGDDVNEF

NPSRFKNGTATAAKHPMAFMPFAYGVRTCIGRAFSEVQCKVIIAIILQRFEVKLSPNYRH

HPVITGPLIPKNGMPVILKPRQNF*

>CYP778B1v1 Selaginella mollendorffii

MWLVLLAVLAGLLLILWFQCCSFLVYHLWRPKVLEKVMSAQGVTGPPQKNLLTQIWELPD

MEQVSHDIVPHAIPFHHARLQKCGPLHINWWRVEPRVELADIDLMRKALLKGPEFFGKSP

VLGMIADDIFGGGVFDASGRDWVEQRRVVVPVFHADKIKGMVRTMYENTQSFLENWVTLI

RNGGTGEKALDVFPEFVELTAPIIGQAAFGASSSTSIAIVKLLRLLFALQWQQVRYASLP

FLPTNRERWRIKREIHRLLRVEIDSRRALTRENCAASHGSDLLGTMLDSNWDDELIITES

KTFYTTGHMSLTSLYSWVMLLLAVNPEWQEKARVEVLELVAREGPLDNAQALDKLKLVEM

IIMETMRLYPAFPIIPRIALEDCYVDHLFIPKGLAVSVHNTVIQHSAEMWGEDANEFNPG

RFANGSLAASKHPMAFMPFSFGARACVGRAYSQVQAKVVVASLLQRFRWSLSPDYRHNPV

AAGLLLPKNGVPIVLKLLDSKTIVTNGMKATGERS*

>CYP779A1v1 Selaginella mollendorffii

MRKLEAALRGQGLKGPPPIFLAGNVVEILFRREAARNKGMDGISHDIVAHVSPDVAAWSK

LYGKPYLIRWLSEPRVVVFDPDSIREILSKQFDKFEKSEQQLEFVLDFIGAGLVGLNGNK

WSHHRSVLSPAFHTQRLKAMLSSMTNCTEKLVEKWSRRVGHAKGLETEVEVQQDLKRLAA

DVISHTSFGSNYEKGERVFQGLTLLGVLLVRCFHNSWLPFFRYLPTKLNFQIWKLRREID

GTLLSLIRERRIAAAKLGERSSHPYGSDLLGLILEEGETGGKSVKFPEQAIVDECKTFYL

AGHETSSSLLAWALLLLATHPDWQEKARAEVQQHFPNGVDDGETLSKLKVVGMIILETLR

LYPAAGEMNRASSHDTVLSNGIKLPRGTGITIPILSLQHDPELWGPDANEFRPERFANGT

TKACKHPNAFLGFSFGPRVCIGQGLAVMEAKVVLAMLLQNFSFRLSPNYRHNPTVQIVIQ

SFTGIQLLVQKI*

>CYP779B1v1 Selaginella mollendorffii

MVFVIPVLATGLVGVIVYALLWKFWIAPSRKEAVLRKQGIKGPSLGPPQLFKGGNKDEVL

KRRFSKRKFTLEFDGAHDILSHVLPDIHSFSKKYEMPYMYWWGNELRMTVTDPEVVRWVL

SKNPQSFGKSASIQATLIKLLGYGLVASNGEHWAQHRRVVGPAFHLEKLKNIMAGTMVDC

TSKVLSRWDNDGEFEIDVEKEFSFLAADVISHTAFGSSFEKGRRIFHLLNLQAELLTKIA

FSPMQWMPFGRLHPLRENLQLWEVQKELDAILLGLVKDRRKSASYGRDLLGLMLEQSQDN

PAFKDDKLVGECKTFYIAGQETTATLLTWAMYLLSQHREWQDRARKEVLEVCKEDEINAE

ALNKLKLVGMILNETLRLYPPIPIIQRGTFNDTTMGDKISIPKGIVLVIPILAMHHDKEQ

WGGDAHEFNPERFARGASKACKHPNAFMPFSFGPRVCIGQTFALIEAKIALAMILRRFSA

SLSPNYQHCPVSGVTLKPLHGMQLTFIRR*

>CYP779C1v1 Selaginella mollendorffii

MAVWLWTIAALCIALVWKGAAKLLLKPWILEAKLRQQGIRGPPRSILSGNVYEIFQMRAR

TEAECIQGPITHDIVEYVQPHLLHWAKLYGLPLLWWWGTEPGVVLTDLDMIKEVLYNKSG

AFWSPEWQRKFQVDILGRGLAVVNGDEWAFRRRILAPAFHAEKIKASNCEMLEKWNALTE

GKDEPIELEVCKELTTLTSDIISRAAFGSSYKKGHKVFELLDQVGGLTCFPLAKRFTHCH

SMLPICKLNREIKTANSKLRSTLEEIVQARRDQKLAGEIDNYGSDLLGIMLDEVDAGHHD

DKTGLSFTTDSLMEECKTFYIAGQETSAKWLAWTMMLLAANPSWQEQAREEVRQVCQSQA

PDAESLSKLKIVGMVLNESLRLYPPAVFNVRSCYKDAKLGHLSFPEGSGVIIPILYLLHD

KDIWGDDANEFNPQRFADGISSASKSRHSCAFLPFSQGQRVCLGQSFAQIEAKVAMAMIL

QRFSFRLSPTYRHSPVHRLALQPQHGLPLLLGRP*

>CYP779D1v1 Selaginella mollendorffii

MEGIGKAVWTGALLVLLALVAKLWRSIVTRYWLEPRSLDTRIRSQGIQGPPRTFLAGNML

QVMKMRDTPKERDMAGLNHDIVEHVLLDYHQWSKEYGKMYFYWWATEPRIMVTEPELIRE

VLAKKVTQFEKSDMMVSAVASIIGRGLIAVNGNEWSHHRRVVAPAFYLEKLKKMVPRIGL

CALEMLDRWEEALREQPEIEMSSEFSKLTADIISHTAFGSSYLKGQKVFETLRAIPEELS

KVDRYNYVPGKSMNPFSELNRAIRNGQKKVNNLLLEIVHARQQLKDSGASSNYGSDLLGL

MLDEVDSSRSFSGSGIKPALAFTSESLIEECKTFYVAGHETTAKLITWAMMLLATNPTWQ

ERARAEVLEVCKSGVPDSEAASKLKIVGMVLNETLRLYPPAVFLVRTAMEDTKLGNLIVP

EGTGVLVPILSILHDKEVWGEDANEFNPQRFADGVANASKHPFAFLPFSHGPRVCLGQGF

ALMEAKVALTMILHRFSFEISPSYQHSPVLRLTLTPKHGMPLLLSRRGV*

>CYP779E1v1 Selaginella mollendorffii

MEGSWILSTCIVLFTAALWRFVTVYWWRPRVIAAQLKKEGIQGPPPRFMVGQIAEIQNMR

SAIKDHDMGSFSHDIFHRVHPSLLKWRKQYGKRFVFWWGTEPRISVSEPEIVREVLSKKF

SQFDKSEAGLRLANLFLGRGLVSVTGEEWSHHRRLVAPAFFHERIKQMTGTITGCASRML

DQWEATRQQNPEIEISGEVRKLTGDVISHTAFGTSYLEGQRVFEILSKKFPELMPKLVSF

SWIPGFRFLPLPINLRLWKLHQKLDSLITGIIDERRNSVKSGGSNTYGNDLLGLMLKECD

SSTNFTSRDLIEECKTFYIAGHATTATLLTWTLMLLGGYPEWQERARAEVHEVCGNEIPD

GESVSRLKLVGMILYETLRLYPPVVEMTRECVEESWLQDLHVPRGVSVSFPIAGLHQDKE

LWGEDAGQFNPDRFKDGISSACKHPNAFMPFSFGPRVCVGQSFAMIEAKVILAMILQRFS

FRLSPNYRHNPAMKHGLKPTHGVPLVLSKM*

>CYP780A10 Selaginella mollendorffii

MELFLQSWNALYFLVFLLSLWLISQKFYKSSTIKLPPGSHGLPLVGESLSLFWGSPLDFL

STRRKRFGGVFWSNLLGSPTIVATTVGSAKFFLSCADCGPSGLFARLIGPKSASEVIGSE

HALYRRIILGMMVPETLKCHVQMIDILAQETLESWGSKKTVSVMEETVKFSYCTVIGLVC

QKLLPSTPEMIDLMRDVQTIENGVLQFPINLPFSPYRKALQQARARLHRFLDGLINERRA

ELAANGETDKDALDEFITHKDDKVGFLSNQQVEDNLMTMLFGGHHTTALALMWLIKHLNE

NPQAFKEVEEEQRRILLGKRSTKYSLTWEDTRQMPATLRVAVHESLRLSNVVGVVTRKIT

KDISYKGYTLPKDWMIHVYMPPIHLDDSIYPNAAKFNPSRFEVPAKTGTFIPFGYGDRIC

PGRALSQLEQMIFIHRLITKYRWEPVNPNSTTSYWPMPSVKDGYLVHAMSI*

>CYP780B1v1 Selaginella mollendorffii

MALWSLYLLLVIPAVAAFLISSKRKSGSVQTPPGNRGWPILGETIQLLRGTAEDFVFQRR

KRFGDIFSAHLFGRQSIVISTPEAVKFFLTNPGARNCCSPSNSGFLIVGKESVGHVEGAT

HARYHRAILSSMSGDPLNNHVQRFDKIAMDLLTSWQRKGCVTVLEETLQLTFDVVTAFIC

DDPRIFQTKTGDFMHDVTVASRGLFKLPINLPFTDYHRALQARKRLHYHLDRLINERRIS

KITHDDLLHKLMNDKDLNSTNQQIEDNIVGLLFAGQHTTPLTLVWMMKRLQENPEILKEV

VEEHQKILREREQPHLTWEDTRRMPVTMRVLQETLRLASGGMLVREMKHAVEYNGYVFPK

GWTLHIFHTAIHLNEDYFADPYKFDPSRFLVPQKPGTLIGFGCGLRTCPGAELAKLEILV

FFHRLVTQYSWKPKAPNGAIRNWPLRIPEDGYVVEINRK*

>CYP781A1 Selaginella mollendorffii

MFWIGWLCVAAAGALLASLGNVYSHWQKLPPGPWGWPIVGCLFCVSRRNLHRSFAELATK

YGPIVYLNMGSRATVVISSPEVARAVFREHDVQFASRPRYSTPFKHISQNFKDLVFAPYG

GRWKNLRKICSTELFTASKVNMFGGIRKAELHDFCNSIAMRAAAGEEVNLSVCFQELLTN

LMSSVLFGKKFYTSDLPPVAEAAAYRATWGMLTQESGKIYLGDYIPALHWLDRLRGKDQR

IRKTIIPALQGLLNSVIEERRKQLRRDKPRDFVDVMVALNDQKSLSNDEIVAIIQDMLLA

GTGTTRSTLEWGFSELVRHPEVQRRAQEELDRVVGRERYVQESDLSGLPYIQALVKEIMR

LHPAAPLGLPHFNSCPVSLAGYTIPANSTLHVNIWTICRDSSSWERAHEFRPERFLGSCH

NLLGQHFELIPFSSGRRRCAGINLALLHVSLTLAYLLHRFEWRPPPGVDVSEIDMSETTG

LACFRTVPLRVSVRPRLELP*

>CYP781B1v1 Selaginella mollendorffii

MARKYGPVMSFRLGVRPHIIISSPEMARQVLKEHDVEFASRPLFSTISRLVSHNFQDLIF

APHGERWKMLRRVCGTELFTASKVSHFASTRKRELGAFGAIVEASAKDGHEFDLSSMLHE

YFTNLMTCVLFGRKFYGTDTPLTPEAEAYKASWAIQAKESRRLFAGDYIPAMRWLDTLRG

TQNRLKNEVLPARSRFLEAVIEEHAKDFDPENPRDFVDVMLTLGGEDKLSNDQIIALLQD

LLLAGTGTSKGTIEWAISELIVNPRVQEKAHEELDRVVGRDRPLEESHLNDLPYIQAIVK

EVFRKRPIAPLGVPHYNDREVTLAGYTIPAHTTVLVNIWAIHHDPSVWSDPELFLPERFL

GSDHSVLGNDFDLLPFSSGRRRCVGIPLAMPHVTLTLAYLLHRWSWRSPLGKPIEMAELA

GAGIAGVASPRIVCASHR*

>CYP782A1v1 Selaginella mollendorffii

MEGHLAFAAIILGFLLFVLRARKSRDRAALPPGPFQWPLIGCLPSFPFHHRHRGFLELSK

KFGPIVTMPIGSSKIFLVHGKDLAMEVLRFKDAQFSSRPLSMTGKYIGFEHSDPNLCPLN

ENWRVVRKAFSNELMAPSRLSSQAWLRREEVLKIVDSLLGITGHGRDWASVDVRKIAEGV

VGRIIMRMLFGDHYLGKNIDNPDAGRITRELEREFEKYLAEGNFLWGELNLADYFPALGI

FDLQGLEGRFKRLMSKLEPLFTMIIQEHRKNTVMIQDEKGKDVIDVLLQNQLSDKQIMGI

LSDALLPGIGTTSAAVEWAMAELAANPHTLSRAQQELDSVVGRSRLMDESAIPSLPYLQA

IAKEVLRLHPSAPLDDPHLNEEESSLGGYAIPAKSTIFVNLWALGRDDRLWSDASRFDPD

RFLGTEIGVHGSHFELLPFSSGRRRCPAHALAMIKLQHIVGALVHGFDWSSAGAVDLIEG

NGIIASPRTPLRLRARRRLDDEAY*

>CYP783A1v1 Selaginella mollendorffii

MEGAWFLALALLILLWWSRNLRARLKLPPGPFPWPIVGSLFIVKEPLPIFFAELGRKYGP

VVYFKLGMVPTVAINSAAAAREVLKSRDLEFASRPDLGNLRQISFDYNDLGVAPYGETWK

LMRRVSATHLFTPSKLNTTASVRHREVKAMIKNILDEGPEVVDLTAATNAAVVQGVVNLL

VGTDDKSLGIDARSLNGIFEKAGEELLNVNLGDLFPFLRRFDVQGLERRFKYVVMPPIKS

LMEKIIAHHKSSSREVEDFVDVLVNLNGEDGLTHIQTIGLLSDFFIAGINTSQTSIDFTL

AELVRHPAILSRAQKEIDQVVGSSRLVQESDLPRLPYLHAVIKESLRLHPPLPLLLPHHN

PAASKIGEYDIPAKSTIFVNAWAIGRDPSTWDRPLEFVPERFLERDVKLTGDDFSLLPFG

AGRRTCAGYLMAMRMLPLSVATVIQAFDLATLEGREVDMGESTGGATRRNKNLMVSATPR

LAKELYA*

>CYP784A1 Selaginella mollendorffii

MELLDLVIASCILALSCTWLMSFRKPQGRLPPGPLSIPLFGSIFSLKQPLHEHFIHLSKK

YGPLIYLKVGMCDLLVANNSAMAKEVLQTHDVEFAYRPDTRSFRLFSLGYKDLLFAPYGD

SWKKLRKVSTTHMFTSSKLNISARQRETELLSIIRSIKTSFDSGNSVELRDLVAEYNTNV

ICLMLFGQKLEAAKTVVGLVEKTASLSLLINIGELFPLMDWLDLHGIYKIMKKEILPDIK

KVLGEIIEQRNGTRKEGQEARDILDVLLTLKDDDGVSEASVMALLMVMFTAGLESSQNVA

EFSIAESLNHPHIIQRAQQELDAVVGRKRLVREEDLVKLPYVQGIVKETLRMHPPGPLGI

PHANPKPVSIAGYTVPANCKVLVNMWAIGRDPACWDRAEEFLPERFINSDYDVAGNHFHF

IPFSAGRRICVGYPLAMRSIPLVVATLLHSFEWKRQDGNSLETAKGLLSIKLASKINLSG

HPRLDESAYY*

>CYP784B1v1 Selaginella mollendorffii

MGITIAILVCLLATAAIIKSLRARRSSRLPPGPISFPVVGSLLSLRQPLHRHFARLADRY

GPIVFLKIGMVPYVIANTARAAEFFLKIHDAEFANRPQSEEFFRIFSFGWSDLAFRSPGP

EWKLMRKICATNLFSNAMLATSAPYRRSQLQSAMDAILDRSRGGEPVNLRTLFARYTSGS

LCLTLFSEECPEVVETINNMAGQAINLNIGEIIPSLDWMDLHGVYAKMRGEIMPRIKALL

DDQVREHQERKKAAGDGFVCRDFVDVLISLDESDKLSDQEIIGLLCDMVGAGFKTSMESI

EWCMAEVISKPEIMRKAQEELDQIIGRERAVEEHDLQNLPFIQAILKEALRLHPAVPLGM

PHYNLRPVELGDGHGTIIPAKCKLLVNLWAANRDPAHWTSPHEFQPERFLGTNISPGGQH

FQIIPFSAGRRMCAGYGLAMRSLFFLLASLLHGFIWSEISDNPIALEESIGTISCPPAKD

LIVAASPRIEERILAQY*

>CYP785A1v1 Selaginella mollendorffii

MDLLSLPSLSALVLLAAALWCSSTRRRNPPGNLPPGPLNLPVIGCLHKLGSLPHISLHKL

SKRYGDVMHLKLGSVSTVIISSERAAREIFKRHGLEFASRAPLICGKYFGNDYSGLVFSQ

YTPEVKLYRKLINTHLLSPTKLKSYDGIRREEQRRLARSLSDDRGNPVLLRQKLHIMNMN

VITYMLFGKHFCGHYKNTANVDEFVQTVVEMVRLAGIFNVSDYIPGIRWLDVQGLEKKYK

QLMNQVNWHLLGILRDRLVDPPVFTSEEPMSFIDVLISMGEKLSDTTKITLLLDVLMGAV

DTSALSLEWAMAELLRHPAEFSRVQSQIDTIVGKKKLVDESDIAKLPYVEAIAKETMRLR

SVVPLGLPKIVQGGPIELDGYTLPNGTVIYISSYSIGLDERFWKDPLEFRPQRFIDLPDI

DVFGQNFNLLPFGTGRRVCPGAKLGFDAVQMGIATLVQGFDWKLDGDLDDPAKLNMDQTF

GLVCQKSQPLVAIPIPRLDSHVY*

>CYP786A1v1 Selaginella mollendorffii

MAAQASSTAALSLLWSLLVAIWKKTLGKLFFRDDRHQQDHMPPGPRPLPVIGNLHQLLGR

PPHQALLDLSKRHGPLMFLRLGCVPTFVASSAEAAREFLHTHDLVFASRPRYAVARELTY

NFADIMWAPYGDHWRHLRKVCSLELFSGKRVDSFERLRKEEISSALATVEEAARASSVVD

LRAVLSDITLYSILRMATSQEFGGKKKQLSRFERRVKETIEHAVEMIGALNVGDYLPSLR

WMDLQGYGRRARKLHALQDAFFQSLIDRKRQYQGRGGAGAGGVDDLLDVLLASQEKNALT

DDTIKAVIQDVIGAGSDTAWVTCEWAMAELLRHPTAMRRAQREIDAVVGRDRVVEESDLP

GLNFLHAIVKETLRLHPPSPVILYESTMPCVSSAGYRIAQGARLLVNVYAISRDANSWER

ALDFWPERFEEGAKKGVDVRGQNFELIPFGSGRRICPGMGMGLRMVQCVLARLLQGFDWE

KVGEIDMREKFGLAMPKLVPLQAIPCPRRS*

>CYP786B1v1 Selaginella mollendorffii

MNLFAAAAFLVIGLVYWFVNRQRPSTPPGPWKLPVVGNLHQLLGKQPHRVITELSKKYGH

LMSLRLGSVQAVVASSSQTAKIFLQTHDVIFSSRPEVANAKLLTYGFSDIMWAPYSQQWR

ELRKLSVLELFTAKRLESFQGIRRDETLNMIHRLLKLAREKKVVNFRDAATELSWSIIGT

MVSNRQEFVNLEEGLKVKSSLDRALQLAGAFNLADYIPFFRAFDVQGFRQQSQILHEQLD

FFFQGLVDSHRRQERPPNASEDFIDVLLSIQKQNGVEYVSDDTIKATIQDIFAAGTDTSS

MTLEWALTELVRHPRSLQKAQDEISFIVGNDRMVSEADIPKLQFLQAVVKETLRLHPPGP

LLQHQSMEDCKVGPYSFPAGTRVIINVYGISRDPSLWEQPLEFDPWRFLDKPTASIDMKG

QHFEFIPFGSGRRICPGLAMGVRTVELALAQSLHCFHWHSPDDRVPDIEEVCGMTLPKKN

PLLLAPSPRLADAVYGEIQRM*

>CYP787A1v1 Selaginella mollendorffii

MPTSCCKMKLPGTFVFVIGSLLFTLLVVACQALDHLSTSPGTQMNWFLLTFACILTAVIS

VSWWLMLKSRLRLPPGPMALPIVGHLHLLLKLPHQSFHKLSHKFGPIMTIKLGNKTAIVI

SSKKAAKEILTSYDRVFASRPVLISPQSLCYNSKNISCCKYGPYWREMRKICTTELFSSK

RLSSFQNTRLEETQNLLQRVAEQLKVPLNMKIELSTLTLNVITRMAIGKKFRHGECSEDA

EPLNVILEAVRLMGAVNLGDYIPFLKRLDPGGYIPRLKTTSKKIDCILQRLVDDHREEKV

KSGDLVDVLQSVGIEDSAIKAVILDILAGGTDTTAVTTEWALSELIRNPDCLRKVQQEIH

VIVGDSRLVNENDLHHLHYLKAVVKETFRLHPAAPMMAPHESIEACTLKGYTIPAKTWLL

INAWSMGRDPAQWDSPEEFMPERFINSSIDVKGCDFELIPFGAGRRMCVGMSLALCMVEL

TLARLVQAFHWALPDGSTMNMEERQGVIVARKHPLIAVANRRLPPEVYINTL*

>CYP787B1v1 Selaginella mollendorffii

MEALSIILVGAATLVLCSLFASRFLYPLPPGPWGTPLFGHLYSLGELPHQTLSKLSKKYG

PIMTVRLGMVPALVIDSPQWAREFLTTHDIAFASRPQNTNSKYLFFNGSDVGFSPYGEHW

RNLKKLITMELFTAKKMEVFKALRANGILRVLKSIAAEEGNVVsirnllsmlnmnnisQM

AFSKQVIDDPIFQRFLAVLEESLDLMAVFVLGDFIPFLKWFDPYGYVAKMKANRKEISGI

YQMIIDEHKLKRKKNCTPTDLVDILLSQGVDETTIKGTIMGMFVAGTDTSSLTSEWALTS

LINNPGCMRRAQEELDrvvgrerrvqeeDLSSLVYLKAIVKETFRlhppaplllpRESTQ

ECTVKGGYKIPKGTRLIINTWSIGRDPAETPSPEEFKPERFLGKSIDIKGQDFELIPFGA

GRRICAGLPLGQTMVELTLASLLQAFEWKTDKTLDMEESEGLTTRMKVPLAAHVTRRTSL

KF*

>CYP787C1v1 Selaginella mollendorffii

MAALVVTLLIVLLPLLLWWLRIYQSKKNVAPGPLAIPLLGHLHLLGRHPHKALSILSKKF

GSVMSINLGSVPTLVISSPDAAKTILSTQDIFFASRPRTAAAKFIFFNARDMVWCEYGSY

WRTMKKVSTLELFTAKRVEESKKLRMEEISRLVTSIAREGDNGRVAIDMNAKLSMTNMNL

VSFMAFSQRFEESSFVELLQEAIDLVTSFVPSDYFPYLSWMDDYLGTVPKMKAVQGKLDK

IFQAIIDEHRRVNGEKQRAPDLVDVLLSLDEVDDNDRKGLIMDMFGAGIDTSSITTEWAL

SELIRNPACMLKAQREIDQAVGFDRAVNEDDLLNLGYVRAIAKETFRLHPPVPLLIPHES

TQESLVNGLRVPARTRATVNVWSIGRDPRWWERPEVFDPDRFAARSVIDVKGQHFELLPF

GSGRRMCPAMGLGLAMVELSLARLIQGFEWNLPAGLQELNMEEEFGVTLRKRVHLSALAM

PRLKAELY

>CYP787D1 Selaginella mollendorffii

MASFVALFLLTLSLGLLWRILTKIVDRSLPPGPPRVPLLGHLHLLGVLPHKSLSDLSSRY

GPVMLLWFGFAPTLVVSSPDAAREVLCTQDLAFASRPKISIAKYMFYNSKDLGWTSYGPY

WRLMRKVTTVELFTAKRLEESRMVRHAQVSKLIGFIVNNGQNGKASVNMKFLLSILNLNV

VSLITFGREFPAGSVELIEEVMQLMGSFVLGDCFPFLSWLGSPVIRKMISAHTKLDQLLQ

EIVDEHKSKFKSSERARDFVDVLLSLEDQGEIDIQCVKAMIMDMMLAGTETSAITTEWAL

SELMNNPTCMIKAQKEIDTIVGRERMVVEADLCKLSYINSVVNEVFRLHLPAPMLLPRHS

TQDCLVNGYKIPKNSRVLVNVWSIARDPSLWESPNLFNPDRFAESSISFKGKNFELLPFG

SGRRICPGLSLGVAMVSHTLARLVHGFEWKVSGKELSMDEISEGVAVRRKVPLEVFATPR

LASHAYL*

>CYP788A1v1 Selaginella mollendorffii

MNLSSIMGEYTQHDNFTAVASLSLVLAAAIALLAALFSRLRNSKRPPLPPSPPSKLITGH

LHLLDQLPNQSLYKLAKIYGPLIQLRLGVVPVVVASTAEMAREFLKVNDSVCASRPRMAA

QKIITYNFTDIGWAAYGAHWRQLRKICTLELFTHRRMQETAKVRARELADTMAGIYRDRE

TSINMNTRIFSLTMNVINQMVMRKKPFSGSDTKEAREFIDLINGVFMVWGAFNIGDYIPG

LSIFDFQGYIGMAKVLHKKLDHLLDKVIEEHIQRRMAKSDEPPDFVDVLLALTLEDGSKV

SHKTIKGIIVDMIAGGTDTAAVTIEWALSELMRKPHILKKAQEEMDRVVGRDRVVDESDL

PNLPYLECIVKEALRLHPSVPILRHESIEDCVVAGYRIPKGTGIMINVWAIGRDSATWEN

PMEFDPDRFISAGNTLDVRGNHFDLIPFGSGRRMCPGMPLGISMLQMSLGRFIQCFDWGL

PPEMKSAEEIDMTETFGLTVPRKYPLHAVPIPRLPAHLYQA*

>CYP789A1v1 Selaginella mollendorffii

MVEIPQLLSSSTPELYLKLAVAGSLLVLLLLLLNLPSSRGARRKSSSSSSSSGSSSSSSP

PLPPGPRGWPIIGNLLDVGTVPHEGMMKLTRAYGPLVYLRLGAIPHVVSDDPAIIKEFLK

IQDHIFASRPGNVILAELLTYGGKDIGFAPYGAHWRNMRKICTLELFSAKSVDSFQRLRR

MEMIHTLGLILDAAVDRRAVDLRDAFNGLTSNMMTRMLLGKRYFGPGDPGPEVGAELKAM

IAEGILMMNGFNISDYLPFLRFLDLQGQERRMKQIMRHIDGLATALLLELAPRIGKKPES

FVDILVNLRGENGEPHLPEDVMKAVMVDMMAAGTDTPGVSCEWAMAELLRDPALLARVRE

EVDRVVCVDRLVDESDLAHFRLLRAVLKESFRLHPVGAILIPHLAMEDAVVAGYGIPKDT

RVLINVFALNRNAQVWERPHEFDPERHLRGLGEGAVVEFGDPECRLIPFGSGRRMCPAAS

LGLTMVLLALANLVHAFDWEVPANLSMERAPGKMVKAQALTALARPRLPRHLYSQQI*

>CYP790A1 Selaginella mollendorffii

MDSLLLELPLVAFIVSLFCFILNRFSRSKQQFSLPPSPPALPVIGHLHLIGDLPHHSMLE

LSKKYGEFMFLKLGSLNTLVVSSPDAAKIVLKTLDPEFAMKPEHLEAKYASYGGRGIIFA

QYGEHWRQARKLCTVQLLSTKRVESAEPNRKLEMGLLLADLWKCADDGAVVNLTNKLSDF

AFNVMLKMVTGKSHSSSASSRDEEEQARSIKEGLMEFVREGTGMHIATFFPWLTWVDKQV

YKLASVHKRVDKILESEIDRHREKLGKSQPSMQHENFIDVMLMDSDANDAHIKAMTVDML

AASTDTASITSEWAISELLNHPAALAKVQAELDEVIGQERTMQESDIRSLTYLQAVINET

LRLHPPVPIYPRENSAQACMISAKWGVPARTRVFINAYAIGRDETLWKEAHRFKPERFLE

EKVGIDARGQDFELIPFGAGRRMCPGMQLGHTNVMLAVGSLLHAFNWIIPGADNGTGKVD

MQEHFGMTVARAAPLQLLPVPRLPAHALALKPGI*

>CYP790B1v1 Selaginella mollendorffii

MVMEQVCTKPQLPPQLPPSPTGLPFIGHLHLLGKLPHQSLLKLAQQYGDVMFLKLGKVNT

LVVSSSDSAKEVLNTQDHIFGSRPKTTFSETIGYGGAGLAFANGENWKSTRKVCMYEVLT

TKRVESFHPIRKFEVSLFMNELLKASREGSAVDLSSKLSDLTFNVISTMVLGKSYSASAL

SEAEKKETMFFKETLDEAAIMAGFHAGDYLPIPDWMDTQVNKIKQLQRDLDQFIQKEVES

HRQRRDPGQAPRDFVDVLLSNSHISDTSIKALIVDMVGGGTESSAVSVVWALAELIKNPR

LMERAQRELKEVVGEDRSLEESDIPNLPFLQAIVKETMRLHPPGPLLIPHESTEECEIGG

YTVPARTRTVVNIYAIARDEDNWEDPLNFDPDRFMGSNIDLKGRHFEYLPFGSGRRICPG

LMLAMATVQFILGSVLHGFNWRLPSGQTIDDLDMSESFGLTVPKAVPLKLVPSPRLEPQI

YVKSLSS

>CYP790C1 Selaginella mollendorffii

MEFLRLALAFLKALVFKLGCMSKSSSKSFSLPPSPRAVPLLGHLHLLGKLPHQSLQKLAS

RYGDVMLLKLGSHRTLVISSAEAARAVLKTHDHVFSSRPSTVAGKIFGYGGAGLVWAPYG

EHWRTVRKLCTLELLTAKRVETSHPVRKREMAFVLDELSRHQQSDKQLEPVDLTTKLSDL

TFNIMTRMVMNKSYLTGTSAEKEAAVRFKDLITEAFVVGTSCLSDSFSWLAWVDPQARKM

ERIHQQQDAYLSKQIAEHRQQPGSNGDFLDVMLAMEELSDTSIKSLSQDMLGAGTDTTAV

TVEWALSELVKDPALLRRAQEELTEMVGDKAMVDESDLPKLRYLQAVVKETLRLHPAGPL

LLPHESAEACVLENYTIPAKTRVIVNAYAIARDSRWWDEPLKFDPERFLEKCQGMDVRGQ

SFEYLPFGSGRRGCPGVTLGMTTVMFILANLIHAFDWKLASGEEMDMTEAFGVTVPRASP

LKLVPSSLNLEFPPKFKS

>CYP790D1v1 Selaginella mollendorffii

MLEMILTIVLTLALILVVLFCTNKRNQSLPPSPRALPIIGHIHLVGKKLPHEYLFRLAKQ

HGGLMYLQLGRIKTLVASTPAAAEEVLKTHDRAFASRPANSAAKYFGYDATDLVWAPYGD

HWRHLRKICTLEFFITKRVQMFQPVRKLEMSMLITELVEACNQRRPVDMTSRFFQFAFNT

MSRMVLNKSISDASGSESEKLKEFLNNLNEASKVGNGLQIGDLIPCLSWADPKVFRIKWL

QTQLVNYLGEQLQEHKKNRESHDEVKDFMDVLIAGGVLDDTRIKALTSDMLAAGTDAIAV

TIDWALAELMRNPELMQEVKQELEEVVGSKGTVEEEHIPKLEFLQAIVKETLRLHPPAPL

LAPHESVESCNIWGYNIPAGTGLLVNAYALGRDESTWSEANKFNPKRFLETKSDVQVTGQ

NFELIPFGSGRRMCPALSMGLTMVHYALATMLHTFEWSLPDGKDEVNMKAYFGIVLIREE

PLMLVPRLAKSCP*

>CYP791A1v1 Selaginella mollendorffii

MEGLFQIGTAAIFLSWIAWSLFFAPRTRIYRGNLPPSPGFALPIIGHLHLLGNLPHVSFI

ELAKRYGPCLMLKLGSYPSLLISSPEFAREALKVNDIVFSSRPSLAASRILADNAAGILW

APYGQEWRNLRKLCSLELLTSRRIEESRPVRAAEVAAAMARAKEISKAGICVNLTSLLED

LTFDIMRVWVMGSSESSRSSAGVYKRVMKESFVAGGEVHVGDYVPWLWWLDLAKVARMKR

VHGEIDDIIQKEIDEHCGKRSGSDDFITATLRNNEICRTDRDRKGLITDVIGGSTDTSAL

TVEWAMAELINNPRSLERAQDELLQTFGKNSLVEEDRLEELEFLTAVVKETLRLHPTAPI

LIYETTHECQLERYTIPPKTRVFINIYGIARSEASWSDPLAFKPERFLGSGAIDVRGRDF

EVLPFGSGRRGCPGIQLGFTMVMLVLANLLHGFHWSLPPGLSRLDMSEESGLTIPRAIPL

ELLAVPRLDARSYSV*

>CYP791B1v1 Selaginella mollendorffii

METWIAPLAVLFGLSVALLLKSWILRQPSGGAGCSLPPSPRALPLIGHLHLVLGKHLHRA

FGEIAREHGPCVFLKFGSSPHLVISSAQAAREAIRVRDSTYSSRPFLSPAARAEEMALAV

AKLVEASRGPMPRTVSLTQVISDVTYGMILRKVVGNGHRRNEEALRFKHLLKEVFVAAGE

FYVGDTMPWLQWLDLRKAAHAKRLYKQVDEYMQRLVDEQRRKGGDIGDDFISIMLRNELF

SKSDSFMKAIVLDMIGAGTDTSAVTIEWAMAELINNPRIMSRLLEELHSVVGPSSLKVEE

AHLDKLVYLDAVVKETLRLHPPAAILIFQAAQPCQVMDYFVPEGTRVFINNYEIARDERC

WEEPLKFKPERFVERNIDIVGVRDFEMLPFGSGRRGCPGIQLGLRVVHFVLANLVHGFEW

KNPSGKELDMSEGSGLTLPRAVPLELTISSRI*

>CYP791C1v1 Selaginella mollendorffii

MEFHYWSLSIFSLLAILGTVWCLVRILYPSKRYKGLPCPRMFPVIGHLHLLRRDPHRVLL

ALAREFGRCMYLKLGQYPCLVLSSAEVTKEALQGHDIAFSSRPALSAARIFGFNGSGVLW

APYGEHLKMVRKLCILELLTPRRVDSFESIRAEERSRFVSQLRDIANRNEAADLTAMLLN

MTLNIMMRIVLGTSSATVDKETSTVKELIAEAFVSTGEFLVGDYLPWLSLLDTKKKSRMK

ALKEQMSSYLQKQIEEHHNQNDKSADFMTLMLQSPEIGSNDVAIKAVIAVQDMIAGGTDT

SAITVEWALAELLKHPDLMAKAQEELDNVLGRKSQVQGGHLPKLEYLAAVIKETLRLHPP

GPLLIHETTQNCQLKNVFVPQKTLAFINLYALGRDESTWVDPLKFDPNRFIDKKNDGCGH

DFGDYLPFGAGRRGCPGMHlaltvvsvtlasllYGFNWKMPDGMSFEHLDMSEGAGFTIP

RALPLKLVPLPR*

>CYP792A1v1 Selaginella mollendorffii

MEHITAFFTFLASFILVIFYYDRSKSRSSHVMPPSPRAFPILGHIPLLASNSRGPHLILF

DLAKKLGPIFYLRLGYTPTLVISSAKIAQEILKTHDRIFSSRPSLTFAEAILPDDLIFAR

YGARWRELRKICTLELFTARRVGSFAAVRQAEMEKFLAMLSQNLRRTVNMTQELSVLTLE

IMQTLVFGTSRTFGANDFLRLAHQANELGGRLHIGDYIPWLKWMDLSLPKLRTLATKFHA

LLQAHIEEHRSSIAKQGHGGESFLDVLLSLDNMSDLTIRCLMLDAVSAGLDTTATAIEWA

LAELLLHPQILAKAQKELDDVIPASSAMVSEADIPKLKYLGAIVKETLRKHPPAPLMVPR

ESTTDCKVTGYTIPAKTQVLINLYAIARDPNIWENPLEFIPESMSSEFNAAVELMTFGFG

RRSCPGMNLGLAAVHLVLANLLYRFNWTTPDGKEVDVGESGSRSCVLVHLHWFHFSASNE

TY*

>CYP793A1 Selaginella mollendorffii

MEVFLLLLVVITFGFFLRTRNRNNILPPGPLAIPFVGHLHLLLKGHPHVVLKALAEKYGP

VMFLRFGVVPIVVVSSSQSAVEFLKVQDKVFTSRPRFLSAGRLLLGFDGEDMVFAPYGMR

WKQLRRLCTTKLFTARNFADVRMSEVRSLVRAIQAFGEASPNSALDLRTKFKHLTFNIIT

RMLMSKRYFEGDTADSKEAEEFIYLMEESFSLAGAFPVSDYLPYSFVKWLNMNQDDRIKT

LSVRSRQFVDKIITEHELRSPTCSDDFLGLLLKLRSTEDVLQRNTIRGLMINLLQAATDT

SSVSLEWTLAELINHPACMSMVQDEIASVVGSNRMVEERDISKLPYLQAIVKESLRLHPP

GPLLLPRECSKTCEVMGYKIPEATTLMVNAYAIGRDPKVWKEPLKFKPERFLDYSCFDVG

GNNLDVIPFGAGSRACPGISIAFSILHLALANLVHAFHWTLPAEVVHVDTSNEKYGLTVT

LAKKLEAIPLCKIDTIVCGDE*

>CYP793B1 Selaginella mollendorffii

MSLRFGHVPVVVASSPAAAKEFLKTHDAAFASRPLSAAGRTIVHYNADIVFAPYGDSWRH

LRKIATLELLTARRIDMFRGARMEEVRSMCRSLLVADDREMGVVDVRGQVTALTFNLITL

MLMGKRYFGKDIENEKGAKKFLEVIASTFKVCGEFPIGDYFPWLPKFLDPAERRMHSLAK

SLHEFLSDNIIEHENKRKNKKKNNNDEDFLDILLSLKDNGDEHLQNENIISVMTNLVTAG

TDTSAVTLEWAMAESIKNPTIAAKAREEIELVLGEKWRTKMVEEPDLSQLTYLQAIVKET

LRLHPAGPLLVPHQSTEAVSNVMGYHVPRGTTVLINAYAIARDSSAWGDDALLFRPERFL

GTDLDIRGRDFEAVPFGSGRRQCPGMALALTTVHLTLANLLHGFEWREPSGESIDTSKEQ

YGLTLLLAKKLRLIATPRLEQGTL*

>CYP794A1 Selaginella mollendorffii

MGAFGLLLYLRNKMKKIQGNKQQLPPSPQSLPIIGHLHHFVSSGKEPHQLFQSLAAVHGP

IFSLRLGYMNVVVVSDRSTAKQVLKTNDLALASRPKLISVKHALYNFQDVVFSDYTKELR

EIRKFLAMELLSAKKLDMFTNVKEDELSWLVLTLANASEQLNTFKMRDYLVGLTYNVITR

MLMGKRYYGAPPDDKEYEEGVAFKKVVDDAIKIGVAGSIADFFPQLEFLDWKVSQAKKVQ

RELDKFLQRMLDEHRVPNRGNSQEDFLDMILEASFMSDDRIKATESMTLLHLQDLITGGT

DSSSSFLEWTLAELIMHPQVLAKAQEEIDTVVGHGRKVKESDIPRMPYLQAVIKEGFRLH

SPVPLLVPHYANQECSINGYTIPCNTTVFVNTYAMGRDPKVWDNPLEFDPERFLSGPHKE

VEVLGQNVNFELLPFGSGRRSCPGSALGNSIVHFTLATLLHCYDWKAGDKIDFAESSGAA

KIMKFPLCVQPTPRLQIQDMYVTNQYTNPIHM*

>CYP795A1v1 Selaginella mollendorffii

MEFLVGFAILLILVVFSSVFYLRVASQSPSLPTPLPIIGHLYLLGKLPHHSLLAIARKYG

PLVQLRLGSVPVVIASSPEMAREFLRNQDLTFASRPTLLTTKYILYDSKDMVFAPYGEHW

RSMRKLCVVELLTDRRLASSQQARLEELQRLLAKIAKVVETSEPFLLLDLLTEFTFNVIT

RMVMNKAYFGSGETMEELAATRDFIHMQEQGTILLGEFHIGDYIPFLKWFDSSVAKMKAL

HKIQDEFPQKVVDQHVLARQSREQTQAHDGDGDFVDTLLSLDSPDPNNQARNIKALIQNL

LGAGTDTSITTIQWAMAELLNNPRALEKAQEELRAKFGNARQEIIQEHELKDLPYLHAVI

KETFRLHPPAPLLIPHQSTQDTTVAGLAIAKGTRLFVNVYAIGRDPALWKSPDDFLPERF

LGSSIDVHGKNFELLPFGSGRRGCPGMALGLITVQLALANLLHRFQWSLAPGVDAHPMAE

CFGVVTTMEIPLRARASPNKD*

>CYP796A1v1 Selaginella mollendorffii

MASLVNATAALQDEGPASNTIRATLTIALVAAVIAWWAIAKSRYGLKNLPPGPRGLPIIG

HFHLIGRLPHVSLQQLSAKFGPLMSLRFGFVPVVVVSSPAMAREILKTHDTAFADRPYKI

AANFIFYGQRSISWSSYGDHFKKARKLCATELFTARRVTSFTHVIRDELWKLSGELRAAS

ASGEVVKLRRHLRGLSFNLMTRILMKKVYFGPGASTDESALQEAKEFVNIIDSVLTVGGA

FAITDFFPGTKWIDWTVPAAKAASDKLNSFLTKVLDEQRPGEVPDFVALTKSYFDGPDQM

KYTKALLVDMFLGGSETSSTVVEWAMAELLHYPKVIAKAQEELERVVGRERMIEESDLPK

LEYFSALVKEVFRLHPPLTMMVPHTTAQNQKVAGYDIAKNSMIFVNVFAIGRDPSVWSNP

LEFNPDRFMGTSFNVHGHDFELLPFGSGKRGCPGLPLGLRNVQLVLSNLLHGFDWSYAGD

IEKHQMTEAMAVVNFMEHPINVRASPRLDDATYKTLSINT*

>CYP796B1v1 Selaginella mollendorffii

MFWAVHIVVFLLTAFILKQWLSSISLNLPPGPRGLPLIGHFHLLAMGKIPHIALQQLSKR

FGPLFHLRLGSVPVFVVSSPEMAKEFLKNHDTEFAYRPRNNVVSIVMDSRSMSFSPYGDY

WKKLRKLCATEIFTAKRMSMNTQIIRDELWELSGELLRASKAGQVVGVRPHLRALSFNVM

TRILMKKTYFGSKASGDPAIAAEASNFIAMIDEILEVGAAFSITDYFPYLSWLDLVARRA

KVAGDKMNGFLQKVLDEQRPGEVPDFVEVTRSHIGNDLVSLRALLMDLLLGGSETSSTVT

EWALAELLHHPDWMVKAQQEIESVVGRTRMVEEGDISKLEVLNAIIKESFRLHPPVSLLI

PHASVEAQNVAGYDISKNAMLIVNVYAIGRDPRVWSDPLEFQPQRFIGSSIGVNGQDFEL

LPFGSGKRACPGLHLGLRNVQLVLSNLLHGFEWKFPGSPKDQTMDEAMGNISFMAHTLKA

KITPRLNESLYRLT*

>CYP796C1v1 Selaginella mollendorffii

MLPAIVLVLTLAFFVTQWMWSKRAIKLPPGPRALPLIGHFHLLGRIPQISLYHLSKKFGP

LMYLRLGSAPLIVISSPAMAREFLKTHDAAFARRPPRVAVDILMYKFKSLSYSEGEYHKN

IRRMCSMELFTARRVTSFTKIIRDELWDLTAELAKASKAGQPVALRGKLRSLSFNVMTRI

LMNKTYFGSKASSDDPQAREFVGVIDEVMDAAGAFSIADYFPSVGWLDWSIARCRRAHQR

MDAFLDKVLNEQRPGEIPDFVEMTKARVDGPEQAQYLKALLMDLLLGGSETSSTVVEWAM

AELLHNPEWMEKLQQEIESVVGRDRMVEESDLAKLELVNAVIKETFRLHPPLSLMVPHTS

PEPRLVAGFEIPAKATVLINTYAIGRDSQAWPNDPDKFKPGRFVGSNINVYGHDFELLPF

GSGRRGCPGLPLGLRNVQLVLSNLIHGFDWRFRDGATRKLSFDSGPGFINIIADAVVAQV

SPRLEQCAFGTLAAS*

>CYP797A1v1 Selaginella mollendorffii

MEHFDLALYLGLILLAGALWRQYRSFKVRLPPGPRGLPLIGHLHLLSTLPHRSLQKLSQA

HGPLMHLRFGTVPVIVASSPAMAKEVLKTHDLAFASRPYLLVGEYAAYNFHNIGLAPYGD

HWKMMRKLCSTELFTAKRIDSFSWVRVEELSGMVSGLLAKSASKEVVQIKSFLTDFTFNV

MTRILMDRAFFGPAGADSQGKAREFRGIVEEILQVAGSFNVSEYIPSAFKWIDWNIPRFK

RLHARQDRFLQEIIDEHKVGHDALAKPRDFIDILLSYFNHGDSRIDLDNIKAVLSDLLPG

GTDTSITTVEWILAELLRNPLALKKAQDELDAVVGKDRMVNESDFPKLHYLHAIIKETFR

LHPPIALLVPHMSRYECKVAGYDVPKGATTLVNVYAIGRDPTVWEDPTRFSPDRFLEGAG

KGMDVRGQDFELLPFGSGRRSCPGLQLGLKTVELALSNLVHGFDWSFPNGGGGKDASMDE

AFGLVNWMATPLRAVVAPRLPPHAYEKV*

>CYP797B1v1 Selaginella mollendorffii

MDLTLSVVVSSLLLLFILAVVIISYKTSPPGPWGLPLIGHLHLLARMPLHRALQSMSQKH

GPIVSLSLGMRPAILISAPALARELFTSQDVNFPSKPYTSVSEHIGYNFRSIGTAPYGEY

YSSIRKLCLTELFTARNIDSFSWIRREELSHLLSAILSRASHGQALDLRKTLSVFTFNSI

TGALMSKRYLSHDTGAASSKEAMEFKNWLIEVLQLVMEPSLSNFVPWYLRWLDWKTPGLR

RLHAKLDKFLQMVVEEHKKSTREQKDFLDILLKAFGEEEAYAKANLLDLMVAGTETSVTG

TEWLMAAVIQEPRILKKAQQELHDAVGNRRMVQESDLSKLGYLDAIIKESLRRYPIVPIY

IRECQGQASKLGGYDVPKGTIVIVNSWALGMDPVVWENPTQFLPERFLASSIDIKGQDFE

LLPFGSGRRRCPGMPLGLRTMKLLVANLIHGFDWSVEPGKIQSMEDCFKSTCIMKHPLRP

VVTPRLHKDAYTTQIHDFFI*

>CYP797C1v1 Selaginella mollendorffii

MELALSSFALPFLLLVLTGALSILVTSWDKKKNLPPSPGWALPLIGHLHLITKQPHRSLQ

ALSKKYGPIMFLKLGMIPSIIVSSPEMAKEALMNNGLAFASRPYLLISEIIGYDFQSIGI

HYSEHSRRLRKMCVTELIAPQKLESSLWVRFQELSRAFRILQKSNEEKVAVDMRYLFSTF

TFNAFTMILMSKRYFGDTTDDNDQHREIKHVINEIFSLAIKFHITEFVPSYLRWLDPTIP

QFKRLHERQDKFMKKIIKEHKEPTARPKDFMDALLESFSAEDTVKAFITVSSLHILLLAS

DSTAVAAEWVMAQLLHNPHVLEKAQFELNLVVGPNRLVQESDFSKLEYLQAIIKETLRLC

PPGPLLIPRSSDEACTIGGYYVPKGSTLFVNAFAIGRDPSIWERPTEFMPERFLGRSVDF

KGQHFDLIPFGSGRRMCPGMPlalkalelllanlVHGFDWSFPPGEIQTLEDCFETTlll

ksplkllAVPRHSASVYAEI*

>CYP797D1v1 Selaginella mollendorffii

MATIFGGVLVFLVLFFLTKRLSFTRQRLPPSPLSLPLIGHLHLLTRMAHQSLQVLSNKYG

PILYLKLGMVPTIVVSSPDMAREILKTHDAKFSSRPYFLVGEYFSYGYCGMGFTSGGEHW

KNLRKLCATELFTINRIDSFEWVRKEEISRMISTIENTTGVINMRNLLITYGFNVMTETV

MSKRFFCENGALLDADQAREFKKVSIETVEMALKFHISEFVPSYLRWIDWNIPKVKILQA

KSDKFMQQIVQEHKRSKNSRKTKDFMDVLLESFTDSSNKQSLKAENTVKALTMELLAGGT

DTSASSIEWALMELLLNPHTMVKAREELVKFVDLTNSTVNEGDLPKLTYLNAVIKETMRL

HPPAPLLVPHKSTVECKIAGFDIPKGTTTIVNLYAIGRDPNVWENPTKFCPERFLGDSRI

DVKGQNFELIPFGSGRRTCPGMILGLRNVQLVLANLIHRFEWALIPGREYGVEETTGTVN

WAKTPLEVLKR*

>CYP797E1v2 Selaginella mollendorffii

MEFPVYLLVALVVCFLGRSLLQSRKRLPPSPWGLPLIGHVHHLSRLPHQSLQNLSRKLGG

IMYLRLGMTPAIVISSPDLAKEALRSNDSSFGFRPYLLVGEYLTYNFKGIGLSNGDHWKN

MRKICITELFSVKRMESFRGLRLAEVSHLVSRLAQASKSQSVVNVRELVTDFNFNIHLTV

LTFNVQTRILMSKRFFGENLSDDELAEARVFKELIDESVKFAFQFHISEFVPSWLKWIDW

NIPQAKRVAAKQDEFLQKIIDEHKAKKSRPTKDFMDILLEQRGDDQEVVKAILMSFAQEI

LIAGMDTSACTVEWALLELVHNPEVMKKAQEELDVVVGRNRMVTETDFSKLTYLEAVIKE

TLRLHPPVPILVPHMSNKACVLAGFDVPKGATTIINFYSISRDPNVWEHPTKFWPERFGQ

ITADVKGQDFELIPFGAGRRMCPGMSLGLKTVHLVLSNLLHSFHWERVPGESYNLDEGVG

SVTWPKSPLQAQLTPRLRNLDVIFNFAQ*

>CYP797F1v1 Selaginella mollendorffii

MEFLVYVLLGSVFLFYLLVRPFLQPRKLLPPSPRGLPFIGHLHLLGRQPHISLQELSNKF

GDIVCLRLGLVPAILISSSAAAREALKTHDQTFSGRPYFLLGDYVYSSKSMVLSPPNEHW

RRMKKLFNAELFTANRLASFLEVRREELASMVSFLIDNQSRVVNVRELVRSYTFNTITRI

VMSKRFFGEKNTVNEEEAMEFMEVMEEIIKFGFAFHISELVPAWLRWIDWKIPAVKRIAA

REDIVIQKILDEHRKTKSSRGTKDFLDILLEHDTKGDGGGNDLDNARGTIMELVGAGTYT

TACVIEWAILELLRNPDVLEKAQHELESIVGQTNRLVEESDIEHLTYLQAIVKETFRLHP

PAPLLLRMSTQECVISNYHIPKGANTFVNVYAIGRDPGLWENPMEFWPERFVGSSMDVRG

QDFELIPFGAGRRTCAGLTLGLKVVQVGLANLLHGFDWSCVAGRDYNVAESSVSVIWPKK

PLEAIVILKSR*

>CYP797G1v1 Selaginella mollendorffii

MDLVTAFIVLLVILLPILRGLFQRSRLPPSPSFglplighlhllgRMPHQSLQALAKKHG

SILFLRLGIIPAVVVSSVDLAKEVLKNQDLTFASRPYFLVGEDVGYHFMGMSLAGYGDHW

KKLRRLYTLELFTAKRIDSFLSLRLEELSHMLSAVLYAHEKNQAVNMRNLLTCFTFNTIT

RILMNKRYFQHQGEELQGIDSSEASVFKAVLSEITEISLQFHISEFVPAYLRWMDLSVYH

MRRLHADQDKFLQKIVDEHKYEKNKSSKDFMDLMLELFDGDPKGDNMIKAALQELVSAGT

ETSATTVEWTFGEILHRAPHVLTKAHEELDSVVGRSRLVDEADLPRLPYLQAIIKEAFRL

HVPVPLLVPHMSMHEASLDGYHVPKGATTIVNAYAIGRDPALWDNPLEFRPERFLGSSMD

VKGQDFELLPFGSGRRACPGMGLGLKTVQLALANLIHGFDWKASGQNALEEAAGAVIWLK

TPLEAVGSPRLQVEVLTSCHI*

>CYP797H1Pv2 Selaginella mollendorffii

MEVAQYAVVLLITLLGLPLIGHLHLLGRILHLSFQTLSTKYGPIVFLRLGMVPAVVISSL

ELVKEVLKIQDANFALGPYLTMGEYNYNFRDIGFVLYCDYWKSMRKLCATELFTVKRIES

FQGVRTRETHGVLSELVNAADYQKPINMYVFHVVMQILMSKPFFEYREHEAEMSSKGKDF

KHIVFEITEQMLQFHISEFVPAFMRRIDWKIPEMMRIHARQDKFFKRIIDDHKARLESET

SQPKDFMDTMLQSLKSEGARGEEELLSGRDTSASLIEWTLLELMHNPLVLQTVVGKERLV

AERGFDKLEYLACKVAGYDIPKGTPTFVICFTIGRDPAVWEDALRFKPERFLGNLIDIKG

QDFGL

>CYP798A1 Pinus taeda (Loblolly pine)

mdngmmvwivlagVVAMAVWYLLVQYQQPKQSHNVPRETLPPGSVGWPFLGEIISFYFRT

PDFVKQRRGRYGNLFRTVLIGYPTVISTDPEVNKFILNNDGRLFVPAYPSYWSQIIGECN

IFVARGDFHKRMRGAFLHFISISVVKNRLLSEIQNIITFSLAGWEGRNVNVLHEAEEMIF

SVMANHMLSLSAGTALESMKRDFLVMMKGLRSLPLRVPGTTFYKSLQKKQVLFNQIKSII

EERKLNMSAYDSYDDLLssiLKSASEKEFTTTQIVDLIVQSVIGSLETTPKIMASVVRHL

SENPHIIKYLKEEHETIIQAKENNQSLSWDDYKSMVFTKSVIKETLRFGmqplnNIMFKK

TLQDVKIEGYTIPKGWTCIIYDLVSDMDNKYCKDPLSFNPQRWQSKEMNEVPFLAFGGGP

RLCPGYELAMLTMSFFLHHLVTKFRWEYLPSKSELRWFDSPLNSVFDCRIH

>CYP799A1 Picea glauca (white spruce)

AVQYLTQKPKALQQLRREHEAILLAKKQKPEKILTWEDYKSMEFTRSVIKETLRLSNVGP

FLFRECVQDTEIKGYRIPKGWKVVACTTAVSHDPAIFPEPSCFNPWRWQEDMAEEKHLQV

FGGGSRYCVGAELAKLEMAVFLHHLVTKFSWDICEGEIIRSPLVLFKDGYPISVRKRAS

>CYP800A1 Ostreococcus tauri

MGGAVRRTRAGRFGAASATETAKAEDDEVASAEVGVSVGASAMGGAEDEASGTACPYTAA

KEALGLAPPPPTRRDENGNPAMVDNVSFLKSLIKASQHPVGMPIAMLDWAAEKGECVGIK

NAIGPFCVSILDPEIVEYVCFTNAKNYRLRMLPDAFRYVIQNKGITGSDGQYNRDHRLMC

QKPFINSFSLAEFSSTVEERIAHMCNTWQQAHAMGGGKPYEINIDYDSQQVTLDVIGKVA

FAYDFKRCEAHEAKTLRGEADEDGNVSKLLAAYNGSSEIMGELFITPGPILKLQNFLGLG

RVRELKEQYAILESVGTKLMSERRAIVKERLAAGDEEDYCLLDVLVKAKDADGQPLSDAD

LWGDINDIMAAGHRTTASNFTVNLWHVARYEHIQEQIEKEVAALGGRPPTFQDVQEGKLP

YTQRVVKESLRKYAPINLFPRLAEGPDTLPSGHKVEEGDFILLSTYAMGRNPRVWEDPNK

FDPDRFTDEALYAQAEKQASAVARGDPAKLEQARERMRRRMNAGRDFTYTPFGAGPRSCI

GGVFALLAATTMLASTVQKFKLSKASHSKGAVGEELNIMYDTTICFPEGVWINLEPRERP

LGA*

>CYP801A1 Ostreococcus tauri

MRSSEVIGNLVNAFDVASNASTIAAVALLGIFTLGLFFILFVRARARATLARASVPAVTW

RPKFLWRLYSRSARSLLQRVEQRFRRGDGSGRGRRAFGAVVGACPFVHVGEARLARDVLR

ETSRKAPLYHAFEAFSGTGIFTAEGDDWDGKRTEVLRAFHDVGLASLRDRAVEESASAVE

EMREVVERGGGEIEARALPILQRLALRVTFAYLTGVSLRRACEDVGRERSEVEGEYLDAA

TTLRHLIPARARSIWIFSDLLYGLTPVGRLEARKIRTTRSLSALALRTAKEESPLGRLRV

GEAHLREKSIKVGKDEYPKGLLDEATTLLFAGHDTQSATLSWCLLRLVQDVEVQSELRAS

LRDDIIEEALGLPPSKSGSRQRRSSEKTTKPAWATSAFAPTLEAVIRETLRLHPVAPLVV

RMLSSDTHSEKMTIPKGCAVGVWLSSVHRDESVWERPEEFDPKRWFGGPAHARTGSMGGS

SNDDDDNGGILTPSRDRSNALRHKGVGYMPFAYGPRSCVGQHLAQVTMRVALAHLVHAFE

FAPSADLDASMPSVGFTVTPSTGAPVRVRLAARAPA*

>CYP802A1 Ostreococcus lucimarinus

MASRATARAAASHDARAATRRARVGGAARESAARRRASGRATSDVDGASGRATSDVDGAS

GRATSADAFDLGQAIGTLARGERGATLPPGRVGALGVRETLEYLADSNGFVRRRVERYGP

IFKTALFFKPAIVFGSREAVREFLKFEGELPADEALPETFRELHTEYGALRMTGSRHAAT

RANFGKVLGRAALESYAPAIGERTREFVEDVARRSGKETSSFRPGAECVDFALDLLFELF

LGHVPEAKYKDAMKAYNGGLLSLGKWSSEFKAGKLALEDLTSYVEAHYRGVKARGELDRP

EYFFYKQYSQAVDEFDEVFSDDRIATTCVLMVWGSYIEAAALMGHACVLLGEHDDARRAV

LREFERVCCDDENGCRRIGTLADIMSMQYTSAVAKESLRVMPQTAGGLRVNPSPRKFASF

DVPAGYVLTADPRIPFRDEANFPDPDAFKPERFVPGTHEAKQNDVSSETYYPGGMGQHQC

PGISLATVMTQIFLAELVSAFPNGWRGKTAPKYVQVPIVILDREYEIEFLR*

>CYP803A1 Micromonas sp. RCC299

MSALVANAVGPVAVARRPPRRMRHAPRERVSAPRASRADDLPGILLGAAAKKLEEDVNSF

IGLFDEDAPTHERPPTLPVAGNTLDIAQGGHRQLLEWAETYGVADGVHEVKMLSQTILHL

TDPKLARELMFERSDSFPDRGVSAMAKFFREDQAAFVNTSGEQWMAYRKMGTATVNGGAL

DRLAGKVAERSEALVTRWVRDASASGDGRNATEVDISDASQAVTLEVIHEALFSEQLDVI

DGERNAVALARSFREFNVANQDLLNDFLTLYQRFETPERARRDTHRRRLRAHFDERADAR

RAAIARDGAAAAPRDLLTALLTARDPATGAALTRDDVNLTLTEMMVAGHDTTAATVACMM

CLLASHPEVRASVTGEVDEFRRNNGGRLPSSVADANALVKLDDAMKETMRLYPAVLIVVR

KAEEGTGGVFTKGPGREVRIPEGSGMWVSPYVLGRLARHWGGDEEDVKRFRPSRFEEARE

RGDSLDAYMPFGGGPRVCLGSRFAMLEGKVLAAHILADWDVELAAETRDAIARNDGELPI

AYAAGLMSFPEPLRLRVRRRGAASGPR*

>CYP804A1 Micromonas sp. RCC299

MGGYFTSDRAAKYGWGVPRLALVTLFAFEIVPRAFLAGSRAPVDALVSTPLPFTARAAAA

PFAPLTLVPYLHSLRWIAHAYTGSIGAGVNVQVLGLLGNLAIMAVAGGLCLWDLTKDISL

VVYFLKTFCGDQLAGKLDTAEWKLVLAFTFALPLAMWGAAGANGAAFAMFLPYARIAPIL

FAIQAVCEFGDAHLEYHPVIGKFFRHRYGFEAFTLCALTMMPGGITAPELRVVQFDLVIC

LFYRVANLGIILHKQGFAVAAAASLAVTIRKGCFRVFGALSGQRIVNVTDAEVATAVMRA

SDVKGDALERHVATPAWRPLLSLESVDHELYRNMLRDFHAVVKACPPPQRVGEIARAKVD

ELMYRTYSEEAEEASPVRGGAEPSPPPSPERPVVDVTLADSPVDSPHPHGGKGGDVGKCP

FVQMQRTMRGGASGQSNAAGRSTPARSDAAPVIDADDVARLSLSVFIEYLFGREWEPKFE

TLLAASWEWRKEIAVRGRADPGVKKAAVELVVDDLIKNSHLWDLFGEKWREPRYYSLIMQ

PFLVSPAINVGDIAVAMKAHPDLALEPAMRRMHPFPIFERWVDKDVVVDGRIAVRADTQV

IMFTSDFANSKHLWPAFGTGPRACAGTSMALGVLNAIHQKMLGRPGFEPERGHKFSGRNN

DGVTSLSEVWYFAKTVLPVVFGFGGEKTTEAAALERAAAAALE*

>71A12 Arabidopsis thaliana

MEMILMVSLCLTTLITLFLLKQFLKRTANKVNLPPSPWRLPLIGNLHQLSLHPHRSLHSL

SLRYGPLMLLHFGRVPILVVSSGEAAQEVLKTHDLKFANRPRSKAVHGLMNGGRDVVFGP

YGEYWRQMKSVCILNLLTNKMVASFEKIREEELNEMIKKLEKASSSSSSENLSELFVTLP

SDVTSRIALGRKHSEDETARDLKKRVRQIMELLGEFPIGDYVPALAWIDRINGFNARIKE

VSQGFSDLMDKVVQEHLEAGNHKEDFVDILLSIESEKSIGFQAQRDDIKFMILDMFIGGT

STSSTLLEWIMTELIRNPNVMKKLQDEIRSTIRPHGSYIKEKDVENMKYLKAVIKEVFRV

HPPLPLILPRLLSEDVKVKGYNIAAGTEVIINAWAIQRDPAIWGPDAEEFKPERHLDSTL

DYHGKDLNFIPFGSGRRICPGINLALGLVEVTVANLVGRFDWRAEAGPNGDQPDLTEAFG

LDVCRKFPLIAFPSSVI

>CYP71B2 Arabidopsis thaliana

MTILLCFFLVSLLTIVSSIFLKQNKTSKFNLPPSPSSLPIIGNLHHLAGLPHRCFHKLSI

KYGPLVFLRLGSVPVVVISSSEAAEAVLKTNDLECCSRPKTVGSGKLSYGFKDITFAPYG

EYWREVRKLAVIELFSSKKVQSFRYIREEEVDFVVKKVSESALKQSPVDLSKTFFSLTAS

IICRVALGQNFNESGFVIDQDRIEELVTESAEALGTFTFSDFFPGGLGRFVDWLFQRHKK

INKVFKELDAFYQHVIDDHLKPEGRKNQDIVTLILDMIDKQEDSDSFKLNMDNLKAIVMD

VFLAGIDTSAVTMIWAMTELIRNPRVMKKAQgSIRTTLGLKKERITeEDLGKVEYLNHIL

KETFRLHPALPFVVPRETMSHIKIQGYDIPPKTQIQLNVWTIGRDPKRWNDPEEFNPERF

ANSSVDFRGQHFDLLPFGSGRRICPGMPMAIASVELALMNLLYYFDWSMPDGTKGEDIDM

EEAGNISIVKKIPLQLVPVQRY

>CYP71B3 Arabidopsis thaliana

MSILLYFFFLPVILSLIFMKKFKDSKRNLPPSPPKLPIIGNLHQLRGLFHRCLHDLSKKH

GPVLLLRLGFIDMVVISSKEAAEEVLKVHDLECCTRPKTNASSKFSRDGKDIAFAPYGEV

SRELRKLSLINFFSTQKVRSFRYIREEENDLMVKKLKESAKKKNTVDLSQTLFYLVGSII

FRATFGQRLDQNKHVNKEKIEELMFEVQKVGSLSSSDIFPAGVGWFMDFVSGRHKTLHKV

FVEVDTLLNHVIDGHLKNPEDKTNQDRPDIIDSILETIYKQEQDESFKLTIDHLKGIIQN

IYLAGVDTSAITMIWAMAELVKNPRVMKKAQEEIRTCIGIKQKERIEEEDVDKLQYLKLV

IKETLRLHPPAPLLLPRETMADIKIQGYDIPRKTILLVNAWSIGRNPELWENPEEFNPER

FIDCPMDYKGNSFEMLPFGSGRKICPGIAFGIATVELGLLNLLYYFDWRLAEEDKDIDME

EAGDATIVKKVPLELVPIIH

>CYP71C6 Triticum aestivum (wheat)

MALEAAYHYLQRAVGHGTSTEALLLTVLLLLIIRVAWVRAFTTTTASTKCKQQLPPTPPG

KLPIIGHLHLIGSHPHVSFRDLAAKHGRDGLMLVHVGAVPTVVVSTPQAAEAVLRTHDHV

FASRPRNPVADIIRYNSTDIAFAPYGDYWRRARKVVNTHLLSVKMVYSKRHDREEEVRLV

VAKICELAMAAPGKALDMTELLGGYASDFVCRAVLGESHRKHGRNELFRELTEISASLLG

GFNLEDYFPRLANLDVFLRVVCSKAMGVSKRWDNLFNELIAEYEHGKEDNAEDFVHLLLS

LKKEYGLSTDNVKAILVNMFEAAIETSFLVLEYSMAELINNRHVMAKVQKEVRESTPKGE

KLDLIMEEDLSRMPYLKATIKEAMRIHPPAPFLLPHFSTNDCEVNGYTIPAGTRVIVNAW

ALARDPSHWERAEEFYPERFLQEGRDAEVDMYGKDIRFVPFGAGRRICAGATFAIATVEV

MLANLIYHFDWELPSEMEAIGAKVDMTDQFGMTLRRTERLHLVPKIYK

>CYP71C12 Oryza sativa (rice)

MAQMLDGLRHDEQASLHAPQEASTMPTMSCSDLLLAMMCPLILLLIIFRCYAYATRSGGM

LSRVPSPPGRLPVIGHMHLISSLPHKSLRDLATKHGPDLMLLHLGAVPTLVVSSARTAQA

ILRTHDRVFASRPYNTIADILLYGATDVAFSPYGDYWRQIKKIVTMNLLTIKKVHSYGQT

RQQEVRLVMAKIVEEAATHMAIDLTELLSCYSNNMVCHAVSGKFFREEGRNQLFKELIEI

NSSLLGGFNLEDYFPSLARLPVVRRLLCAKAYHVKRRWDQLLDQLIDDHASKRRSSMLDN

NDEESDFIDVLLSIQQEYGLTKDNIKANLVVMFEAGTDTSYIELEYAMAELIQKPQLMAK

LQAEVRGVVPKGQEIVTEEQLGRMPYLKAVIKETLRLHPAAPLLVPHVSMVDCNVEGYTI

PSGTRVIVNAWAIARDPSYWENAEEFMPERFLSNTMAGYNGNNFNFLPFGTGRRICPGMN

FAIAAIEVMLASLVYRFNWKLPIDQAANGGIDMTETFGITIHLKEKLLLVPHLP*

>CYP71C14 Oryza sativa (rice)

MAVMLVPIPLLLLHQHHNHEHEHPSPVAPQPTMASYYTLLLALLCPLLLLLIKLCRAKTR

DDELFDKLPSPPGRLPVIGHLHLIGSLPYVSFRELAIKHGPDLMLLRLGTVPTLVVSSAR

AAQAILRTNDHVFASRTYSAVTDILFYGSSDVAFSPYGEYWRQVKKIATTHLLTNKKVRS

YSRARQQEVRLVMARINEAAVARTTVDLSELLNWFTNDIVCHAVSGKFFREEGQNQMFWE

LIQANSLLLGGFNLEDYFPNLARVTTVRRLLCAKAHNVNKRWDQLLDKLIDDHATKRSSS

VLDLDNEESDFIDVLLSIQHEYGLTRDNVKAILVIMFEGGTDTAYIELEYAMAELIRKPQ

LMAKLQAEVRSVVPRGQEIVTEEQLGRMPYLKAVIKEMLRLHLAGPLLVPYLSIAECDIE

GYTIPSGTRVFVNAWALSRDPSFWENAEEFIPERFLNSIAPDYNGNNFHFLPFGSGRRIC

PGINFAIATIEIMLANLVYRFDWEIPADQAAKGGIDMTEAFGLTVHRRRSSSLFLGSHKI

K*

>CYP71C15 Oryza sativa (rice)

MELNNTEPLTASRAQAAAVFLLLPVALLLLLLRFARATTMAGDRNSELLLSKLPSPPLRL

PVIGHMHLVGSLPHVSLRDLAAKHGRDGLMLVHLGSVPTLVVSSPRAAEAVLRTHDLAFA

SRPRAMVPDIITYGATDSCYGPYGDHFRKVRKAVTVHLLNSHKVQAYRPAREEEVRLVIA

KLRGAAAMAGAPVDMTELLHSFANDLICRAVSGKFFREEGRNKLFRELIDTNASLLGGFN

LEDYFPSLARTKLLSKVICVRAMRVRRRWDQLLDKLIDDHATRLVRRHDHDQQQDSDFID

ILLYHQEEYGFTRDNIKAILVDMFEAGTDTSYLVLESAMVELMRKPHLLAKLKDEVRRVI

PKGQEVVNEDNIVDMVYLKAVIKETLRLHPPAPLYIPHLSREDCSISGYMIPTGIRVFVN

AWALGRDAKFWDMPDEFLPERFMDSNIDFKGHDFHYLPFGSGRRMCPGIHSATVTLEIML

ANLMYCFNWKLPAGVKEEDIDMTEVFGLTVHRKEKLFLVPQAA*

>CYP71C16 Oryza sativa (rice)

MELILQLEAKTAAQAVVTVFFFFLLPLALLFYFARAAISSRDSKTRELILSKLPSPPFKL

PVIGHMHLIGPLPYVSLRDLAAKHGRDGLMLVRLGSVPTLVVSSPRAAEAVLRTHDLAFA

SRPRSMVTDIIMYGALDSCFAPYSDHFRSVKKVVTVHLLNSKRVQAYRHVREEEVRLVMA

RLRGAAAAAAAVDLSQTLQFFANDLICRAVSGKFLCEQGRNKVFRDLMEANSNLLGGFNL

EAYFPGLARMPLISKLICARAIRIRRRWDQLLDMLIDDHVASARDRAKNDDDDFIHVLLS

LQDEYGFTRDHIKAISIDMFEAGTDTSHLVLEYAMVELTRKPHILTKLQDEVRRITPKGQ

HMVTEDDIVGMVYLKAVIKETLRLHAPGGFTIPHLAREDCNVDGYMIPAGTRVLINLWAL

SRDANYWDKPDEFLPERFMDGSNKNTDFKGQDFQFLPFGSGRRMCPGIHSGKVTLEIMLA

NLVYCFNWKLPSGMKKEDIDMTDVFGLAIHRKEKLFLVPQIANY*

>CYP71C17 Oryza sativa (rice)

MVVQLMLFFHDKFMAPMAEEPLPFVLIMIIILLLLVLLHYYLSASTRRSSAASKSNDDVL

PPSPPRLPVIGHMHLVGSNPHVSLRDLAEKHAADGFMLLQLGQVRNLVVSSPRAAEAVLR

AHDHVFASRPRSAIADILAYGSSNISFSPYGDYWRKARKLVAAHLLSPKKVQSLRRGREE

EVGIAVAKLHEAAAAGAAVDMRELLGSFTNDVLCRAVCGKSSFRREGRNKLFMELAAGNA

DQYAGFNLEDYFPSLAKVDLLRRVVSADTKKLKEKWDSVLGDIVSEHEKKSSLRRDDQVQ

MDDDRDDDQEEQESDFVDILLDRQQEYNLTRHNIHAILMDMFAAGTDTSYIALEFAMSEL

IRKPHLMTKLQDEVRKNTTTQMVSEDDLNNMPYLKAVVKETLRLHPPVPLLLPRLSMAQC

NANGYTIPANTRVIINVWALGRDAKCWENSEEFMPERFMDSGDTIDNVDFKGTDFQFLPF

GAGRRICPGMNFGMASVELMLSNLMYCFDWELPVGMDKDDVDMTDQFALTMARKEKLYLI

PRSHVIKIT*

>CYP71C19 Oryza sativa (rice)

MEQAAGLVYQLFQHEMFPWTFSVLALFPFLLLVLHYLATNHRTPTTCKETKNHHPPPPSP

PRLPIIGHLHLIGGLLHVSLRELAHRYGPDLMLLHLGQVPNLIVSSPRAAEAVLRTHDLV

FASRPYSLIADILLYGPSDVGLSPYGE*WRRRIITTHLLTNKKVRSYRVAREEEVHKVMA

KVHELSTKGMAVDMTELFSTFSNDLICRLVSGKNFQGEGRNKLFRQLFKANSVLLAGFNL

KDYYPGLARLKAVSMVMCAKARNTRKLWDELLDEIIDERMSKQQCEHDEGNDQDEMNFVN

VLLLQEQGITREHLKAILVDMYQAGTETSSVVLVFAMAELMQKPHLMAKLQAELRTTIPK

QGHELITERDLTDMTYLKAVIKETLRLHPPTPLLLPHLAMADCNIDGYTVRSGTRVIVNA

WAIGRNSESWEAAEEFLPERFVDDGSAANVDFIGTDFQFLPFGAGRRICPGINFASASME

IILANLLYHFDWDVSAEAAIDKDGIDMAEAFGLSVQLKEKLLLVPVDYKDGMQDSAVILL

*

>CYP71C20 Oryza sativa (rice)

MAQMLAAFLLDGLISHEHGHESLGAPPQAGTMAWYSLVLMTSLLFPLLVLLVMRCYVTRS

GAKLLDKLPSVPGRLPVIGHLHLIGSLPHISLRDLATKHSPDMMLLHLGAVPTLVVSSSR

VAQSILHTHDDIFASRPYSPIANILFYGATDVGFSPYNEYWRQIKKITTTHLLTMKKVRS

YVSARQREVRIVMARITEAASKHVVVDLTEMLSCYSNNIVCHAVCGKFSLKEGWNQLLRE

LVKVNTSLLGGFNIEDYFPSFTRLAAVRRLLLSCAKAHNINKRWDQLLEKLIDDHTTKHI

RSSSMLNHYDEEAGFIDVLLSIQHEYGLTKDNIKANLAAMLMAGMDTSFIELEYAMAELM

QKPHVMGKLQAEVRRVMPKGQDIVTEEQLGCMPYLKAVIKETLRLHPPAPLLMPHLSISD

CNINGYTIPSGTRVIVNVWALARDSNYWENADEFIPERFIVNTLGDYNGNNFHFLPFGSG

RRICPGINFAIATIEIMLANLVYRFDWELPADQAAKGGIDMTETFGVAVHRKEKLLLIPH

LHLR*

>CYP71D16 Nicotiana tabacum

MQFFNFFSLFLFVSFLFLFKKWKNSNSQTKRLPPGPWKLPILGSMLHMLGGLPHHVLRDL

AKKYGPIMHLQLGEVSLVVISSPGMAKEVLKTHDLAFANRPLLVAAKIFSYNCMDIALSP

YGNYWRQMRKICLLELLSAKNVKSFNSIRQDEVHRMIKFFRSSPGKPVNVTKRISLFTNS

MTCRSAFGQEYKEQDEFVQLVKKVSNLIEGFDVADIFPSLKFLHVLTGMKAKVMNTHNEL

DAILENIINEHKKTSKSDGESGGEGIIGVLLRLMKEGGLQFPITNDNIKAIISDIFGGGT

ETSSTTINWAMVEMMKNPSVFSKAQAEVREILRGKETFGEIDVEEFKYLKMVIKETFRLH

PPLPLLLPRECREEIDLNGYTIPLKTKVVVNAWAMGRDPKYWDDVESFKPERFEHNSMDY

IGNNYEYLPFGSGRRICPGISFGLANVYFPLAQLLNHFDWKLPTGINPRNCDLTEAAGAA

CARKNDLHLIATAYQHCEE

>CYP71D97 Ammi majus

MMKMALQFVPIFMFMIILFMLLNLLKKLFQRSTKKLPPGPFKFPIVGNLLQVTGGLPHRR

LYNLSKTHGPLMHLQLGEVSAVVISNPRVAKEVLKTHDLCFADRPTLLLGNIVLSNCRDI

VLAKYGEHWRQFRKICTLELLSASKVRSFRTIREEEASDLIQSIQSTSGSPVNVSEKVSH

LANSITCRSTIGKRCKYEHELIEATENIAYWGAGFFMADLFPSMLVFPVLSGMKPALKKI

RRDLDHIFDYIINEHKEKLASRKDQGTKLDAEEEDLVDILLRINDTLQLEFPVTSNDIQG

IVQDMFTAGTDTSSAVLEWAMSELMKKPSAMKKAQDELRNALRGKERICEADIQGLTYLK

LVIKETLRLHPPVPLLLPRECRKECEIDGYTIPVGTKVMVNAWAIGRDPDYWVDADSFIP

ERFDGSSVNYNGANFEYIPFGAGRRMCAGITFGIASIELPLAQLLYHFDWTLPNGMKPED

LDMDETFGATTKRKNSLVLNVTSHISSLEE*

>CYP71E1 Sorghum bicolor

MATTATPQLLGGSVPQQWQTCLLVLLPVLLVSYYLLTSRSRNRSRSGKLGGAPRLPPGPA

QLPILGNLHLLGPLPHKNLRELARRYGPVMQLRLGTVPTVVVSSAEAAREVLKVHDVDCC

SRPASPGPKRLSYDLKNVGFAPYGEYWREMRKLFALELLSMRRVKAACYAREQEMDRLVA

DLDRAAASKASIVLNDHVFALTDGIIGTVAFGNIYASKQFAHKERFQHVLDDAMDMMASF

SAEDFFPNAAGRLADRLSGFLARRERIFNELDVFFEKVIDQHMDPARPVPDNGGDLVDVL

INLCKEHDGTLRFTRDHVKAIVLDTFIGAIDTSSVTILWAMSELMRKPQVLRKAQAEVRA

AVGDDKPRVNSEDAAKIPYLKMVVKETLRLHPPATLLVPRETMRDTTICGYDVPANTRVF

VNAWAIGRDPASWPAPDEFNPDRFVGSDVDYYGSHFELIPFGAGRRICPGLTMGETNVTF

TLANLLYCYDWALPGAMKPEDVSMEETGALTFHRKTPLVVVPTKYKNRRAA

>CYP71F1 Triticum aestivum

MEEWLLSLCFIALSTATVLAFWFLKLSGGKADPHKKQLPPGPWTLPVIGSLHHVISALPH

RTMMQLSCRHGPLMLLRLGEVPAVVVSTADAAALVMKTHDLVFVDRPRSPTMDIASSGGK

DIVFAPYGGHWRQMRKICVVQLLSSTQVSRMEGVRAEEVGSLLRDITAAASTGATINVSE

KVMALTNDIVTRAVFGGKFARQCEFLREMDKAFKLVGGFCLADLFPSSRLVRWLSNGERD

MKRCHGLIHHIIAEVVENRKAARASGVGRSIPGDEDMLDVLLTLQEDDSLEFPLTTETMG

AVLHDVFAGATETTGNTLAWVISELMHNPHTMAKAQHEVRDVLGEGRSVITNSDLGELHY

MPMILKEALRLHPPGPLIPRMAREDCTVMGYDIPKGTNVYINIFAISRDPRYWINPEEFM

PERFENNNVNYKGTYFEFIPFGAGRRQCPGIQFSSSITEMALANLLYHFDWMLPDGANLA

SFDMSEKFGFAVSKKYDLKLRAIPHVWSNAMTLK

>CYP71G1v1 Asparagus

MTVSITAAVQLFLLLLLLLPLLFVHHKTKPKTKCRSPPGPPPLPVIGNLHQLSLLLHQSL

YRLSKIHGPIFKLSLGRVPVLVISSPSLAKQVLKTHDLACCSRASTVSFKEYTYDGCDVA

GAPYGDSWRNLRKIFVLKLLSSKKLTSFRLVQEEEIEGMISSIRTRSDTNATVNITEFVV

RLANNITFRVAFGYRSEGEYGEKSRFQRLLESGNDTVASFYVGDYFPGLGWLDKMTGKLG

KMKRNARDLDEFYQEVIDAHMKDGRKEDGKEDIVDVLLRLREEGQLTMDHIKGALMNIFV

GGTDTSAASIAWAMAELARKPKVMKKAQEEVRKAASKKGKVEENDLAQLQYIKCVVNETL

RLHLPLPLLVPRETIQHCEINGYDVSAKTRVLVNAWAIGRDEDAWENPEEFNPDRFVGSS

LDYKGQDFQFIPFGAGRRICPGIQFGVETVELALANLLYAFNWELPPGVERENIDMHEAP

GLVTRRATDLRLVATNYEEAN

>CYP71J1 Asparagus officinalis

MPLILVILLLLPILLLVIRREKSTSSKLPPCPPKLPLIGNLHQLGSLPHQSLHALSVKYG

PLMLLKLGEIPTLIVSSSDMAREIMRTHDHIFASRPSLLTSDILLNGATDVVFAPYGEHW

RQMRKLCVNHLLSAKMVQSFRLMREEEVSSMLTRISGLVNMSEVLNLFTSKILFNAISGK

FFVEEEGRINVFCKLIRENIAILAQLSVSDFFPSLGWLDLVFGVGARARATAKKWDDVLD

EVIEDHVKRSNETGDADDQEERADFVSVLMALQEDDNTGFTLNRNIIKAILQDMIAAGTE

TSFLVLDWGMTELVRNPGTMKKLKDEVRSVAGSETVVREEDISKMFYLKAVIKEILRLHP

PVPLLIPRESMDHCNVQQYEVPSKTRVLINAWSMGRDPKVWEDPEEFRPERFLDSDIDFR

GQCFEFVPFGAGRRICPGMHFAAANLELALANLMYRFDWELPDGMKSEDLDMGDSPGLTT

RRRQNLHLVARPFQRVKR

>CYP71K1 Oryza sativa (rice)

MAELPLYLLLLALLVAVPFLCLTRWSLRHGGGGGGRLPPSPWALPVIGHLHHVAGALPHR

AMRDLARRHGPLMLLRLCELRVVVACTAEAAREVTKTHDLAFATRPITPTGKVLMADSVG

VVFAPYGDGWRTLRRICTLELLSARRVRSFRAVREEEVGRLLRAVAAAAAVAALTTPGAT

AAVNLSERISAYVADSAVRAVIGSRFKNRAAFLRMLERRMKLLPAQCLPDLFPSSRAAML

VSRMPRRMKRERQEMMDFIDDIFQEHHESRAAAGAEEDLLDVLLRIQSQDKTNPALTNDN

IKTVIIDMFVASSETAATSLQWTMSELMRNPRVMRKAQDEVRRALAIAGQDGVTEESLRD

LPYLHLVIKESLRLHPPVTMLLPRECRETCRVMGFDVPEGVMVLVNAWAIGRDPAHWDSP

EEFAPERFEGVGAADFKGTDFEYIPFGAGRRMCPGMAFGLANMELALAALLYHFDWELPG

GMLPGELDMTEALGLTTRRCSDLLLVPALRVPLRDHER

>CYP71L1 Hordeum vulgare (barley)

MAAASLVLELLRQQWQVTVAILLLPLASFLLTRRRSSNLNGCNESGGLRLPPCPWRLPMV

GNLHQIGSLPHRDLARLARRHGPVMMVRLGMVPAVVLSTAAAAEEAFKTNDKDCSSRPLT

VGPGKLTYGYKDVVFAPWSDYVREMRKLFIIEMLSARRVKAAYFARETQIERMVAKLEAV

GPNPIRIDEHIFTTVDAIVSLFVFGELNAGEQFKGELVDLLNETTDLLTSFTAEDYFPNA

AGRLIDRITGMHGRRETLFRKLDSMMEYLLAMYEDPGHKRKADADGSDLVQEVVDLMKRP

PAKGMITFTRDHAKSILFDTFMAATDTSSLSSYWVMTELIRHPRVLHKAQAEVRAAAGGA

PQVRISDMPKLKYLRMVLSETFRMHPPATMLVPRETMRPIRLGGYDIPANTMLMVNAWAI

GRDPASWKDPEVFYPERFEELDVDFNGGHYELLPFGAGRRICPGLAMGVANTEFILANLL

YCFNWALPQGMRSEDVGVEEFGGLTFRKKKPLVLVPTRYYPDKEEK*

>CYP71M1v1 Hordeum vulgare (barley)

MAPDLNPSPELKHVLLWSVPLLIIAPTVIFLYMQAVGKKKKNTIRLPPSPLRLPIIGHLH

LMVHEPHRSLQRLARSLGPVVHLQLGGVAAIVVSSPEAAKEVLKTHDVHCCSRPSSPGAK

LITYGNQDIAFSPYNASWRERRKLFVSELVSSKRVQSFAYALQAQVGELIQSLSLRSPPR

EPVNLNETLFTLIDGFIGTVAFGSMKGAKLMKYAKFQQVFSEAMVALSAFSAQDFFPASR

MSRWFDKLVGLEARYQRIFLELDSYFEMVLSQHMDPGRVKTDKDDLVDVLISLWKGQGKV

TKDHLKALLMDAFIGGTTTSSVTLLWAMSELIKNPTVMKKAQTEIRSLVGDKRRLVQVDD

LSKLNYLKMIVKETLRLHPPAPLLVPRETMDHVKVLGYDIPTKTRIFVNVWAMGRDPACW

DKPEEFYPERFDGVDTDFYGSHYELLPFGAGRRICPAIPMGATIVEFTLASLLHSFDWEL

PDGMTKEDVSMEGTGRQIFCRKTPLYLVPSFYTG*

>CYP71N1 Musa acuminata (banana)

MALPPLLLSPLPSLLVVLALLSSLLLAGRKARGGSATWKLPPGPPKLPVIGHLHLLGSSL

LHRSLWELSKKHGPLMHLKFGRVPVVVVSSPEMAKEVLKTHDLECCSRPSLLSFSKFSYG

LSDVAFIPYGERWRQLRKLCTVELLSTRKINSFRDIRKEEMERVTKLICSHVRASSMVNL

SELLLSLSCNMTCRSAFGSGFDDGGDIQLHDMLREAQEELSGLFLSDYLPLLGWVDRLSG

MRSRLERAFLKLDSIYQRRIDYHQDRLRQQGKEDGDVLDALLRMQKDEEGLTEDHIKGVL

MDIFIAGTDTSSATVEWAMAELIRQPELMKRAQDEVRRCVGSKGEVEESDLHQLHFFKCV

IKETMRLHPPAPLLLPRETMQHFKLNGYDILPKTWMYVNAWAIGRDPNSWGRPHVFDPER

FMHDSTEASGQDFKLIPFGEGRRICPGKNLGMLMVELVLANLLYSFDWHLPPGMVKEDIS

MEEAPGVTVHREYALCLMATKYDATTA

>CYP71P1 Oryza sativa (rice)

MSLALLVLSAAYVLVALRRSRSSSLKPRRLPPSPPGWPVIGHLHLMSGMPHHALAELART

MRAPLFRMRLGSVPAVVISKPDLARAALTTNDAALASRPHLLSGQFLSFGCSDVTFAPAG

PYHRMARRVVVSELLSARRVATYGAVRVKELRRLLAHLTKNTSPAKPVDLSECFLNLAND

VLCRVAFGRRFPHGEGDKLGAVLAEAQDLFAGFTIGDFFPELEPVASTVTGLRRRLKKCL

ADLREACDVIVDEHISGNRQRIPGDRDEDFVDVLLRVQKSPDLEVPLTDDNLKALVLDMF

VAGTDTTFATLEWVMTELVRHPRILKKAQEEVRRVVGDSGRVEESHLGELHYMRAIIKET

FRLHPAVPLLVPRESVAPCTLGGYDIPARTRVFINTFAMGRDPEIWDNPLEYSPERFESA

GGGGEIDLKDPDYKLLPFGGGRRGCPGYTFALATVQVSLASLLYHFEWALPAGVRAEDVN

LDETFGLATRKKEPLFVAVRKSDAYEFKGEELSEV*

>CYP71Q1 Oryza sativa (rice)

PPLLQLSAAVLFFLLPLLYLLFLRGSNGEVRGRQGNSASAPSLPGPCRQLPVLGNLLQIG

SRPHRYFQAVSRRYGPVVQVQLGGVRTVVVHSPEAAEDVLRTNDVHCCSRPPSPGSYNYL

DVAFAPYSDYWREMRKLFVVELTSVSRVRSFAYARAAEVARLVDTLAASPPGVPVDLSCA

LYQLLDGIIGTVAFGKGYGAAQWSTERAVFQDVLSELLLVLGSFSFEDFFPSSALARWAD

ALAGVERRRRRIFRQVDGFLDSVIDKHLEPERLSAGVQEDMVDALVKMWREQQDRPSGVL

TREHIKAILMNTFAGGIDTTAITAIWIMSEIMRNPRVMQKARAEVRNTVKNKPLVDEEDS

QNLKYLEMIIKENFRLHPPGNLLVPRQTMQPCLIGGYNVPSGTRVFINIWAMGRGPMIWD

NPEEFYPERFEDRNMDFRGSNFELVPFGSGRRICPGVAMAVTSLELVVANLLYCFDWKLP

KGMKEEDIDMEEIGQISFISFRRKVELFIVPVKHEQYQLMGHIN*

>CYP71R4Loliumrigidum

MASFELDSTLVLLCLVFVVSCFAVVVRGSGTGRKYGVRAVPPGPLALPIIGNLHKLGGAH

PHRSLQGLARRHGPLFLLHLGSVPTVVVSSASLAEALLRTQDHVFCSRPQPYTARGTLYG

CRDIAFSPYGEKWRQIRRIAVVHLLSMKRVDSFRALRVEEVARFVQRIGAASGRERVDVS

ELIIDLTNTVISKAAFGNKLGGMEPATVRDMMKELTVLLGTIAVSDVFPRLGWLDWAMGL

DARVKRTAARLDTVVERTLAEHEGNRVKDGEACDLLDDLLSIYKDGDQGFKLDRTDVKAL

ILDMFIAGTDTIYKTIEWTMAELVRNPREMAKVQYEVRLHAAASAQGVVLEEELEKMSLL

HAAIKEALRLHPPVPLLIPRESIEDTRLHGYDILAKTRVMVNTWAIGRDSESWENAEEFL

PERFIGQAMEYNGKDTRFIPFGAGRRGCPGIAFGTRLVELTLANMMYHFDWKLPNGQDIE

SFELIESSGLSPGLKSALILAVKPL

>CYP71S1 1016223.1 Oryza sativa (rice)

PRPRGLPLIGNLHQVGALPHRSLAALAARHATPLMLLHLGSVPTLVVSTADAARALFRDN

DRALSGRPALYAATRLSYGQKNISFAPDGAYWRAARRACMSALLGAPRVRELRDAREREA

AALIAAVAAAGASPVNLSDMVAATSSRIVRRVALGDGDGDESMDVKAVLDETQALLGGLW

VADYVPWLRWVDTLSGMRRRLELRFHQLDALYERVIDDHLNNRKHASDEEDDLVDVLLRL

HGDPAHRSTFGSRSHIKGILDMFIAGSDTSAVTVQWAMTELVRNPDVLAKAQHEVRRVVA

AGDKVREADLPELHYLRLVIKETLRLHPAAPLLVPREMTEPFRTAHGVEIPARTRVVVNA

MAIHTDPGVWGPDAERFVPERHRDDADGCAQQHDGFALVPFGIGRRRCPGVHFAAAAVEL

LLANLLFCFDWRAPPGREVDVEEENGLVVHKKNPLVLI

>CYP71T1 Oryza sativa (rice)

MELSSSLAAVLHSPLFLLAALLLLPVFTLLSFSSAKKPGDGGGRRLPLPPSPRGVPFLGH

LPLLGSLPHRKLRSMAEAHGPVMLLWFGRVPTVVASSAAAAQEAMRARDAAFASRARVSM

AERLIYGRDMVFAPYGEFWRQARRVSVLHLLSPRRIASFRGVREQEVAALLDRVRRRCGV

RGGGETVNLSDMLMSYANGVISRAAFGDGAYGLDGDEGGGKLRELFANFEALLGTATVGE

FVPWLAWVDKLMGLDAKAARISAELDGLLERVIADHRERRRLSQPDGGDGDGDGDENVDH

RDFVDVLLDVSEVEEGAGAGEVLLFDAVAIKAIILDMIAAATDTTFTTLEWAMAELINHP

PVMRKLQCEIRAAVGVPGASGGAEVTEDHLGELRLLRAVVKETLRLHAPVPLLVPRETVE

DTELLGYRVPARTRVIINVWAIGRPXAAWGDRAEEFVPERWLDGGGGGEAVEYAAQLGQD

FRFVPFGAGRRGCPGAGFAAPSIELALTNLLYHFDWELPPHADGAAAATAARLDMGELFG

LSMRMKTTLNLVAKPWSSDV

>CYP71T3 Oryza sativa (rice)

MAVSLVVVVVVVIAIVVPLLYLVLLPAWKPARRDDGDGGMRRRLPPSPPWGLPLLGHLHL

LGALPHRALRSLAAAHGPVLLLRLGRVPVVVVSSAAAAEEVMRTRDLEFASRPRVAMAER

LLYGGRDVAFAPYGEYWRQTRRICVVHLLSARRVLSFRRVREEEAAALVARVRAAGGAVD

LVEHLTAYSNTVVSRAVFGDESARGLYGDVDRGRVLRKLFDDFVELLGQEPMGELLPWLG

WVDALNGMEVKVQRTFEALDGILEKVIDDHRRRRREVGRQMDDGGGGDHRDFVDVLLDVN

ETDMDAGVQLGTIEIKAIILDMFAAGTDTTTTVIEWAMAELITHPDAMRNAQDEIKAVVG

ITSHITEDHLDRLPYLKAVLKETLRLHPPLPLLVPHEPSSDTKILGYSIPACTRIVINAW

TIGRDQATWGEHAEEFIPERFLESGLDYIGQDFVLVPFGAGRRGCPGVGFAVQAMEMALA

SLLYNFDWETRVVDRRSEFGTSSLDMSEMNGLSVRLKYGLPLIAISRFP*

>CYP71U2 Oryza sativa (rice)

MDELSIENHSPISMDELSFGSLCLVAMATLALALALMVVMGAHRRGGEKGATTGAKNLPP

GPWNLPVTGSLHHLLGASPPPHRALLRLSRRHGPLMLVRLGEVPTVIVSGSDAAMEGWVL

KAHDPAFADRARSTTVDAVSFGGKGIIFAPYGEHWRQARRVCLAELLSARQVRRLESIRQ

EEVSRLVGSIAGSSNAAAVDMTRALAALTNDVIARAVFGGKCARQEEYLRELGVLTALVA

GFSMADLFPSSRVVRWLSRRTERRLRRSHAQMARIVGSIIEERKEKKASDDGVGAKDEDD

DLLGVLLRLQEEDSLTSPLTAEVIGALVIDIFGAATDTTASTLEWVMVELMRNPRAMEKA

QQEVRNTLGHEKGKLIGTDISELHYLRMVIKETLRLHPSSALILRQSQGNCRVMGYDIPQ

ATPVLINTFAVARDAKYWDNAEEFKPERFENSGADIRTSTAHLGFVPFGAGCRQCPGALF

ATTTLELILANLLYHFDWALPDGVSPESLDMSEVMGITLHRSSSLHLHATLSRLGFVSHS

GQ*

>CYP71V1 Oryza sativa (rice)

MDDYFFLQSLLLCVAAVALLQLAKVAATMRRRPRTPPGPWRLPVIGSMHHLVNALPHRAM

RDLAGVHGPLMMLRLGETPVVVASSRGAARAVLKTHDANFATRPRLLAGEIVGYGWADIL

FSPSGDYWRKLRQLCAAEILSPKRVLSFRHIREDEVTARVEEIRAAAAPSTPVNLSVLFH

STTNDIVARAAFGRKRKSAPEFMAAIKAGVGLSSGFKIPDLFPTWTTALAAVTGMKRSLR

GIHKTVDAILQEIIDERRCVRGDKINNGGAADDQNADENLVDVLIALQEKGGFGKSVTTP

WVIVTHMICTLDVQDMFAGGTGTSASALEWAMSELMRNPAVMKKLQGQIREAFHRKAVVT

EADLQASNLRYLKLVIKEALRLHPPAPLLVPRESIDTCELDGYTIPAKSRVIVNVWAIGR

DPKDAEEFKPEQFDDDAIDFMGGSYEFIPFGSGRRMCPGFNYGLASMELVLVAMLYHFDW

SLLVGVKEVDMEEAPGLGVRRRSPLLLCATPFVPAAVSADY*

>CYP71W1 Oryza sativa (rice)

MELTTLLLLALISFFFLVKLIARYASPSGRESALRLPPGPSQLPLIGSLHHLLLSRYGDL

PHRAMRELSLTYGPLMLLRLGAVPTLVVSSAEAAAEVMRAHDAAFAGRHLSATIDILSCG

GKDIIFGPYTERWRELRKVCALELFNHRRVLSFRPVREDEVGRLLRSVSAASAEGGAACF

NLSERICRMTNDSVVRAAFGARCDHRDEFLHELDKAVRLTGGINLADLYPSSRLVRRLSA

ATRDMARCQRNIYRIAESIIRDRDGAPPPERDEEDLLSVLLRLQRSGGLKFALTTEIIST

VIFDIFSAGSETSSTTLDWTMSELMKNPRILRKAQSEVRETFKGQDKLTEDDVAKLSYLQ

LVIKETLRLHPPAPLLIPRECRETCQVMGYDVPKGTKVFVNVWKIGREGEYWGDGEIFRP

ERFENSTLDFRGADFEFIPFGAGRRMCPGIALGLANMELALASLLYHFDWELPDGIKSEE

LDMTEVFGITVRRKSKLWLHAIPRVPYYSTY*

>CYP71X2 Oryza sativa (rice)

MYDAVACVVAVVVVVVFAMLWVKLARSGDGGGGGSGGVRLPPGPWRLPVIGSLHHVVGDR

LLHRSMARIARRLGDAPLVYLQLGEVPVVVASSPGAAREVTRTHDLAFADRALNPTARRL

RPGGAGVALAPYGALWRQLRKICVVELLSARRVRSFRRVREEEAGRLVGALAAAAASPGE

EAAVNFTERIAEAVSDAALRAMIGDRFERRDEFLQELTEQMKLLGGFSLDDLFPSSWLAS

AIGGRARRAEANSRKLYELMDCAIRQHQQQRAEAAVVDGGAGVEDDKNQDLIDVLLNIQK

QGELETPLTMEQIKAVILDLFSGGSETSATTLQWAMSELIKNPMVMQKTQAELRDKLRRK

PTVTEDDLSGLKYVKLIIKETLRLHPVVPLLVARECRESCKVMGYDVPKGTTVFVNAWAI

GRDPKYWDDAEEFRPERFEHSTVDFKGIDLEFIPFGAGRRICPGMAFAEAIMELLLAALL

YHFDWELPNGMAASELDMTEEMGITVRRKNDLHLRPHPPCVVRSNFRSFVERERERHFV*

>CYP71Y1 Oryza sativa (rice)

MEDATHGYVYVGLALVSLFVVLLARRRRSPPPAAHGDGGLRLPPGPWTLPIIGSLHHLVG

QIPHRAMRDLARRHGPVMLLRIGEVPTLVVSSRDAAREVTKTHDTAFAMRPLSATLRVLT

NGGRDLVFAPYGDYWRQVRKIAVTELLTARRVHSFRSIREEEVAALLRAVAVAAGTVEMR

AALSALVSDITARTVFDNRCKDRGEFLVLLERTIEFAGGFNPADLWPSSRLAGRLSSVVR

RAEECRNSVYKILDGIIQEHQERTSAGGEDLVDVLLRIQKEGGLQFPLAMDDIKSIIFDI

FSAGSETSATTLAWAMAELIRNPTAMHKVMAEVRRAFAAAGAVSEDALGELRYLQLVIRE

TLRLHPPLPLLLPRECREPCRVLGYDVTRGTQVLVNAWAIGLDERYWPGGSPEEFRPERF

EDGEATAAVDFRGTDFEFLPFGAGRRMCPGMAFGLANVELPLASLLFHFDWEVPGLADPA

KLDMTEAFGITARRKADLHLRPCLLVSVPGV*

>CYP71Z1 Oryza sativa (rice)

MGASILLVVVVSKLMISFAAKPRLNLPPGPWTLPLIGSIHHVVSSRESVHSAMRRLARRH

GAPLMQLWFGEVGTVVASSPEAAREVLRSHDLAFADRHLTAAAAAFSFGGRDVVLSPYGE

RWRQLRKLLTQELLTASRVRSFRRVREEEVARLMRDLSAAATAGAAVNLSEMVTRMVNDT

VLRCSVGSRCEHSGEYLAALHAVVRLTSGLSVADLFPSSRLAAMVSAAPRAAIANRDKMV

RIIEQIIRERKAQIEADDRAADSKSCACSLDDLLRLQKEGGSPIPITNEVIVVLLMDMFA

GGTDTSSTTLIWAMAELIRSPRVMAKVQSEMRQIFDGKNTITEDDLVQLSYLKMVIKETL

RLHCPLPLLAPRKCRETCKIMGYDVPKGTSAFVNVWAICRDSKYWEDAEEFKPERFENND

IEFKGSNFEFLPFGSGRRVCPGINLGLANMEFALANLLYHFDWKLPNGMLHKDLDMREAP

GLLVYKHTSLNVCPVTHIASSCA*

>CYP71AA2 Oryza sativa (rice)

MAGIMDSTTASYYTTLLCGALLLAAVVFKLKTAAAFSRHNAGVNLPPGPWALPVIGSIHC

LLGSLPHHAMRELSRRYGPVMLLRLGHVRTLVLSSPEAAREVMKTHDVAFANRAVTPTAS

ILTYGARDIVFAPFGKHLRELRKLCALELLSPRRVRSFRHVREEEAARLARSVAAAASAS

SAVNVSELVKIMTNDVTMRAIIGDRCPQREEYLEALDKTMDLLAGFNLVDLFPGSPLARV

LGGRSLRTTKRVHEKLHQITEAIIQGHGIKDTVGDEHHECEDILDVLLRFQRDGGLGITL

TKEIVSAVLFDLFAGGSETTSTTILWAMSELMRSPHVMEQAKYEIRQVLQGKAMVSEADI

EGRLHYLQLVIKETLRLHPPVPIVIPRLCSKPNSKIMGYDIPQGTSVLVNVSAIGRDEKI

WKNVNEFRPERFKDDIVDFSGTDFRFIPGGSGRRMCPGLTFGVSNIEIALVTLLYHFDWK

LPSETDTHELDMRETYGLTTRRRSELLLKATPSY

>CYP71AB1 Oryza sativa (rice)

MANLIYYSLLIILPFLFLIKFYKAMFSSRKQARRLPPCPWQLPIMGSIHHLIGDLPHRAL

RDLSRRYGPVMLLKFGQVPFIIVSSPEAAKDIMKTHDSIFATRPQSEIMKIITKRGQGLV

FAPYDDQWRQLRKICIRELLCAKRVQSFCAIREEEAARLVKSISSDQAHLVNLSKKLADY

ATDAAIRIITGTRFENQEVRDKFQYYQDEGVHLAASFCPANLCPSLQLGNTLSRTAHKAE

IYREGMFAFIGGIIDEHQERRAQDMSHKEDLIDVLLRIQQEGSLESPVSMETIKFLIFDI

LAGGSETVTTVLQWAMAELMRNPTVMSKVQDEVREVFKWKEMVSNDDINKLTYLQFVIKE

TLRLHTPGPLFMRECQEQCQVMGYDMPKGTKFLLNLWSISRDPKYWDDPETFKPERFEDD

ARDFKGNDFEFISFGAGRRMCPGMLFGLANIELALANLLFYFDWSLPDGVLPSELDMTEN

FGVTVRKKEDLLLHASLYAQLSC*

>CYP71AC1 Oryza sativa (rice)

MDLMKSNPLQGSPWSLLNLLVLIIVAAMICGELCRRRRRRRGDENGGATRLPPGPWRLPF

VGSLHHLAVMRPRGVVVHRALAELARRHDAPVMYLRLGELPVVVASSPEAAREVLKTHDA

AFATRAMSVTVRESIGDKVGILFSPYGKKWRQLRGICTLELLSVKRVRSFRPIREEQVAR

LVDAIAAAAASSTAEAAAVNISRQITGPMTDLALRAIMGECFRWREEFLETLAEALKKTT

GLGVADMFPSSRLLRAVGSTVRDVKLLNAKLFELVECAIEQHREQIRAAHDNGGDDDDAH

GHGDKECFLNTLMRIQKEGDDLDDLTMATVKAVILDMFAGGSESTSTTLEWALSELVRNP

HVMQKAQAEIRHALQGRTRVTEDDLINLKYPKNIIKETLRLHPVAPLLVPKECQESCKIL

GYDVPKGTIMFVNAWAIGRDPRYWNDAEVFMPERFEKVAVDFRGTNFEFKPFGAGRRMCP

GITFANATIEMALTALLYHFDWHLPPGVTPDGLDMEEEFGMSVSRKRDLYLRPTLHMGLE

TI*

>CYP71AD1 Oryza sativa (rice)

MEIELSPVLLLLPFLLLGFLYLTGGVLRSGGNARRRLAPAPRGLPVIGNLHQVGALPHRA

LRALAAATGAPHLLRLRLGHVTALVASSPAAAAAVMREHDHVFATRPYFRTAEILTYGFK

DLVFAPYGEHWRHARRLCSEHVLSAARSHRYGPMREQEVALLVNAIRTEAAAAAVDVSKA

LYAFTNAVICRAVSGRLSREDEGRSELFRELIEENATLLGGFCVGDYFPALAWADAFLSG

FAARACRNLRRWDELLEEVIAEHEARLRGGDDGGGEEHREEDFVDVLLALQEESQRHDGS

FKLTRDIIKSLLQDMFAAGTDTSFITLEWAMSELVKNPAAMRKLQDEVRRGGGATTAATP

YLKAVVKETLRLHPPVPLLVPRECARDTDDDATVLGYHVAGGTRVFVNAWAIHRDAGAWS

SPEEFRPERFLPGGGEAEAMDLRGGHFQLVPFGAGRRVCPGMQFALATVELALASLVRLF

DWEIPPPGELDMSDDPGFTVRRRIPLRLVAKPVGSEDDK*

>CYP71AE1 Oryza sativa (rice)

MASLATVPNLPLLLLLHYALATFTASRARKNNKDRLPPSPLALLVIGHLLHLMGSLPRTS

PSAASPHGTGPTCSSGLAPCRCSLRRRRVPAAEAILRTHDHVFASRPRTVLLANIVFYRS

RDVRFAPYGDHWRQARKLVTTHLLSAKKVRSLRLAREEEVSLVMTKISKAATASAVVDIG

QILRSFTNDMICRTVSGKCPRDDR*KRIFQELANETSLLLGGFDIEEYFPVLARVGLVGK

MMCLKAERLKKRWDELLEELINDHENDDHSCNLISDQNDEDFVDILLSVRQEYGFTREHV

KAILDVFFGGIDTSALVLEFTIAELMQRPRMLKKLQDEVRACIPKGQKIVSEVDINNMAY

LRAVIKEGIRLHPVAPVLAPHISMDDCNIDGYMIPSGTRVLVNVWAIGRDPRFWEDAEEF

VPERFIDSMSSAAANVNFTENDYQYLPFGYGRRMXPGMKFGIAVVEIMLANLMWKFDWTL

PPGTEIDMSEVFGLSVHRKEKLLLVPNNMSSC*

>CYP71AF1 Oryza sativa (rice)

MEQYLFLATLLILSLAFVKLRPRNNGENPPPGPWQLPVIGSLHHLAGALPHRALRDLATR

HGELMLLRLGELPVVVASSPAAAREVMRTHDAAFATRPQTATLRALTRDGLGVAFAPQGE

HWRCLRKLCVTELLGARRVRCLRRAREAEAAALVASLSTTTPEPVNVSSLVARYVTDAVV

RAVVGDRISDRDAFLERLEEGVKVAAGFTLADVFPSSRLARALSGTARRAEAHSREMTRL

MDGVIEEHRQRRAATGWRDEEDEDLLDVLLRIQKDGGLQIPLDMGTIRAIIIDLFSAGSE

TTGTTLQWAMAELMRNPAALRKAQAEVRGVLAGHSHVTEDALPDLHYLHLVIKETLRLHV

AVPLLLPRECQEPRLRVLGYDVPERAMVLVNAWAICRDTAVWGPDAEEFRPERFDGGAVD

FKGTDFEFVPFGAGRRMCPGVAFAVAIMELGLASLLFHFDWELAGGTAAGELDMAEGLGI

TARRKSDLWLHATVSVPVPNTETS*

>CYP71AG1 Sorghum bicolor ortholog

MTLSAPLSYNTIILVFVVFIISYVSLLVGGGGKKSVANAAANRLPPPSPRGLPVIGNLHQ

LGSLPHRSLRSLAAAHGPVMLIRLGQVPAVVVSSASAAREVLQAQDHVFAGRPSLTIPRR

LLYGCTDIAFAPHGAYWRGARKMSVRHLLSPPRVRAYRAVREQEVDALVRRVLEQACGAG

GGVVRLSELLNDFAKDVAGRIVLGLRAAGDDGWRGKVDALLEESNVLLGAFHVGDYIPWL

SWVSHVDGTDARVTRAFEKMDRILEEMVDAAATRGREMPLSDSGEEASGGDDAFIHVLLS

LQQQQRQQQEEEPTAEWRLSRDNVKALLEDLFGAGTEATIIVLEWAMAELLRNKGVMEKL

QREVRQAQARARRSSSSDIIVGEQDLAGTGMEYLRAVIKETMRLHTPGPLLLPHKSMEAT

RISHGHGYDVPSDTMVIVNAWAIGRDPEAWESPADEFRPERFVGSGVDFRGHHFQLIPFG

AGRRMCPGINLAMSVVELALANLVARFDWALPGAELELDMEETTGCTARKKAPLCAVATL

LP

>CYP71AH1 tobacco

MKFLLVVASLFLFVFLILSATKRKSKAKKLPPGPRKLPVIGNLLQIGKLPHRSLQKLSNE

YGDFIFLQLGSVPTVVVFSAGIAREIFRTQDLVFSGRPALYAGKRFSYNCCNVSFAPYGN

YWREARKILVLELLSTKRVQSFEAIRDEEVSSLVQIICSSLSSPVNISTLALSLANNVVC

RVAFGKGSDEGGNDYGERKFHEILFETQELLGEFNVADYFPGMAWINKINGLDERLEKNF

RELDKFYDKIIEDHLNSSSWMKQRDDEDVIDVLLRIQKDPNQEIPLKDDHIKGLLADIFI

AGTDTSSTTIEWAMSELIKNPRVLRKAQEEVREVAKGKQKVQESDLCKLEYLKLVIKETL

RLHPPAPLLVPRVTTASCKIMEYEIPADTRVLINSTAIGTDPKYWENPLTFLPERFLDKE

IDYRGKNFELLPFGAGRRGCPGINFSIPLVELALANLLFHYNWSLPEGMLPKDVDMEEAL

GITMHKKSPLCLVASHYNLL

>CYP71AJ1 Ammi majus

MKMLEQNPQYLYFFSLFLVTIFLYKWLTLKKTPLKNLPPSPPQYPIIGNLHQIGPDPQAS

LRDLAQKYGPLMFLKFGTVPVLVVSSADAAREALKTHDLVFADRPYSSVANKIFYNGKDM

VFARYTEYWRQVKSICVTQLLSNKRVNSFHYVREEEVDLLVQNLENSHSKVANLTELLIE

VTGNVVCRVSVGSGDKVDSYKILILEIMDMLGYSRSIEDFFPLLGWVDWLTGLRGKVAEA

AKGVDTFLEGVLKEHLSTTGSKYNDFVSILLEIQEADAGSSMDNECIKSLIWDMLGAGTE

TISTALEWTLAALIKNPDAMFKLQNEVREIGKGKSKISEADLVKMNYLQAVMKESMRLYF

TAPLLVPREARQDIKFMGYDISSGTQVLINAWAIARDPLLWDKPEEFRPERFLNSPIDYK

GFHYEFLPFGAGRRGCPGIQFAMCINELVVANLVHKFNFELPDGKRLEDLDMTAASGITL

RKKSPLLVVARPHV

>CYP71AK1 Oryza sativa (rice)

MSSYVVVAAALLVFVVVVVAAIKNLGKGKLPPSPPSLPFVGHLHLVGELPHRSLDALHRR

YGSDGGLMFLRLGRAGALVVSTAAAAADLYRGHDLAFASRPPSHSAERLFYGGRNMSFAP

LGDAWRRTKKLAVAHLLSPRRARRAQRGAGAVQPRELRTPNKKGVITRVAAGGSGATAER

FRKMMADTSELLAGFQWVDRLPEAAGWAARKLTGLNKKLDDMADESDRFLGEILAAHDDE

KAEGEEEDFVDVLLRLRRQGAAAAGGLELAEDNVKAIIKDIMGAATDTSFVTLEWIMTEL

IRNTQVMSKLQNEIIQVTGSKPTVTEEDLTKLDYLKAVIKEVLRLHPPAPLLIPHHSTMP

TTIQGYHIPAKTIAFINVWAIGRDPAAWDTPDEFRPERFMGSAVDFRGNDYKFIPFGAGR

RLCPGIILALPGLEMVIASLLYHFDWELPDGMDVQDLDMAEAPGLTTPPMNPVWLIPRCR

TI*

>CYP71AL1 Centaurium erythraea (Common Centaury)

MEITDFSTFLLAFLLLSYLLVTGRRLISKKSTGKLPPGPKKFPIVGNLPQLALAGTLPHR

AMRDLAKTYGPLMHLRLGEVSQLVVSSPEMAKEVLKTLDPMFASRPDLILADIMLYDNAG

LTFAKYGDYWRQLKKIFATELLSAKRVKSFRSLREEETLNTIRWISSNEGKPINMTNTLL

NLVFGVLSRATFGKKSPEQDKLVYIVNKAAELATGGNISDLFPSIKFFRLISVVNYKLKS

MFAESNRLLDMIMKEHKKGNGSGESKDLVDVLLGYQRENAEFSLTDENIKAVLLDIFIGG

TDGSFTTLDWAMSELMRAPTVLKRAQEEVRQAFETDGYIDEEKFEDLKYVTSIIKETLRL

HPPAPLLVPRSNDETAHILGYEVPAKSKILVNVWAINRDPRYWEDAESFKPERFLGSSVG

YKGTDFHFLTFGAGRRMCPGMVYGYANIVHPLVKLLYYFDWNLPSGIKPEELDMTEEHGL

SVKRKADLYLIPSVRNSISHL

>CYP71AM1 Sorghum bicolor

MDEYFVDLPYPNLCLYGSCLVLAVVVARAIILSGSGKKPGGLPPGPWQLPVIGSLHHLLR

GLPHHAIRDLSLRHGPLMLLRICERTAIVVSSAEAVAEMLKRHDAAFSERPSSPGIEELS

RHGQGVIFAPYGDHWRLLRRILMTELLSPRRVEAFRHIREDEAARLVSSLSSLPQPVDMD

ERLEVFVADSSVRAILGDRLPDRAAFLKMVKAGQDPSSLFDLRDLFPSSWLVRMLPRSRK

AERHLQEMFRLMDDILVSHSQRRVDDDSPDGGGGGAVDEEHDMVDVLLRIQKQGDMRVSL

NHGVIRAALIDAVGAALDTTSTTLRWAMAELIANPRVMHKAQLEIRRVMAAGQQRRVHEA

TLRDLHYLKAVIKETLRLHPPAPFVPRVCLDDGIKIQGYHVPRGTIVVANVWAISRDPKY

WEDPDMFIPERFHQGDPDHHRCFDFKGFDFEFTPFGAGRRMCPGMNFAHMNVEIALASLL

YHFDWKLPDGATPEEIDMTELWGVTVARKAKLLLHPIPCIPAAASIDA.

>CYP71AN1 cottonwood

MT*LLYFQQTWQEIRPKIGLNYLVFFLIFLSFILFLFKLTRSRKLNLPPSPPKLPVIGNI

HHLGTLPHRSLQALSEKYGPLMLLHMGHVPTLIVSSAEAASEIMKTHDIVFANRPQTTAA

SIFFHGCVDVGFAPFGEYWRKVRKISVQELLGPKTVQSFHHVREEEAAGLIDKIRFACHS

GTSVNISEMLISVSSDIVSRCVLGRKADKEGGNSKFGELTRTFMVQLTAFSFGDLFPYLG

WMDTLTGLIPRLKATSRALDSFLDQVIEEHRSLESDGDRCAQTDFLQALLQLQKNGKLDV

QLTRDNIIAVVLDMFVGGTDTSSTMMEWAIAELVRNQTIMRKAQEEVRRIVGKKSKVEAN

DIEEMGYLKCIIKETLRLHPAAPLLVPRETSASFELGGYYIPPKTRVLVNAFAIQRDPSF

WDRPDEFLPERFENNPVDFKGQDFQFIPFGSGRRGCPGALFGVTAVEFMIANLLYWFDWR

LPDGATQEELDMSEICGMTAYKKTPLLLVPSLYSP*

>CYP71AP1 cottonwood

MSLLQWLKECSKPTLFVVTIFLVVVLKFLMKDKLKKRKLNLPPSPAKLPIIGNLHQLGNM

PHISLRGLAKKYGPIIFLQLGEIPTVVISSAGLAKEVLKTHDLVLSSRPQLFSAKHLLYG

CTDIAFAPYGAYWRNIRKICILELLSAKRVRSYSYVREEEVARLIRRIAESYPGITNLSS

MIALYTNDVLCRVALGRDFSGGGEYDRHGFQKMFDDFQALLGGFSLGDYFPSMEFVHSLT

GMKSKLQYTFRRFDQFFDEVIAEHRSSKGKQEEKKDLVDVLLDIQKDGSSEIPLTMDNIK

AVILDMFAGGTDTTFITLDWAMTELIMNPHVMEKAQAEVRSVVGDRRVVQESDLPRLNYM

KAVIKEILRLHPAAPVLLPRESLEDVIIDGYNIPAKTRIYVNVWGMGRDPELWENPETFE

PERFMGSGIDFKGQDFELIPFGAGRRICPAITFGIATVEIALAQLLHSFDWKLPPGLEAK

DIDNTEAFGISMHRTVPLHVIAKPHFD*

>CYP71AQ1 cottonwood

MILHPYSLACLLFIFVTKWFFFNSARNKNLPPSPLKIPVVGNLLQLGLYPHRSLQSLAKR

HGPLMLLHLGNAPTLVVSSADGAHEILRTHDVIFSNRPDSSIARRLLYDYKDLSLALYGE

YWRQIRSICVAQLLSSKRVKLFHSIREEETALLVQNVELFSSRSLQVDLSELFSELTNDV

VCRVSFGKKYREGGSGRKFKKLLEEFGAVLGVFNVRDFIPWLGWINYLTGLNVRVEWVFK

EFDRFLDEVIEEFKANRVGVNEDKMNFVDVLLEIQKNSTDGASIGSDSIKAIILDMFAAG

TDTTHTALEWTMTELLKHPEVMKKAQDEIRRITGSKISVTQDDVEKTLYLKAVIKESLRL

HPPIPTLIPRESTKDVKVQGYDILAKTRVIINAWAIGRDPSSWENPDEFRPERFLESAID

FKGNDFQFIPFGAGRRGCPGTTFASSVIEITLASLLHKFNWALPGGAKPEDLDITEAPGL

AIHRKFPLVVIATPHSF*

>CYP71AR1 strawberry, wild

MAELINTETLSLVLLAVFLILFYIWSSSTSTTRNSPPSPPKLPIIGNLHQLLGSPGTPPH

RALQALSKLHGPLMLLHFGSFPVLVVSSAEAAREIMKTHDLAFASRPRTTAFEKLLYNYK

DVAAAPYGDYWRQVKSICVLNLLSAKKVRSFRTLREEETRSMINNIKETSRRGEVVDVRK

MVMGLTNDVVSRAALGKKYYNDGEFKELITEFTELAGSIHIGDYIPSLGWLSRLGGLDAK

LVSLAKRYDAFLDTVLQEHIDRSSETTSNRNDKSVDDQNEDNKDFVDVLLDIQRENSLHF

PLNRISIKAVVQDVFLAGTDTTSTLLEWAMAEILRHPRVMSKLQKELRSVKKGEEEILTE

DDMVDMHYLKAVIKEALRLHPPFTLLLPKMSIQDVKIKGYDIKANTQVLVNAWQIGRDPE

SFSYKPEEFEPEGFLEVNSGLSYKGTDFEFIPFGAGRRICPGIQFATTVNEIGLANLLHK

FDWKLPGGVRNEDLDMNESSGLTIHKKHPLKAVAIPYSSA

>CYP71AS1 Citrus sinensis

LLLIPLLLILKKLKAQNKQQLPPSPPKLPVIGNFHQLGELPHQSLWQLSKKYGPVMLLKL

GRVPLVVISSAEAARDVLKVHDLDCCSRPPLIGSGKFTYNYSDIAFSPYSDYWRELRKIS

VLEVFSLRRVQSFGFIREEEVALLMNSISESSSSASPVDLSEKMFALTGSIVFRMAFGRR

FRGSNFDNHSFQELVHAVESLLGGFAAAECFPYVGWIIDRLNGYHAKLERVFQELDTLFQ

QIIDDHLKPAETTKQEHVQQDIIDVMLKIERDQAESHESEAWLTKNHIKAVLLNIFLGGV

DTSAITVIWAMAELCKNPRLMKKAQAEIRNHIGNKGRVTEADIDQLQYLKMVIKETLRLH

PPAPLLIARDTLYRFKVNGYDIYPKTLIQVNAWAIGRDSKYWESPEEFIPERFIDKPVDV

KGQDFEYLPFGSGRRICPGINLGLIMSELALANLLYCFDWKLPNGREEDCVNMNMEEATG

VSLTLSKKTPLILVPVNYLQ

>CYP71AT1 Lycopersicon esculentum (tomato)

MILFLLFVALPIILIFVLPKAKKGAKNTQPPGPVGLPFIGNLHQFDSLTPHIYFWKLSKK

YGKIFSLKLGSTPMVVVSSAKLAKEVLKTQDLVYCSRPSILGQQKLSYNGRDIVFAPYND

YWREMRKISVLHLFSLKKVQLYKPIREDEVSRMIKKISLHAASSQITNLSNLMISLISTI

ICRFAFGVRFDDEAHERKRFDYLLAETQAMMASFFVSDVFPFLGWIDKLTGLTDRLKKNL

KELDEFYEELIEQHQNPNRPKSMEGDIVDLLLQLKKEKSIPIDLTLEDIKGLLMNVLVAG

SDTSAAGIVWTMTALMKNPKAMKKVQEEIRKSIGNKGIVNEDDIQNMPYFKAVIKESFRL

YPPVPLLVPRESMKKSTLEGYEIQAGTIVHVNSWAIARDPEIWENPEEFIPERFLNSDID

YKGQNYELIPFGAGRRGCPGMTLGVASMELALSNLLYAFDWELPHGMKKEDIDTNVRPGI

TMHKKNDLCLIPKSYF*

>CYP71AU2 Lycopersicon esculentum (tomato)

MISLFAVFPFLIFLGFILLSFFQLSSKKSKKNLPPSPPKLPLIGNFHQLGQQPHRSLQKL

TNEYGPMMMLQFGSVPVLIASSAEAASHIMKTQDLGFANKPKSIIPSKLFFGPKDVAFTP

YGEYWRNARSVCMLQLLNNKRVQSFSKIREEETSLLLRKINHSIGNSQVVDLTDLFVSMT

NDVLCRVALGRKYCDGEEGKKFKSLLLEFVELLGVFNIGDYMPWLAWVNRFNGLNAKVDK

VAEEFSAFLEGVIEEHKEKIKTDEKEEGSADFVDILLQVQKENKSGFNVEMDSIKAIIMD

MFSAGTDTTSTLLEWTMNELIRNPNALRKLRDEVRKVTQGKSDVTEDDLEHMPYLNAVMK

ESLRLHSPVPLLPREAIKDTKVLGYDVAAGTQVFVCPWAISRDPTIWENPEEFQPERFLD

SCVDYKGLHFELIPFGAGRRGCPGITFAKVVNELALARMLFHFEFSLPNGAKAEDLDVDE

ALGITVRRKFPLLVVATPRI*

>CYP71AV1 clone name 71DA4 Artemisia annua L. (Sweet Annie, an asterid)

MKSILKAMALSLTTSIALATILLFVYKFATRSKSTKKSLPEPWRLPIIGHMHHLIGTTPH

RGVRDLARKYGSLMHLQLGEVPTIVVSSPKWAKEILTTYDITFANRPETLTGEIVLYHNT

DVVLAPYGEYWRQLRKICTLELLSVKKVKSFQSLREEECWNLVQEIKASGSGRPVNLSEN

VFKLIATILSRAAFGKGIKDQKELTEIVKEILRQTGGFDVADIFPSKKFLHHLSGKRARL

TSLRKKIDNLIDNLVAEHTVNTSSKTNETLLDVLLRLKDSAEFPLTSDNIKAIILDMFGA

GTDTSSSTIEWAISELIKCPKAMEKVQAELRKALNGKEKIHEEDIQELSYLNMVIKETLR

LHPPLPLVLPRECRQPVNLAGYNIPNKTKLIVNVFAINRDPEYWKDAEAFIPERFENSSA

TVMGAEYEYLPFGAGRRMCPGAALGLANVQLPLANILYHFNWKLPNGVSYDQIDMTESSG

ATMQRKTELLLVPSF

>CYP71AX1 Lycopersicon esculentum (tomato)

YFLLVPLLAFIYFLHQCFFSPSNTQKRLLPPSPTKLPIIGNLHQLGSLPHRSLHKLSKKY

GPVMLLHLGSKPVIIASSVDAARDIMKTHDLVWSNRPKSSMADGLFYGSKDVTFSPYGEY

WRQIRSITVLHLLSNKRVQSYRRVREEEISNMIDKIRQKCDSVIDLRDVFSCLANNIISR

VNIGRTYNEGECGIAVKSLIEELLILIGTFNIGDYTPWFKWVNKIKGVDSRVKKVAKDLD

AFIESVIEERLIRNKKAECSAVEAKDFLGVLLEIQDGKETGFPLQRDSLKALLLDAFVAG

TDSTYTVLEWTMTELLRHPRVMTKLEDEVRELGQGKTEITEDDLRNMHYLKAVIKESLRL

HAPVPLLVARESMEEVKLLDYDIPAKTEVLINAWSIGRDPLLWDHPEEYMPERFLSSDID

VKGLNFELIPFGAGRRGCPGIPFAIMVNELALANLVYKFNFALPKGIKGEDLDMTECNGL

AVRRKSPLLVVATPKSMV*

>CYP71AY1 Catharanthus roseus (Madagascar periwinkle)

ARVNFSLTSPIFLLLSSLFLIILLNKLMRGNKIQKGKKLPPGPKKIAIIGNLPSNGRFTS

LIVFLNNLAEKYGPIMHLRIGQLSAVIISSAEKAKEILNTHGVRVADRPQTTVAKIMLYN

SLGVTFAPYGDYLKQLRQIYAMELLSPKTVKSFWTIMDDELSTMITSIKSEVGQPMILHD

KMMTYLYAMLCRATVGSVCNGRETLIMAAKETSALSASIRIEDLFPSVKILPVISGLKSK

LTNLLKELDIVLEDIISAREKKLLSQPQQPLMLDEEDMLGVLLKYKNGKGNDTKFRVTNN

DIKAIVFELILAGTLSSAAIVEWCMSELMKNPELLKKAQDEVRQVLKGKKTISGSDVGKL

EYVKMVVKESVRLHPPAPLLFPRECREEFEIDGMTIPKKSWVIINYWAIGRDPKIWPNAD

KFEPERFSNNNIDFYGSNFELIPFGAGRRVCPGILFGTTNVELLLAAFLFHFDWELPGGM

KPEELDMNELFGAGCIRENPLCLIPSISTVVEGN

>CYP71AZ1 Ammi majus

MQMDAVVILLILAFPIASVYVLFYHKKRVDGLSEPPGPPGLPFIGNFYQLYKAPCIHEYL

CTLSKRYGSLMTLRMGSVPILVVSSPKMAKEVLKTQDLAYCSRPMMTGMQKLSYNGLDVA

FSPYSEHWRQVRKFCTLELFTQKRAQIDFRHVHEQEVSRMIARLSETAAASKDVNAFECF

SNLATSIISRVAFGKRHDEDGIGKERLQRMLSELDTMLSVYFVSDFFPMFGWIDSLTGMR

ARLDRTFKEMDMFYEELIDDHLKPDRPESLTEDIIDVMLKNKGCSSSSLTKDTMKAILLN

VFNGGTGTSASLLVWAMTALMRNRGVMKKVQEEIRSVIGKKGNVDEDDIQNLPYLRAVVK

ETMRLYPTGALLIPRKTIESSIIGEDKDHMYMIKPKTLVYVSMWAIGRDPEIWKNPMKFV

PERFLERHDINYQGQQFEYIPFGAGRRICPGIHLGLTTVELALANLLYTFNWEPPVGTRF

EDINDETVNGITLQKKNALYIRPKTYMFS*

>CYP71BC1 Vitis vinifera (Pinot noir grape)

MTMKISENMLLLFSQSSANQWLLALGILSFPILYLFLLQRWKKKGIEGAARLPPSPPKLP

IIGNLXQLGKLPHRSLSKLSQEFGPVLLLQLGRIPTLLISSADMAKEVLKTHDIDCCSRA

PSQGPKRLSYNFLDMCFSPYSDYWRAMRKVFVLELLSAKRAHSLWHAWEVEVSHLISSLS

EASPNPVDLHEKIFSLMDGILNMFAFGKNYGGKQFKNEKFQDVLVEAMKMLDSFSAEDFF

PSVGWIIDALTGLRARHNKCFRNLDNYFQMVVDEHLDPTRPKPEHEDLVDVLLGLSKDEN

FAFHLTNDHIKAILLNTFIGGTDTGAVTMVWAMSELMANPRVMKKVQAEVRSCVGSKPKV

DRDDLAKLKYLKMVVKETFRMHPAAPLLIPHRTRQHCQINANGCTYDIFPQTTILVNAFA

IGRDPNSWKNPDEFYPERFEDSDIDFKGQHFELLPFGAGRRICPAIAMAVSTVEFTLANL

LYCFDWEMPMGMKTQDMDMEEMGGITTHRKTPLCLVPIKYGCVE*

>CYP71BE1 Vitis vinifera (Pinot noir grape)

MEFPSSFLFPFLLFLFILFKVSKKSKPQISIPKRPPGPWKLPLIGNLHQLVGSLPHHSLR

DLAKKYGPLMHLQLGQVSMLVVSSPEIAKEVMKTHDINFAQRPHLLATRIATYDSTDVAF

SPYGDYWRQLRKICVVELLSAKRVKSFQVIRKEEVSKLIRIINSSSRFPINLRDRISAFT

YSVISRAALGKECKDHDPLTAALGEITKLASGFCLADLYPSVKWIPLVSGVRHKLEKVQQ

RIDGILQIVVDEHRERMKTTTGKLEEEKDLVDVLLKLQQDGDLELPLTDDNIKAVILDIF

GGGGDTVSTAVEWTMAEMMKNPEVMKKAQAEVRRVFDGKGNVDEAGIDELKFLKAVISET

LRLHPPFPLLLPRECREKCKINGYEVPVKTRVVINAWAIGRYPDCWXEAERFYPERFLDS

SIDYKGADFGFIPFGSGRRICPGILFGIPVIELPLAQLLFHFDWKLPNGMRPEDLDMTEV

HGLAVRKKHNLHLIPIPYSPLTVG*

>CYP71BF1 Carica papaya

MWNSLWFTVLFIFLFRYFFLRCYSSSKKNSPPSPPKLPIVGDLHRLGSSPHRSLRALAQQ

YGPFMLLRFGSVPVLVISSAHAALDVMKTHDNIFSSRFKSSVFDKLVYNCKDVVLAPYGE

YWRQMKSICVIHLLSSKKVQCFQKVREEETMILTKKIQESYCSPMNLSETFTVLANDILC

RVAFGRKYGGDEENGKKLKELLTRFAQLTGTVDIGDYIPWLSWVSCVNGLNTKLEKLAKE

LDDLFEGIVEEHVNHLKNKSNTSDYGDVQDTDSKDFVDVLLWIQRENTIGFPIDRVTIKA

LILDMFIAGTDTTSTTLEWAMTELLRHPKVMKKLRNEVRTIAGDKSNIIEEDLGKMKYLK

AVIKEVLRLHPPLPLLMPRESVKDANVRGYDITAGTQVLINAWAIGRDPDSWEEAEEFKP

ERFLNSCIDFKGHDFQLIPFGAGRRGCPGIHFATILIEMILANVLHKCDWKPMPYRGAKE

HGIDLTESAGISVHRKFPLIAIPSPPRF*

>CYP71BG1 Solanum tuberosum

MEASILQLLLLLSLTSCTILFYKIRRWRRPPSPPSLPIIGHLHLLTDMPHHTFFHLSQKL

GPIIHLQLGQIPTLIISSPRLAELILKTNDHIFCSRPQIIAAQYLSFGCSDITFSPYGPY

WRQARKICVTELLSSKRVNSFQFIRNEEINRMIQLISSHFDSELSSELDLSQVFFALAND

ILCRVAFGKRFIDDRLKDKDLVSVLTETQALLAGFCLGDFFPDWEWVNWLSGMKKRLMNN

LKDLGEVCDEIIDEHLMKKRDDDQNGDGSEDFVDVLLRVQKRDDLQVPITDDNLKALILD

MFVAGTDTSAATLEWTMTELARHPSVMKKAQDEVREIAANKGKVEEFDLQHLHYMKAVIK

ETMRLHPPVPLLVPRESIEKCTLDDYEIPAKTRVLINTYAIGRDPEYWNNPLDYNPERFM

EKDIDFRGQDFRFLPFGGGRRGCPGYALGLATIELSLARLLYHFDWKLPTGVEAQDVNLS

EIFGLATRKRVALKLVPTINKLYLLSD*

>CYP51G1 sterol 14-demethylase [Arabidopsis thaliana]

MELDSENKLLKTGLVIVATLVIAKLIFSFFTSDSKKKRLPPTLKAWPPLVGSLIKFLKGP

IIMLREEYPKLGSVFTVNLVHKKITFLIGPEVSAHFFKASESDLSQQEVYQFNVPTFGPG

VVFDVDYSVRQEQFRFFTEALRVNKLKGYVDMMVTEAEDYFSKWGESGEVDIKVELERLI

ILTASRCLLGREVRDQLFDDVSALFHDLDNGMLPISVLFPYLPIPAHRRRDRAREKLSEI

FAKIIGSRKRSGKTENDMLQCFIESKYKDGRQTTESEVTGLLIAALFAGQHTSSITSTWT

GAYLMRYKEYFSAALDEQKNLIAKHGDKIDHDILSEMDVLYRCIKEALRLHPPLIMLMRA

SHSDFSVTARDGKTYDIPKGHIVATSPAFANRLPHIFKDPDTYDPERFSPGREEDKAAGA

FSYIAFGGGRHGCLGEPFAYLQIKAIWSHLLRNFELELVSPFPEIDWNAMVVGVKGNVMV

RYKRRQLS

>CYP51G sterol 14 desaturase [Chlamydomonas reinhardtii]

MDLPPELAVLADKVLSLSPVVLVALGSAVLILALAVGRVLFNLLPSKRPPVWEGLPFIGG

LLKFTGGPWKLLENGYAKFGECFTVPVAHRRVTFLIGPEVSPHFFKAGDDEMSQSEVYDF

NIPTFGRGVVFDVEQKVRTEQFRMFTEALTKNRLKSYVPHFNKEAEEYFAKWGETGVVDF

KDEFSKLITLTAARTLLGREVREQLFDEVADLLHGLDEGMVPLSVFFPYAPIPVHFKRDR

CRKDLAAIFAKIIRARRESGRREEDVLQQFIDARYQNVNGGRALTEEEITGLLIAVLFAG

QHTSSITTSWTGIFMAANKEHYNKAAEEQQDIIRKFGNELSFETLSEMEVLHRNITEALR

MHPPLLLVMRYAKKPFSVTTSTGKSYVIPKGDVVAASPNFSHMLPQCFNNPKAYDPDRFA

PPREEQNKPYAFIGFGAGRHACIGQNFAYLQIKSIWSVLLRNFEFELLDPVPEADYESMV

IGPKPCRVRYTRRKL
