# Supplementary material for: CYP96T1 of Narcissus sp. aff. pseudonarcissus Catalyzes Formation of the Para-Para' C-C Phenol Couple in the Amaryllidaceae Alkaloids
Source: Front Plant Sci. 2016 Feb 25;7:225. doi: 10.3389/fpls.2016.00225 (PMC4766306; doi:10.3389/fpls.2016.00225)
Supplement: Supplementary file 2 [file Table2.DOCX]

Supplementary Material

# CYP96T1 of *Narcissus* sp*. aff. pseudonarcissus* Catalyzes Formation of the *Para-Para’* *C-C* Phenol Couple in the Amaryllidaceae Alkaloids

**Matthew B. Kilgore, Megan M. Augustin , Gregory D. May, John A. Crow, Toni M. Kutchan^*^**

^*^**Correspondence:** Toni M. Kutchan: [tkutchan@danforthcenter.org](mailto:tkutchan@danforthcenter.org)

**Table S1. *N.* sp. *aff. pseudonarcissus* cytochrome P450s co-expressing with *N4OMT***

| **Contig number** | **Family of closest BLASTP hit** | **Accession number of closest BLASTP hit** | **Query coverage (%)** | **Percent identity (%)** | **Assemblies with a *N4OMT* co-expressing homologue** |
| --- | --- | --- | --- | --- | --- |
| **medp_9narc_20101112\|12135** | CYP71 | XP_008779328.1 | 99 | 56 |  |
| **medp_9narc_20101112\|12202** | CYP90 | XP_008805787.1 | 82 | 70 |  |
| **medp_9narc_20101112\|1292** | CYP81 | XP_010906301.1 | 91 | 46 |  |
| **medp_9narc_20101112\|13806** | CYP73 | AAS48416.1 | 93 | 57 |  |
| **medp_9narc_20101112\|14090** | CYP704 | XP_010934572.1 | 99 | 69 |  |
| **medp_9narc_20101112\|17246** | CYP88 | XP_010926147.1 | 100 | 60 | *Galanthus* sp. ABySS and MIRA |
| **medp_9narc_20101112\|2134** | CYP86 | XP_010911437.1 | 98 | 47 |  |
| **medp_9narc_20101112\|22907** | CYP86 (CYP96 on closer examination) | [XP_010911437.1](http://www.ncbi.nlm.nih.gov/protein/743891946?report=genbank&log$=prottop&blast_rank=1&RID=9GRR9PHN01R) | 98 | 58 | *Galanthus* sp. ABySS and MIRA, *Galanthus elwesii* ABySS and MIRA, *Galanthus* sp. Trinity, *Galanthus elwesii* Trinity |
| **medp_9narc_20101112\|2593** | CYP95 | XP_009381127.1 | 95 | 53 | *Galanthus* sp. ABySS and MIRA |
| **medp_9narc_20101112\|2772** | CYP71 | XP_010911323.1 | 97 | 53 | *Galanthus* sp. ABySS and MIRA |
| **medp_9narc_20101112\|2865** | CYP71 | XP_010911323.1 | 96 | 44 |  |
| **medp_9narc_20101112\|32696** | CYP86 | XP_009397903.1 | 93 | 48 | *Galanthus* sp. ABySS and MIRA and *Galanthus* *elwesii* Trinity |
| **medp_9narc_20101112\|39515** | CYP86 | AJD25207.1 | 100 | 94 |  |
| **medp_9narc_20101112\|40939** | CYP88 | XP_010934998.1 | 96 | 83 |  |
| **medp_9narc_20101112\|5034** | CYP88 | XP_008797526.1 | 96 | 69 | *Galanthus* sp. ABySS and MIRA |
| **medp_9narc_20101112\|5391** | CYP72 | XP_008784485.1 | 99 | 54 |  |
| **medp_9narc_20101112\|63732** | CYP71 | XP_010905756.1 | 96 | 58 | *Galanthus elwesii* ABySS and MIRA |
| **medp_9narc_20101112\|9161** | CYP81 | XP_008787116.1 | 88 | 59 |  |
